# Oxyenamides as Versatile Building Blocks for a Highly Stereoselective One‐Pot Synthesis of the 1,3‐Diamino‐2‐ol‐Scaffold Containing Three Continuous Stereocenters

**DOI:** 10.1002/anie.202109752

**Published:** 2021-09-28

**Authors:** Sara‐Cathrin Krieg, Jennifer Grimmer, Philipp Kramer, Michael Bolte, Harald Kelm, Georg Manolikakes

**Affiliations:** ^1^ Department of Chemistry Technical University Kaiserslautern Erwin-Schrödinger-Strasse Geb. 54 67663 Kaiserslautern Germany; ^2^ Institute of Inorganic and Analytical Chemistry Goethe University Frankfurt am Main Max-von-Laue-Strasse 7 60438 Frankfurt am Main Germany

**Keywords:** 1,3-diamine, enamides, Lewis acid, one-pot reaction, stereoselective synthesis

## Abstract

A highly diastereoselective one‐pot synthesis of the 1,3‐diamino‐2‐alcohol unit bearing three continuous stereocenters is described. This method utilizes 2‐oxyenamides as a novel type of building block for the rapid assembly of the 1,3‐diamine scaffold containing an additional stereogenic oxygen functionality at the C2 position. A stereoselective preparation of the required (Z)‐oxyenamides is reported as well.

## Graphical abstract

2‐Oxyenamides are introduced as novel, highly versatile building blocks for the rapid construction of the 1,3‐diamino‐2‐ol scaffold. A Lewis‐acid‐mediated reaction of 2‐oxyenamides with acylimine precursors and a terminal nucleophile enables a modular assembly of the 1,3‐diamino‐2‐ol structure bearing three continuous stereocenters with excellent levels of diastereoselectivity.

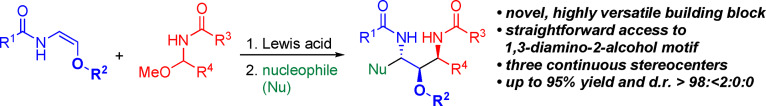

The synthesis of acyclic molecules containing multiple stereogenic centers in a rapid manner with precise control over all formed stereocenters still represents a formidable challenge for any organic chemist.^[1]^ Usually a stepwise synthesis, viz. the creation of a single stereocenter and/or a single carbon−carbon bond in one chemical step, offers a reliable access to the desired scaffold. However, such a stepwise construction will result in a time‐ and resource‐intensive route. Therefore, the controlled synthesis of several bonds and stereocenters in a simple one‐pot operation is receiving increasing attention as an attractive and more efficient alternative for the construction of structurally complex molecules.[^2^, ^3^] The 1,3‐diamino‐2‐alcohol unit represents such a structurally complex scaffold. This moiety contains three adjacent functional groups attached to three continuous stereocenters. The 1,3‐diamino‐2‐alcohol motif can be found in various drugs or natural products, for example, the bromopyrrole alkaloid manzacidin B^[4]^ (Figure 1). Interestingly, several HIV‐protease inhibitors, such as fosamprenavir, amprenavir and nelfinavir, contain this core motif.^[5]^ The preparation of such molecules usually requires a multistep synthesis. In the last years several groups have shown that enamides or encarbamates are highly useful building blocks for a rapid and stereocontrolled construction of the parent 1,3‐diamine unit (Scheme 1 a).[^6^, ^7^] However, the highly relevant 1,3‐diamino‐2‐alcohol motif cannot be accessed directly with these methods. We envisioned that starting from the corresponding oxyenamides of type **1**, one should be able to directly access the 1,3‐diamino‐2‐ol core structure in a similar manner (Scheme 1 b). However, reactions with oxyenamides have been scarcely reported so far.^[8]^ Indeed, even methods for their synthesis are rare.^[9]^ Considering the potential utility of oxyenamides not only as building block for the construction of the 1,3‐diamino‐2‐alcohol unit, but as a general tool for the stereoselective synthesis of the 1,2‐aminoalcohol scaffold, a systematic study on their synthesis and application would be highly desirable. Herein we describe a first uniform approach for the stereoselective synthesis of (*Z*)‐oxyenamides and their application in a one‐pot transformation for the construction of the 1,3‐diamino‐2‐alcohol substructure (Scheme 1 b). This experimentally facile, sequential one‐pot operation offers a rapid and highly stereoselective access to the 1,3‐diamino‐2‐ol motif with up to three continuous stereocenters.

**Figure 1:**
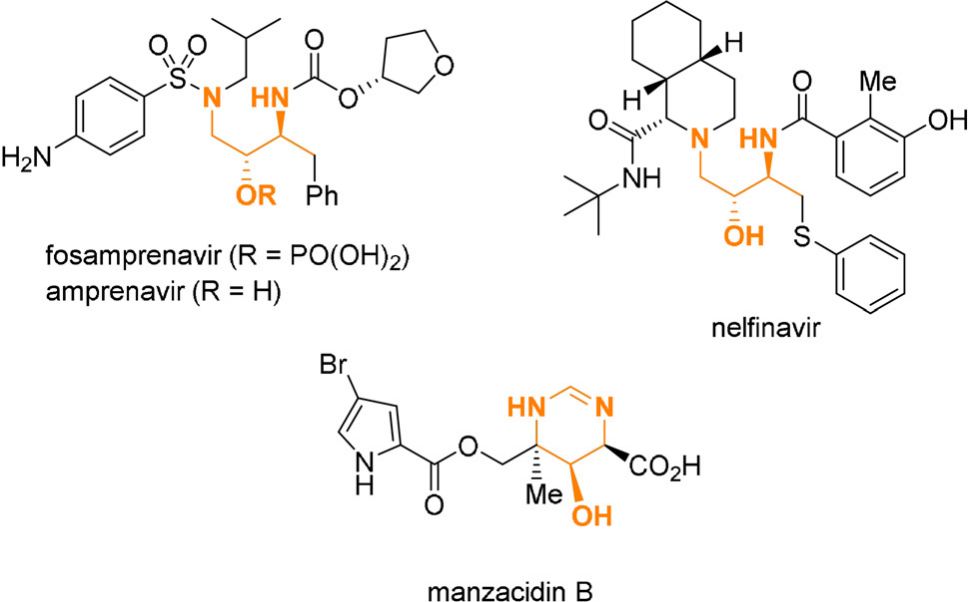
Biologically active 1,3‐diamino‐2‐alcohols.

**Scheme 1:**
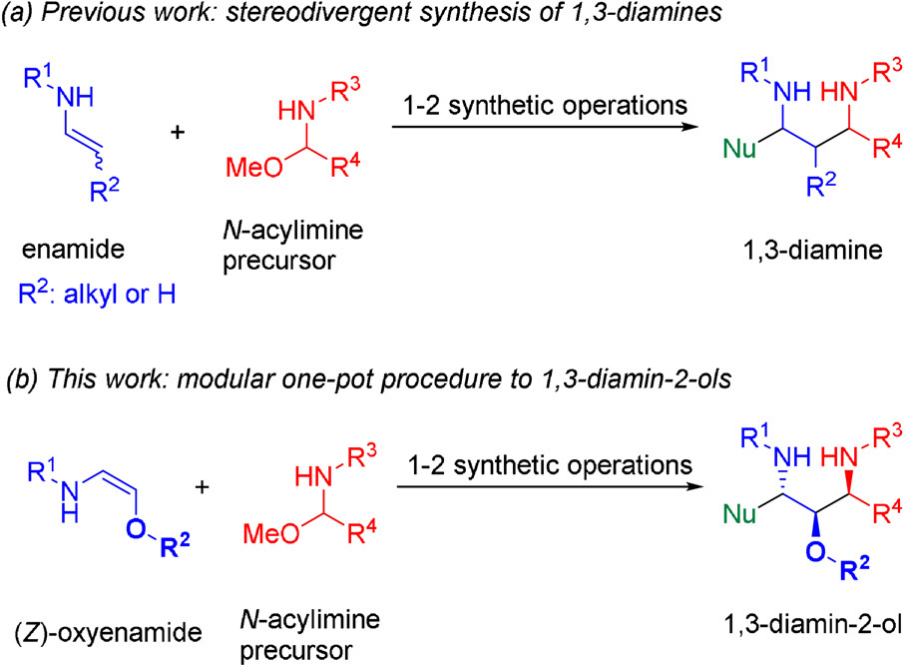
Established procedures for the assembly of 1,3‐diamines from enamides and the analogous synthesis of 1,3‐diamino‐2‐alcohol scaffold from 2‐oxyenamides.

At the onset of our studies, we decided to investigate the synthesis and application of vinyl ester‐type enamides (**1**) due to the following reasons. An electron‐withdrawing residue on the oxygen atom should render the enamide moiety more nucleophilic than the enol ether/ester functionality embedded in the same molecule.^[10]^ Thereby, a chemoselective reaction with electrophiles at the β‐carbon (highlighted in blue) can be expected (Scheme 2).^[11]^ This type of compounds should be readily accessible from the corresponding protected amino aldehydes **2**, which leads back to 2‐aminoethanol as common starting material. Furthermore, the incorporated ester functionality should enable a facile liberation of the free alcohol functionality in the final product.

**Scheme 2:**
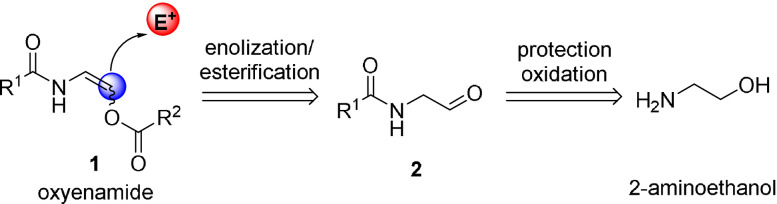
Retrosynthetic rationale towards ester‐protected oxyenamides and their expected reactivity.

To our delight, oxyenamides of type **1** could be synthesized in three steps using the envisioned approach. Selective acylation of the amine functionality followed by alcohol oxidation afforded the N‐protected α‐amino aldehydes in 63–64 % overall yield (Scheme 3 a). Treatment of the aldehydes **2 a**–**c** with a carboxylic acid chloride in the presence of NEt_3_ afforded the desired oxyenamides (**1**) in 56–73 % yield. In all cases exclusive formation of the (*Z*)‐isomer was observed (*E*/*Z* ≤2:98). We assume that stabilization of the (*Z*)‐enolate via intramolecular hydrogen bonding leads to the observed stereoselective formation of the (*Z*)‐oxyenamides (Scheme 3 b). Using this approach, the benzoyl‐, pivaloyl‐ and acetyl‐protected oxyenamides **1 a**–**c** as well as the Boc‐ and the Cbz‐protected enecarbamates **1 d** and **1 e** could be prepared in only three steps from 2‐aminoethanol. We have utilized this streamlined procedure for the routine synthesis of oxyenamides of type **1** on a 1 g scale. With sufficient quantities of the oxyenamides (**1**) at hand, we started to explore their application in the construction of the 1,3‐diamino‐2‐alcohol scaffold. Therefore, the oxyenamides (**1**) were reacted with acylimine precursor **3 a** in the presence of different Lewis acids (Scheme 4).

**Scheme 3:**
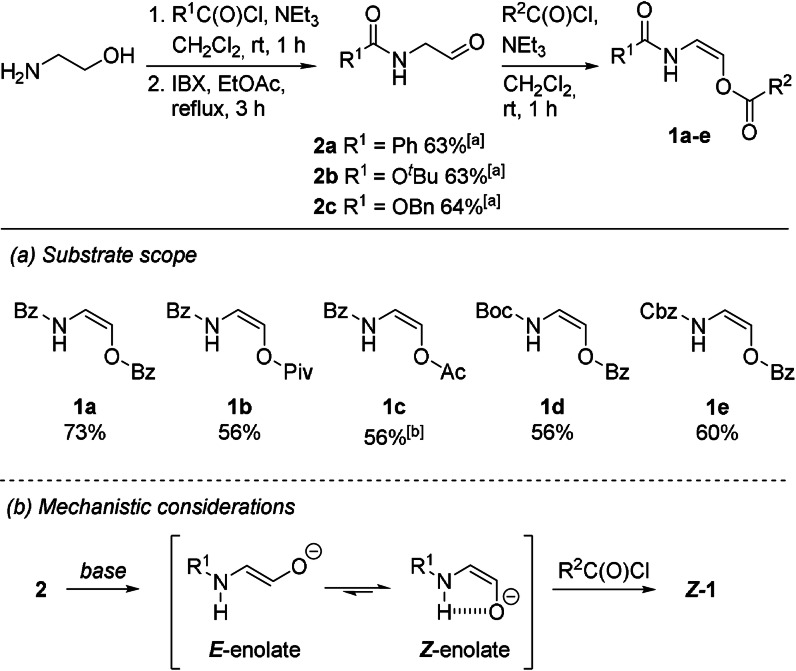
Synthesis of oxyenamides of type **1**. Given yields refer to isolated yield of the analytically pure product [a] Yield over two steps. Bz=benzoyl; Piv=pivaloyl; Ac=acetyl; Boc=*tert*‐butoxycarbonyl; Cbz=benzyloxycarbonyl.

**Scheme 4:**
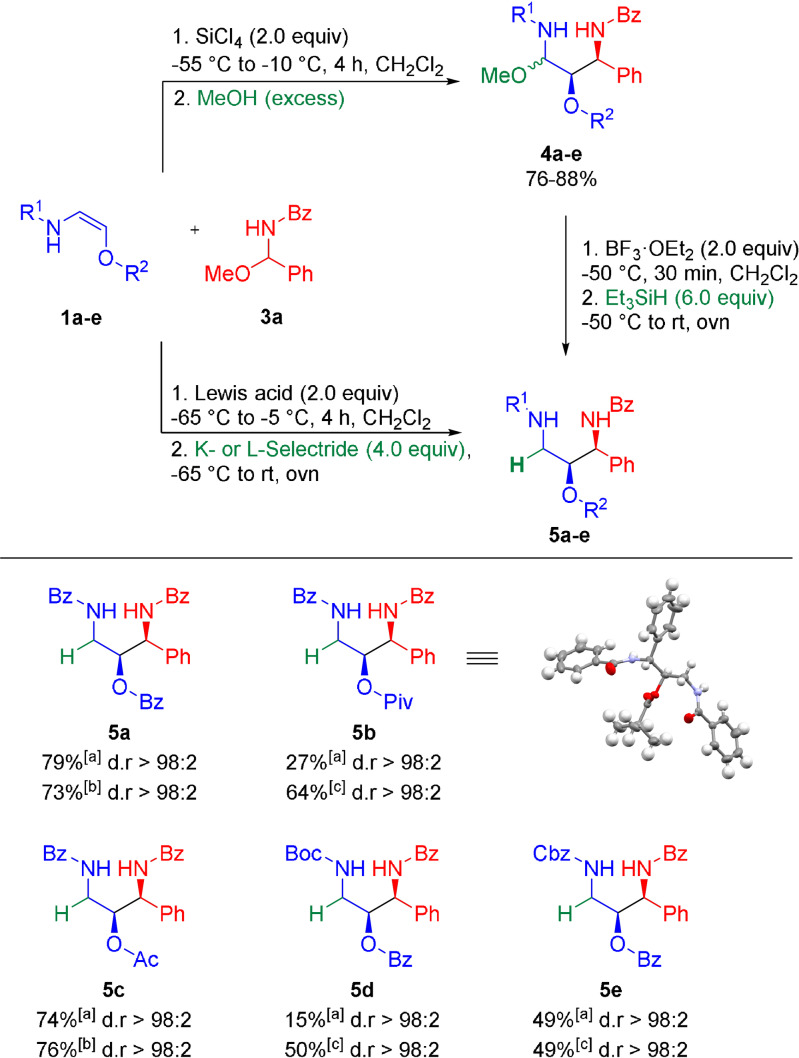
Addition–reduction sequence (both sequential and one‐pot). Given yields refer to isolated yield of the major diastereomer; The reported diastereomeric ratio (d.r.) refers to the diastereomeric ratio of the crude reaction mixture as determined by ^1^H NMR. [a] From reduction of the *N*,*O*‐acetal. [b] Via one‐pot reaction with SiCl_4_ and K‐Selectride. [c] Via one‐pot reaction with BF_3_⋅OEt_2_ and L‐Selectride.

Although a variety of Lewis acids could mediate this transformation, best results were obtained with SiCl_4_. The desired addition products **4 a**–**e** were obtained in 76–88 %.^[12]^ Reduction of the newly formed *N*,*O*‐acetals (**4**) with Et_3_SiH in the presence of BF_3_⋅OEt_2_ furnished the 1,2‐*syn*‐1,3‐diamino‐2‐alcohol products **5 a**–**e** in varying yields (15–79 %) and with excellent diastereoselectivties (d.r. ≥98:2).^[13]^ In general, better yields were obtained with a modified one‐pot protocol without isolation of the intermediates of type **4**. Reaction of the oxyenamides (**1**) with acylimine precursors **3 a** in the presence of SiCl_4_ or BF_3_⋅OEt_2_, followed by direct addition of either K‐Selectride (for SiCl_4_) or L‐Selectride (for BF_3_⋅OEt_2_) afforded the desired 1,3‐amino‐2‐alcohols **5 a**–**e** in 49–76 % yield with excellent diastereoselectivies. In all cases only the 1,2‐*syn* diastereomer could be observed in the crude reaction mixture (d.r. ≥98:2). These results demonstrate that oxyenamides of type **1** show a reactivity profile similar to their β‐carbon‐substituted counterparts and can be used as building blocks for stereoselective transformations. Therefore, we turned our attention towards the stereoselective construction of 1,3‐diamino‐2‐alcohols containing three continuous stereogenic centers. Accordingly, the reducing agent was replaced with 1,3,5‐trimethoxybenzene as terminal nucleophile (Scheme 5). To our delight, this modified reaction directly afforded the 1,2‐*syn*‐2,3‐*anti*‐configurated products **6 a**–**e** in 58–90 % yield in a simple one‐pot operation. In case of oxyenamides **1 a**–**c** the reaction proceeded with excellent stereoselectivities, furnishing the products **6 a**–**c** essentially as a single diastereomer (d.r. >98:<2:0:0). In case of the Cbz‐derived encarbamate (**1 e**) a lower diastereoselectivity (d.r.=71:29:0:0) was observed. For the Boc‐protected oxyenamide **1 d**, only trace amounts of the product could be detected. Presumably, a prolonged stirring of intermediate **4 d** in the presence of SiCl_4_ leads to cleavage of the Boc group and side reactions with the free amine. In a similar manner, other nucleophilic components could be utilized in this one‐pot process (Scheme 6). Reactions with different electron‐rich arenes or heteroarenes lead to the formation of the 1,2‐*syn*‐2,3‐*anti*‐1,3‐diamino‐2‐alcohols **7 a**–**h** with three continuous stereocenters in 69–87 % yield with uniformly high diastereoselectivities. Heterocycles, such as indole, furan or methoxythiophene, performed particularly well. In most cases only the formation of a single diastereomer could be observed. For some reactive heterocycles the desired products (**7 e**, **7 g** and **7 h**) were obtained with slightly lower stereoselectivties. The reaction with pyrazole afforded the N‐alkylated product **7 i** in 81 % yield and with a diastereomeric ratio of 87:13. Employing NaN_3_ or EtSH as terminal nucleophile furnished the products **7 j** and **7 k**, containing a useful handle for further transformations, in 57 % and 83 % yield, albeit with slightly lower diastereoselectivities. So far, the final trapping with a terminal nucleophile is mainly limited to electron‐rich (hetero)arenes. In case of less reactive nucleophiles (e.g. anisole or allylsilane), we did only observe decomposition of the intermediates of type **4** upon prolonged stirring at temperatures >0 °C.

**Scheme 5:**
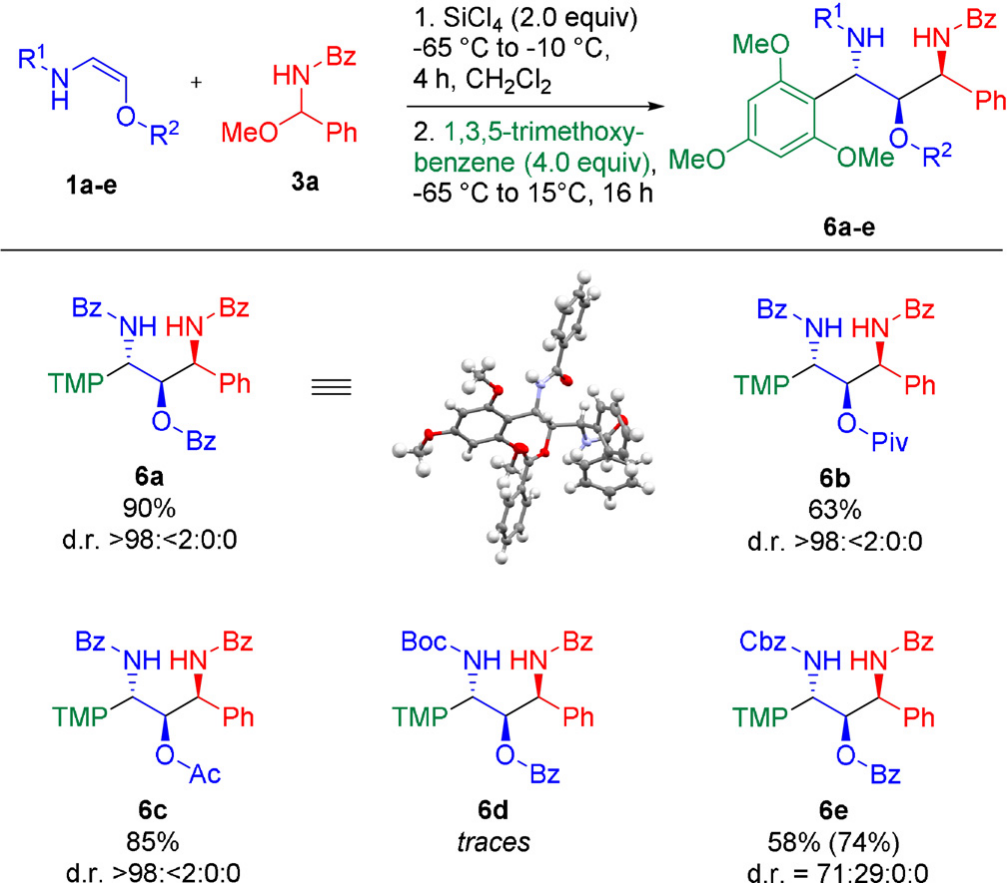
One‐pot reaction with 1,3,5‐trimethoxybenzene. Given yields refer to the isolated yield of the major diastereomer. Values in parentheses represent the overall isolated yield of all diastereomers. The reported diastereomeric ratio (d.r.) refers to the diastereomeric ratio of the crude reaction mixture as determined by ^1^H NMR (TMP=1,3,5‐trimethoxyphenyl).

**Scheme 6:**
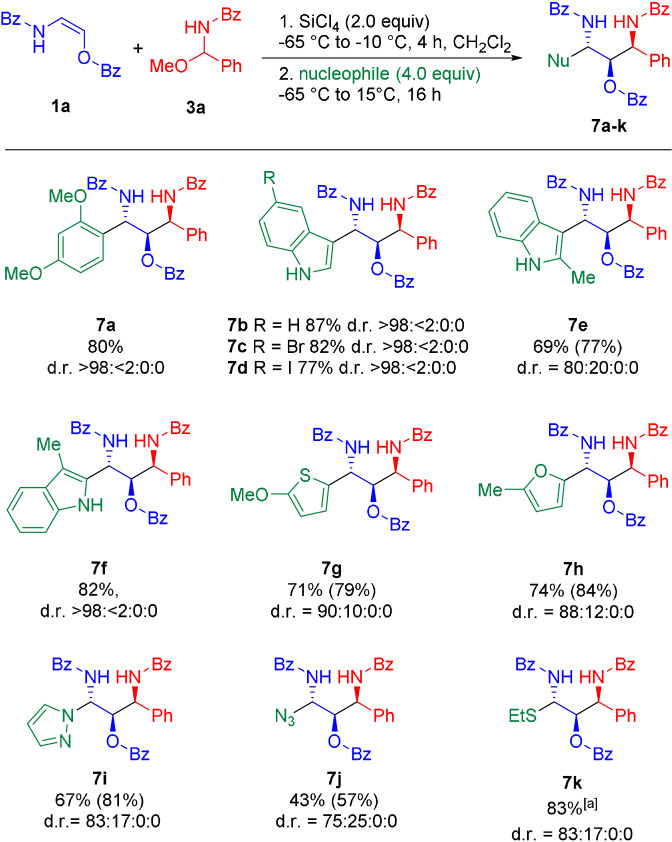
One‐pot reaction with different nucleophiles. Given yields refer to the isolated yield of the major diastereomer. Values in parentheses represent the overall isolated yield of all diastereomers. The reported diastereomeric ratio (d.r.) refers to the diastereomeric ratio of the crude reaction mixture as determined by ^1^H NMR. [a] Overall yield for both diastereomers, no separation of diastereomers could be achieved in the case of **7 k**.

Next, we investigated reactions with different *N*‐acylimine precursors of type **3** (Scheme 7). In general, *N*,*O*‐acetals derived from aromatic aldehydes proved to be suitable starting materials for our one‐pot approach, leading to the formation of the 1,2‐*syn*‐2,3‐*anti*‐configured products **8 a**–**i** in 55–95 % yield with excellent diastereoselectivities in all cases (d.r. >98:<2:0:0). Different electron‐withdrawing or ‐donating substituents as well as different substitution patterns were well tolerated. To our delight, also a Cbz‐derived carbamoyl imine precursor reacted smoothly, affording the orthogonally protected 1,3‐diamine‐2‐ol **8 h** in 56 % yield and perfect diastereoselectivity. Unfortunately, reactions with alkyl aldehyde‐derived as well as heterocyclic *N*,*O*‐acetals did not furnish any desired product under the standard conditions.

**Scheme 7:**
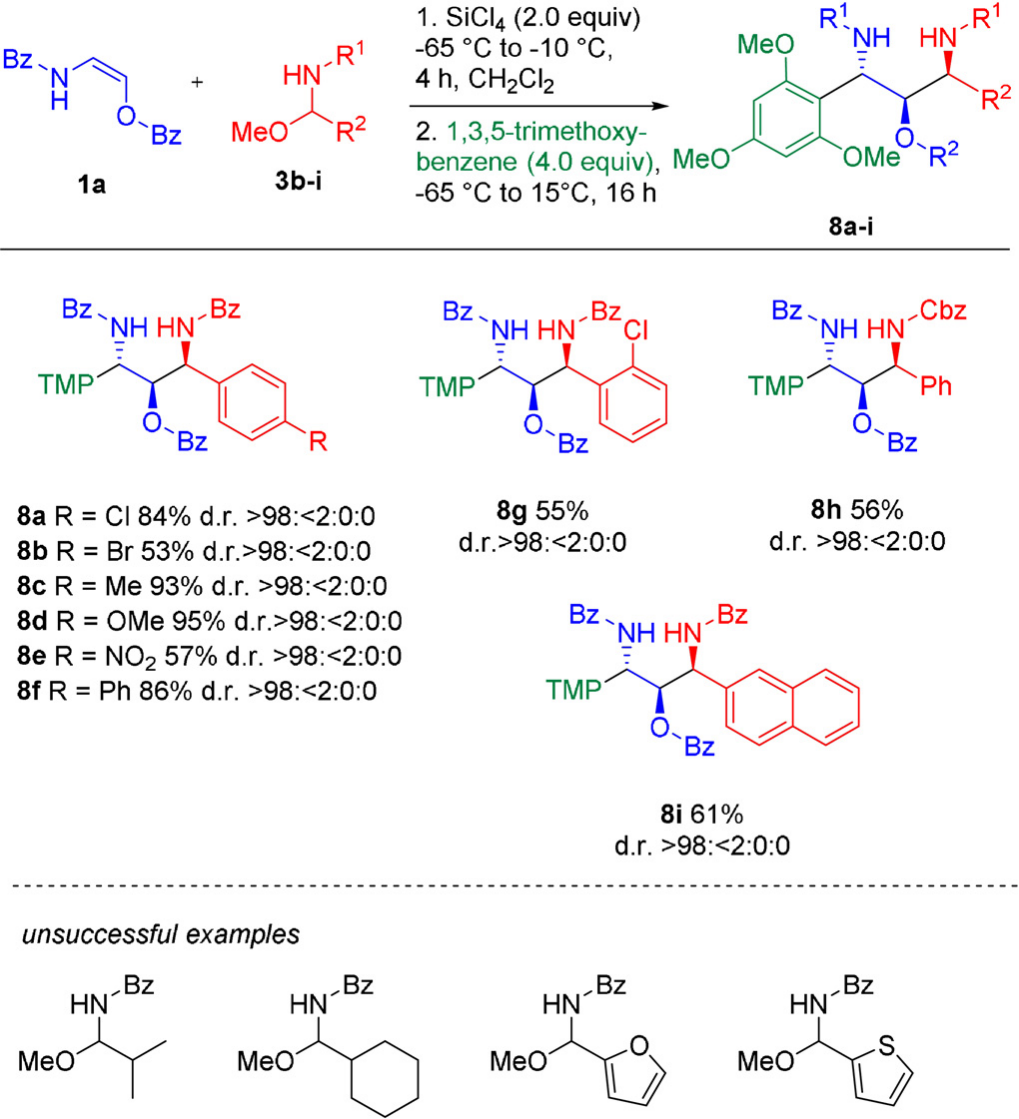
One‐pot reaction with different imine precursors. Given yields refer to the isolated yield of the major diastereomer. The reported diastereomeric ratio (d.r.) refers to the diastereomeric ratio of the crude reaction mixture as determined by ^1^H NMR (TMP=1,3,5‐trimethoxyphenyl).

Finally, we investigated the deprotection of the introduced masked alcohol functionality on two selected examples. Removal of the benzoyl group with sodium methoxide in MeOH^[14]^ proceeded smoothly, affording the unprotected 1,3‐diaminoalcohols **9 a** and **9 b** in high yields with complete retention of configuration (Scheme 8).

**Scheme 8:**
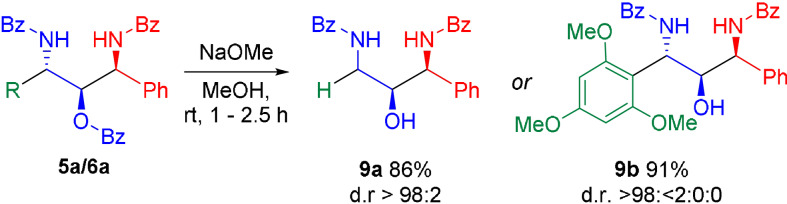
Deprotection of the benzoyl‐protected 1,3‐diamino‐2‐alcohols **5 a** and **6 a**. Given yields refer to the isolated yield of the major diastereomer.

Based on the observed results and previous reports on similar transformations with carbon‐substituted enamides,[^15a^, ^15b^, ^15c^] we assume the following reaction pathway for the first transformation. In the presence of a Lewis acid, precursor **3 a** liberates a reactive *N*‐acylimine, a known electron‐deficient heterodiene (Scheme 9 a).[^15d^, ^15e^, ^15f^] An inverse electron‐demand hetero‐Diels–Alder reaction between **I** and the oxyenamide **1 a**, proceeding in an *endo*‐fashion,^[15c]^ furnishes the 1,2‐*syn*‐configured dihydrooxazine intermediate **II**. Ring‐opening via cleavage of the hemiaminal functionality leads to a new acylimine **III**. Addition of MeOH affords the *N*,*O*‐acetal **4 a**. We assume that under the reaction conditions, compounds **II**, **III** and **4 a** exist in an equilibrium. In the presence of SiCl_4_ as coordinating Lewis acid, a 6‐membered *N*‐acylimine intermediate of type **IV** can be formed.^[16]^ Addition of the nucleophile from the sterically less hindered side leads to the selective formation of the third stereocenter and the 2,3‐*anti*‐configured product.

**Scheme 9:**
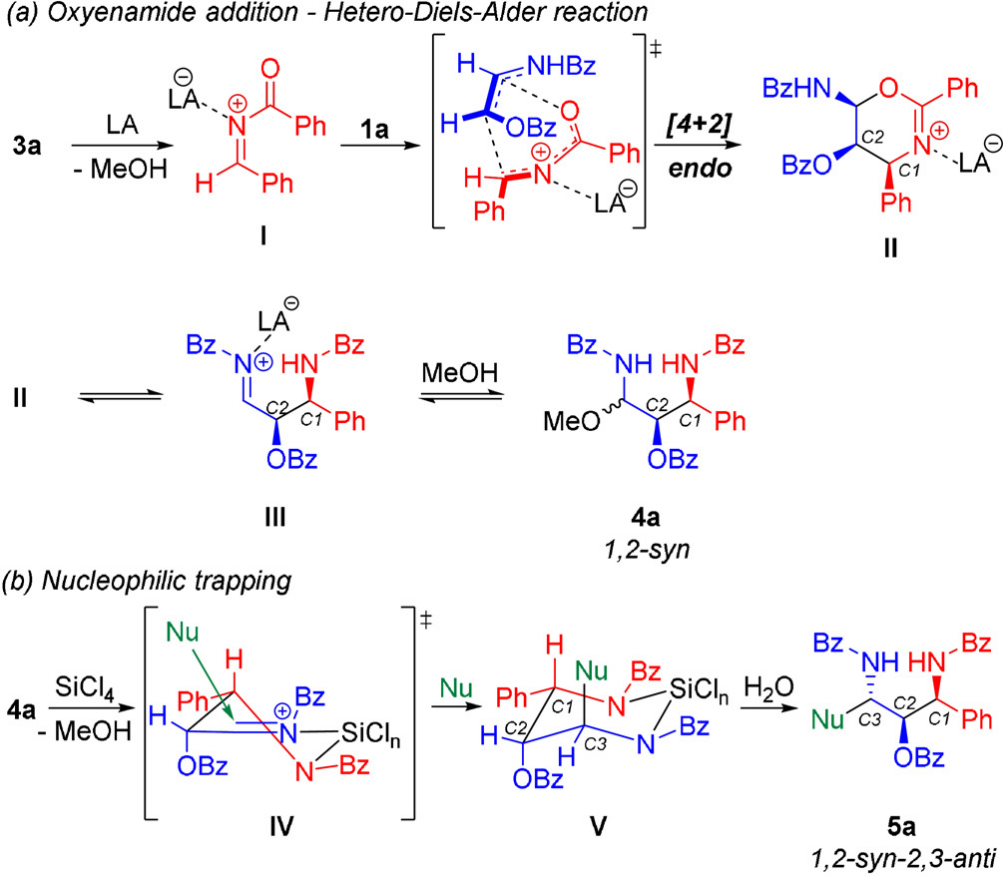
Tentative reaction mechanisms for the diastereoselective formation the three stereocenters.

In summary, we have reported a simple procedure for the synthesis of (*Z*)‐oxyenamides from common starting materials in only three steps. These oxyenamides represent a highly useful building block for the rapid assembly of the 1,3‐diamino‐2‐alcohol substructure, a common motif in natural products and drugs. A Lewis‐acid‐mediated one‐pot reaction between the oxyenamide and an *N*‐acylimine precursor followed by trapping with a terminal nucleophile enables a rapid and highly modular assembly of the 1,3‐diamino‐2‐alcohol scaffold containing up to three continuous stereocenters in good yields and with excellent diastereoselectivities. Facile removal of the acyl group directly affords unprotected 1,3‐diamino‐2‐alcohol. Further research towards the controlled synthesis of other stereoisomers, the development of an asymmetric version and applications in the synthesis of bioactive molecules as well as detailed mechanistic investigations are currently performed in our laboratories.

## Conflict of interest

The authors declare no conflict of interest.

## Supporting information

As a service to our authors and readers, this journal provides supporting information supplied by the authors. Such materials are peer reviewed and may be re‐organized for online delivery, but are not copy‐edited or typeset. Technical support issues arising from supporting information (other than missing files) should be addressed to the authors.

Supporting Information

Supporting Information
