# Supplementary material for: Oxyenamides as Versatile Building Blocks for a Highly Stereoselective One‐Pot Synthesis of the 1,3‐Diamino‐2‐ol‐Scaffold Containing Three Continuous Stereocenters
Source: Angew Chem Int Ed Engl. 2021 Sep 28;60(44):23667–71. doi: 10.1002/anie.202109752 (PMC8597051; doi:10.1002/anie.202109752)
Supplement: Supplementary file 1 — Supporting Information [file ANIE-60-23667-s001.zip › 21023ocu_checkcif.pdf]

checkCIF (full publication check) running

Checking for embedded fcf data in CIF ...
Found embedded fcf data in CIF. Extracting fcf data from uploaded CIF, please wait . . .

checkCIF/PLATON (full publication check)

Structure factors have been supplied for datablock(s) I

THIS REPORT IS FOR GUIDANCE ONLY. IF USED AS PART OF A REVIEW PROCEDURE FOR PUBLICATION, IT SHOULD NOT REPLACE THE EXPERTISE OF AN EXPERIENCED CRYSTALLOGRAPHIC REFEREE.

No syntax errors found.
Please wait while processing ....

CIF dictionary
Interpreting this report

Structure factor report

Datablock: 21023ocu

Bond precision: C-C = 0.0018 A Wavelength=1.54184

Cell:	a=16.4179(2)	b=12.6017(1)	c=17.2107(2)
	alpha=90	beta=112.839(2)	gamma=90

Temperature: 150 K

	Calculated	Reported
Volume	3281.61(8)	3281.61(8)
Space group	P 21/n	P 21/n
Hall group	-P 2yn	-P 2yn
Moiety formula	C39 H36 N2 O7	C39 H36 N2 O7
Sum formula	C39 H36 N2 O7	C39 H36 N2 O7
Mr	644.70	644.70
Dx,g cm-3	1.305	1.305
Z	4	4
Mu (mm-1)	0.732	0.732
F000	1360.0	1360.0
F000'	1364.26	
h,k,lmax	18,14,19	18,14,19
Nref	5257	5249
Tmin,Tmax	0.748,0.791	0.739,1.000
Tmin'	0.725	

Correction method= # Reported T Limits: Tmin=0.739 Tmax=1.000 AbsCorr = MULTI-SCAN

Data completeness= 0.998 Theta(max)= 62.686

R(reflections)= 0.0318(4960) wR2(reflections)= 0.0841(5249)

S = 1.043 Npar= 443

The following ALERTS were generated. Each ALERT has the format

test-name_ALERT_alert-type_alert-level.
Click on the hyperlinks for more details of the test.

Alert level C

THETM01_ALERT_3_C The value of sine(theta_max)/wavelength is less than 0.590
Calculated sin(theta_max)/wavelength = 0.5763
PLAT023_ALERT_3_C Resolution (too) Low [sin(theta)/Lambda < 0.6].. 0.58 Ang-1
PLAT906_ALERT_3_C Large K Value in the Analysis of Variance ...... 2.073 Check
PLAT911_ALERT_3_C Missing FCF Refl Between Thmin & STh/L= 0.576 8 Report

Alert level G

PLAT142_ALERT_4_G s.u. on b - Axis Small or Missing .... 0.00010 Ang.
PLAT793_ALERT_4_G Model has Chirality at C2 (Centro SPGR) R Verify
And 2 other PLAT793 Alerts
More ...
PLAT883_ALERT_1_G No Info/Value for _atom_sites_solution_primary . Please Do !
PLAT909_ALERT_3_G Percentage of I>2sig(I) Data at Theta(Max) Still 100% Note
PLAT913_ALERT_3_G Missing # of Very Strong Reflections in FCF .... 2 Note
PLAT978_ALERT_2_G Number C-C Bonds with Positive Residual Density. 10 Info
PLAT992_ALERT_5_G Repd & Actual _reflns_number_gt Values Differ by 2 Check

0 **ALERT level A** = Most likely a serious problem - resolve or explain
0 **ALERT level B** = A potentially serious problem, consider carefully
4 **ALERT level C** = Check. Ensure it is not caused by an omission or oversight
9 **ALERT level G** = General information/check it is not something unexpected

1 ALERT type 1 CIF construction/syntax error, inconsistent or missing data
1 ALERT type 2 Indicator that the structure model may be wrong or deficient
6 ALERT type 3 Indicator that the structure quality may be low
4 ALERT type 4 Improvement, methodology, query or suggestion
1 ALERT type 5 Informative message, check

checkCIF publication errors

Alert level A

PUBL004_ALERT_1_A The contact author's name and address are missing,
_publ_contact_author_name and _publ_contact_author_address.
PUBL005_ALERT_1_A _publ_contact_author_email, _publ_contact_author_fax and
_publ_contact_author_phone are all missing.
At least one of these should be present.
PUBL006_ALERT_1_A _publ_requested_journal is missing
e.g. 'Acta Crystallographica Section C'
PUBL008_ALERT_1_A _publ_section_title is missing. Title of paper.
PUBL009_ALERT_1_A _publ_author_name is missing. List of author(s) name(s).
PUBL010_ALERT_1_A _publ_author_address is missing. Author(s) address(es).

```
PUBL012_ALERT_1_A  _publ_section_abstract is missing.
            Abstract of paper in English.
```

Alert level G

```
PUBL017_ALERT_1_G The _publ_section_references section is missing or
            empty.
```

```
   7 ALERT level A = Data missing that is essential or data in wrong format
   1 ALERT level G = General alerts. Data that may be required is missing
```

Publication of your CIF

You should attempt to resolve as many as possible of the alerts in all categories. Often the minor alerts point to easily fixed oversights, errors and omissions in your CIF or refinement strategy, so attention to these fine details can be worthwhile. In order to resolve some of the more serious problems it may be necessary to carry out additional measurements or structure refinements. However, the nature of your study may justify the reported deviations from journal submission requirements and the more serious of these should be commented upon in the discussion or experimental section of a paper or in the "special_details" fields of the CIF. *checkCIF* was carefully designed to identify outliers and unusual parameters, but every test has its limitations and alerts that are not important in a particular case may appear. Conversely, the absence of alerts does not guarantee there are no aspects of the results needing attention. It is up to the individual to critically assess their own results and, if necessary, seek expert advice.

If level A alerts remain, which you believe to be justified deviations, and you intend to submit this CIF for publication in a journal, you should additionally insert an explanation in your CIF using the Validation Reply Form (VRF) below. This will allow your explanation to be considered as part of the review process.

Validation response form

Please find below a validation response form (VRF) that can be filled in and pasted into your CIF.

```
# start Validation Reply Form
_vrf_PUBL004_GLOBAL
;
PROBLEM: The contact author's name and address are missing,
RESPONSE: ...
;
_vrf_PUBL005_GLOBAL
;
PROBLEM: _publ_contact_author_email, _publ_contact_author_fax and
RESPONSE: ...
;
_vrf_PUBL006_GLOBAL
;
PROBLEM: _publ_requested_journal is missing
RESPONSE: ...
;
_vrf_PUBL008_GLOBAL
;
PROBLEM: _publ_section_title is missing. Title of paper.
RESPONSE: ...
;
_vrf_PUBL009_GLOBAL
;
```

```
PROBLEM: _publ_author_name is missing. List of author(s) name(s).
RESPONSE: ...
;
_vrf_PUBL010_GLOBAL
;
PROBLEM: _publ_author_address is missing. Author(s) address(es).
RESPONSE: ...
;
_vrf_PUBL012_GLOBAL
;
PROBLEM: _publ_section_abstract is missing.
RESPONSE: ...
;
# end Validation Reply Form
```

If you wish to submit your CIF for publication in Acta Crystallographica Section C or E, you should upload your CIF via the web. If you wish to submit your CIF for publication in IUCrData you should upload your CIF via the web. If your CIF is to form part of a submission to another IUCr journal, you will be asked, either during electronic submission or by the Co-editor handling your paper, to upload your CIF via our web site.

PLATON version of 05/12/2020; check.def file version of 05/12/2020

Datablock I - ellipsoid plot

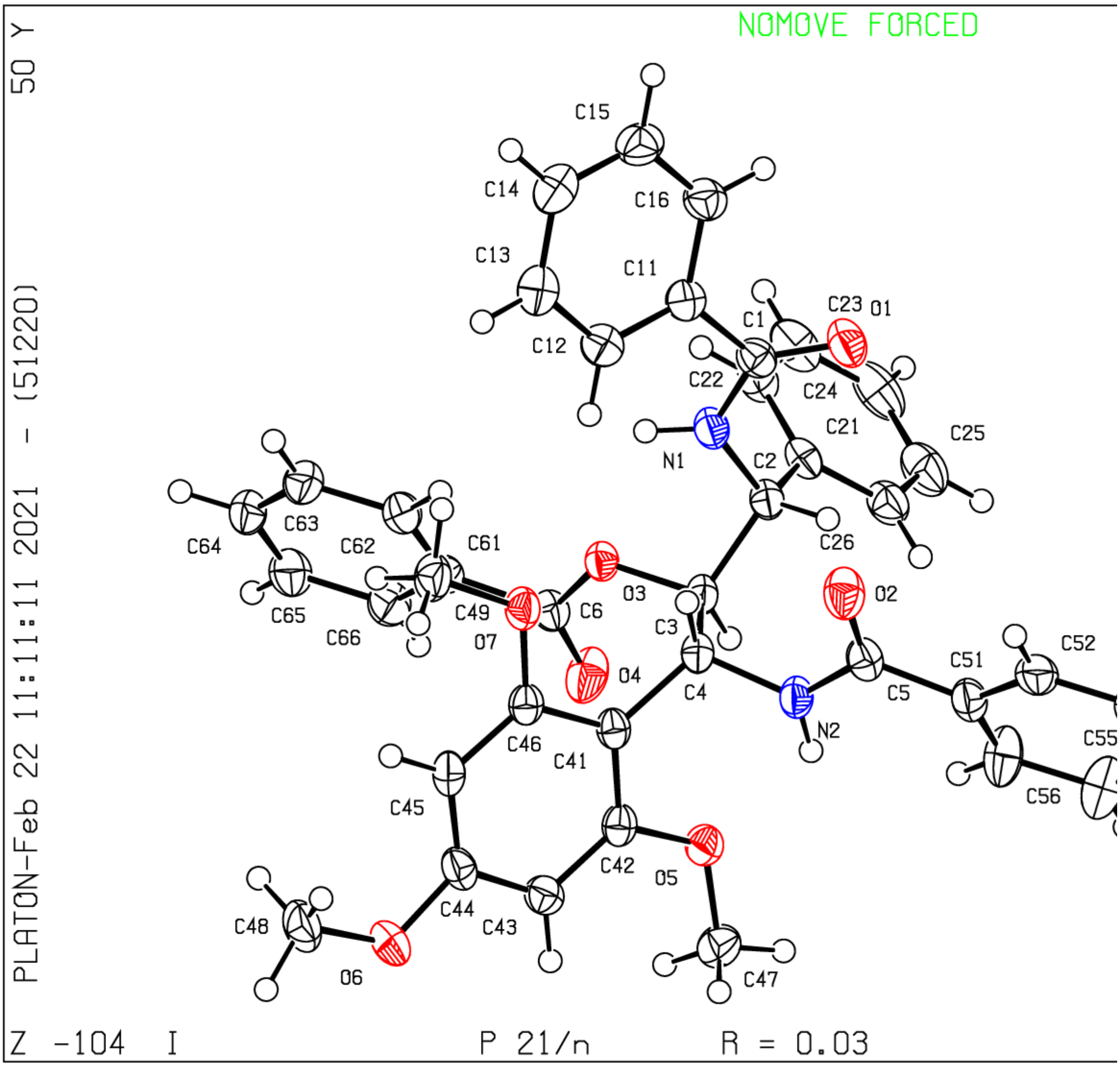

[Download CIF editor (publCIF) from the IUCr]
[Download CIF editor (enCIFer) from the CCDC]
[Test a new CIF entry]
