# Supplementary material for: Oxyenamides as Versatile Building Blocks for a Highly Stereoselective One‐Pot Synthesis of the 1,3‐Diamino‐2‐ol‐Scaffold Containing Three Continuous Stereocenters
Source: Angew Chem Int Ed Engl. 2021 Sep 28;60(44):23667–71. doi: 10.1002/anie.202109752 (PMC8597051; doi:10.1002/anie.202109752)
Supplement: Supplementary file 1 — Supporting Information [file ANIE-60-23667-s001.zip › 21031ocu_checkcif .pdf]

checkCIF (full publication check) running

Checking for embedded fcf data in CIF ...
Found embedded fcf data in CIF. Extracting fcf data from uploaded CIF, please wait . .

checkCIF/PLATON (full publication check)

Structure factors have been supplied for datablock(s) I

THIS REPORT IS FOR GUIDANCE ONLY. IF USED AS PART OF A REVIEW PROCEDURE FOR PUBLICATION, IT SHOULD NOT REPLACE THE EXPERTISE OF AN EXPERIENCED CRYSTALLOGRAPHIC REFEREE.

No syntax errors found.
Please wait while processing ....

CIF dictionary
Interpreting this report

Structure factor report

Datablock: 21031ocu

Bond precision:	C-C = 0.0036 A		Wavelength=1.54184
Cell:	a=12.3348(7)	b=12.8057(8)	c=13.5088(6)
	alpha=61.803(6)	beta=65.982(5)	gamma=63.355(6)
Temperature:	150 K		

	Calculated	Reported
Volume	1628.48(19)	1628.5(2)
Space group	P -1	P -1
Hall group	-P 1	-P 1
Moiety formula	C38 H30 Br N3 O4	C38 H30 Br N3 O4
Sum formula	C38 H30 Br N3 O4	C38 H30 Br N3 O4
Mr	672.55	672.56
Dx,g cm-3	1.372	1.372
Z	2	2
Mu (mm-1)	2.084	2.084
F000	692.0	692.0
F000'	692.50	
h,k,lmax	14,14,15	14,14,15
Nref	5228	5172
Tmin,Tmax	0.740,0.882	0.615,0.885
Tmin'	0.489	

Correction method= # Reported T Limits: Tmin=0.615 Tmax=0.885 AbsCorr = ANALYTICAL

Data completeness= 0.989 Theta(max)= 62.717

R(reflections)= 0.0355(4649) wR2(reflections)= 0.0959(5172)

S = 1.034 Npar= 415

The following ALERTS were generated. Each ALERT has the format

test-name_ALERT_alert-type_alert-level.
Click on the hyperlinks for more details of the test.

Alert level C

THETM01_ALERT_3_C The value of sine(theta_max)/wavelength is less than 0.590
Calculated sin(theta_max)/wavelength = 0.5764
PLAT023_ALERT_3_C Resolution (too) Low [sin(theta)/Lambda < 0.6].. 0.58 Ang-1
PLAT220_ALERT_2_C NonSolvent Resd 1 C Ueq(max)/Ueq(min) Range 4.2 Ratio
PLAT222_ALERT_3_C NonSolvent Resd 1 H Uiso(max)/Uiso(min) Range 4.2 Ratio
PLAT241_ALERT_2_C High 'MainMol' Ueq as Compared to Neighbors of C15 Check
PLAT414_ALERT_2_C Short Intra D-H..H-X H2A ..H56 1.96 Ang.
x,y,z = 1_555 Check
PLAT420_ALERT_2_C D-H Without Acceptor N2 --H2A . Please Check
PLAT906_ALERT_3_C Large K Value in the Analysis of Variance ...... 3.472 Check
PLAT911_ALERT_3_C Missing FCF Refl Between Thmin & STh/L= 0.576 56 Report

---

Alert level G

PLAT007_ALERT_5_G Number of Unrefined Donor-H Atoms .... 3 Report
PLAT793_ALERT_4_G Model has Chirality at C2 (Centro SPGR) S Verify
And 2 other PLAT793 Alerts
Less ...

PLAT793_ALERT_4_G Model has Chirality at C3 (Centro SPGR) R Verify

PLAT793_ALERT_4_G Model has Chirality at C4 (Centro SPGR) S Verify

PLAT802_ALERT_4_G CIF Input Record(s) with more than 80 Characters 1 Info
PLAT883_ALERT_1_G No Info/Value for _atom_sites_solution_primary . Please Do !
PLAT909_ALERT_3_G Percentage of I>2sig(I) Data at Theta(Max) Still 100% Note
PLAT933_ALERT_2_G Number of OMIT Records in Embedded .res File ... 5 Note
PLAT941_ALERT_3_G Average HKL Measurement Multiplicity .... 2.4 Low
PLAT965_ALERT_2_G The SHELXL WEIGHT Optimisation has not Converged Please Check
PLAT978_ALERT_2_G Number C-C Bonds with Positive Residual Density. 9 Info

---

0 **ALERT level A** = Most likely a serious problem - resolve or explain
0 **ALERT level B** = A potentially serious problem, consider carefully
9 **ALERT level C** = Check. Ensure it is not caused by an omission or oversight
11 **ALERT level G** = General information/check it is not something unexpected

```
   1 ALERT type 1 CIF construction/syntax error, inconsistent or missing
data
   7 ALERT type 2 Indicator that the structure model may be wrong or
deficient
   7 ALERT type 3 Indicator that the structure quality may be low
   4 ALERT type 4 Improvement, methodology, query or suggestion
   1 ALERT type 5 Informative message, check
```

checkCIF publication errors

Alert level A

```
PUBL004_ALERT_1_A  The contact author's name and address are missing,
            _publ_contact_author_name and _publ_contact_author_address.
PUBL005_ALERT_1_A  _publ_contact_author_email, _publ_contact_author_fax and
            _publ_contact_author_phone are all missing.
            At least one of these should be present.
PUBL006_ALERT_1_A  _publ_requested_journal is missing
            e.g. 'Acta Crystallographica Section C'
PUBL008_ALERT_1_A  _publ_section_title is missing. Title of paper.
PUBL009_ALERT_1_A  _publ_author_name is missing. List of author(s) name(s).
PUBL010_ALERT_1_A  _publ_author_address is missing. Author(s) address(es).
PUBL012_ALERT_1_A  _publ_section_abstract is missing.
            Abstract of paper in English.
```

Alert level G

```
PUBL017_ALERT_1_G The _publ_section_references section is missing or
            empty.
```

```
   7 ALERT level A = Data missing that is essential or data in wrong format
   1 ALERT level G = General alerts. Data that may be required is missing
```

Publication of your CIF

You should attempt to resolve as many as possible of the alerts in all categories. Often the minor alerts point to easily fixed oversights, errors and omissions in your CIF or refinement strategy, so attention to these fine details can be worthwhile. In order to resolve some of the more serious problems it may be necessary to carry out additional measurements or structure refinements. However, the nature of your study may justify the reported deviations from journal submission requirements and the more serious of these should be commented upon in the discussion or experimental section of a paper or in the "special_details" fields of the CIF. *checkCIF* was carefully designed to identify outliers and unusual parameters, but every test has its limitations and alerts that are not important in a particular case may appear. Conversely, the absence of alerts does not guarantee there are no aspects of the results needing attention. It is up to the individual to critically assess their own results and, if necessary, seek expert advice.

If level A alerts remain, which you believe to be justified deviations, and you intend to submit this CIF for publication in a journal, you should additionally insert an explanation in your CIF using the Validation Reply Form (VRF) below. This will allow your explanation to be considered as part of the review process.

Validation response form

Please find below a validation response form (VRF) that can be filled in and pasted into your CIF.

```
# start Validation Reply Form
_vrf_PUBL004_GLOBAL
;
PROBLEM: The contact author's name and address are missing,
RESPONSE: ...
;
_vrf_PUBL005_GLOBAL
;
PROBLEM: _publ_contact_author_email, _publ_contact_author_fax and
RESPONSE: ...
;
_vrf_PUBL006_GLOBAL
;
PROBLEM: _publ_requested_journal is missing
RESPONSE: ...
;
_vrf_PUBL008_GLOBAL
;
PROBLEM: _publ_section_title is missing. Title of paper.
RESPONSE: ...
;
_vrf_PUBL009_GLOBAL
;
PROBLEM: _publ_author_name is missing. List of author(s) name(s).
RESPONSE: ...
;
_vrf_PUBL010_GLOBAL
;
PROBLEM: _publ_author_address is missing. Author(s) address(es).
RESPONSE: ...
;
_vrf_PUBL012_GLOBAL
;
PROBLEM: _publ_section_abstract is missing.
RESPONSE: ...
;
# end Validation Reply Form
```

If you wish to submit your CIF for publication in Acta Crystallographica Section C or E, you should upload your CIF via the web. If you wish to submit your CIF for publication in IUCrData you should upload your CIF via the web. If your CIF is to form part of a submission to another IUCr journal, you will be asked, either during electronic submission or by the Co-editor handling your paper, to upload your CIF via our web site.

PLATON version of 05/12/2020; check.def file version of 05/12/2020

Datablock I - ellipsoid plot

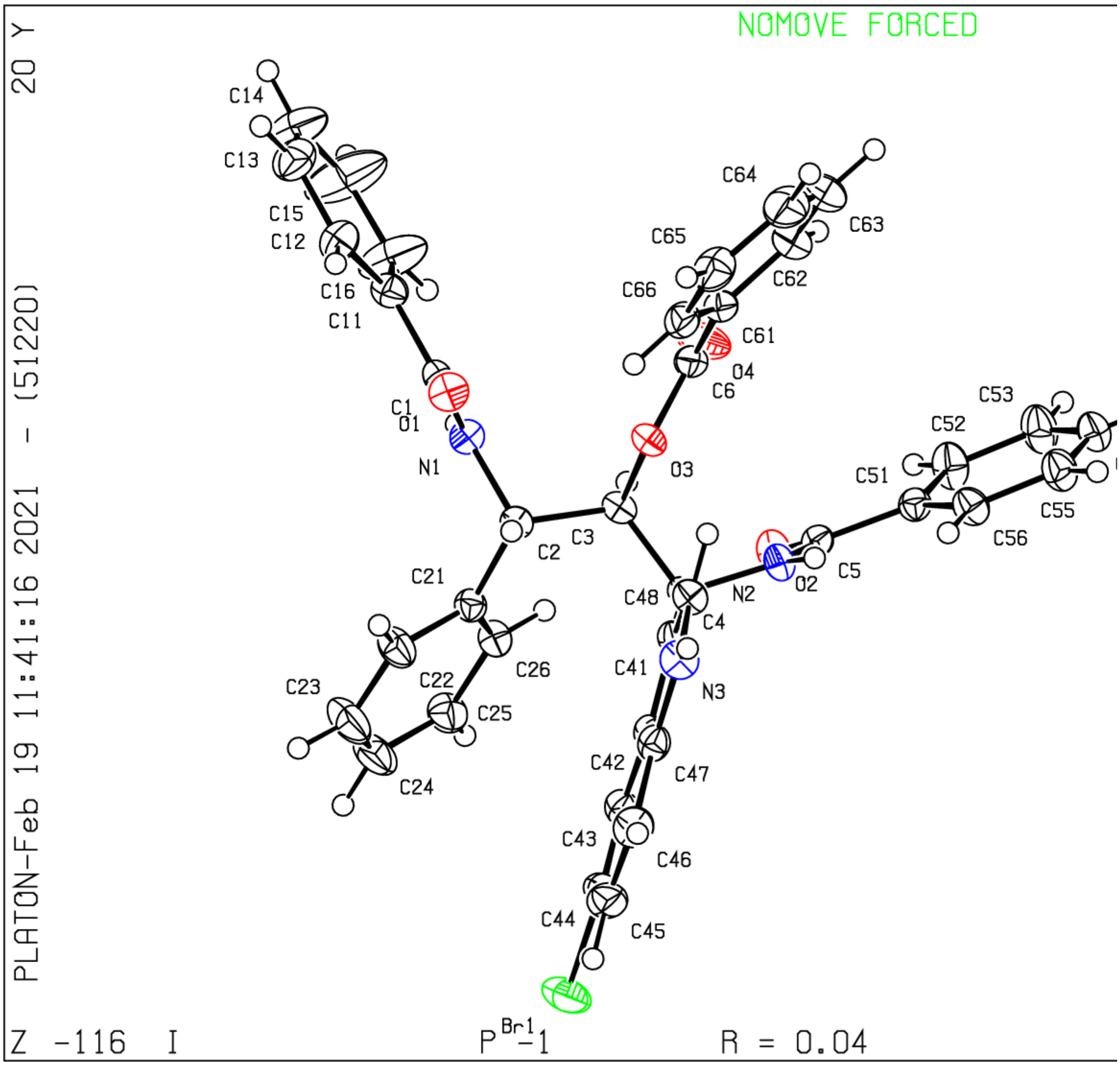

Download CIF editor (publCIF) from the IUCr
Download CIF editor (enCIFer) from the CCDC
Test a new CIF entry
