# Supplementary material for: Oxyenamides as Versatile Building Blocks for a Highly Stereoselective One‐Pot Synthesis of the 1,3‐Diamino‐2‐ol‐Scaffold Containing Three Continuous Stereocenters
Source: Angew Chem Int Ed Engl. 2021 Sep 28;60(44):23667–71. doi: 10.1002/anie.202109752 (PMC8597051; doi:10.1002/anie.202109752)
Supplement: Supplementary file 1 — Supporting Information [file ANIE-60-23667-s001.zip › ma105checkcif.pdf]

checkCIF (basic structural check) running

Checking for embedded fcf data in CIF ...
Found embedded fcf data in CIF. Extracting fcf data from uploaded CIF, please wait ....
....

checkCIF/PLATON (basic structural check)

Structure factors have been supplied for datablock(s) ma105

THIS REPORT IS FOR GUIDANCE ONLY. IF USED AS PART OF A REVIEW PROCEDURE FOR PUBLICATION, IT SHOULD NOT REPLACE THE EXPERTISE OF AN EXPERIENCED CRYSTALLOGRAPHIC REFEREE.

No syntax errors found. CIF dictionary
Please wait while processing .... Interpreting this report

Structure factor report

Datablock: ma105

```
Bond precision:       C-C = 0.0041 A              Wavelength=0.71073
Cell:        a=20.4044(7)      b=19.7803(10)     c=24.9582(9)
             alpha=90          beta=90           gamma=90
Temperature: 173 K
```

	Calculated	Reported
Volume	10073.3(7)	10073.3(7)
Space group	P b c a	P b c a
Hall group	-P 2ac 2ab	-P 2ac 2ab
Moiety formula	C28 H30 N2 O4	C28 H30 N2 O4
Sum formula	C28 H30 N2 O4	C28 H30 N2 O4
Mr	458.54	458.54
Dx,g cm-3	1.209	1.209
Z	16	16
Mu (mm-1)	0.081	0.081
F000	3904.0	3904.0
F000'	3905.76	
h,k,lmax	24,23,29	24,23,29
Nref	8895	8890
Tmin,Tmax	0.991,0.997	0.787,1.000
Tmin'	0.986	

```
Correction method= # Reported T Limits: Tmin=0.787 Tmax=1.000
AbsCorr = MULTI-SCAN
Data completeness= 0.999          Theta(max)= 25.027
R(reflections)= 0.0568( 5822)        wR2(reflections)= 0.1092( 8890)
S = 0.994                Npar= 629
```

The following ALERTS were generated. Each ALERT has the format
test-name_ALERT_alert-type_alert-level.
Click on the hyperlinks for more details of the test.

Alert level C

PLAT340_ALERT_3_C Low Bond Precision on C-C Bonds .... 0.00406 Ang.
PLAT480_ALERT_4_C Long H...A H-Bond Reported H3 ..O4A . 2.62 Ang.
PLAT906_ALERT_3_C Large K Value in the Analysis of Variance ...... 26.905 Check
And 2 other PLAT906 Alerts
More ...
PLAT910_ALERT_3_C Missing # of FCF Reflection(s) Below Theta(Min). 5 Note

Alert level G

PLAT720_ALERT_4_G Number of Unusual/Non-Standard Labels .... 8 Note
PLAT793_ALERT_4_G Model has Chirality at C2 (Centro SPGR) R Verify
And 3 other PLAT793 Alerts
More ...
PLAT883_ALERT_1_G No Info/Value for _atom_sites_solution_primary . Please Do !
PLAT909_ALERT_3_G Percentage of I>2sig(I) Data at Theta(Max) Still 40% Note
PLAT913_ALERT_3_G Missing # of Very Strong Reflections in FCF .... 1 Note
PLAT978_ALERT_2_G Number C-C Bonds with Positive Residual Density. 1 Info

0 **ALERT level A** = Most likely a serious problem - resolve or explain
0 **ALERT level B** = A potentially serious problem, consider carefully
6 **ALERT level C** = Check. Ensure it is not caused by an omission or oversight
9 **ALERT level G** = General information/check it is not something unexpected

1 ALERT type 1 CIF construction/syntax error, inconsistent or missing data
1 ALERT type 2 Indicator that the structure model may be wrong or deficient
7 ALERT type 3 Indicator that the structure quality may be low
6 ALERT type 4 Improvement, methodology, query or suggestion
0 ALERT type 5 Informative message, check

It is advisable to attempt to resolve as many as possible of the alerts in all categories. Often the minor alerts point to easily fixed oversights, errors and omissions in your CIF or refinement strategy, so attention to these fine details can be worthwhi In order to resolve some of the more serious problems it may be necessary to carry out additional measurements or structu refinements. However, the purpose of your study may justify the reported deviations and the more serious of these should normally be commented upon in the discussion or experimental section of a paper or in the "special_details" fields of the C checkCIF was carefully designed to identify outliers and unusual parameters, but every test has its limitations and alerts th are not important in a particular case may appear. Conversely, the absence of alerts does not guarantee there are no aspe of the results needing attention. It is up to the individual to critically assess their own results and, if necessary, seek expert advice.

Publication of your CIF in IUCr journals

A basic structural check has been run on your CIF. These basic checks will be run on all CIFs submitted for publication in IUCr journals (*Acta Crystallographica*, *Journal of Applied Crystallography*, *Journal of Synchrotron Radiation*); however, if yo intend to submit to *Acta Crystallographica Section C* or *E* or *IUCrData*, you should make sure that full publication checks a run on the final version of your CIF prior to submission.

Publication of your CIF in other journals

Please refer to the *Notes for Authors* of the relevant journal for any special instructions relating to CIF submission.

PLATON version of 13/07/2021; check.def file version of 13/07/2021

Datablock ma105 - ellipsoid plot

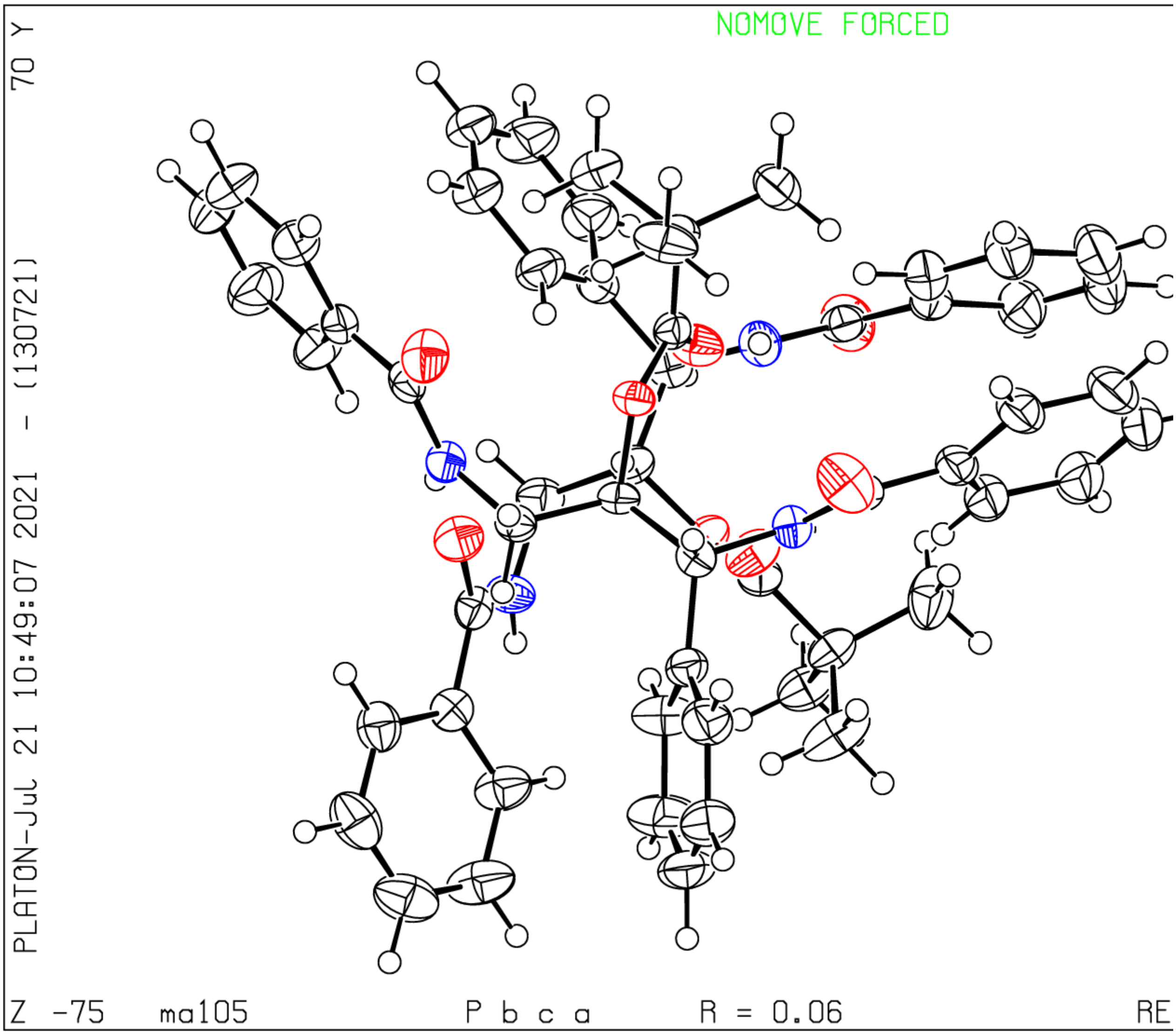

Download CIF editor (publCIF) from the IUCr
Download CIF editor (enCIFer) from the CCDC
Test a new CIF entry
