# Supplementary material for: Oxyenamides as Versatile Building Blocks for a Highly Stereoselective One‐Pot Synthesis of the 1,3‐Diamino‐2‐ol‐Scaffold Containing Three Continuous Stereocenters
Source: Angew Chem Int Ed Engl. 2021 Sep 28;60(44):23667–71. doi: 10.1002/anie.202109752 (PMC8597051; doi:10.1002/anie.202109752)
Supplement: Supplementary file 2 — Supporting Information [file ANIE-60-23667-s002.pdf]

Angewandte Chemie
Eine Zeitschrift der Gesellschaft Deutscher Chemiker

Supporting Information

Oxyenamides as Versatile Building Blocks for a Highly Stereoselective One-Pot Synthesis of the 1,3-Diamino-2-ol-Scaffold Containing Three Continuous Stereocenters

*Sara-Cathrin Krieg[+], Jennifer Grimmer[+], Philipp Kramer, Michael Bolte, Harald Kelm, and Georg Manolikakes**

anie_202109752_sm_miscellaneous_information.pdf

1 General Information 2
2 Preparation and analytical data 4
2.1 Preparation of the *N*-protected aminoalcohols 4
2.2 Preparation of the aldehydes 6
2.3 Preparation of the (*Z*)-Oxyenamides/(*Z*)-Oxyencarbamates 7
2.4 Preparation of *N,O*-Acetals 10
2.5 Reductions 13
2.6 1,3-Diamino-2-ols from different (*Z*)-Oxyenamides/(Z)-Oxyencarbamates 19
2.7 1,3-Diamino-2-ols from different Nucleophiles 22
2.8 1,3-Diamino-2-ols from different *N*-Acylimine precoursors 31
2.9 Deprotection 38
3 NMR Data 41
4 X-ray Data 85
5 References 95

1 General Information

Experimental methods. Unless otherwise mentioned, all reactions were carried out under nitrogen atmosphere in flame dried glassware applying standard Schlenk techniques. All yields refer to isolated yields of compounds estimated to be > 95% pure as determined by ^{1}H-NMR.

Chromatography. Flash column chromatography was performed using a puriflash XS 420+ Flash purifier machine from Interchim with prepacked flash columns (Puriflash_SilicaHP_30µm_F0012, Puriflash_SilicaHP_30µm_F0025 or Puriflash_SilicaHP_30µm_F0040) and the respectively specified solvent mixture. All yields refer to isolated yields of compounds estimated to be >95% pure as determined by ^{1}H NMR. Thin layer chromatography was performed on aluminum sheets coated with SiO_2 (TLC silica gel 60 F254). The spots were visualized by ultraviolet light, iodine, cerium ammonium molybdate (CAM) or vanillin.

Solvents. Solvents for reactions and column chromatography were obtained from different commercial suppliers in >97% purity and used as received. Solvents for column chromatography were technical standard.

Materials. All starting materials obtained from commercial sources were used without further purification. Anhydrous $BF_3{\cdot}OEt_2$ was obtained from different providers and used directly. Prolonged storage times were avoided.

N-Acylimine precoursors were synthesized from the corresponding amides and aldehydes using the previously reported protocol of Halli *et al*.[1]

Analytical Data and Instrumentation

NMR spectroscopy Proton nuclear magnetic resonance spectra (^{1}H NMR) and carbon spectra (^{13}C NMR) were recorded at 400 or 600 MHz (^{1}H) and 101 MHz (^{13}C), respectively. Chemical shifts are reported as δ - values relative to the residual $CDCl_3$ (δ = 7.26 ppm for ^{1}H and δ = 77.16 ppm for ^{13}C), DMSO-d^{6} (δ = 2.50 ppm for 1H and δ = 39.51 ppm for ^{13}C). Coupling constants (*J*) are given in Hz and multiplicities of the signals are abbreviated as follows: s = singlet; d = doublet; t = triplet; q = quartet; sp = septet; m = multiplet; dd = doublet of doublets and dt = doublet of triplets dqd = doublet of quartets of doublets.

Melting points. Melting points are reported uncorrected.

Mass spectrometry. Mass spectra (MS) were measured using electrospray ionization (ESI) techniques. High resolution mass spectra (HRMS) were measured using electron ionization mass spectroscopy (EI-MS-TOF).

Infrared spectroscopy. Infrared spectra (IR) of neat substances were recorded on a FT-IR (Fourier transform infrared spectroscopy) spectrometer equipped with a diamond universal ATR sampling technique (attenuated total reflectance). The absorption bands are reported in wave numbers (cm^{-1}).

Elemental analysis. Elemental analyses (CHN) were performed with a vario Micro cube.

Diastereomeric ratio. The diastereomeric ratios (*dr*) were determined via ^{1}H-NMR analysis both for the crude product after aqueous workup and after isolation via column chromatography. A diastereomeric ratio of *dr* > 98:2 indicates that no other isomer was observed by ^{1}H NMR. Yields refer to isolated yields of the analytically pure 1,2-*syn*-2,3-*anti*-1,3-diamin-2-ol (*dr* > 98:2). If other diastereomers could be isolated, a combined yield is given in addition. Minor diastereomers were not fully characterized. In some cases no minor isomers could be isolated after column chromatography, although their formation was observed via NMR analysis of the crude reaction mixture. Presumably, small amounts of the side products were lost during column chromatography.

Crystal structure determinations.

Data for **1c**, **5b** and **5c** (CCDC 2087484, 2087485, 2087486) were collected on a STOE IPDS II two-circle diffractometer with a Genix Microfocus tube with mirror optics using MoKα radiation (λ = 0.71073 Å). The data were scaled using the frame scaling procedure in the X-AREA program system (Stoe & Cie, 2002)[2]. The structures were solved by direct methods using the program SHELXS-2014/6 and refined against F^2 with full-matrix least-squares techniques using the program SHELXL-2014/6.[3]

Data for **6a**, **7a**, **7c**, **7g**, **7h** and **7i** (CCDC 2097900, 2097895, 2097896, 2097898, 2097987, 2097899) were collected at 150.0(1) K on a Gemini S Ultra by Rigaku Oxford Diffraction, equipped with a molybdenum (λ = 0.71073 Å) and a copper (λ = 1.54184 Å) radiation source and a low-temperature control device. Due to the positioning of the two sources in the device, data collection is somewhat limited to smaller angles, which may result in alerts in some checkcif files. Absorption correction was done with CrysAlis Pro 1.171.38.41 and 1.171.40.67a, respectively. All structures were solved using the software programs SHELXS-2018/3, and the positions of all non-hydrogen atoms were refined with SHELXL-2018/3.[3]

2 Preparation and analytical data

2.1 Preparation of the *N*-protected aminoalcohols

Typical procedure 1 (TP1)

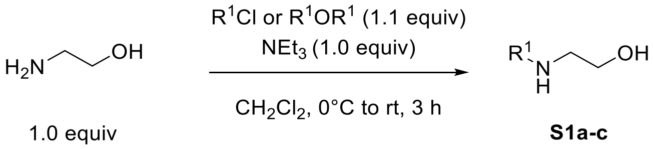

To a solution of ethanolamine (1.0 equiv) and Et_3N (1.0 equiv) in dichlormethane (3 mL/mmol) was added dropwise the corresponding acyl chloride or acid anhydride (1.1 equiv) at 0 °C. After stirring for 3 h at room temperature, the reaction was quenched with saturated aqueous NH_4Cl (100 mL) and the mixture was extracted with CH_2Cl_2 (3x 100 mL). The combined organic layers were dried over Na_2SO_4, filtered and the solvents were evaporated under reduced pressure. Purification of the crude residue by flash column chromatography (*n*-hexane/EtOAc) afforded the analytically pure product.[4]

2.1.1 *N*-(2-hydroxyethyl)benzamide **S1a**

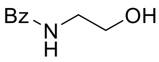

S1a

Prepared according to **TP1** from ethanolamine (3.1 mL, 50 mmol, 1.0 equiv), Et_3N (9.0 mL, 55 mmol, 1.1 equiv) and benzoyl chloride (5.8 mL, 50.0 mmol, 1.0 equiv) in a total of 200 mL dichlormethane. Purification of the crude residue by flash column chromatography (*n*-hexane/EtOAc = 1:1 → 1:9) afforded the analytically pure product as a colorless solid (5.26 g, 64%). Analytical data are in accordance with the literature.[4]

$\mathbf{R_f}$ (*n*-hexane:EtOAc = 1:1) 0.07

m.p. 62-64 °C

^{1}H NMR (400 MHz, *Chloroform-d*) δ = 7.82 – 7.67 (m, 2H), 7.48 – 7.41 (m, 1H), 7.39 – 7.31 (m, 2H), 7.14 (d, J=6.2, 1H), 3.82 – 3.68 (m, 3H), 3.55 (q, J=5.2, 2H).

^{13}C-NMR (101 MHz, *Chloroform-d*) δ = 168.9, 134.1, 131.8, 128.6, 127.1, 62.0, 42.9.

IR (ATR, ν in cm^{-1}): 3336 (m), 3272 (w), 2946 (w), 2775 (w), 1632 (s), 1576 (m), 1542 (s), 1488 (m), 1417 (m), 1311 (m), 1229 (m),1185 (w), 1056 (s), 933 (w) 899 (w), 804 (w), 695 (s).

MS (ESI) m/z calcd for $C_9H_{12}NO_2Na$ 188.1 $[M+Na]^+$, found 188.1 $[M+Na]^+$.

2.1.2 *tert*-butyl (2-hydroxyethyl)carbamate **S1b**

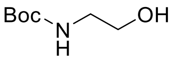

S1b

Prepared according to **TP1** from ethanolamine (1.2 mL, 20 mmol, 1.0 equiv), Et_3N (2.8 mL, 20 mmol, 1.0 equiv) and di-*tert*-butyldicarbonat (4.2 mL, 22.0 mmol, 1.1 equiv) in a total of 60 mL dichlormethane. Purification of the crude residue by flash column chromatography (*n*-hexane/EtOAc = 1:1) afforded the analytically pure product as a colorless liquid (2.20 g, 68%). Analytical data are in accordance with the literature.[5]

R_f (*n*-hexane:EtOAc = 1:1) 0.27.

^{1}H NMR (400 MHz, *Chloroform-d*) δ = 3.68 (dd, *J*=5.6, 4.6, 2H), 3.27 (t, *J*=5.1, 2H), 1.44 (s, 9H).

^{13}C-NMR (101 MHz, *Chloroform-d*) δ = 156.9, 79.9, 62.5, 43.4, 28.5.

IR (ATR, ν in cm^{-1}): 3352 (w), 2977 (w), 2877 (w), 1687 (s), 1519 (s), 1454 (w), 1392 (m), 1366 (s), 1274 (s), 1249 (s), 1161 (s), 1064 (m), 865 (w).

MS (ESI) m/z calcd for $C_7H_{15}NO_3Na$ 184.1 $[M+Na]^+$, found 184.1 $[M+Na]^+$.

2.1.3 benzyl (2-hydroxyethyl)carbamate **S1c**

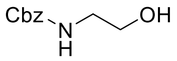

S1c

Prepared according to **TP1** from ethanolamine (1.2 mL, 20 mmol, 1.0 equiv), Et_3N (2.8 mL, 20 mmol, 1.0 equiv) and benzyl chloroformate (3.8 mL, 22.0 mmol, 1.1 equiv) in a total of 60 mL dichlormethane. Purification of the crude residue by flash column chromatography (*n*-hexane/EtOAc = 1:1) afforded the analytically pure product as a colorless solid (2.60 g, 67%). Analytical data are in accordance with the literature.[5]

R_f (*n*-hexane:EtOAc = 1:1) 0.19.

m.p. 63-65 °C

^{1}H NMR (400 MHz, *Chloroform-d*) δ = 7.44 – 7.28 (m, 5H), 5.30 (bs, 1H), 5.10 (s, 2H), 3.69 (t, *J*=5.1, 2H), 3.34 (d, *J*=5.3, 2H), 2.51 (s, 1H).

^{13}C-NMR (101 MHz, *Chloroform-d*) δ = 157.3, 136.4, 128.6, 128.3, 128.2, 67.0, 62.2, 43.6.

IR (ATR, ν in cm^{-1}): 3319 (m), 3061 (w), 2940 (w), 1690 (s), 1539 (s), 1449 (m), 1323 (w), 1262 (s), 1213 (m), 1148 (m), 1113 (w), 1058 (w), 1031 (m), 989 (m), 774 (m).

MS (ESI) m/z calcd $C_{10}H_{13}NO_3Na$ 218.1 $[M+Na]^+$, found 218.1 $[M+Na]^+$.

2.2 Preparation of the aldehydes

Typical procedure 2 (TP2)

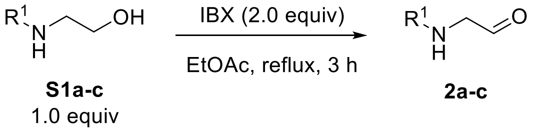

To a solution of *N*-protected aminoalcohol **S1** (1.0 equiv) in ethylacetate (5 mL/mmol) was added 2-iodoxybenzoic acid (2.0 equiv) in one portion. The resulting suspension was refluxed for 3 h. After TLC analysis showed complete consumption of the starting material the reaction mixture was filtered through a short plug of Celite and the residue washed with ethylacetate. The solvents were removed under reduced pressure and the crude product was dried for 2-3 h under oil pump vacuum (10^{-2} mbar) to afford the desired aldehyde. All aldehydes of type **2** are not stable and rapidly decompose upon prolonged storage. Therefore, all aldehydes were used directly without further purification.[7]

2.2.1 *N*-(2-oxoethyl)benzamide 2a

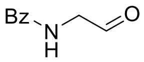

2a

Prepared according to **TP2** from *N*-(2-hydroxyethyl)benzamide **S1a** (1.01 g, 6.10 mmol, 1.0 equiv), 2-iodoxybenzoic acid (3.42 g, 12.2 mmol, 2.0 equiv) in a total of 30 mL ethylacetate. Removal of the solvents afforded the crude aldehyde as a colorless liquid (990 mg, 99%). Analytical data are in accordance with the literature.[4]

R_f (n-hexane:EtOAc = 3:7) 0.27.

^{1}H NMR (400 MHz, *Chloroform-d*) δ = 9.75 (s, 1H), 7.90 – 7.73 (m, 2H), 7.56 – 7.49 (m, 1H), 7.48 – 7.41 (m, 2H), 7.03 (s, 1H), 4.39 (d, J=4.8, 2H).

2.2.2 *tert*-butyl (2-oxoethyl)carbamate 2b

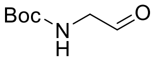

2b

Prepared according to **TP2** from *tert*-butyl (2-hydroxyethyl)carbamate **S1b** (708 mg, 4.39 mmol, 1.0 equiv), 2-iodoxybenzoic acid (2.46 g, 8.78 mmol, 2.0 equiv) in a total of 23 mL ethylacetate. Removal of the solvents afforded the crude aldehyde as a colorless liquid (640 mg, 92%). Analytical data are in accordance with the literature.[8]

R_f (*n*-hexane:EtOAc = 1:1) 0.43.

^{1}H NMR (400 MHz, *Chloroform-d*) δ = 9.65 (s, 1H), 5.18 (s, 1H), 4.08 (d, J=5.1, 2H), 1.45 (s, 9H).

2.2.3 benzyl (2-oxoethyl)carbamate **2c**

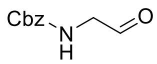

2c

Prepared according to **TP2** from benzyl (2-hydroxyethyl)carbamate **S1c** (1.01 g, 5.15 mmol, 1.0 equiv), 2-iodoxybenzoic acid (2.88 g, 10.3 mmol, 2.0 equiv) in a total of 25 mL ethylacetate. Removal of the solvents afforded the crude aldehyde as a colorless liquid (994 mg, 95%). Analytical data are in accordance with the literature.[9]

R_f (*n*-hexane:EtOAc = 1:1) 0.42.

^{1}H NMR (400 MHz, *Chloroform-d*) δ = 9.67 (s, 1H), 7.42 – 7.27 (m, 5H), 5.42 (s, 1H), 5.13 (s, 2H), 4.17 (d, *J*=5.0, 2H).

2.3 Preparation of the (*Z*)-Oxyenamides/(*Z*)-Oxyencarbamates

Typical procedure 3 (TP3)

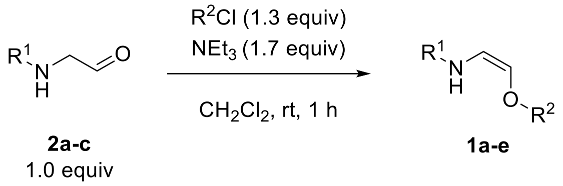

To a stirred solution of triethylamine (1.7 equiv) and the corresponding acyl chloride (1.3 equiv) in dichlormethane (2 mL/mmol) was added dropwise a solution of the aldehyde **2a-c** (1.0 equiv) in dichlormethane (2 mL/mmol) over 5 min. The reaction mixture was stirred for 1 h at room temperature. After TLC analysis showed complete consumption of the aldehyde, saturated $NaHCO_3$ solution was added (20 mL). The organic layers were separated and the aqueous phase was extracted with dichlormethane (2x 30 mL). The combined organic layers were dried over Na_2SO_4, filtered and the solvents were evaporated under reduced pressure. Purification by flash chromatography afforded the desired (*Z*)-oxyenamide/(Z)-oxyencarbamate **1a-e** as analytically pure product. All oxyenamides of type **1** tend to decompose upon contact to any type of acid. Therefore, column chromotography was performed with 0.2 vol% NEt_3 as additive. $CDCl_3$ for NMR spectroscopy was passed through short plug of basic alumina before use.

2.3.1 (*Z*)-2-benzamidovinyl benzoate **1a**

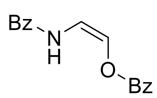

1a

Prepared according to **TP3** from *N*-(2-oxoethyl)benzamide **2a** (774 mg, 4.74 mmol, 1.0 equiv), triethylamine (1.1 mL, 8.06 mmol, 1.7 equiv) and benzoyl chloride (0.72 mL, 6.16 mmol, 1.3 equiv) in

a total of 20 mL dichlormethane. Purification by flash chromatography (*n*-hexane:EtOAc + 0.2 vol% NEt_3 = 19:1 → 9:1) afforded (*Z*)-2-benzamidovinyl benzoate **1a** (927 mg, 73%) as colorless solid.

R$_f$ (*n*-hexane:EtOAc = 4:1) 0.38.

m.p. 172-175 °C.

^{1}H NMR (*400 MHz, Chloroform-d*) δ = 8.17 – 8.06 (m, 2H), 7.97 (d, J=10.2, 1H), 7.91 – 7.81 (m, 2H), 7.68 – 7.62 (m, 1H), 7.61 – 7.56 (m, 1H), 7.55 – 7.48 (m, 4H), 7.11 (d, J=5.1, 1H), 6.86 (dd, J=10.7, 5.1, 1H).

^{13}C NMR (101 MHz, *Chloroform-d*) δ = 164.0, 162.7, 134.0, 133.4, 132.4, 130.0, 129.0, 128.9, 128.8, 127.3, 121.9, 109.5.

IR (ATR, ν in cm^{-1}): 3275 (w), 3110 (w), 3032 (w), 1723 (m), 1686 (w), 1643 (m), 1601 (w), 1580 (w), 1519 (s), 1484 (s), 1451 (w), 1249 (s), 1146 (m), 1113 (s), 1093 (s), 1068 (s), 798 (w), 687 (s).

MS (ESI) m/z calcd for $C_{16}H_{13}NO_3Na$ 290.1 [M+Na]$^+$, found 290.2 [M+Na]$^+$.

HRMS (EI) m/z calcd for $C_{16}H_{13}NO_3$ 267.0895 [M]$^+$, found 267.0901 [M]$^+$.

2.3.2 (*Z*)-2-benzamidovinyl pivalate 1b

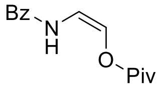

1b

Prepared according to **TP3** from *N*-(2-oxoethyl)benzamide **2a** (721 mg, 4.42 mmol, 1.0 equiv), triethylamine (1.04 mL, 7.51 mmol, 1.7 equiv) and pivaloyl chloride (0.71 mL, 5.75 mmol, 1.3 equiv) in a total of 20 mL dichlormethane. Purification by flash chromatography (*n*-hexane:EtOAc + 0.2 vol% NEt_3 = 19:1 → 9:1) afforded (*Z*)-2-benzamidovinyl pivalate **1b** (609 mg, 56%) as colorless solid.

R$_f$ (*n*-hexane:EtOAc = 4:1) 0.45.

m.p. 92-94 °C.

^{1}H NMR (400 MHz, *Chloroform-d*) δ = 7.94 – 7.70 (m, 3H), 7.61 – 7.54 (m, 1H), 7.53 – 7.42 (m, 2H), 6.90 (d, *J*=5.0, 1H), 6.75 (dd, *J*=10.6, 5.0, 1H), 1.32 (s, 9H).

^{13}C NMR (101 MHz, *Chloroform-d*) δ = 174.1, 163.6, 133.4, 132.4, 129.1, 127.1, 122.0, 108.9, 39.2, 27.3.

IR (ATR, ν in cm^{-1}): 3345 (w), 2984 (w), 1741 (s), 1687 (w), 1645 (s), 1503 (s), 1478 (s), 1395 (w), 1364 (w), 1272 (s), 1124 (s), 1026 (w), 929 (w), 885 (m), 751 (m), 717 (m), 694 (m).

MS (ESI) m/z calcd for $C_{14}H_{17}NO_3Na$ 270.1 [M+Na]$^+$, found 270.1 [M+Na]$^+$

HRMS (EI) m/z calcd for $C_{14}H_{17}NO_3$ 247.1208 [M]$^+$, found 247.1206 [M]$^+$.

2.3.3 (*Z*)-2-benzamidovinyl acetate 1c

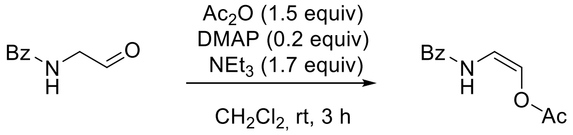

2a 1.0 equiv — **1c** 56%

To a stirred solution of triethylamine (1.17 mL, 8.43 mmol, 1.7 equiv), 4-dimethylaminopyridine (122 mg, 0.992 mmol, 0.2 equiv) and acetic anhydride (0.58 mL, 7.44 mmol, 1.3 equiv) in dichlormethane (10 mL) was added dropwise a solution of the aldehyde **1a** (810 mg, 4.96 mmol, 1.0 equiv) in dichlormethane (10 mL). The reaction mixture was stirred for 3 h at room temperature. After TLC analysis showed complete consumption of the aldehyde, saturated $NaHCO_3$ solution was added (20 mL). The organic layers were separated and the aqueous phase was extracted with dichlormethane (2x 30 mL). The combined organic layers were dried over Na_2SO_4, filtered and the solvents were evaporated under reduced pressure. Purification by flash chromatography (*n*-hexane:EtOAc + 0.2 vol% NEt_3 = 9:1 → 4:1) afforded (*Z*)-2-benzamidovinyl acetate **1c** (572 mg, 56%) as colorless solid.

R_f (*n*-hexane:EtOAc = 4:1) 0.22.

m.p. 80-82 °C.

^{1}H NMR (400 MHz, *Chloroform-d*) δ = 7.97 – 7.78 (m, 3H), 7.60 – 7.51 (m, 1H), 7.52 – 7.43 (m, 2H), 6.90 (d, *J*=5.2, 1H), 6.71 (dd, *J*=10.7, 5.1, 1H), 2.22 (s, 3H).

^{13}C NMR (101 MHz, *Chloroform-d*) δ = 166.7, 164.0, 133.4, 132.4, 128.9, 127.3, 121.5, 108.6, 20.8.

IR (ATR, ν in cm^{-1}): 3255 (w), 3206 (w), 3221 (w), 1750 (m), 1702 (w), 1640 (s), 1603 (w), 1579 (w), 1514 (s), 1484 (s), 1362 (m) 1294 (m), 1207 (s), 1147 (s), 1116 (s), 1040 (m), 1028 (m), 878 (m), 756 (m), 682 (m).

MS (ESI) m/z calcd for $C_{11}H_{11}NO_3Na$ 228.1 $[M+Na]^+$, found 228.1 $[M+Na]^+$.

HRMS (EI) m/z calcd for $C_{11}H_{11}NO_3$ 205.0739 $[M]^+$, found 205.0740 $[M]^+$.

2.3.4 (*Z*)-2-((*tert*-butoxycarbonyl)amino)vinyl benzoate 1d

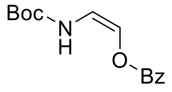

1d

Prepared according to **TP3** from *tert*-butyl (2-oxoethyl)carbamate **2b** (637 mg, 4.0 mmol, 1.0 equiv), triethylamine (0.95 mL, 6.80 mmol, 1.7 equiv) and benzoyl chloride (0.60 mL, 5.20 mmol, 1.3 equiv) in a total of 16 mL dichlormethane. Purification by flash chromatography (*n*-hexane:EtOAc + 0.2 vol% NEt_3 = 19:1 → 9:1) afforded (*Z*)-2-((*tert*-butoxycarbonyl)amino)vinyl benzoate **1d** (567 mg, 54%) as colorless solid.

R_f (*n*-hexane:EtOAc = 9:1) 0.43.

m.p. 143-144 °C.

^{1}H NMR (400 MHz, *Chloroform-d*) δ = 8.09 (dd, *J*=8.1, 1.5, 2H), 7.71 – 7.57 (m, 1H), 7.49 (dd, *J*=8.4, 7.1, 2H), 6.88 (d, *J*=5.0, 1H), 6.47 (d, *J*=11.2, 1H), 6.33 (dd, *J*=11.5, 4.8, 1H), 1.51 (s, 9H).

^{13}C NMR (101 MHz, *Chloroform-d*) δ = 162.9, 152.2, 133.8, 130.0, 129.0, 128.8, 119.2, 110.8, 81.3, 28.4.

IR (ATR, ν in cm^{-1}): 3295 (w), 3124 (w), 2982 (w), 1702 (s), 1700 (m), 1686 (s), 1602 (w), 1502 (s), 1494 (s), 1454 (m), 1369 (m), 1259 (s), 1240 (s), 1148 (s), 1094 (s), 1073 (s), 1027 (m), 886 (m), 704 (s).

MS (ESI) m/z calcd for $C_{14}H_{17}NO_4Na$ 286.1 [M+Na]$^+$, found 286.2 [M+Na]$^+$.

HRMS (EI) m/z calcd for $C_{14}H_{17}NO_3$ 263.1158 [M+H]$^+$, found 263.1159 [M+H]$^+$.

2.3.5 (*Z*)-2-(((benzyloxy)carbonyl)amino)vinyl benzoate **1e**

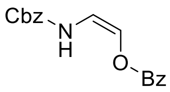

1e

Prepared according to **TP3** from benzyl (2-oxoethyl)carbamate **2c** (945 mg, 4.89 mmol, 1.0 equiv), triethylamine (1.16 mL, 8.31 mmol, 1.7 equiv) and benzoyl chloride (0.74 mL, 6.36 mmol, 1.3 equiv) in a total of 20 mL dichlormethane. Purification by flash chromatography (*n*-hexane:EtOAc + 0.2 vol% NEt_3 = 19:1 → 9:1) afforded (*Z*)-2-(((benzyloxy)carbonyl)amino)vinyl benzoate **1e** (870 mg, 60%) as colorless solid.

R$_f$ (*n*-hexane:EtOAc = 9:1) 0.24.

m.p. 69-71 °C.

^{1}H NMR (400 MHz, *Chloroform-d*) δ = 8.20 – 7.98 (m, 2H), 7.68 – 7.54 (m, 1H), 7.48 (t, *J*=7.8, 2H), 7.44 – 7.27 (m, 5H), 7.01 – 6.80 (m, 1H), 6.71 (d, *J*=11.0, 1H), 6.38 (dd, *J*=11.0, 5.1, 1H), 5.20 (s, 2H).

^{13}C NMR (101 MHz, *Chloroform-d*) δ = 162.8, 153.1, 135.7, 133.9, 130.0, 128.8, 128.8, 128.7, 128.7, 119.9, 110.5, 67.9.

IR (ATR, ν in cm^{-1}): 3270 (w), 3128 (w), 3030 (w), 1729 (s), 1689 (s), 1601 (w), 1518 (s), 1450 (m), 1360 (w), 1228 (s), 1159 (m), 1100 (s), 1074 (s), 1026 (m), 906 (w), 741 (m), 696 (s).

MS (ESI) m/z calcd for $C_{17}H_{15}NO_4Na$ 320.1 [M+Na]$^+$, found 320.2 [M+Na]$^+$.

HRMS (EI) m/z calcd for $C_{17}H_{25}NO_3$ 297.1001 [M]$^+$, found 297.0997 [M]$^+$.

2.4 Preparation of *N,O*-Acetals

Typical procedure 4 (TP4)

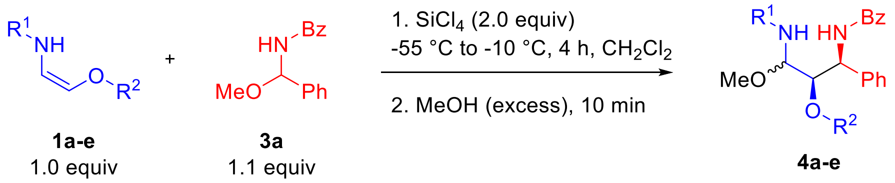

A flame dried and nitrogen flushed Schlenk tube, equipped with a septum and a magnetic stirrer, was charged with (*Z)*-oxyenamide/-encarbamate **1a-e** (1.0 equiv), *N*-acylimine precursor **3a** (1.1 equiv) and dichloromethane (10 mL/mmol). The solution was cooled to -55 °C and $SiCl_4$ (2.0 equiv) was added dropwise. The resulting mixture was warmed to -10 °C within 2.5 h to 4 h. After TLC analysis of an aliquot showed complete consumption of the *(Z)*-oxyenamide/-encarbamate, methanol (3 mL/mmol) was added at -10 °C. The resulting solution was warmed to room temperature and stirred for 10 min.

Saturated aqueous NH_4Cl (20 mL) was added and the organic layer was separated. The aqueous phase was extracted with dichloromethane (2x 25 mL). The combined organic layers were dried over Na_2SO_4 and the solvents were evaporated under reduced pressure. Purification by flash chromatography afforded the *N,O*-acetal as analytically pure product. All *N,O*-acteals **4a-e** were only briefly characterized via ^{1}H-NMR and used directly as mixture of diastereomers in the subsequent transformations.

2.4.1 *N,O*-Acetal **4a**

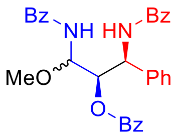

4a

Prepared according to **TP4** from (*Z*)-2-benzamidovinyl benzoate **1a** (1.34 g, 5.0 mmol, 1.0 equiv), *N*-acylimine precursor **3a** (1.33 g, 5.5 mmol, 1.1 equiv), $SiCl_4$ (1.15 mL, 10.0 mmol, 2.0 equiv) and methanol (15 mL) in a total of 50 mL dichloromethane. Purification by flash chromatography via puriflash XS 420+ machine, HP_15µm_F0040 flash column (*n*-hexane:EtOAc = 95:5 → 50:50) afforded the desired *N,O*-acetal **4a** as a colorless foam (2.28 g, 90%, isolated *dr* = 70:30).

^{1}H NMR (400 MHz, *Chloroform-d*) δ = 8.09 – 8.01 (m, 1.4H), 8.00 – 7.93 (m, 0.6H), 7.80 – 7.78 (m, 0.6H), 7.73 – 7.63 (m, 3.4H), 7.61 – 7.29 (m, 14H), 7.10 (t, J=9.6, 1.3H), 6.81 (d, J=9.8, 0.3H), 5.88 – 5.77 (m, 1H), 5.71 – 5.65 (m, 1H), 5.55 (dd, J=9.9, 6.3, 0.3H), 5.48 (dd, J=9.5, 3.1, 0.7H), 3.45 (s, 0.9H), 3.39 (s, 2.1H). *(peaks not assigned to single stereoisomers)*

2.4.2 *N,O*-Acetal **4b**

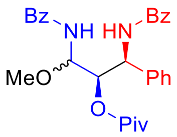

4b

Prepared according to **TP4** from (*Z*)-2-benzamidovinyl pivalate **1b** (742 mg, 3.0 mmol, 1.0 equiv), *N*-acylimine precursor **3a** (796 mg, 3.3 mmol, 1.1 equiv), $SiCl_4$ (0.69 mL, 6.0 mmol, 2.0 equiv) and methanol (9 mL) in a total of 30 mL dichloromethane. Purification by flash chromatography via puriflash XS 420+ machine, HP_15µm_F0040 flash column (*n*-hexane:EtOAc = 93:7 → 30:70) afforded the desired *N,O*-acetal **4b** as a colorless foam (1.24 g, 85%, isolated *dr* = 83:17).

^{1}H NMR (400 MHz, *Chloroform-d*) δ = 7.87 – 7.81 (m, 0.4H), 7.80 – 7.71 (m, 2H), 7.70 – 7.62 (m, 1.6H), 7.58 – 7.29 (m, 11H), 7.15 (d, J=9.3, 0.2H), 7.08 (d, J=8.7, 1.8H), 6.85 (d, J=9.6, 0.8H), 6.66 (d, J=8.8, 0.2H), 5.77 (dd, J=9.2, 3.4, 0.2H), 5.70 (dd, J=8.7, 6.4, 0.8H), 5.45 – 5.33 (m, 2H), 3.43 (s, 0.5H), 3.36 (s, 1.5H), 1.17 (s, 7.5H), 1.05 (s, 1.5H). *(peaks not assigned to single stereoisomers)*

2.4.3 *N,O*-Acetal **4c**

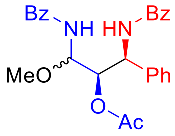

4c

Prepared according to **TP4** from (*Z*)-2-benzamidovinyl acetate **1c** (616 mg, 3.0 mmol, 1.0 equiv), *N*-acylimine precursor **3a** (796 mg, 3.3 mmol, 1.1 equiv), $SiCl_4$ (0.69 mL, 6.0 mmol, 2.0 equiv) and methanol (9 mL) in a total of 30 mL dichloromethane. Purification by flash chromatography via puriflash XS 420+ machine, HP_15µm_F0040 flash column (*n*-hexane:EtOAc = 95:5 → 50:50) afforded the desired *N,O*-acetal **4c** as a colorless foam (1.18 g, 88%, isolated *dr* = 67:33).

^{1}H NMR (400 MHz, *Chloroform-d*) δ = 7.92 – 7.67 (m, 4H), 7.63 – 7.29 (m, 11H), 7.03 (dd, *J*=12.9, 9.0, 1H), 6.92 (d, *J*=9.5, 0.5H), 6.76 (d, *J*=9.0, 0.5iH), 5.71 (ddd, *J*=19.3, 9.0, 5.3, 1H), 5.49 – 5.36 (m, 2H), 3.41 (s, 1.6H), 3.37 (s, 1.4H), 2.13 (s, 1.4H), 2.03 (s, 1.6H). *(peaks not assigned to single stereoisomers)*

2.4.4 *N,O*-Acetal **4d**

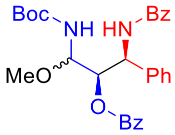

4d

Prepared according to **TP4** from (*Z*)-2-((tert-butoxycarbonyl)amino)vinyl benzoate **1d** (132 mg, 0.5 mmol, 1.0 equiv), *N*-acylimine precursor **3a** (133 mg, 0.55 mmol, 1.1 equiv), $SiCl_4$ (0.12 mL, 1.0 mmol, 2.0 equiv) and methanol (3.5 mL) in a total of 5 mL dichloromethane. Purification by flash chromatography via puriflash XS 420+ machine, HP_15µm_F0025 flash column (*n*-hexane:EtOAc = 95:5 → 60:40) afforded the desired *N,O*-acetal **4d** as a colorless foam (214 mg, 85%, isolated *dr* = 60:40).

^{1}H NMR (400 MHz, *Chloroform-d*) δ = 8.09 (d, *J*=7.5, 0.8H), 8.01 (d, *J*=7.5, 1.2H), 7.87 – 7.72 (m, 1H), 7.64 (d, *J*=7.6, 0.8H), 7.60 – 7.52 (m, 1H), 7.52 – 7.27 (m, 11H), 7.09 (d, *J*=8.5, 0.2H), 5.75 – 5.62 (m, 1H), 5.58 (t, *J*=5.8, 1H), 5.18 – 5.10 (m, 1H), 4.89 (dd, *J*=10.6, 5.9, 0.6H), 4.79 (dd, *J*=10.4, 2.7, 0.4H), 3.35 (s, 1.5H), 3.30 (s, 1.5H), 1.38 (s, 3H), 1.34 (s, 6H). *(peaks not assigned to single stereoisomers)*

2.4.5 *N,O*-Acetal **4e**

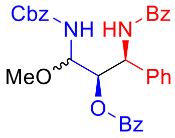

4e

Prepared according to **TP4** from (*Z*)-2-(((benzyloxy)carbonyl)amino)vinyl benzoate **1e** (297 mg, 1.0 mmol, 1.0 equiv), *N*-acylimine precursor **3a** (265 mg, 1.1 mmol, 1.1 equiv), $SiCl_4$ (0.23 mL, 2.0 mmol, 2.0 equiv) and methanol (7 mL) in a total of 10 mL dichloromethane. Purification by flash

chromatography via puriflash XS 420+ machine, HP_15µm_F0025 flash column (*n*-hexane:EtOAc = 95:5 → 60:40) afforded the desired *N,O*-acetal **4e** as a colorless foam (432 mg, 80%, isolated *dr* = 52:48).
^{1}H NMR (400 MHz, *Chloroform-d*) δ = 8.05 (d, *J*=7.6, 1H), 7.99 (d, *J*=7.7, 1H), 7.75 (d, *J*=7.6, 0.8H), 7.64 – 7.55 (m, 2.2H), 7.52 – 7.27 (m, 14H), 7.24 – 7.19 (m, 1.2H), 7.05 (d, *J*=8.5, 0.8H), 5.78 – 5.64 (m, 1H), 5.61 – 5.57 (m, 1H), 5.46 (d, *J*=10.3, 1H), 5.13 – 4.94 (m, 2.5H), 4.86 (dd, *J*=10.2, 2.8, 0.5H), 3.36 (s, 1.2H), 3.31 (s, 1.8H). *(peaks not assigned to single stereoisomers)*

2.5 Reductions

Typical procedure 5 (TP5)– Reduction of the *N,O*-acetals

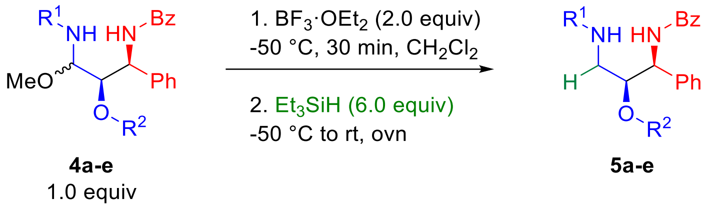

A flame dried and nitrogen flushed Schlenk tube, equipped with a septum and a magnetic stirrer, was charged with *N,O*-acetal **4a-e** (1.0 equiv) and dichloromethane (10 mL/mmol) and cooled to -50 °C. After addition of BF_3·OEt_2 (2.0 equiv) the reaction mixture was stirred for 30 min at -50 °C. Triethylsilane (6.0 equiv) was added at -50 °C. The solution was allowed to slowly warm to room temperature and stirred for additionally 24 h at room temperature. After TLC analysis of an aliquot showed complete consumption of the starting material, the reaction was quenched with saturated aqueous NH_4Cl (3 mL) and diluted with dichloromethane. The organic layer was separated and the aqueous phase was extracted with dichloromethane (2x 5 mL). The combined organic layers were dried over Na_2SO_4 and the solvents were evaporated under reduced pressure. Purification by flash chromatography afforded the desired 1,2-*syn*-1,3-diamin-2-ol **5a-e** as analytically pure product.

Typical procedure 6 (TP6)– One-Pot-Reduction

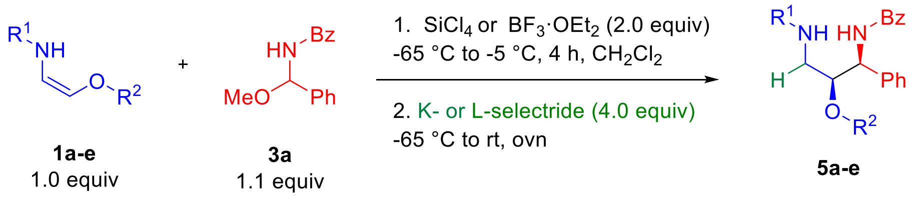

A flame dried and nitrogen flushed Schlenk tube, equipped with a septum and a magnetic stirrer, was charged with (*Z*)-oxyenamide/-encarbamate **1a-e** (1.0 equiv), *N*-acylimine precursor **3a** (1.1 equiv) and dichloromethane (10 mL/mmol). The solution was cooled to -65 °C and $SiCl_4$ or BF_3·OEt_2 (2.0 equiv) was added. The reaction was allowed to warm to -10 °C within 4 h. After TLC showed complete consumption of (*Z*)-oxyenamide/-encarbamate the reaction mixture was again cooled to -65 °C and K-selectride (4.0 equiv with $SiCl_4$ as Lewis acid) or L-selectride (4.0 equiv with BF_3·OEt_2 as Lewis acid) was added. The mixture was allowed to slowly warm to room temperature overnight and then quenched

with saturated aqueous NH_4Cl (3 mL). The organic layer was separated and the aqueous phase was extracted with dichloromethane (2x 5 mL). The combined organic layers were dried over Na_2SO_4 and the solvents were evaporated under reduced pressure. Purification by flash chromatography (*n*-hexane:EtOAc) afforded the desired 1,2-*syn*-1,3-diamin-2-ol **5a-e** as analytically pure product.

2.5.1 1,3-Diamino-2-ol 5a

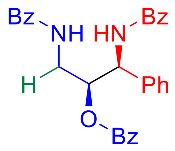

5a

a) From the reduction of the N,O-acetal

Prepared according to **TP5** from *N,O*-acetal **4a** (131 mg, 0.25 mmol, 1.0 equiv), $BF_3{\cdot}OEt_2$ (0.13 mL, 0.5 mmol, 2.0 equiv) and triethylsilane (0.25 mL, 1.5 mmol, 6.0 equiv) in a total of 2.5 mL dichloromethane. Purification by flash chromatography via puriflash XS 420+ machine, HP_15µm_F0012 flash column (*n*-hexane:EtOAc = 95:5 → 50:50) afforded the desired 1,3-diamino-2-ol **5a** as a colorless foam (96.7 mg, 79%, isolated *dr* >98: 2; *dr* of the crude mixture >98: 2, as determined by ^{1}H NMR analysis of the crude product after aqueous workup).

b) Via the One-Pot-Reduction

Prepared according to **TP6** from (*Z*)-2-benzamidovinyl benzoate **1a** (66.8 mg, 0.25 mmol, 1.0 equiv), *N*-acylimine precursor **3a** (66.4 mg, 0.275 mmol, 1.1 equiv), $SiCl_4$ (58 mL, 0.5 mmol, 2.0 equiv) and K-selectride (1.0 mL, 1 mmol, 4.0 equiv, 1 M solution in THF) in a total of 2.5 mL dichloromethane. Purification by flash chromatography via puriflash XS 420+ machine, HP_15µm_F0012 flash column (*n*-hexane:EtOAc = 95:5 → 50:50) afforded the desired 1,3-diamino-2-ol **5a** as colorless solid (87.3 mg, 73%, isolated *dr* > 98:2 *dr* of the crude mixture >98: 2 as determined by ^{1}H NMR analysis of the crude product after aqueous workup).

R_f (*n*-hexane:EtOAc = 6:4) 0.37.

m.p. 196-199 °C.

^{1}H NMR (400 MHz, *Chloroform-d*) δ = 8.07 – 7.93 (m, 2H), 7.80 (dd, *J*=7.3, 1.8, 4H), 7.59 – 7.53 (m, 1H), 7.52 – 7.46 (m, 4H), 7.45 – 7.27 (m, 9H), 7.24 (d, *J*=9.0, 1H), 7.11 (t, *J*=6.1, 1H), 5.78 – 7.53 (m, 1H), 5.68 (dd, *J*=8.8, 5.8, 1H), 3.96 (dt, *J*=13.7, 6.7, 1H), 3.60 (dt, *J*=14.2, 5.3, 1H).

^{13}C NMR (101 MHz, *Chloroform-d*) δ = 167.7, 166.8, 138.0, 134.1, 133.9, 133.6, 132.0, 131.8, 129.9, 129.4, 129.2, 128.8, 128.7, 128.7, 128.5, 127.3, 127.2, 127.2, 75.1, 54.9, 41.1.

IR (ATR, ν in cm^{-1}): 3237 (w), 3066 (w), 2933 (w), 1711 (m), 1636 (s), 1602 (w), 1536 (s), 1489 (m), 1451 (w), 1297 (m), 1269 (s), 1106 (m), 1071 (w), 1041 (w), 800 (w), 690 (s).

MS (ESI) m/z calcd for $C_{30}H_{26}N_2NaO_4$ 501.2 $[M+Na]^+$, found 501.4 $[M+Na]^+$.

HRMS (EI) m/z calcd for $C_{30}H_{27}N_2O_4$ 479.1965 [M+H]$^+$, found 479.1966 [M+H]$^+$.

2.5.2 1,3-Diamino-2-ol 5b

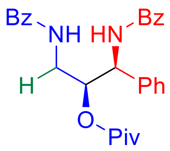

5b

a) From the reduction of the N,O-acetal

Prepared according to **TP5** from *N,O*-acetal **4b** (122 mg, 0.25 mmol, 1.0 equiv), $BF_3{\cdot}OEt_2$ (0.13 mL, 0.5 mmol, 2.0 equiv) and triethylsilane (0.25 mL, 1.5 mmol, 6.0 equiv) in a total of 2.5 mL dichloromethane. Purification by flash chromatography via puriflash XS 420+ machine, HP_15µm_F0012 flash column (*n*-hexane:EtOAc = 95:5 → 50:50) afforded the desired 1,3-diamino-2-ol **5b** as a colorless foam (28.3 mg, 27%, isolated *dr* >98: 2; *dr* of the crude mixture >98:2, as determined by ^{1}H NMR analysis of the crude product after aqueous workup).

b) Via the One-Pot-Reduction

Prepared according to **TP6** from (*Z*)-2-benzamidovinyl pivalate **1b** (61.8 mg, 0.25 mmol, 1.0 equiv), *N*-acylimine precursor **3a** (66.4 mg, 0.275 mmol, 1.1 equiv), $BF_3{\cdot}OEt_2$ (0.13 mL, 0.5 mmol, 2.0 equiv) and L-selectride (1.0 mL, 1 mmol, 4.0 equiv, 1 M solution in THF) in a total of 2.5 mL dichloromethane. Purification by flash chromatography via puriflash XS 420+ machine, HP_15µm_F0012 flash column (*n*-hexane:EtOAc = 95:5 → 50:50) afforded the desired 1,3-diamino-2-ol **5b** as colorless solid (73.4 mg, 64%, isolated *dr* > 98:2; *dr* of the crude mixture >98:2, as determined by ^{1}H NMR analysis of the crude product after aqueous workup).

R_f (*n*-hexane:EtOAc = 6:4) 0.35.

m.p. 102-106 °C.

^{1}H NMR (400 MHz, *Chloroform-d*) δ = 7.87 – 7.80 (m, 2H), 7.82 – 7.74 (m, 2H), 7.58 – 7.38 (m, 8H), 7.39 – 7.27 (m, 3H), 7.07 (d, *J*=9.0, 1H), 6.85 (t, *J*=6.1, 1H), 5.58 (dd, *J*=9.0, 5.8, 1H), 5.50 (dt, *J*=6.8, 5.5, 1H), 3.86 (dt, *J*=14.2, 7.0, 1H), 3.46 (dt, *J*=14.2, 5.2, 1H), 1.11 (s, 9H).

^{13}C NMR (101 MHz, *Chloroform-d*) δ = 178.8, 167.5, 167.2, 138.1, 134.0, 133.8, 132.0, 131.8, 129.1, 128.8, 128.7, 128.3, 127.2, 127.1, 127.1, 74.1, 54.9, 41.0, 39.1, 27.2.

IR (ATR, ν in cm^{-1}): 3305 (w), 2970 (w), 1710 (m), 1641 (s), 1603 (w), 1579 (w), 1527 (s), 1484 (s), 1397 (w), 1281 (s), 1151 (s), 1032 (m), 800 (w), 694 (s).

MS (ESI) m/z calcd for $C_{28}H_{30}N_2NaO_4$ 481.2 [M+Na]$^+$, found 481.4 [M+Na]$^+$.

HRMS (EI) m/z calcd for $C_{28}H_{31}N_2O_4$ 459.2278 [M+H]$^+$, found 459.2284 [M+H]$^+$.

2.5.3 1,3-Diamino-2-ol **5c**

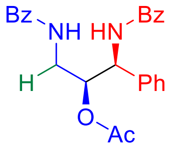

5c

a) From the reduction of the N,O-acetal

Prepared according to **TP5** from *N,O*-acetal **4c** (112 mg, 0.25 mmol, 1.0 equiv), $BF_3{\cdot}OEt_2$ (0.13 mL, 0.5 mmol, 2.0 equiv) and triethylsilane (0.25 mL, 1.5 mmol, 6.0 equiv) in a total of 2.5 mL dichloromethane. Purification by flash chromatography via puriflash XS 420+ machine, HP_15µm_F0012 flash column (*n*-hexane:EtOAc = 90:10 → 30:70) afforded the desired 1,3-diamino-2-ol **5c** as a colorless foam (79.4 mg, 74%, isolated *dr* >98: 2; *dr* of the crude mixture >98:2, as determined by ^{1}H NMR analysis of the crude product after aqueous workup).

b) Via the One-Pot-Reduction

Prepared according to **TP6** from (*Z*)-2-benzamidovinyl acetate **1c** (61.8 mg, 0.25 mmol, 1.0 equiv), *N*-acylimine precursor **3a** (66.4 mg, 0.275 mmol, 1.1 equiv), $SiCl_4$ (58 mL, 0.5 mmol, 2.0 equiv) and K-selectride (1.0 mL, 1 mmol, 4.0 equiv, 1 M solution in THF) in a total of 2.5 mL dichloromethane. Purification by flash chromatography via puriflash XS 420+ machine, HP_15µm_F0012 flash column (*n*-hexane:EtOAc = 90:10 → 30:70) afforded the desired 1,3-diamino-2-ol **5c** as colorless solid (70.2 mg, 67%, isolated *dr* > 98:2; *dr* of the crude mixture >98:2, as determined by ^{1}H NMR analysis of the crude product after aqueous workup).

R_f (*n*-hexane:EtOAc = 6:4) 0.14.

m.p. >200 °C.

^{1}H NMR (400 MHz, *Chloroform-d*) δ = 7.89 – 7.78 (m, 4H), 7.60 – 7.28 (m, 11H), 7.10 (t, J=6.0, 1H), 7.01 (d, J=9.0, 1H), 5.59 (dd, J=9.1, 4.8, 1H), 5.56 – 5.48 (m, 1H), 4.03 – 3.87 (m, 1H), 3.50 – 3.31 (m, 1H), 2.04 (s, 3H).

^{13}C NMR (101 MHz, *Chloroform-d*) δ = 170.8, 167.8, 167.5, 138.0, 134.0, 133.8, 132.2, 131.8, 129.2, 128.9, 128.7, 128.4, 127.2, 127.2, 127.0, 74.1, 54.2, 40.7, 21.1.

IR (ATR, v in cm^{-1}): 3268 (w), 3071 (w), 1730 (s), 1632 (s), 1604 (w), 1538 (s), 1490 (m), 1424 (w), 1369 (m), 1316 (m), 1232 (s), 1113 (w), 1043 (m), 943 (w), 803 (w), 693 (s).

MS (ESI) m/z calcd for $C_{25}H_{24}N_2NaO_4$ 439.2 $[M+Na]^+$, found 439.3 $[M+Na]^+$.

HRMS (EI) m/z calcd for $C_{25}H_{25}N_2O_4$ 417.1809 $[M+H]^+$, found 417.1812 $[M+H]^+$.

2.5.4 1,3-Diamino-2-ol **5d**

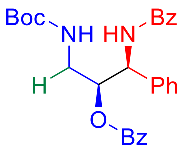

5d

a) From the reduction of the N,O-acetal

Prepared according to **TP5** from *N,O*-acetal **4d** (126 mg, 0.25 mmol, 1.0 equiv), $BF_3{\cdot}OEt_2$ (0.13 mL, 0.5 mmol, 2.0 equiv) and triethylsilane (0.25 mL, 1.5 mmol, 6.0 equiv) in a total of 2.5 mL dichloromethane. Purification by flash chromatography via puriflash XS 420+ machine, HP_15µm_F0012 flash column (*n*-hexane:EtOAc = 95:5 → 50:50) afforded the desired 1,3-diamino-2-ol 5**d** as a colorless foam (16.9 mg, 15%, isolated *dr* >98: 2; *dr* of the crude mixture >98:2, as determined by ^{1}H NMR analysis of the crude product after aqueous workup).

b) Via the One-Pot-Reduction

Prepared according to **TP6** from *(Z)*-2-((*tert*-butoxycarbonyl)amino)vinyl benzoate **1d** (65.8 mg, 0.25 mmol, 1.0 equiv), *N*-acylimine precursor **3a** (66.4 mg, 0.275 mmol, 1.1 equiv), $BF_3{\cdot}OEt_2$ (0.13 mL, 0.5 mmol, 2.0 equiv) and L-selectride (1.0 mL, 1 mmol, 4.0 equiv, 1 M solution in THF) in a total of 2.5 mL dichloromethane. Purification by flash chromatography via puriflash XS 420+ machine, HP_15µm_F0012 flash column (*n*-hexane:EtOAc = 95:5 → 50:50) afforded the desired 1,3-diamino-2-ol **5d** as colorless solid (59.8 mg, 50%, isolated *dr* > 98:2; *dr* of the crude mixture >98:2, as determined by ^{1}H NMR analysis of the crude product after aqueous workup).

R_f (*n*-hexane:EtOAc = 7:3) 0.38.

m.p. 169-171 °C.

^{1}H NMR (400 MHz, *Chloroform-d*) δ = 8.03 (d, *J*=7.4, 2H), 7.73 (d, *J*=7.6, 2H), 7.55 (d, *J*=7.4, 1H), 7.49 – 7.27 (m, 10H), 7.21 (d, *J*=8.4, 1H), 5.70 – 5.58 (m, 1H), 5.54 (t, *J*=7.8, 1H), 4.98 (s, 1H), 3.53 – 3.29 (m, 2H), 1.39 (s, 9H).

^{13}C NMR (101 MHz, *Chloroform-d*) δ = 167.1, 155.9, 138.3, 134.1, 133.6, 131.8, 130.0, 129.5, 129.2, 128.7, 128.6, 128.4, 127.3, 127.1, 79.9, 75.5, 55.2, 41.7, 28.4.

IR (ATR, ν in cm^{-1}): 3406 (w), 3273 (w), 2974 (w), 1717 (m), 1698 (s), 1631 (s), 1511 (m), 1452 (w), 1365 (m), 1268 (s), 1241 (s), 1164 (m), 1108 (s), 1067 (m), 1026 (m), 855 (w), 708 (s), 690 (s).

MS (ESI) m/z calcd for $C_{28}H_{30}N_2NaO_5$ 497.2 $[M+Na]^+$, found 497.4 $[M+Na]^+$.

HRMS (EI) m/z calcd for $C_{28}H_{31}N_2O_5$ 475.2227 $[M+H]^+$, found 475.2225 $[M+H]^+$.

2.5.5 1,3-Diamino-2-ol **5e**

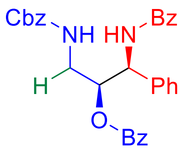

5e

a) From the reduction of the N,O-acetal

Prepared according to **TP5** from *N,O*-acetal **4e** (135 mg, 0.25 mmol, 1.0 equiv), $BF_3 \cdot OEt_2$ (0.13 mL, 0.5 mmol, 2.0 equiv) and triethylsilane (0.25 mL, 1.5 mmol, 6.0 equiv) in a total of 2.5 mL dichloromethane. Purification by flash chromatography via puriflash XS 420+ machine, HP_15µm_F0012 flash column (*n*-hexane:EtOAc = 95:5 → 50:50) afforded the desired 1,3-diamino-2-ol **5e** as a colorless foam (62. mg, 27%, isolated *dr* >98: 2; *dr* of the crude mixture >98:2, as determined by ^{1}H NMR analysis of the crude product after aqueous workup).

b) Via the One-Pot-Reduction

Prepared according to **TP6** from (*Z*)-2-(((benzyloxy)carbonyl)amino)vinyl benzoate **1e** (61.8 mg, 0.25 mmol, 1.0 equiv), *N*-acylimine precursor **3a** (66.4 mg, 0.275 mmol, 1.1 equiv), $BF_3 \cdot OEt_2$ (0.13 mL, 0.5 mmol, 2.0 equiv) and L-selectride (1.0 mL, 1 mmol, 4.0 equiv, 1 M solution in THF) in a total of 2.5 mL dichloromethane. Purification by flash chromatography via puriflash XS 420+ machine, HP_15µm_F0012 flash column (*n*-hexane:EtOAc = 95:5 → 50:50) afforded the desired 1,3-diamino-2-ol **5e** as colorless solid (61.7 mg, 50%, isolated *dr* > 98:2; *dr* of the crude mixture >98:2, as determined by ^{1}H NMR analysis of the crude product after aqueous workup).

R_f (*n*-hexane:EtOAc = 7:3) 0.20.

m.p. 185-187 °C.

^{1}H NMR (400 MHz, *Chloroform-d*) δ = 8.01 (d, *J*=8.2, 1.4, 2H), 7.71 (d, *J*=7.6, 2H), 7.62 – 7.52 (m, 1H), 7.50 – 7.27 (m, 15H), 7.06 (d, *J*=8.7, 1H), 5.66 (td, *J*=7.0, 4.5, 1H), 5.55 (t, *J*=7.9, 1H), 5.29 – 5.19 (m, 1H), 5.06 (s, 2H), 3.67 – 3.50 (m, 1H), 3.49 – 3.34 (m, 1H).

^{13}C NMR (101 MHz, *Chloroform-d*) δ = 167.2, 167.1, 156.5, 138.2, 136.4, 134.0, 133.7, 131.8, 130.0, 129.4, 129.3, 128.7, 128.7, 128.6, 128.5, 128.3, 128.3, 127.3, 127.1, 75.2, 67.1, 55.1, 42.2.

IR (ATR, ν in cm^{-1}): 3378 (w), 3340 (w), 3029 (w), 1730 (m), 1702 (s), 1632 (m), 1062 (w), 1525 (s), 1489 (w) 1451 (w), 1341 (w), 1275 (s), 1245 (s), 1169 (w), 1115 (m), 1027 (w), 899 (w), 718 (s), 694 (s).

MS (ESI) m/z calcd for $C_{31}H_{28}N_2NaO_5$ 531.2 [M+Na]$^+$, found 531.4 [M+Na]$^+$.

HRMS (EI) m/z calcd for $C_{31}H_{29}N_2O_5$ 509.2071 [M+H]$^+$, found 509.2083 [M+H]$^+$.

2.6 1,3-Diamino-2-ols from different (*Z*)-Oxyenamides/(Z)-Oxyencarbamates

Typical procedure 7 (TP7)

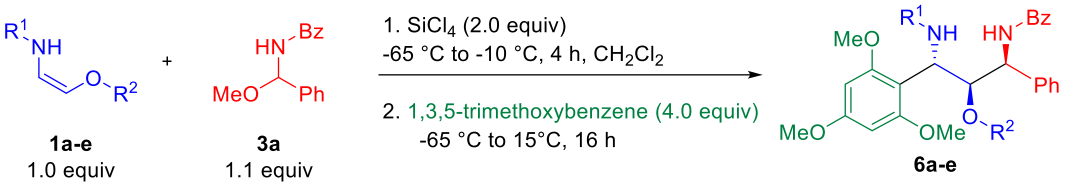

A flame dried and nitrogen flushed Schlenk tube, equipped with a septum and a magnetic stirrer, was charged with (*Z*)-oxyenamide/-encarbamate **1a-e** (1.0 equiv), *N*-acylimine precursor **3a** (1.1 equiv) and dichloromethane (10 mL/mmol). The solution was cooled to -65 °C and $SiCl_4$ (2.0 equiv) was added. The reaction was allowed to warm to -10 °C within 4 h. After TLC analysis showed complete consumption of the (*Z*)-oxyenamide/-encarbamate, the mixture was again cooled to -65 °C and 1,3,5-trimethoxybenzene (4.0 equiv) was added in one portion. The reaction was allowed to warm to 15°C overnight and then quenched with saturated aqueous NH_4Cl (3 mL). The organic layer was separated and the aqueous phase was extracted with dichloromethane (2x 5 mL). The combined organic layers were dried over Na_2SO_4 and the solvent was evaporated under reduced pressure. Purification by flash chromatography afforded the desired 1,2-*syn*-2,3-*anti*-1,3-diamin-2-ol **6a-e** as analytically pure product.

2.6.1 1,3-Diamino-2-ol **6a**

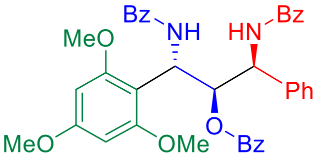

6a

Prepared according to **TP7** from (*Z*)-2-benzamidovinyl benzoate **1a** (66.8 mg, 0.25 mmol, 1.0 equiv), *N*-acylimine precursor **3a** (66.4 mg, 0.275 mmol, 1.1 equiv), $SiCl_4$ (58 µL, 0.5 mmol, 2.0 equiv) and 1,3,5-trimethoxybenzene (172 mg, 1.0 mmol, 4.0 equiv) in a total of 2.5 mL dichloromethane. Purification by flash chromatography via puriflash XS 420+ machine, HP_15µm_F0012 flash column (*n*-hexane:EtOAc = 92:8 → 40:60) afforded the desired 1,3-diamino-2-ol **6a** as a colorless foam (145 mg, 90%, isolated *dr* >98:2; *dr* of the crude mixture >98:2, as determined by ^{1}H NMR analysis of the crude product after aqueous workup).

R_f (*n*-hexane:EtOAc = 6:4) 0.25.

m.p. 110-115 °C.

^{1}H NMR (400 MHz, *Chloroform-d*) δ = 7.83 (d, *J*=9.8, 1H), 7.73 – 7.64 (m, 4H), 7.61 – 7.52 (m, 2H), 7.47 – 7.33 (m, 5H), 7.33 – 7.24 (m, 6H), 7.22 – 7.16 (m, 3H), 7.15 – 7.04 (m, 1H), 6.45 (dd, *J*=9.8, 7.1, 1H), 5.95 – 5.87 (m, 3H), 5.66 (dd, *J*=8.5, 4.1, 1H), 3.65 (s, 6H), 3.60 (s, 3H).

^{13}C NMR (101 MHz, *Chloroform-d*) δ = 166.7, 166.5, 165.3, 161.1, 159.1, 139.6, 134.8, 134.7, 133.1, 131.4, 131.4, 129.9, 129.5, 128.6, 128.5, 128.3, 127.5, 127.3, 127.2, 127.1, 106.4, 91.0, 77.1, 56.0, 55.4, 54.7, 46.2.

IR (ATR, v in cm^{-1}): 3441 (w), 4004 (w), 2970 (w), 1736 (s), 1662 (m), 1600 (m), 1511 (m), 1482 (m), 1452 (m), 1367 (m), 1225 (s), 1204 (s), 1120 (s), 1068 (m), 1028 (m), 950 (w), 814 (w) 705 (s).

MS (ESI) m/z calcd for $C_{28}H_{36}N_2NaO_7$ 667.2 [M+Na]$^+$, found 667.6 [M+Na]$^+$.

HRMS (EI) m/z calcd for $C_{28}H_{36}N_2O_7$ 644.2523 [M+H]$^+$, found 644.2507 [M+H]$^+$.

2.6.2 1,3-Diamino-2-ol **6b**

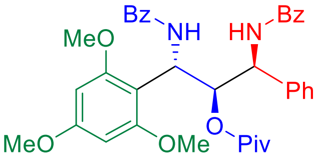

6b

Prepared according to **TP7** from (*Z*)-2-benzamidovinyl pivalate **1b** (mg, 0.25 mmol, 1.0 equiv), *N*-acylimine precoursor **3a** (66.4 mg, 0.275 mmol, 1.1 equiv), $SiCl_4$ (58 µL, 0.5 mmol, 2.0 equiv), 1,3,5-trimethoxybenzene (172 mg, 1.0 mmol, 4 equiv) in a total of 2.5 mL dichloromethane. The reaction was allowed to warm to rt overnight. Purification by flash chromatography via puriflash XS 420+ machine, HP_15µm_F0012 flash column (*n*-hexane:EtOAc = 92:8 → 40:60) afforded the desired 1,3-diamin-2-ol **6b** as a colorless foam (98.4 mg, 63%, isolated *dr* >98: 2; *dr* of the crude mixture >98:2, as determined by ^{1}H NMR analysis of the crude product after aqueous workup).

R_f (*n*-hexane:EtOAc = 6:4) 0.28.

m.p. 104-109 °C.

^{1}H NMR (400 MHz, $CDCl_3$) δ 7.88 – 7.84 (m, 2H), 7.69 (d, J = 10.0 Hz, 1H), 7.64 – 7.59 (m, 2H), 7.53 – 7.47 (m, 1H), 7.45 – 7.40 (m, 3H), 7.36 – 7.32 (m, 4H), 7.27 – 7.23 (m, 2H), 7.17 -7.12 (m, 2H), 6.53 – 6.39 (m, 1H), 6.10 (s, 2H), 5.75 (dd, J = 8.8, 3.4 Hz, 1H), 5.60 (dd, J = 8.3, 3.4 Hz, 1H), 3.85 (s, 6H), 3.77 (s, 3H), 0.85 (s, 9H).

^{13}C NMR (151 MHz, $CDCl_3$) δ 176.5, 166.7, 166.3, 161.1, 159.3, 139.6, 134.9, 134.7, 131.5, 131.4, 128.7, 128.5, 128.5, 127.4, 127.3, 127.1, 126.8, 107.0, 91.2, 75.4, 56.2, 55.5, 54.4, 45.3, 38.8, 27.0.

IR (ATR, v in cm^{-1}): 3447 (w), 2970 (w), 1740 (s), 1664 (m), 1607 (m), 1512 (m), 1482 (m), 1455 (m) 1367 (m) 1223 (m), 1204 (s), 1146 (s), 1122 (s), 1033 (m), 950 (w), 815 (w), 695 (m).

MS (ESI) m/z calcd for $C_{37}H_{40}N_2NaO_7$ 647.3 [M+Na]$^+$, found 647.6 [M+Na]$^+$.

HRMS (EI) m/z calcd for $C_{37}H_{40}N_2O_7$ 624.2836 [M+H]$^+$, found 624.2824 [M+H]$^+$.

2.6.3 1,3-Diamino-2-ol 6c

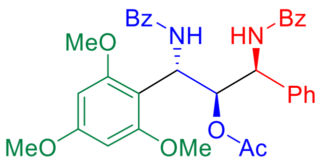

6c

Prepared according to **TP7** from (*Z*)-2-benzamidovinyl acetate **1c** (51.3 mg, 0.25 mmol, 1.0 equiv), *N*-acylimine precoursor **3a** (66.4 mg, 0.275 mmol, 1.1 equiv), $SiCl_4$ (58 µL, 0.5 mmol, 2.0 equiv), 1,3,5-trimethoxybenzene (172 mg, 1.0 mmol, 4.0 equiv) in a total of 2.5 mL dichloromethane. The reaction was allowed to warm to rt overnight. Purification by flash chromatography via puriflash XS 420+ machine, HP_15µm_F0012 flash column (*n*-hexane:EtOAc = 75:25 → 10:90) afforded the desired 1,3-diamino-2-ol **6c** as a colorless foam (111 mg, 76%, isolated *dr* >98:2; *dr* of the crude mixture >98:2, as determined by ^{1}H NMR analysis of the crude product after aqueous workup).

$\mathbf{R_f}$ (*n*-hexane:EtOAc = 6:4) 0.17.

m.p. 98-102 °C.

^{1}H NMR (400 MHz, $CDCl_3$) δ 7.90 (d, *J*=9.9, 1H), 7.81 – 7.76 (m, 2H), 7.69 – 7.63 (m, 2H), 7.52 – 7.25 (m, 10H), 7.22 – 7.16 (m, 1H), 7.11 (d, *J*=8.2, 1H), 6.42 – 6.33 (m, 1H), 6.11 (s, 2H), 5.65 (dd, *J*=7.3, 2.9, 1H), 5.57 (dd, *J*=8.2, 2.8, 1H), 3.84 (s, 6H), 3.77 (s, 3H), 1.79 (s, 3H).

^{13}C NMR (151 MHz, $CDCl_3$) δ 169.4, 166.8, 166.4, 161.2, 159.3, 140.0, 134.9, 134.8, 131.5, 131.4, 128.6, 128.6, 127.5, 127.3, 127.1, 126.7, 106.3, 91.1, 76.7, 56.2, 55.4, 54.2, 46.1, 20.6.

IR (ATR, ν in cm^{-1}): 3440 8w), 2970 (w), 2943 (w), 1742 (s), 1662 (m), 1605 (m), 1512 (m), 1482 (m) 1420 (w), 1368 (m), 1221 (s), 1204 (s), 1149 (w), 1122 (m), 1032 (w), 949 (w), 815 (w), 695 (m).

MS (ESI) m/z calcd for $C_{34}H_{34}N_2NaO_7$ 605.2 [M+Na]$^+$, found 605.5 [M+Na]$^+$.

HRMS (EI) m/z calcd for $C_{34}H_{34}N_2O_7$ 582.2366 [M]$^+$, found 582.2372 [M]$^+$.

2.6.4 1,3-Diamino-2-ol 6e

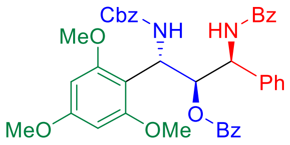

6e

Prepared according to **TP7** from (Z)-2-(((benzyloxy)carbonyl)amino)vinyl benzoate **1e** (74.3 mg, 0.25 mmol, 1.0 equiv), *N*-acylimine precoursor **3a** (66.4 mg, 0.275 mmol, 1.1 equiv), $SiCl_4$ (58 µL, 0.5 mmol, 2.0 equiv), 1,3,5-trimethoxybenzene (172 mg, 1.0 mmol, 4.0 equiv) in a total of 2.5 mL dichloromethane. The reaction was allowed to warm to rt overnight. Purification by flash chromatography via puriflash XS 420+ machine, HP_15µm_F0012 flash column (*n*-hexane:EtOAc = 95:5 → 45:55) afforded the desired 1,3-diamin-2-ol **6e** as a colorless foam (92.2 mg, 58%, crude *dr* = 71:29

as determined by ^{1}H NMR analysis (400 MHz) of the crude product after aqueous workup; overall isolated yield for all diastereomers 117 mg, 74%; *dr* = 79:21, as calculated from the isolated products).

R$_f$ (*n*-hexane:EtOAc = 6:4) 0.33.

m.p. 84-89 °C.

^{1}H NMR (400 MHz, *Chloroform-d*) δ = 7.85 (d, *J*=7.5, 2H), 7.71 (d, *J*=7.7, 2H), 7.55 – 7.39 (m, 4H), 7.34 (p, *J*=7.3, 6.1, 8H), 7.27 – 7.04 (m, 5H), 6.27 (d, *J*=10.3, 1H), 6.06 (dd, *J*=10.2, 8.3, 1H), 5.96 (s, 2H), 5.89 (dd, *J*=8.4, 3.3, 1H), 5.83 – 5.68 (m, 1H), 5.15 – 4.88 (m, 2H), 3.73 (s, 6H), 3.67 (s, 3H).

^{13}C NMR (101 MHz, *Chloroform-d*) δ = 166.9, 164.9, 161.1, 159.0, 156.0, 139.7, 136.6, 135.2, 133.0, 131.4, 129.8, 129.4, 128.7, 128.6, 128.3, 128.2, 127.5, 127.3, 126.9, 106.7, 90.9, 67.1, 56.0, 55.4, 54.3, 47.4.

IR (ATR, ν in cm^{-1}): 3438 (w), 2940 (w), 1715 (m), 1664 (m), 1595 (m), 1500 (m), 1452 (m) 1419 (w), 1260 (m), 1204 (m), 1150 (m), 1111 (s), 1067 (m), 1026 (m), 950 (w), 814 (w), 696 (s).

MS (ESI) m/z calcd for $C_{40}H_{38}N_2NaO_8$ 697.2 [M+Na]$^+$, found 697.6 [M+Na]$^+$.

HRMS (EI) m/z calcd for $C_{28}H_{36}N_2O_7$ 674.2628 [M+H]$^+$, found 674.2617 [M+H]$^+$.

2.7 1,3-Diamino-2-ols from different Nucleophiles

Typical procedure 8 (TP8)

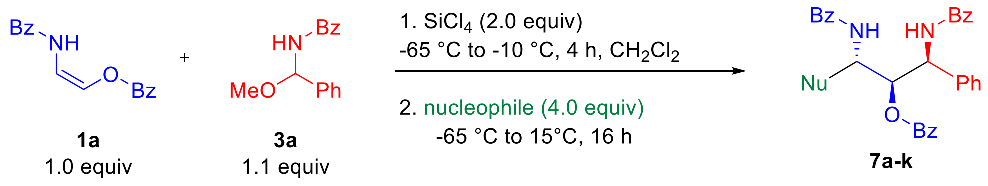

A flame dried and nitrogen flushed Schlenk tube, equipped with a septum and a magnetic stirrer, was charged with (*Z*)-oxyenamide **1a** (1.0 equiv), *N*-acylimine precursor **3a** (1.1 equiv) and dichloromethane (10 mL/mmol). The solution was cooled to -65 °C and $SiCl_4$ (2.0 equiv) was added. The reaction was allowed to warm to -10 °C within 4 h. After TLC analysis showed complete consumption of the (*Z*)-oxyenamide, the mixture was again cooled to -65 °C and the corresponding nucleophile (4.0 equiv) was added in one portion. The reaction was allowed to warm to 15°C overnight and then quenched with saturated aqueous NH_4Cl (3 mL). The organic layer was separated and the aqueous phase was extracted with dichloromethane (2x 5 mL). The combined organic layers were dried over Na_2SO_4 and the solvent was evaporated under reduced pressure. Purification by flash chromatography afforded the desired 1,2-*syn*-2,3-*anti*-1,3-diamino-2-ol **7a-k** as analytically pure product.

2.7.1 1,3-Diamino-2-ol 7a

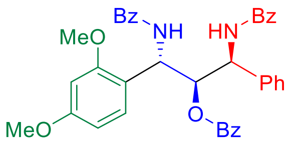

7a

Prepared according to **TP8** from (*Z*)-2-benzamidovinyl benzoate **1a** (66.8 mg, 0.25 mmol, 1.0 equiv), *N*-acylimine precoursor **3a** 66.4 mg, 0.275 mmol, 1.1 equiv), $SiCl_4$ (58 µL, 0.5 mmol, 2.0 equiv), 1,3,-dimethoxybenzene (134 µg, 1 mmol, 4.0 equiv) in a total of 2.5 mL dichloromethane. Purification by flash chromatography via puriflash XS 420+ machine, HP_15µm_F0012 flash column (*n*-hexane:EtOAc = 92:8 → 40:60) afforded the desired 1,3-diamin-2-ol **7a** as a colorless foam (123 mg, 80%, isolated *dr* >98: 2; *dr* of the crude mixture >98:2, as determined by ^{1}H NMR analysis of the crude product after aqueous workup).

R$_f$ (*n*-hexane:EtOAc = 6:4) 0.28.

m.p. 111-117 °C.

^{1}H NMR (400 MHz, *Chloroform-d*) δ = 7.94 (d, J=8.4, 1H), 7.88 – 7.73 (m, 4H), 7.66 – 7.58 (m, 2H), 7.49 (tdd, J=7.7, 4.8, 1.3, 2H), 7.45 – 7.21 (m, 12H), 7.18 (d, J=8.4, 1H), 6.95 (d, J=8.6, 1H), 6.41 (dd, J=8.4, 2.4, 1H), 6.28 (d, J=2.4, 1H), 6.15 (dd, J=6.1, 5.2, 1H), 5.79 (dd, J=8.4, 5.2, 1H), 5.56 (dd, J=8.6, 6.1, 1H), 3.74 (s, 3H), 3.63 (s, 3H).

^{13}C NMR (151 MHz, *Chloroform-d*) δ = 167.0, 166.8, 166.4, 160.9, 158.4, 139.1, 134.4, 134.1, 133.3, 131.6, 131.5, 130.2, 129.7, 129.6, 128.9, 128.6, 128.5, 128.5, 128.0, 127.7, 127.3, 127.1, 117.4, 104.5, 99.1, 76.5, 55.5, 55.5, 54.7, 52.6.

IR (ATR, ν in cm^{-1}): 330 (w), 3061 (w), 2937 (w), 1715 (m), 1646 (m), 1608 (m), 1582 (m), 1507 (s), 1482 (s), 1452 (m), 1262 (s), 1207 (m), 1110 (m), 1207 (m), 931 (w), 833 (w), 705 (s), 695 (s).

MS (ESI) m/z calcd for $C_{38}H_{34}N_2O_6Na$ 637.2 [M+Na]$^+$, found 637.5 [M+Na]$^+$.

HRMS (EI) m/z calcd for $C_{38}H_{34}N_2O_6$ 614.2417 [M]$^+$, found 614.2429 [M]$^+$.

2.7.2 1,3-Diamino-2-ol 7b

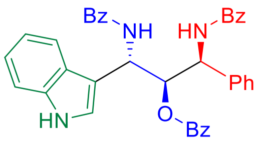

7b

Prepared according to **TP8** from (*Z*)-2-benzamidovinyl benzoate **1a** (66.8 mg, 0.25 mmol, 1.0 equiv), *N*-acylimine precoursor **3a** 66.4 mg, 0.275 mmol, 1.1 equiv), $SiCl_4$ (58 µL, 0.5 mmol, 2.0 equiv), indole (118 mg, 1.0 mmol, 4.0 equiv) in a total of 2.5 mL dichloromethane. Purification by flash chromatography via puriflash XS 420+ machine, HP_15µm_F0012 flash column (*n*-hexane:EtOAc = 92:8 → 40:60) afforded the desired 1,3-diamin-2-ol **7b** as a colorless foam (129 mg, 87%, isolated *dr* >98:2;

dr of the crude mixture >98:2, as determined by ^{1}H NMR analysis of the crude product after aqueous workup).

R$_f$ (*n*-hexane:EtOAc = 6:4) 0.20.

m.p. 138-143 °C.

^{1}H NMR (400 MHz, *Chloroform-d*) δ = 9.46 – 9.34 (m, 1H), 8.10 – 7.96 (m, 2H), 7.79 (d, J=2.4, 1H), 7.61 – 7.58 (m, 2H), 7.49 – 7.44 (m, 3H), 7.40 (d, J=8.0, 1H), 7.38 – 7.28 (m, 6H), 7.26 – 7.16 (m, 8H), 7.11 (td, J=8.8, 8.1, 2.6, 2H), 7.04 – 6.98 (m, 1H), 6.71 (d, J=7.4, 1H), 6.48 (dd, J=9.1, 3.0, 1H), 5.83 (t, J=9.0, 1H), 5.77 (dd, J=7.3, 3.0, 1H).

^{13}C NMR (101 MHz, *Chloroform-d*) δ = 167.7, 167.4, 167.0, 138.0, 136.3, 134.1, 134.0, 133.5, 131.6, 130.0, 129.6, 129.2, 128.7, 128.6, 128.5, 128.5, 127.7, 127.1, 126.9, 126.7, 124.3, 122.5, 120.0, 119.1, 111.7, 110.5, 77.4, 55.3, 47.9.

IR (ATR, ν in cm^{-1}): 3409 (w), 3276 (w), 3060 (w), 2359 (w), 1710 (m), 1639 (m), 1602 (m), 1579 (m), 1511 (m), 1482 (m) 1338 (m), 1265 (s), 1178 (w), 1096 (m), 1070 (m), 928 (w), 744 (m), 693 (s).

MS (ESI) m/z calcd for $C_{38}H_{31}N_3O_4Na$ 616.2 [M+Na]$^+$, found 616.1 [M+Na]$^+$.

HRMS (EI) m/z calcd for $C_{38}H_{31}N_3O_4$ 593.2315 [M]$^+$, found 593.2300 [M]$^+$.

2.7.3 1,3-Diamino-2-ol **7c**

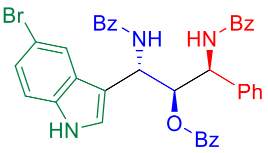

7c

Prepared according to **TP8** from (*Z*)-2-benzamidovinyl benzoate **1a** (66.8 mg, 0.25 mmol, 1.0 equiv), *N*-acylimine precoursor **3a** 66.8 mg, 0.275 mmol, 1.1 equiv), $SiCl_4$ (58 µL, 0.5 mmol, 2.0 equiv), 5-bromoindole (196 mg, 1.0 mmol, 4.0 equiv) in a total of 2.5 mL dichloromethane. Purification by flash chromatography via puriflash XS 420+ machine, HP_15µm_F0012 flash column (*n*-hexane:EtOAc = 92:8 → 30:70) afforded the desired 1,3-diamin-2-ol **7c** as a colorless foam (138 mg, 82%, isolated *dr* >98:2; *dr* of the crude mixture >98:2, as determined by ^{1}H NMR analysis of the crude product after aqueous workup).

R$_f$ (*n*-hexane:EtOAc = 6:4) 0.17.

m.p. >200 °C.

^{1}H NMR (400 MHz, DMSO-*d*$_6$) δ = 11.16 (d, *J*=2.7, 1H), 9.23 (d, *J*=9.3, 1H), 9.01 (d, *J*=8.7, 1H), 7.98 – 7.88 (m, 2H), 7.81 – 7.74 (m, 2H), 7.73 – 7.65 (m, 3H), 7.58 – 7.52 (m, 1H), 7.51 – 7.34 (m, 11H), 7.28 – 7.15 (m, 4H), 7.08 (dd, *J*=8.6, 2.0, 1H), 6.18 (dd, *J*=7.7, 5.7, 1H), 5.85 – 5.73 (m, 2H).

^{13}C NMR (101 MHz, DMSO-*d*$_6$) δ = 167.5, 165.9, 164.7, 139.7, 135.6, 135.0, 134.3, 132.9, 131.0, 130.7, 129.6, 129.5, 128.4, 128.3, 128.1, 128.0, 128.0, 127.6, 127.6, 127.5, 127.2, 125.5, 123.4, 121.4, 113.3, 112.4, 111.3, 76.7, 53.7, 45.8.

IR (ATR, ν in cm^{-1}): 3421 (w), 3247 (w), 3060 (w), 2361 (w), 1717 (w), 1637 (m), 1579 (w), 1509 (m), 1481 (m), 1451 (w), 1314 (w), 1264 (s), 1177 (w), 1106 (m), 1026 (m), 883 (m), 796 (m), 760 (m), 698 (s)

MS (ESI) m/z calcd for $C_{38}H_{30}BrN_3O_4Na$ 694.1 [M+Na]$^+$, found 694.0 [M+Na]$^+$.

HRMS (EI) m/z calcd for $C_{38}H_{32}BrN_3O_4$ 672.1420 [M]$^+$, found 672.1400 [M]$^+$.

2.7.4 1,3-Diamino-2-ol 7d

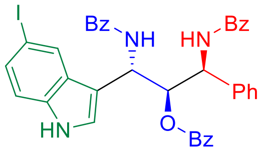

7d

Prepared according to **TP8** from (*Z*)-2-benzamidovinyl benzoate **1a** (66.8 mg, 0.25 mmol, 1.0 equiv), *N*-acylimine precoursor **3a** (66.8 mg, 0.275 mmol, 1.1 equiv), $SiCl_4$ (58 µL, 0.5 mmol, 2.0 equiv), 5-iodoindole (248 mg, 1.0 mmol, 4.0 equiv) in a total of 2.5 mL dichloromethane. Purification by flash chromatography via puriflash XS 420+ machine, HP_15µm_F0012 flash column (*n*-hexane:EtOAc = 92:8 → 40:60) afforded the desired 1,3-diamin-2-ol **7d** as a colorless foam (138 mg, 77%, isolated *dr* >98:2; *dr* of the crude mixture >98:2, as determined by ^{1}H NMR analysis of the crude product after aqueous workup).

R$_f$ (*n*-hexane:EtOAc = 6:4) 0.16.

m.p. 138-143 °C.

^{1}H NMR (400 MHz, *Chloroform-d*) δ = 9.57 (d, *J*=2.6, 1H), 8.06 – 7.99 (m, 2H), 7.70 – 7.61 (m, 4H), 7.55 – 7.47 (m, 3H), 7.44 – 7.38 (m, 1H), 7.32 (ddt, *J*=13.9, 9.8, 6.3, 11H), 7.22 (t, *J*=7.7, 2H), 7.06 (d, *J*=9.0, 1H), 6.93 (d, *J*=8.5, 1H), 6.75 (d, *J*=7.1, 1H), 6.48 (dd, *J*=9.3, 3.1, 1H), 5.69 (t, *J*=9.2, 1H), 5.64 (dd, *J*=7.1, 3.1, 1H).

^{13}C NMR (101 MHz, *Chloroform-d*) δ = 167.8, 167.5, 167.2, 137.7, 135.3, 134.0, 133.9, 133.6, 131.8, 131.7, 130.9, 129.9, 129.4, 129.1, 128.9, 128.9, 128.7, 128.6, 127.9, 127.8, 127.1, 126.9, 125.1, 113.7, 109.9, 83.6, 55.4, 47.8.

IR (ATR, ν in cm^{-1}): 3417 (w), 3270 (w), 3060 (w), 1711 (w), 1640 (m), 1602 (w), 1579 (w), 1509 (m), 1482 (m), 1450 (m), 1314 (w), 1264 (s), 1177 (w), 1096 (m), 1069 (m) 1026 (m), 878 (m), 795 (m), 697 (s).

MS (ESI) m/z calcd for $C_{38}H_{29}IN_3O_4$ 718.1 [M-H]$^-$, found 718.3 [M-H]$^-$.

HRMS (EI) m/z calcd for $C_{38}H_{32}IN_3O_4$ 719.1281 [M]$^+$, found 719.1244 [M]$^+$.

2.7.5 1,3-Diamino-2-ol **7e**

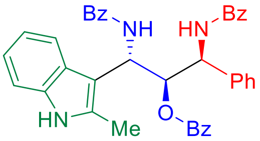

7e

Prepared according to **TP8** from (*Z*)-2-benzamidovinyl benzoate **1a** (66.8 mg, 0.25 mmol, 1.0 equiv), *N*-acylimine precoursor **3a** 66.8 mg, 0.275 mmol, 1.1 equiv), $SiCl_4$ (58 µL, 0.5 mmol, 2.0 equiv), 2-methylindole (131 mg, 1.0 mmol, 4.0 equiv) in a total of 2.5 mL dichloromethane. Purification by flash chromatography via puriflash XS 420+ machine, HP_15µm_F0012 flash column (*n*-hexane:EtOAc = 92:8 → 40:60) afforded the desired 1,3-diamin-2-ol **7e** as a colorless foam (105 mg, 69%, crude *dr* = 80:20, as determined by ^{1}H NMR analysis (400 MHz) of the crude product after aqueous workup; overall isolated yield for all diastereomers 117 mg, 77%; *dr* = 90:10, as calculated from the isolated products).

R_f (*n*-hexane:EtOAc = 6:4) 0.14.

m.p. 125-130 °C.

^{1}H NMR (400 MHz, *Chloroform-d*) δ = 8.11 (s, 1H), 8.02 – 7.96 (m, 2H), 7.74 (d, *J*=8.0, 1H), 7.71 – 7.65 (m, 2H), 7.59 – 7.51 (m, 1H), 7.48 – 7.27 (m, 13H), 7.25 – 7.18 (m, 3H), 7.14 (t, *J*=7.5, 2H), 7.01 (t, *J*=7.5, 1H), 6.63 (d, *J*=8.8, 1H), 6.55 (dd, *J*=7.3, 3.7, 1H), 5.83 (dd, *J*=6.8, 3.7, 1H), 5.53 (dd, *J*=8.8, 7.2, 1H), 2.06 (s, 3H).

^{13}C NMR (101 MHz, *Chloroform-d*) δ = 167.3, 167.2, 166.5, 139.0, 135.9, 134.5, 134.2, 134.0, 133.6, 131.7, 131.5, 130.4, 129.5, 129.2, 128.7, 128.6, 128.5, 128.3, 127.8, 127.1, 126.8, 121.7, 120.1, 119.7, 111.1, 106.8, 55.1, 49.5, 12.1.

IR (ATR, ν in cm^{-1}): 3303 (w), 3060 (w), 2921 (w), 1721 (m), 1638 (m), 1602 (w), 1579 (w), 1517 (m), 1484 (m), 1453 (m), 1311 (w), 1261 (s), 1094 (m), 1069 (m), 1026 (m), 925 (w), 799 (w), 743 (w), 693 (s).

MS (ESI) m/z calcd for $C_{39}H_{33}N_3O_4Na$ 630.2 [M+Na]$^+$, found 630.1 [M+Na]$^+$.

HRMS (EI) m/z calcd for $C_{39}H_{33}N_3O_4$ 607.2471 [M]$^+$, found 607.2483 [M]$^+$.

2.7.6 1,3-Diamino-2-ol **7f**

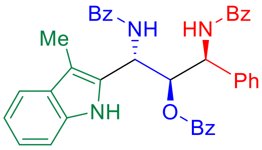

7f

Prepared according to **TP8** from (*Z*)-2-benzamidovinyl benzoate **1a** (66.8 mg, 0.25 mmol, 1.0 equiv), *N*-acylimine precoursor **3a** 87.3 mg, 0.275 mmol, 1.1 equiv), $SiCl_4$ (58 µL, 0.5 mmol, 2.0 equiv), 3-methylindole (131 mg, 1.0 mmol, 4.0 equiv) in a total of 2.5 mL dichloromethane. Purification by flash chromatography via puriflash XS 420+ machine, HP_15µm_F0012 flash column (*n*-hexane:EtOAc = 95:5 → 50:50) afforded the desired 1,3-diamin-2-ol **7f** as a colorless foam (124 mg, 82%, isolated *dr* >98:2;

dr of the crude mixture >98:2, as determined by ^{1}H NMR analysis of the crude product after aqueous workup).

R$_f$ (*n*-hexane:EtOAc = 6:4) 0.49.

m.p. >200 °C.

^{1}H NMR (400 MHz, *Chloroform-d*) δ = 9.52 (s, 1H), 8.01 – 7.95 (m, 2H), 7.69 – 7.64 (m, 2H), 7.62 – 7.59 (m, 2H), 7.58 – 7.51 (m, 2H), 7.49 – 7.40 (m, 5H), 7.39 – 7.28 (m, 9H), 7.25 – 7.20 (m, 1H), 7.16 – 7.10 (m, 1H), 7.01 (d, *J*=7.5, 1H), 6.84 (d, *J*=8.9, 1H), 6.42 (dd, *J*=7.2, 4.5, 1H), 5.70 (dd, *J*=7.5, 4.4, 1H), 5.63 (dd, *J*=9.0, 7.1, 1H), 2.10 (s, 3H).

^{13}C NMR (101 MHz, *Chloroform-d*) δ = 168.0, 167.1, 166.6, 137.6, 136.3, 133.8, 133.8, 133.7, 132.0, 131.8, 129.8, 129.5, 129.4, 129.0, 128.8, 128.8, 128.8, 128.6, 128.5, 127.5, 127.1, 127.0, 122.7, 119.5, 119.1, 111.7, 111.6, 76.5, 54.8, 47.5, 8.7.

IR (ATR, ν in cm^{-1}): 3311 (w), 3059 (w), 3031 (w), 1722 (m), 1636 (s), 1602 (w), 1518 (s), 1485 (s), 1453 (m), 1347 (w), 1267 (s), 1111 (m), 1071 (w), 1027 (w), 921 (w), 798 (w), 707 (s), 685 (w).

MS (ESI) m/z calcd for $C_{39}H_{33}N_3O_4Na$ 630.2 [M+Na]$^+$, found 630.1 [M+Na]$^+$.

HRMS (EI) m/z calcd for $C_{39}H_{33}N_3O_4$ 607.2471 [M]$^+$, found 607.2462 [M]$^+$.

2.7.7 1,3-Diamino-2-ol **7g**

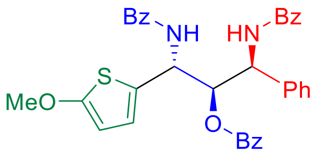

7g

Prepared according to **TP8** from (*Z*)-2-benzamidovinyl benzoate **1a** (66.8 mg, 0.25 mmol, 1.0 equiv), *N*-acylimine precoursor **3a** 87.3 mg, 0.275 mmol, 1.1 equiv), $SiCl_4$ (58 µL, 0.5 mmol, 2.0 equiv), 2-methoxythiophene (102 µL, 1.0 mmol, 4.0 equiv) in a total of 2.5 mL dichloromethane. Purification by flash chromatography via puriflash XS 420+ machine, HP_15µm_F0012 flash column (*n*-hexane:EtOAc = 92:8 → 40:60) afforded the desired 1,3-diamin-2-ol **7g** as a colorless foam (105 mg, 71%, crude *dr* = 90:10, as determined by ^{1}H NMR analysis (400 MHz) of the crude product after aqueous workup; overall isolated yield for all diastereomers 117 mg, 79%; *dr* = 90:10, as calculated from the isolated products).

R$_f$ (*n*-hexane:EtOAc = 6:4) 0.37.

m.p. 110-115 °C.

^{1}H NMR (400 MHz, $CDCl_3$) δ = 8.19 – 8.11 (m, 2H), 7.65 – 7.60 (m, 2H), 7.59 – 7.51 (m, 3H), 7.48 – 7.28 (m, 12H), 7.24 (s, 1H), 6.89 (d, J=8.7, 1H), 6.67 – 6.64 (m, 2H), 6.21 (dd, J=9.3, 2.8, 1H), 6.06 (d, J=3.9, 1H), 5.49 (t, J=9.0, 1H), 5.41 (dd, J=7.3, 2.8, 1H), 3.89 (s, 3H).

^{13}C NMR (101 MHz, $CDCl_3$) δ = 167.8, 167.2, 166.7, 166.5, 138.0, 134.0, 133.9, 133.9, 131.8, 131.6, 130.2, 129.3, 129.1, 128.8, 128.8, 128.7, 128.5, 128.0, 127.1, 126.9, 126.8, 123.3, 76.9, 60.2, 55.5, 51.7.

IR (ATR, ν in cm^{-1}): 3294 (w), 3030 (w), 2970 (w), 1722 (m), 1640 (m), 102 (w), 1579 (w), 1501 (m), 1482 (s), 1429 (w), 1366 (w), 1264 (s), 1206 (s), 1096 (m), 1026 (m), 798 (w), 767 (w), 703 (s).

MS (ESI) m/z calcd for $C_{35}H_{30}N_2NaO_5S$ 613.2 $[M+Na]^+$, found 613.5 $[M+Na]^+$.

HRMS (EI) m/z calcd for $C_{28}H_{24}N_2O_3S$ 468.1508 $[M-BzH]^+$, found 468.1576 $[M-BzH]^+$. (We were not able to obtain HRMS data for the parent molecule without fragmentation. Therefore an additional CHN analysis was performed.)

CHN calcd for $C_{35}H_{30}N_2O_5S$: C 71.17; H 5.12, N 4.74, S 5.43, found: C 71.15, H 5.07, N 4.74, S 5.42.

2.7.8 1,3-Diamino-2-ol 7h

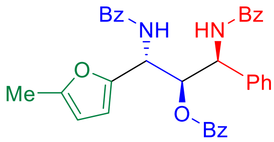

7h

Prepared according to **TP8** from (*Z*)-2-benzamidovinyl benzoate **1a** (66.8 mg, 0.25 mmol, 1.0 equiv), *N*-acylimine precoursor **3a** 87.3 mg, 0.275 mmol, 1.1 equiv), $SiCl_4$ (58 µL, 0.5 mmol, 2.0 equiv), 2-methylfuran (172 mg, 1.0 mmol, 4.0 equiv) in a total of 2.5 mL dichloromethane. Purification by flash chromatography via puriflash XS 420+ machine, HP_15µm_F0012 flash column (*n*-hexane:EtOAc = 92:8 → 40:60) afforded the desired 1,3-diamin-2-ol **7h** as a colorless foam (106 mg, 74%, crude *dr* = 88:12, as determined by ^{1}H NMR analysis (400 MHz) of the crude product after aqueous workup; overall isolated yield for all diastereomers 118 mg, 84%; *dr* = 90:10, as calculated from the isolated products).

R_f (*n*-hexane:EtOAc = 6:4) 0.47.

m.p. 98-105 °C.

^{1}H NMR (400 MHz, *Chloroform-d*) δ = 8.10 – 8.04 (m, 2H), 7.77 – 7.72 (m, 2H), 7.64 – 7.60 (m, 2H), 7.55 (d, *J*=7.4, 1H), 7.53 – 7.28 (m, 13H), 7.12 (d, *J*=7.9, 1H), 7.06 (d, *J*=8.5, 1H), 6.22 (d, *J*=3.1, 1H), 6.08 (dd, *J*=8.2, 3.0, 1H), 5.94 (dd, *J*=3.1, 1.2, 1H), 5.62 – 5.42 (m, 2H), 2.22 (s, 3H).

^{13}C NMR (101 MHz, *Chloroform-d*) δ = 167.4, 166.9, 166.8, 152.6, 147.3, 138.2, 133.9, 133.9, 133.7, 131.8, 131.6, 130.0, 129.4, 129.2, 128.7, 128.6, 128.6, 127.9, 127.3, 127.0, 110.4, 106.6, 55.3, 49.3, 13.6.

IR (ATR, ν in cm^{-1}): 3285 (w), 3062 (w), 2922 (w), 1721 (m), 1640 (m), 1602 (w), 1579 (w), 1517 (m), 1483 (s), 1450 (m), 1264 (s), 1109 (m), 1070 (m), 1025 (m), 935 (w), 793 (w), 693 (s).

MS (ESI) m/z calcd for $C_{35}H_{30}N_2O_5Na$ 581.2 $[M+Na]^+$, found 581.5 $[M+Na]^+$.

HRMS (EI) m/z calcd for $C_{28}H_{24}N_2O_3$ 436.1787 $[M-BzH]^+$, found 436.1799 $[M-BzH]^+$. (We were not able to obtain HRMS data for the parent molecule without fragmentation. Therefore an additional CHN analysis was performed.)

CHN calcd for $C_{35}H_{30}N_2O_5$: C 75.25; H 5.41, N 5.01, found: C 75.14, H 5.49, N 4.96.

2.7.9 1,3-Diamino-2-ol **7i**

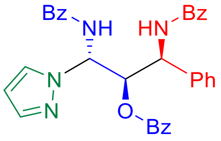

7i

Prepared according to **TP8** from (*Z*)-2-benzamidovinyl benzoate **1a** (66.8 mg, 0.25 mmol, 1.0 equiv), *N*-acylimine precoursor **3a** 87.3 mg, 0.275 mmol, 1.1 equiv), $SiCl_4$ (58 µL, 0.5 mmol, 2.0 equiv), pyrazole (69.5 mg, 1.0 mmol, 4.0 equiv) in a total of 2.5 mL dichloromethane. Purification by flash chromatography via puriflash XS 420+ machine, HP_15µm_F0012 flash column (*n*-hexane:EtOAc = 95:5 → 35:65) afforded the desired 1,3-diamin-2-ol **7i** as a colorless foam (96 mg, 67%, crude *dr* = 83:17, as determined by ^{1}H NMR analysis (400 MHz) of the crude product after aqueous workup; overall isolated yield for all diastereomers 110 mg, 81%; *dr* = 87:13, as calculated from the isolated products).

R_f (*n*-hexane:EtOAc = 6:4) 0.31.

m.p. 194-198 °C.

^{1}H NMR (400 MHz, *Chloroform-d*) δ = 7.91 – 7.85 (m, 2H), 7.83 (d, *J*=9.0, 1H), 7.68 – 7.58 (m, 5H), 7.56 – 7.44 (m, 5H), 7.41 – 7.27 (m, 9H), 7.26 – 7.15 (m, 2H), 6.78 (dd, *J*=9.1, 6.5, 1H), 6.31 (t, *J*=6.6, 1H), 6.16 (t, *J*=2.1, 1H), 5.78 (dd, *J*=8.8, 6.6, 1H).

^{13}C NMR (101 MHz, *Chloroform-d*) δ = 167.0, 166.9, 166.4, 140.9, 138.4, 134.0, 133.8, 132.8, 132.3, 131.8, 130.6, 129.9, 128.8, 128.7, 128.6, 128.5, 127.5, 127.1, 105.8, 75.8, 65.9, 54.6.

IR (ATR, ν in cm^{-1}): 3377 (w), 3060 (w), 3032 (w), 1719 (w), 1662 (m), 1602 (w), 1580 (w), 1520 (m), 1487 (m), 1449 (w), 1317 (m), 1285 (m), 1257 (s), 1092 (m), 1026 (m), 970 (w), 786 (w), 757 (m), 707 (s), 690 (s).

MS (ESI) m/z calcd for $C_{33}H_{28}N_4O_4Na$ 567.2 $[M+Na]^+$, found 567.4 $[M+Na]^+$.

HRMS (EI) m/z calcd for $C_{30}H_{24}N_2O_4$ 476.1736 $[M-C_3H_4N_2]^+$, found 476.1754 $[M-C_3H_4N_2]^+$. (We were not able to obtain HRMS data for the parent molecule without fragmentation. Therefore an additional CHN analysis was performed.)

CHN calcd for $C_{33}H_{28}N_4O_4$: C 72.78; H 5.18, N 10.29, found: C 72.96; H 5.36, N 9.99.

2.7.10 1,3-Diamino-2-ol **7j**

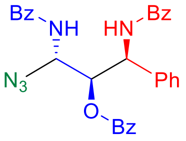

7j

Prepared according to **TP8** from (*Z*)-2-benzamidovinyl benzoate **1a** (66.8 mg, 0.25 mmol, 1.0 equiv), *N*-acylimine precoursor **3a** 87.3 mg, 0.275 mmol, 1.1 equiv), $SiCl_4$ (58 µL, 0.5 mmol, 2.0 equiv),

trimethylsilyl azide (132 µL, 1.0 mmol, 4.0 equiv) in a total of 2.5 mL dichloromethane. Purification by flash chromatography via puriflash XS 420+ machine, HP_15µm_F0012 flash column (*n*-hexane:EtOAc = 92:8 → 40:60) afforded the desired 1,3-diamin-2-ol **7j** as a colorless foam (61.2 mg, 43%, crude *dr* = 75:25, as determined by ^{1}H NMR analysis (400 MHz) of the crude product after aqueous workup; overall isolated yield for all diastereomers 78.4 mg, 57%; *dr* = 87:13, as calculated from the isolated products).

R_f (*n*-hexane:EtOAc = 6:4) 0.47.

m.p. 102-109 °C.

^{1}H NMR (600 MHz, *Chloroform-d*) δ = 8.05 – 8.01 (m, 2H), 7.94 (d, *J*=8.5, 1H), 7.87 – 7.82 (m, 2H), 7.62 – 7.59 (m, 2H), 7.59 – 7.55 (m, 3H), 7.54 – 7.52 (m, 1H), 7.46 – 7.36 (m, 8H), 7.34 – 7.30 (m, 2H), 6.75 (d, *J*=8.6, 1H), 5.88 (dd, *J*=8.5, 3.0, 1H), 5.76 (dd, *J*=8.6, 7.4, 1H), 5.66 (dd, *J*=7.4, 3.1, 1H).

^{13}C NMR (151 MHz, *Chloroform-d*) δ = 167.6, 167.4, 138.1, 134.2, 133.7, 132.8, 132.5, 131.9, 130.2, 129.7, 129.0, 128.9, 128.8, 128.7, 128.5, 127.7, 127.6, 127.0, 76.4, 66.6, 54.8.

IR (ATR, ν in cm^{-1}): 3282 (w), 3062 (w), 2970 (w), 2109 (s), 1725 (m), 1693 (s), 1602 (w), 1580 (w), 1516 (s), 1484 (s), 1450 (m), 1313 (m), 1248 (s), 1147 (w), 1094 (m), 1069 (m), 1026 (m), 926 (W), 799 (w), 691 (s).

MS (ESI) m/z calcd for $C_{30}H_{25}N_5O_4Na$ 542.2 [M+Na]$^+$, found 542.4 [M+Na]$^+$.

HRMS (EI) m/z calcd for $C_{30}H_{24}N_2O_4$ 476.1753 [M-HN$_3$]$^+$, found 476.1736 [M-HN$_3$]$^+$. (We were not able to obtain HRMS data for the parent molecule without fragmentation. Therefore an additional CHN analysis was performed.)

CHN calcd for $C_{30}H_{25}N_5O_4$: C 69.35; H 4.85, N 13.48, found: C 69.06, H 4.86, N 13.28.

2.7.11 1,3-Diamino-2-ol **7k**

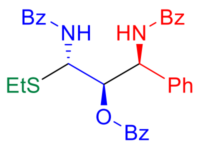

7k

Prepared according to **TP8** from (*Z*)-2-benzamidovinyl benzoate **1a** (66.8 mg, 0.25 mmol, 1.0 equiv), *N*-acylimine precoursor **3a** 87.3 mg, 0.275 mmol, 1.1 equiv), $SiCl_4$ (58 µL, 0.5 mmol, 2.0 equiv), ethanethiol (75.6 µL, 1.0 mmol, 4.0 equiv) in a total of 2.5 mL dichloromethane. Purification by flash chromatography via puriflash XS 420+ machine, HP_15µm_F0012 flash column (*n*-hexane:EtOAc = 95:5 → 70:30) afforded the desired 1,3-diamin-2-ol **7k** as a colorless foam (112 mg, 83%, isolated *dr* = 90:10; *dr* of the crude mixture 83:17 as determined by ^{1}H NMR analysis of the crude product after aqueous workup). Separation of diastereomers was not possible for **7k**. Analytical data are given for the isolate mixture of two diastereomers (*dr* = 90: 10).

R_f (*n*-hexane:EtOAc = 6:4) 0.47.

m.p. 95-100 °C.

^{1}H NMR (400 MHz, *Chloroform-d*) δ = 8.11 – 8.03 (m, 1.7H), 7.98 (dd, *J*=8.4, 1.4, 0.3H), 7.78 (ddd, *J*=11.7, 8.4, 1.4, 0.5H), 7.74 – 7.64 (m, 3.5H), 7.63 – 7.56 (m, 3H), 7.52 – 7.31 (m, 11H), 7.08 (d, *J*=6.5, 1H), 6.92 (d, *J*=8.9, 0.85H), 6.85 (d, *J*=9.3, 0.15H), 5.94 – 5.81 (m, 1.85H), 5.76 – 5.63 (m, 0.3H), 5.42 (dd, *J*=8.9, 1.8, 0.85H), 2.78-2.66 (m, 0.2H), 2.58 (qd, *J*=7.3, 2.3, 1.8H), 1.32 (t, *J*=7.3, 0.2H), 1.19 (dt, *J*=14.8, 7.4, 2.8H). *(peaks not assigned to single stereoisomers)*

^{13}C NMR (101 MHz, *Chloroform-d*) δ = 167.5, 167.4, 167.2, 167.0, 166.8, 166.6, 138.1, 138.0, 134.3, 134.0, 133.9, 133.8, 133.7, 133.4, 132.1, 132.1, 131.8, 131.7, 130.2, 129.9, 129.3, 129.3, 129.0, 128.9, 128.8, 128.8, 128.7, 128.6, 128.6, 127.7, 127.4, 127.2, 127.2, 127.1, 78.5, 77.9, 56.5, 55.9, 54.9, 54.4, 25.9, 25.5, 14.9, 14.9. *(peaks not assigned to single stereoisomers)*

IR (ATR, ν in cm^{-1}): 3295 (w), 3061 (w) 2967 (w), 1710 (w), 1638 (m), 1602 (w), 1579 (w), 1513 (m), 1482 (s), 1449 (m), 1313 (m), 1259 (s), 1179 (w), 1108 (m), 1070 (m), 1026 (m), 928 (w), 799 (w), 693 (s).

MS (ESI) m/z calcd for $C_{32}H_{30}N_2O_4SNa$ 561.2 [M+Na]$^+$, found 561.4 [M+Na]$^+$.

HRMS (EI) m/z calcd for $C_{30}H_{25}N_2O_4$ 477.1814 [M-C_2H_5S]$^+$, found 477.1823 [M-C_2H_5S]$^+$. (We were not able to obtain HRMS data for the parent molecule without fragmentation. Therefore an additional CHN analysis was performed.)

CHN calcd for $C_{32}H_{30}N_2O_4S$: C 71.35; H 5.61, N 5.20, S 5.95, found: C 71.07, H 5.64, N 5.23, S 6.15.

2.8 1,3-Diamino-2-ols from different *N*-Acylimine precoursors

Typical procedure 9 (TP9)

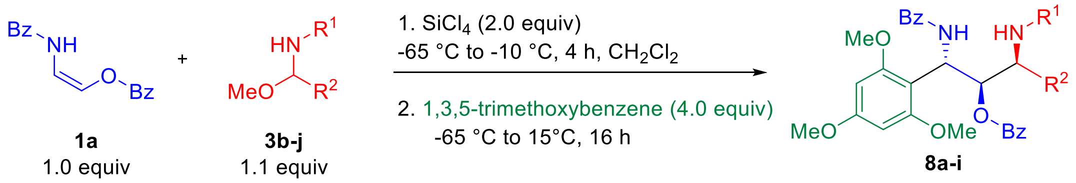

A flame dried and nitrogen flushed Schlenk tube, equipped with a septum and a magnetic stirrer, was charged with (*Z*)-oxyenamide **1a** (1.0 equiv), *N*-acylimine precursor **3b-j** (1.1 equiv) and dichloromethane (10 mL/mmol). The solution was cooled to -65 °C and $SiCl_4$ (2.0 equiv) was added. The reaction was allowed to warm to -10 °C within 4 h. After TLC analysis showed complete consumption of the (*Z*)-oxyenamide, the mixture was again cooled to -65 °C and 1,3,5-trimethoxybenzene (4.0 equiv) was added in one portion. The reaction was allowed to warm to 15°C overnight and then quenched with saturated aqueous NH_4Cl (5 mL). The organic layer was separated and the aqueous phase was extracted with dichloromethane (2x). The combined organic layers were dried over Na_2SO_4 and the solvent was evaporated under reduced pressure. Purification by flash chromatography afforded the desired 1,2-*syn*-2,3-*anti*-1,3-diamino-2-ol **8a-i** as analytically pure product.

2.8.1 1,3-Diamino-2-ol **8a**

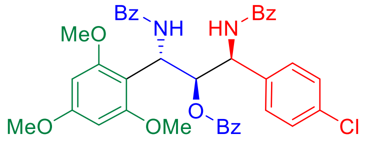

8a

Prepared according to **TP9** from (*Z*)-2-benzamidovinyl benzoate **1a** (66.8 mg, 0.25 mmol, 1.0 equiv), *N*-acylimine precoursor **3b** (75.8 mg, 0.275 mmol, 1.1 equiv), $SiCl_4$ (58 µL, 0.5 mmol, 2.0 equiv), 1,3,5-trimethoxybenzene (172 mg, 1.0 mmol, 4.0 equiv) in a total of 2.5 mL dichloromethane. Purification by flash chromatography via puriflash XS 420+ machine, HP_15µm_F0012 flash column (*n*-hexane:EtOAc = 92:8 → 40:60) afforded the desired 1,3-diamino-2-ol **8a** as a colorless foam (142 mg, 84%, isolated *dr* >98:2; *dr* of the crude mixture >98:2, as determined by ^{1}H NMR analysis of the crude product after aqueous workup).

R_f (*n*-hexane:EtOAc = 6:4) 0.26.

m.p. 93-101 °C.

^{1}H NMR (400 MHz, *Chloroform-d*) δ 7.93 (d, J = 9.7 Hz, 1H), 7.79 – 7.70 (m, 4H), 7.68 – 7.60 (m, 2H), 7.53 – 7.45 (m, 3H), 7.42 – 7.32 (m, 9H), 7.23 (d, J = 8.5 Hz, 2H), 6.48 (dd, J = 9.7, 7.1 Hz, 1H), 5.98 (s, 2H), 5.92 (dd, J = 7.1, 4.0 Hz, 1H), 5.68 (dd, J = 8.3, 3.9 Hz, 1H), 3.71 – 3.67 (m, 9H).

^{13}C NMR (151 MHz, *Chloroform-d*) δ 166.7, 166.6, 165.2, 161.2, 159.0, 138.2, 134.6, 134.5, 133.3, 133.2, 131.6, 131.5, 129.7, 129.5, 128.8, 128.7, 128.6, 128.6, 128.4, 127.3, 127.1, 106.1, 91.0, 56.0, 55.4, 54.3, 46.1.

IR (ATR, ν in cm^{-1}): 3436 (w), 2939 (w), 2839 (w), 1721 (w), 1663 (m), 1596 (m), 1512 (m), 1484 (s), 1452 (m),1329 (w), 1261 (m), 1203 (w), 1149 (m), 1121 (s), 1092 (s), 1068 (m), 1028 (m), 950 (w), 815 (m), 709 (s).

MS (ESI) m/z calcd for $C_{39}H_{35}ClN_2O_7Na$ 701.2 $[M+Na]^+$, found 701.6 $[M+Na]^+$.

HRMS (EI) m/z calcd for $C_{39}H_{35}ClN_2O_7$ 678.2133 $[M]^+$, found 678.2152 $[M]^+$.

2.8.2 1,3-Diamino-2-ol **8b**

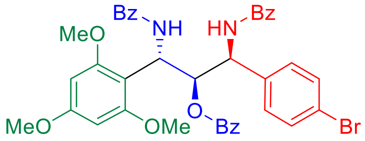

8b

Prepared according to **TP9** from (*Z*)-2-benzamidovinyl benzoate **1a** (66.8 mg, 0.25 mmol, 1.0 equiv), *N*-acylimine precoursor **3c** (75.8 mg, 0.275 mmol, 1.1 equiv), $SiCl_4$ (58 µL, 0.5 mmol, 2.0 equiv), 1,3,5-trimethoxybenzene (172 mg, 1.0 mmol, 4.0 equiv) in a total of 2.5 mL dichloromethane. Purification by flash chromatography via puriflash XS 420+ machine, HP_15µm_F0012 flash column (*n*-hexane:EtOAc = 92:8 → 40:60) afforded the desired 1,3-diamino-2-ol **8b** as a colorless foam (95.7 mg,

53%, isolated *dr* >98:2; *dr* of the crude mixture >98:2, as determined by ^{1}H NMR analysis of the crude product after aqueous workup).

R$_f$ (*n*-hexane:EtOAc = 6:4) 0.26.

m.p. 102-111 °C.

^{1}H NMR (400 MHz, *Chloroform-d*) δ = 7.93 (d, J=9.8, 1H), 7.74 (ddd, J=10.1, 8.2, 1.3, 4H), 7.69 – 7.61 (m, 2H), 7.56 – 7.42 (m, 3H), 7.42 – 7.28 (m, 11H), 6.47 (dd, J=9.7, 7.1, 1H), 5.98 (s, 2H), 5.91 (dd, J=7.1, 4.0, 1H), 5.66 (dd, J=8.3, 4.0, 1H), 3.71 (s, 6H), 3.67 (s, 3H).

^{13}C NMR (151 MHz, *Chloroform-d*) δ = 166.7, 166.6, 165.2, 161.2, 159.0, 138.7, 134.6, 134.5, 133.2, 131.6, 131.6, 131.5, 129.7, 129.5, 129.2, 128.6, 128.6, 128.4, 127.3, 127.1, 121.6, 106.2, 91.0, 56.0, 55.4, 54.4, 46.1.

IR (ATR, ν in cm^{-1}): 3435 (w), 3062 (w), 2939 (w), 1722 (w), 1663 (m), 1596 (m), 1512 (s), 1483 (s), 1452 (m), 1429 (w), 1310 (w), 1261 (m), 1203 (m), 1149 (m), 1121 (s), 1068 (m), 1008 (m), 950 (w), 815 (m), 708 (s).

MS (ESI) m/z calcd for $C_{39}H_{35}BrN_2O_7Na$ 745.2 [M+Na]$^+$, found 745.5 [M+Na]$^+$.

HRMS (EI) m/z calcd for $C_{39}H_{35}BrN_2O_7$ 722.1628 [M]$^+$, found 722.1638 [M]$^+$.

2.8.3 1,3-Diamino-2-ol **8c**

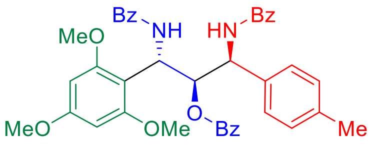

8c

Prepared according to **TP9** from (*Z*)-2-benzamidovinyl benzoate **1a** (66.8 mg, 0.25 mmol, 1.0 equiv), *N*-acylimine precoursor **3d** (70.2 mg, 0.275 mmol, 1.1 equiv), $SiCl_4$ (58 µL, 0.5 mmol, 2.0 equiv), 1,3,5-trimethoxybenzene (172 mg, 1.0 mmol, 4.0 equiv) in a total of 2.5 mL dichloromethane. Purification by flash chromatography via puriflash XS 420+ machine, HP_15µm_F0012 flash column (*n*-hexane:EtOAc = 92:8 → 40:60) afforded the desired 1,3-diamino-2-ol **8c** as a colorless foam (153 mg, 93%, isolated *dr* >98:2; *dr* of the crude mixture >98:2, as determined by ^{1}H NMR analysis of the crude product after aqueous workup).

R$_f$ (*n*-hexane:EtOAc = 6:4) 0.23.

m.p. 98-101 °C.

^{1}H NMR (400 MHz, *Chloroform-d*) δ 7.86 – 7.74 (m, 3H), 7.74 – 7.66 (m, 2H), 7.65 – 7.57 (m, 2H), 7.52 – 7.42 (m, 3H), 7.37 – 7.31 (m, 8H), 7.14 (d, J = 8.5 Hz, 1H), 7.05 (d, J = 7.9 Hz, 2H), 6.50 (dd, J = 9.8, 7.2 Hz, 1H), 6.00 – 5.93 (m, 3H), 5.68 (dd, J = 8.5, 4.5 Hz, 1H), 3.72 (s, 6H), 3.68 (s, 3H), 2.24 (s, 3H).

^{13}C NMR (151 MHz, *Chloroform-d*) δ 166.6, 166.3, 165.6, 161.0, 159.1, 137.1, 136.8, 134.9, 134.8, 133.1, 131.3, 131.3, 130.0, 129.6, 129.3, 128.5, 128.5, 128.4, 127.2, 127.2, 127.1, 106.6, 91.0, 56.0, 55.4, 54.6, 46.3, 21.2.

IR (ATR, v in cm^{-1}): 3439 (w), 2939 (w), 2839 (w),), 1721 (w), 1663 (m), 1596 (m), 1512 (s), 1483 (s), 1452 (m), 1419 (w), 1311 (w), 1260 (m), 1203 (m), 1149 (m), 1120 (s), 1068 (m), 1028 (m), 949 (w), 814 (m), 708 (s).

MS (ESI) m/z calcd for $C_{40}H_{38}N_2O_7Na$ 681.3 $[M+Na]^+$, found 681.6 $[M+Na]^+$.

HRMS (EI) m/z calcd for $C_{40}H_{38}N_2O_7$ 658.2679 $[M]^+$, found 658.2688 $[M]^+$.

2.8.4 1,3-Diamino-2-ol **8d**

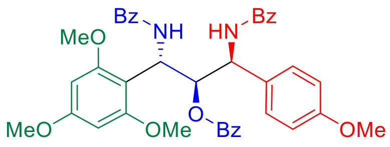

8d

Prepared according to **TP9** from (*Z*)-2-benzamidovinyl benzoate **1a** (66.8 mg, 0.25 mmol, 1.0 equiv), *N*-acylimine precoursor **3e** (74.6 mg, 0.275 mmol, 1.1 equiv), $SiCl_4$ (58 µL, 0.5 mmol, 2.0 equiv), 1,3,5-trimethoxybenzene (172 mg, 1.0 mmol, 4.0 equiv) in a total of 2.5 mL dichloromethane. Purification by flash chromatography via puriflash XS 420+ machine, HP_15µm_F0012 flash column (*n*-hexane:EtOAc = 92:8 → 40:60) afforded the desired 1,3-diamino-2-ol **8d** as a colorless foam (160 mg, 95%, isolated *dr* >98:2; *dr* of the crude mixture >98:2, as determined by ^{1}H NMR analysis of the crude product after aqueous workup).

R$_f$ (*n*-hexane:EtOAc = 6:4) 0.17.

m.p. 79-85 °C.

^{1}H NMR (400 MHz, *Chloroform-d*) δ 7.87 – 7.80 (m, 1H), 7.79 – 7.75 (m, 2H), 7.73 – 7.69 (m, 2H), 7.64 – 7.60 (m, 2H), 7.52 – 7.48 (m, 1H), 7.46 – 7.42 (m, 2H), 7.39 – 7.33 (m, 8H), 7.16 (d, J=8.4, 1H), 6.78 (d, J=8.7, 2H), 6.49 (dd, J=9.8, 7.1, 1H), 5.98 (s, 2H), 5.95 (dd, J=7.1, 4.7, 1H), 5.66 (dd, J=8.5, 4.6, 1H), 3.72 (s, 6H), 3.71 (s, 3H), 3.68 (s, 3H).

^{13}C NMR (151 MHz, *Chloroform-d*) δ 166.6, 166.4, 165.6, 161.1, 159.1, 159.0, 134.9, 134.8, 133.1, 131.9, 131.34, 131.3, 129.6, 128.5, 128.5, 128.4, 127.5, 127.2, 127.1, 114.0, 106.6, 91.0, 56.0, 55.4, 55.3, 54.2, 46.3.

IR (ATR, v in cm^{-1}): 3438 (w), 2937 (w), 1720 (w), 1656 (m), 1607 (m), 1511 (s), 1484 (m), 1452 (m), 1330 (w), 1247 (s), 1203 (m), 1179 (m), 1149 (m), 1121 (s), 1068 (m), 1028 (m), 950 (w), 815 (m), 709 (s).

MS (ESI) m/z calcd for $C_{40}H_{38}N_2O_8Na$ 697.3 $[M+Na]^+$, found 697.6 $[M+Na]^+$.

HRMS (EI) m/z calcd for $C_{40}H_{38}N_2O_8$ 674.2628 $[M]^+$, found 674.2633 $[M]^+$.

2.8.5 1,3-Diamino-2-ol **8e**

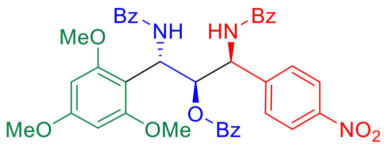

8e

Prepared according to **TP9** from (*Z*)-2-benzamidovinyl benzoate **1a** (66.8 mg, 0.25 mmol, 1.0 equiv), *N*-acylimine precoursor **3f** (79.0 mg, 0.275 mmol, 1.1 equiv), $SiCl_4$ (58 µL, 0.5 mmol, 2.0 equiv), 1,3,5-trimethoxybenzene (172 mg, 1.0 mmol, 4.0 equiv) in a total of 2.5 mL dichloromethane. Purification by flash chromatography via puriflash XS 420+ machine, HP_15µm_F0012 flash column (*n*-hexane:EtOAc = 92:8 → 40:60) afforded the desired 1,3-diamino-2-ol **8e** as a colorless foam (98.8 mg, 57%, isolated *dr* >98:2; *dr* of the crude mixture >98:2, as determined by ^{1}H NMR analysis of the crude product after aqueous workup).

$\mathbf{R_f}$ (*n*-hexane:EtOAc = 6:4) 0.20.

m.p. 119-123 °C.

^{1}**H NMR** (400 MHz, *Chloroform-d*) δ 8.11 (d, *J* = 8.7 Hz, 2H), 7.97 (d, *J* = 9.6 Hz, 1H), 7.83 – 7.77 (m, 2H), 7.72 – 7.64 (m, 4H), 7.61 (d, *J* = 8.7 Hz, 2H), 7.55 – 7.33 (m, 10H), 6.50 (dd, *J* = 9.6, 7.3 Hz, 1H), 5.99 (s, 2H), 5.94 (dd, *J* = 7.2, 3.5 Hz, 1H), 5.76 (dd, *J* = 7.7, 3.4 Hz, 1H), 3.73 (s, 6H), 3.68 (s, 3H).

^{13}**C NMR** (151 MHz, *Chloroform-d*) δ 167.0, 166.7, 165.0, 161.3, 147.4, 147.3, 134.3, 134.2, 133.4, 131.8, 131.8, 129.5, 129.4, 128.7, 128.5, 128.3, 127.4, 127.1, 123.8, 105.8, 91.1, 76.6, 56.1, 55.5, 54.8, 46.1.

IR (ATR, ν in cm^{-1}): 3431 (w), 2940 (w), 1723 (m), 1663 (m), 1600 (m), 1513 (s), 1483 (s), 1453 (m), 1419 (w), 1344 (s), 1257 (s), 1224 (w), 1204 (m), 1150 (m), 1121 (s), 1068 (m), 1028 (m), 950 (w), 815 (w), 708 (s).

MS (ESI) m/z calcd for $C_{39}H_{35}N_3O_9Na$ 712.2 [M+Na]$^+$, found 712.6 [M+Na]$^+$.

HRMS (EI) m/z calcd for $C_{39}H_{35}N_3O_9$ 689.2373 [M]$^+$, found 689.2385 [M]$^+$.

2.8.6 1,3-Diamino-2-ol **8f**

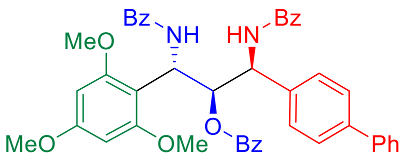

8f

Prepared according to **TP9** from (*Z*)-2-benzamidovinyl benzoate **1a** (66.8 mg, 0.25 mmol, 1.0 equiv), *N*-acylimine precoursor **3g** (87.3 mg, 0.275 mmol, 1.1 equiv), $SiCl_4$ (58 µL, 0.5 mmol, 2.0 equiv), 1,3,5-trimethoxybenzene (172 mg, 1.0 mmol, 4.0 equiv) in a total of 2.5 mL dichloromethane. Purification by flash chromatography via puriflash XS 420+ machine, HP_15µm_F0012 flash column (*n*-hexane:EtOAc = 92:8 → 40:60) afforded the desired 1,3-diamino-2-ol **8h** as a colorless foam (156 mg,

86%, isolated *dr* >98:2; *dr* of the crude mixture >98:2, as determined by ^{1}H NMR analysis of the crude product after aqueous workup).

R$_f$ (*n*-hexane:EtOAc = 6:4) 0.19.

m.p. 115-119 °C.

^{1}H NMR (400 MHz, *Chloroform-d*) δ 7.89 (d, *J* = 9.8 Hz, 1H), 7.78 – 7.74 (m, 4H), 7.64 – 7.60 (m, 2H), 7.56 – 7.27 (m, 19H), 6.54 (dd, *J* = 9.8, 7.2 Hz, 1H), 6.02 (dd, *J* = 7.2, 4.5 Hz, 1H), 5.98 (s, 2H), 5.80 – 5.72 (m, 1H), 3.72 (s, 6H), 3.68 (s, 3H).

^{13}C NMR (151 MHz, *Chloroform-d*) δ 166.7, 166.5, 165.5, 161.1, 159.1, 141.0, 140.4, 138.8, 134.8, 134.7, 133.1, 131.4, 129.9, 129.6, 128.8, 128.6, 128.6, 128.4, 127.8, 127.4, 127.3, 127.2, 127.1, 106.5, 91.0, 56.0, 55.4, 54.6, 46.3.

IR (ATR, ν in cm^{-1}): 3439 (w), 2939 (w), 1721 (w), 1663 (m), 1597 (m), 1512 (m), 1483 (s), 1452 (m), 1418 (w), 1311 (w), 1261 (m), 1203 (m), 1149 (m), 1120 (s), 1068 (m), 1028 (m), 950 (w), 815 (w), 708 (s), 693 (s).

MS (ESI) m/z calcd for $C_{45}H_{40}N_2O_7Na$ 743.3 [M+Na]$^+$, found 743.6 [M+Na]$^+$.

HRMS (EI) m/z calcd for $C_{45}H_{40}N_2O_7$ 720.2836 [M]$^+$, found 720.2849 [M]$^+$.

2.8.7 1,3-Diamino-2-ol **8g**

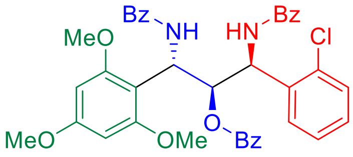

8g

Prepared according to **TP9** from (*Z*)-2-benzamidovinyl benzoate **1a** (66.8 mg, 0.25 mmol, 1.0 equiv), *N*-acylimine precoursor **3h** (75.8 mg, 0.275 mmol, 1.1 equiv), $SiCl_4$ (58 µL, 0.5 mmol, 2.0 equiv), 1,3,5-trimethoxybenzene (172 mg, 1.0 mmol, 4.0 equiv) in a total of 2.5 mL dichloromethane. Purification by flash chromatography via puriflash XS 420+ machine, HP_15µm_F0012 flash column (*n*-hexane:EtOAc = 92:8 → 40:60) afforded the desired 1,3-diamino-2-ol **8f** as a colorless foam (93.8 mg, 55%, isolated *dr* >98:2; *dr* of the crude mixture >98:2, as determined by ^{1}H NMR analysis of the crude product after aqueous workup).

R$_f$ (*n*-hexane:EtOAc = 6:4) 0.21.

m.p. 103-110 °C.

^{1}H NMR (400 MHz, *Chloroform-d*) δ 7.89 (d, *J* = 10.1 Hz, 1H), 7.85 (d, *J* = 7.2 Hz, 2H), 7.74 – 7.70 (m, 4H), 7.54 – 7.42 (m, 5H), 7.41 – 7.32 (m, 5H), 7.25 – 7.21 (m, 1H), 7.13 – 7.06 (m, 1H), 7.03 – 6.99 (m, 1H), 6.56 (dd, *J* = 9.8, 8.6 Hz, 1H), 6.13 (dd, *J* = 8.4, 2.6 Hz, 1H), 6.07 (dd, *J* = 7.6, 2.5 Hz, 1H), 6.01 (s, 2H), 3.83 (s, 6H), 3.68 (s, 3H).

^{13}C NMR (151 MHz, *Chloroform-d*) δ 166.8, 166.5, 164.8, 161.1, 137.2, 135.0, 135.0, 133.1, 132.8, 131.5, 131.4, 130.0, 129.8, 129.4, 128.8 128.7, 128.6, 128.4, 127.7, 127.4, 127.2, 126.9, 106.7, 91.2, 75.2, 56.2, 55.4, 52.2, 45.7.

IR (ATR, ν in cm^{-1}): 3441 (w), 2940 (w), 1725 (m), 1666 (s), 1595 (m), 1509 (s), 1482 (s), 1419 (w),1329 (w), 1257 (m), 1203 (m), 1149 (m), 1121 (s), 1067 (m), 1030 (m), 950 (w), 815 (w), 706 (s).

MS (ESI) m/z calcd for $C_{39}H_{35}ClN_2O_7Na$ 701.2 [M+Na]$^+$, found 701.5 [M+Na]$^+$.

HRMS (EI) m/z calcd for $C_{39}H_{35}ClN_2O_7$ 678.2133 [M]$^+$, found 678.2123 [M]$^+$.

2.8.8 1,3-Diamino-2-ol 8h

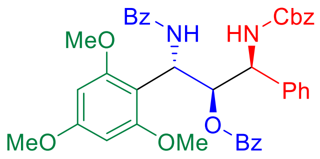

8h

Prepared according to **TP9** from (*Z*)-2-benzamidovinyl benzoate **1a** (66.8 mg, 0.25 mmol, 1.0 equiv), *N*-acylimine precoursor **3i** (74.6 mg, 0.275 mmol, 1.1 equiv), $SiCl_4$ (58 µL, 0.5 mmol, 2.0 equiv), 1,3,5-trimethoxybenzene (172 mg, 1.0 mmol, 4.0 equiv) in a total of 2.5 mL dichloromethane. Purification by flash chromatography via puriflash XS 420+ machine, HP_15µm_F0012 flash column (*n*-hexane:EtOAc = 92:8 → 40:60) afforded the desired 1,3-diamino-2-ol **8i** as a colorless foam (64.4 mg, 38%, isolated *dr* >98:2; *dr* of the crude mixture >98:2, as determined by ^{1}H NMR analysis of the crude product after aqueous workup).

R$_f$ (*n*-hexane:EtOAc = 6:4) 0.30.

m.p. 102-107 °C.

^{1}H NMR (400 MHz, *Chloroform-d*) δ = 7.81 (d, *J*=9.9, 1H), 7.77 (d, *J*=7.7, 2H), 7.74 – 7.69 (m, 2H), 7.55 – 7.45 (m, 2H), 7.44 – 7.27 (m, 10H), 7.29 – 7.07 (m, 4H), 6.43 (t, *J*=9.0, 1H), 5.99 (s, 2H), 5.91 (dd, *J*=7.8, 3.8, 1H), 5.80 (d, *J*=9.1, 1H), 5.34 (dd, *J*=9.0, 4.1, 1H), 5.05 (s, 2H), 3.74 (s, 6H), 3.69 (s, 3H).

^{13}C NMR (101 MHz, *Chloroform-d*) δ = 166.4, 165.1, 161.0, 159.2, 155.8, 140.0, 136.6, 135.1, 133.0, 131.3, 129.8, 129.6, 128.6, 128.4, 128.3, 128.1, 127.5, 127.1, 126.9, 106.6, 91.0, 77.0, 67.0, 56.0, 55.8, 55.3, 45.9.

IR (ATR, ν in cm^{-1}): 3438 (w), 2939 (w), 1715 (m), 1664 (m), 1594 (m), 1508 (m), 1484 (m), 1452 (m), 1330 (w), 1259 (m), 1223 (m), 1204 (m), 1149 (m), 1119 (s), 1053 8m), 1027 (m), 949 8w), 815 (w), 698 (s).

MS (ESI) m/z calcd for $C_{40}H_{38}N_2O_8Na$ 697.3 [M+Na]$^+$, found 697.6 [M+Na]$^+$.

HRMS (EI) m/z calcd for $C_{28}H_{36}N_2O_7$ 674.2628 [M+H]$^+$, found 674.2646 [M+H]$^+$.

2.8.9 1,3-Diamino-2-ol **8i**

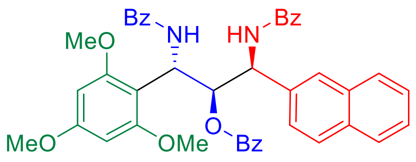

8i

Prepared according to **TP9** from (*Z*)-2-benzamidovinyl benzoate **1a** (66.8 mg, 0.25 mmol, 1.0 equiv), *N*-acylimine precoursor **3j** (80.0 mg, 0.275 mmol, 1.1 equiv), $SiCl_4$ (58 µL, 0.5 mmol, 2.0 equiv), 1,3,5-trimethoxybenzene (172 mg, 1.0 mmol, 4.0 equiv) in a total of 2.5 mL dichloromethane. Purification by flash chromatography via puriflash XS 420+ machine, HP_15µm_F0012 flash column (*n*-hexane:EtOAc = 92:8 → 40:60) afforded the desired 1,3-diamino-2-ol **8g** as a colorless foam (107 mg, 61%, isolated *dr* >98:2; *dr* of the crude mixture >98:2, as determined by ^{1}H NMR analysis of the crude product after aqueous workup).

R$_f$ (*n*-hexane:EtOAc = 6:4) 0.17.

m.p. 103-110 °C.

^{1}H NMR (400 MHz, *Chloroform-d*) δ 7.94 (s, 1H), 7.88 (d, *J* = 9.7 Hz, 1H), 7.81 – 7.69 (m, 7H), 7.59 – 7.55 (m, 3H), 7.51 – 7.28 (m, 12H), 6.57 (dd, *J* = 9.8, 7.1 Hz, 1H), 6.09 (dd, *J* = 7.0, 4.5 Hz, 1H), 5.97 (s, 2H), 5.88 (dd, *J* = 8.5, 4.5 Hz, 1H), 3.67 – 3.66 (m, 9H).

^{13}C NMR (151 MHz, *Chloroform-d*) δ 166.7, 166.5, 165.5, 161.1, 159.1, 137.1, 134.8, 134.7, 133.5, 133.1, 133.0, 131.4, 131.4, 129.9, 129.6, 128.6, 128.5, 128.4, 128.3, 128.2, 127.6, 127.3, 127.1, 126.3, 126.0, 125.9, 125.5, 106.5, 91.0, 56.0, 55.4, 54.9, 46.3.

IR (ATR, ν in cm^{-1}): 3438 (w), 2939 (w), 1720 (w), 1663 (m), 1597 (m), 1509 (s), 1482 (s), 1452 (m), 1419 (w), 1314 (w), 1262 (m), 1203 (m), 1149 (m), 1119 (s), 1068 (m), 1028 (m), 950 (m), 815 (m), 752 (w), 709 (s).

MS (ESI) m/z calcd for $C_{43}H_{38}N_2O_7Na$ 717.3 [M+Na]$^+$, found 717.6 [M+Na]$^+$.

HRMS (EI) m/z calcd for $C_{43}H_{38}N_2O_7$ 694.2679 [M]$^+$, found 694.2672 [M]$^+$.

2.9 Deprotection

2.9.1 1,3-Diamino-2-ol **9a**

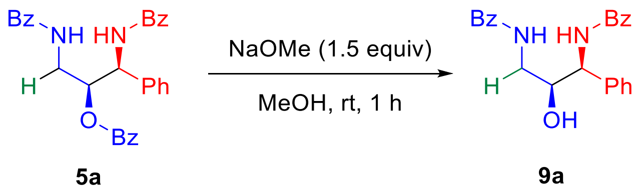

To a solution of 1,3-diamin-2-ol **5a** (120 mg, 0.25 mmol, 1.0 equiv) in methanol (3.5 mL) was added at room temperature sodium methoxide (21mg, 0.375 mmol, 1.5 equiv) in one portion. After stirring for 1 h the reaction mixture was quenched with saturated aqueous NH_4Cl (5 mL). The aqueous phase was extracted with dichloromethane (3x 5 mL) and the combined organic layers were dried over Na_2SO_4

before the solvent was evaporated under reduced pressure. Purification by flash chromatography via puriflash XS 420+ machine, HP_15µm_F0012 flash column (*n*-hexane:EtOAc = 90:10 → 20:80) afforded the deprotected 1,2-*syn*-1,3-diamin-2-ol **9a** as a colorless solid (80.5 mg, 86%, isolated *dr* >98:2; *dr* of the crude mixture >98:2, as determined by ^{1}H NMR analysis of the crude product after aqueous workup).

R$_f$ (*n*-hexane:EtOAc = 1:1) 0.47.

m.p. 194-199 °C.

^{1}H NMR (400 MHz, *DMSO-d$_6$*) δ = 8.59 (d, *J*=8.6, 1H), 8.39 (t, *J*=5.5, 1H), 7.93 – 7.88 (m, 2H), 7.87 – 7.80 (m, 2H), 7.60 – 7.42 (m, 8H), 7.33 (dd, *J*=8.3, 6.8, 2H), 7.28 – 7.22 (m, 1H), 5.20 (d, *J*=6.4, 1H), 5.12 (dd, *J*=8.6, 5.3, 1H), 4.20 – 4.00 (m, 1H), 3.39 – 3.34 (m, 1H), 3.26 – 3.15 (m, 1H).

^{13}C NMR (101 MHz, *DMSO-d$_6$*) δ = 166.5, 166.3, 141.4, 134.6, 134.6, 131.3, 131.1, 128.3, 128.1, 127.5, 127.4, 127.2, 126.8, 71.7, 56.3, 43.6.

IR (ATR, ν in cm^{-1}): 3316 (m), 3030 (w), 2870 (w), 1635 (s), 1619 (m), 1577 (m), 1525 (s), 1487 (m),1440 (w), 1357 (w), 1316 (w), 1279 (m), 1147 (w), 1108 (s), 1091 (m), 1029 (w), 923 (w), 802 (w), 693 (s).

MS (ESI) m/z calcd for $C_{23}H_{22}N_2O_3Na$ 397.2 [M+Na]$^+$, found 397.4 [M+Na]$^+$.

HRMS (EI) m/z calcd for $C_{23}H_{20}N_2O_2$ 356.1525 [M-H$_2$O], found 356.1524 [M-H$_2$O].

2.9.2 1,3-Diamino-2-ol 9b

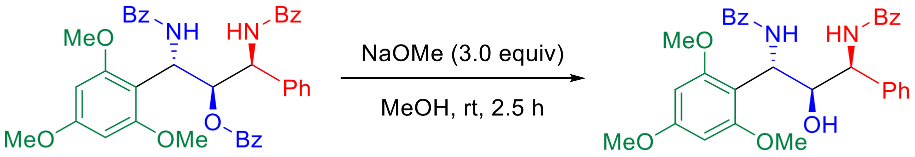

To a solution of 1,3-diamin-2-ol **5a** (161 mg, 0.25 mmol, 1.0 equiv) in methanol (3.5 mL) was added at room temperature sodium methoxide (42 mg, 0.75 mmol, 3.0 equiv) in one portion. After stirring for 2.5 h the reaction mixture was quenched with saturated aqueous NH_4Cl (5 mL). The aqueous phase was extracted with dichloromethane (3x 5 mL) and the combined organic layers were dried over Na_2SO_4 before the solvent was evaporated under reduced pressure. Purification by flash chromatography via puriflash XS 420+ machine, HP_15µm_F0012 flash column (*n*-hexane:EtOAc = 90:10 → 20:80) afforded the deprotected 1,2-*syn*-2,3-*anti*-1,3-diamin-2-ol **9b** as a colorless foam (123 mg, 91%, isolated *dr* >98:2; *dr* of the crude mixture >98:2, as determined by ^{1}H NMR analysis of the crude product after aqueous workup).

R$_f$ (*n*-hexane:EtOAc = 1:1) 0.27.

m.p. 106-110 °C.

^{1}H NMR (400 MHz, *Chloroform-d*) δ = 8.47 (d, *J*=8.6, 1H), 7.71 – 7.66 (m, 2H), 7.65 – 7.60 (m, 2H), 7.52 – 7.27 (m, 8H), 7.26 – 7.14 (m, 4H), 6.24 (dd, *J*=8.7, 4.7, 1H), 6.19 (s, 2H), 5.30 (d, *J*=8.0, 1H), 4.31 (dd, *J*=4.7, 1.4, 1H), 3.88 (s, 6H), 3.81 (s, 3H), 2.71 (bs, 1H).

^{13}C NMR (101 MHz, *Chloroform-d*) δ = 168.2, 166.3, 161.2, 159.0, 141.9, 134.5, 134.0, 131.6, 131.2, 128.6, 128.4, 128.4, 127.1, 127.0, 127.0, 126.8, 105.1, 91.2, 77.8, 56.2, 55.4, 54.1, 50.8.

IR (ATR, ν in cm^{-1}): 3423 (w), 2939 (w), 1646 (m), 1605 (m), 1512 (s), 1482 (s), 1453 (m), 1418 (m),1329 (w), 12247 (w), 1203 (m), 1148 (m), 1121 (s), 1057 (m), 1031 (m), 951 (w), 923 (w), 814 (w), 694 (s).

MS (ESI) m/z calcd for $C_{32}H_{32}N_2O_6Na$ 563.2 [M+Na]$^+$, found 563.5 [M+Na]$^+$.

HRMS (EI) m/z calcd for $C_{32}H_{30}N_2O_5$ 522.2155 [M-H_2O], found 522.2155 [M-H_2O].

3 NMR Data

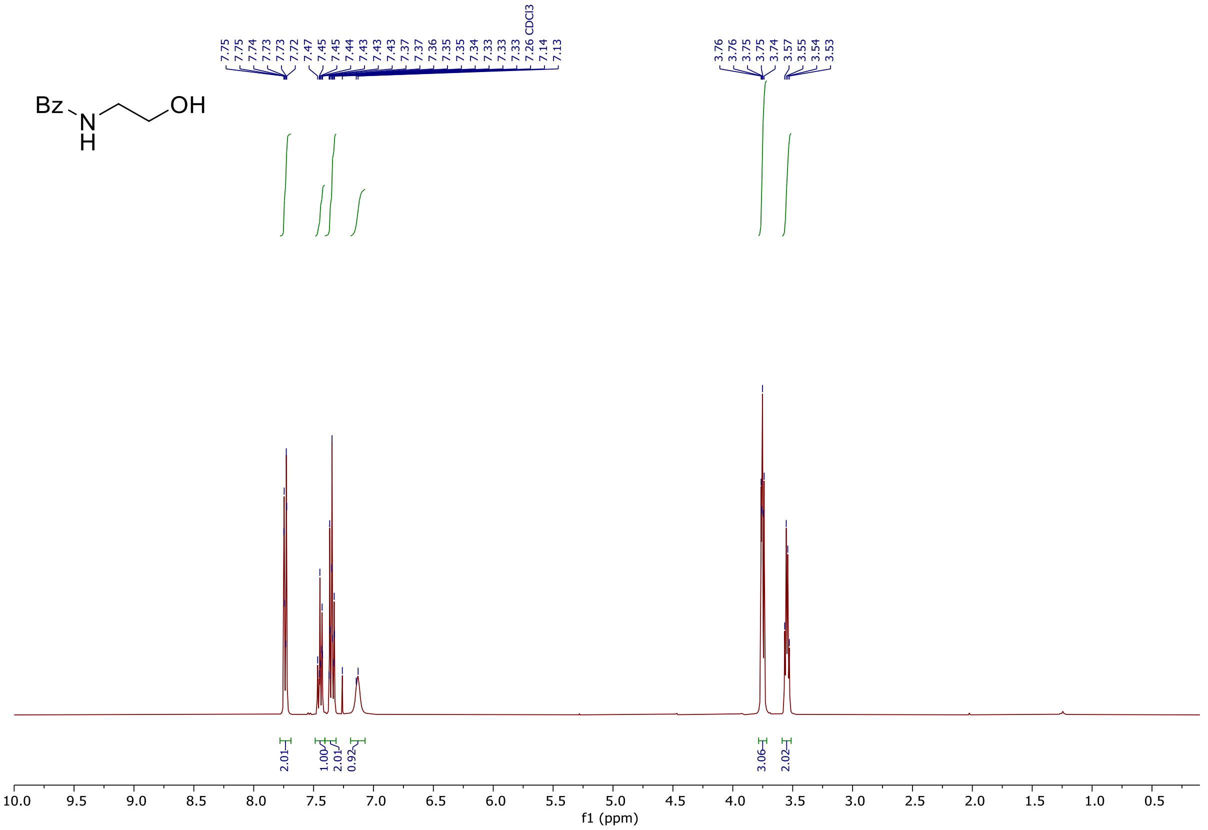

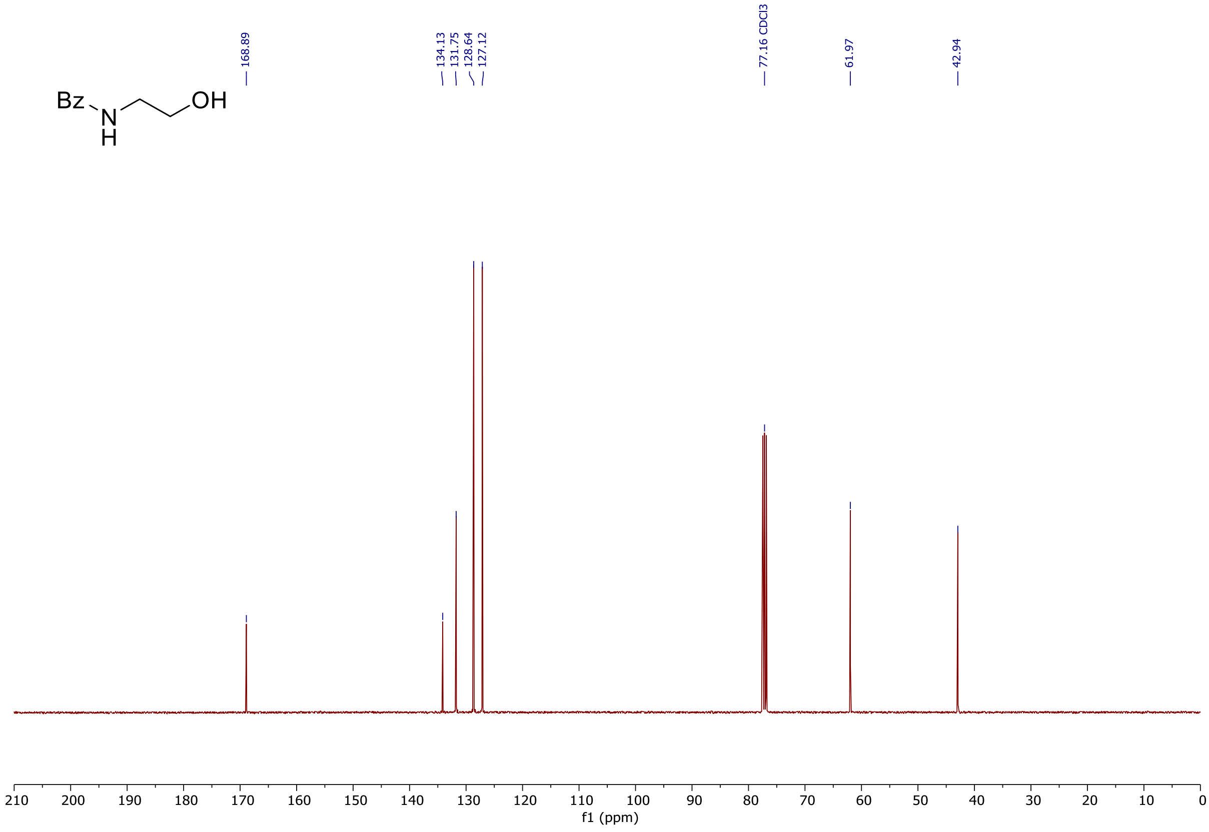

*Figure 1: ^{1}H (400 MHz) and ^{13}C (101 MHz) NMR spectra of **S1a** in $CDCl_3$.*

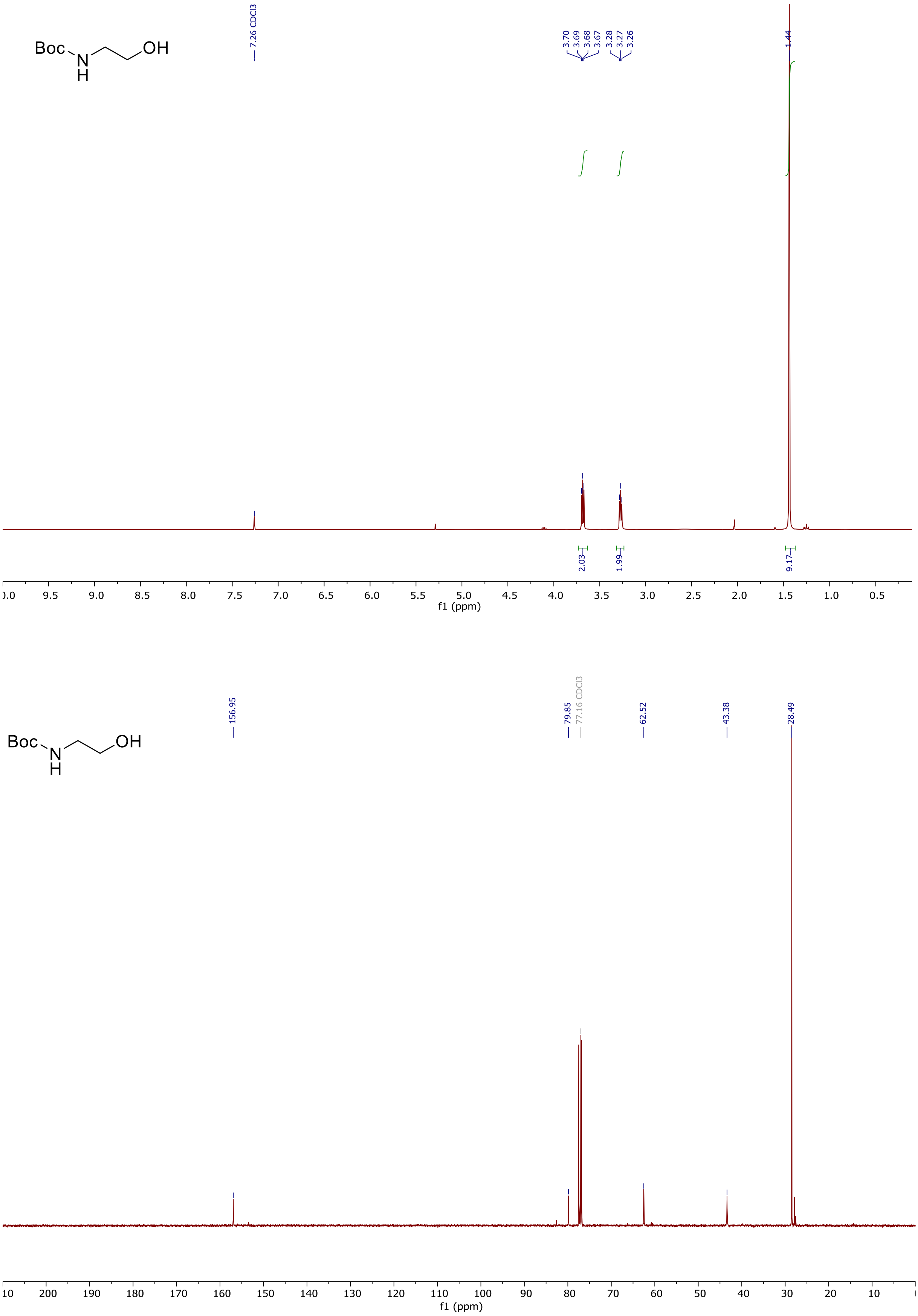

*Figure 2: ^{1}H (400 MHz) and ^{13}C (101 MHz) NMR spectra of **S1b** in $CDCl_3$.*

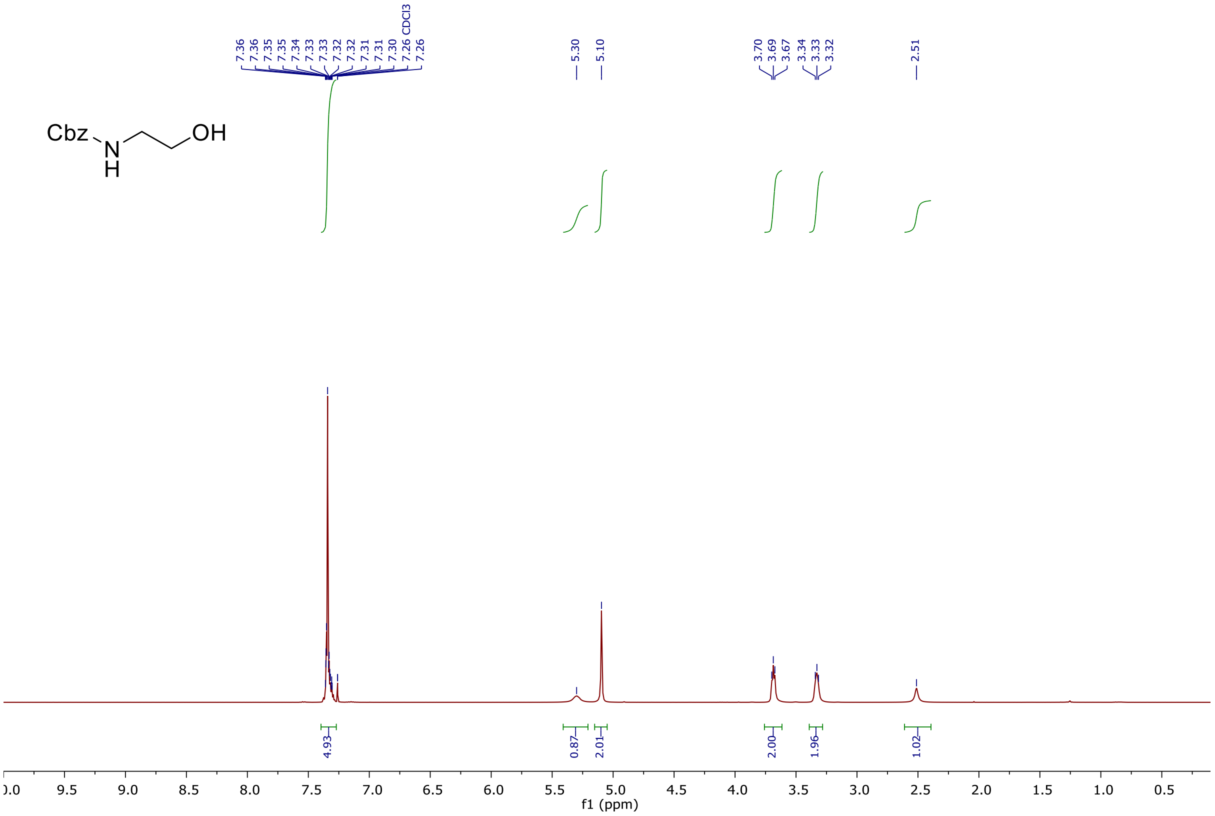

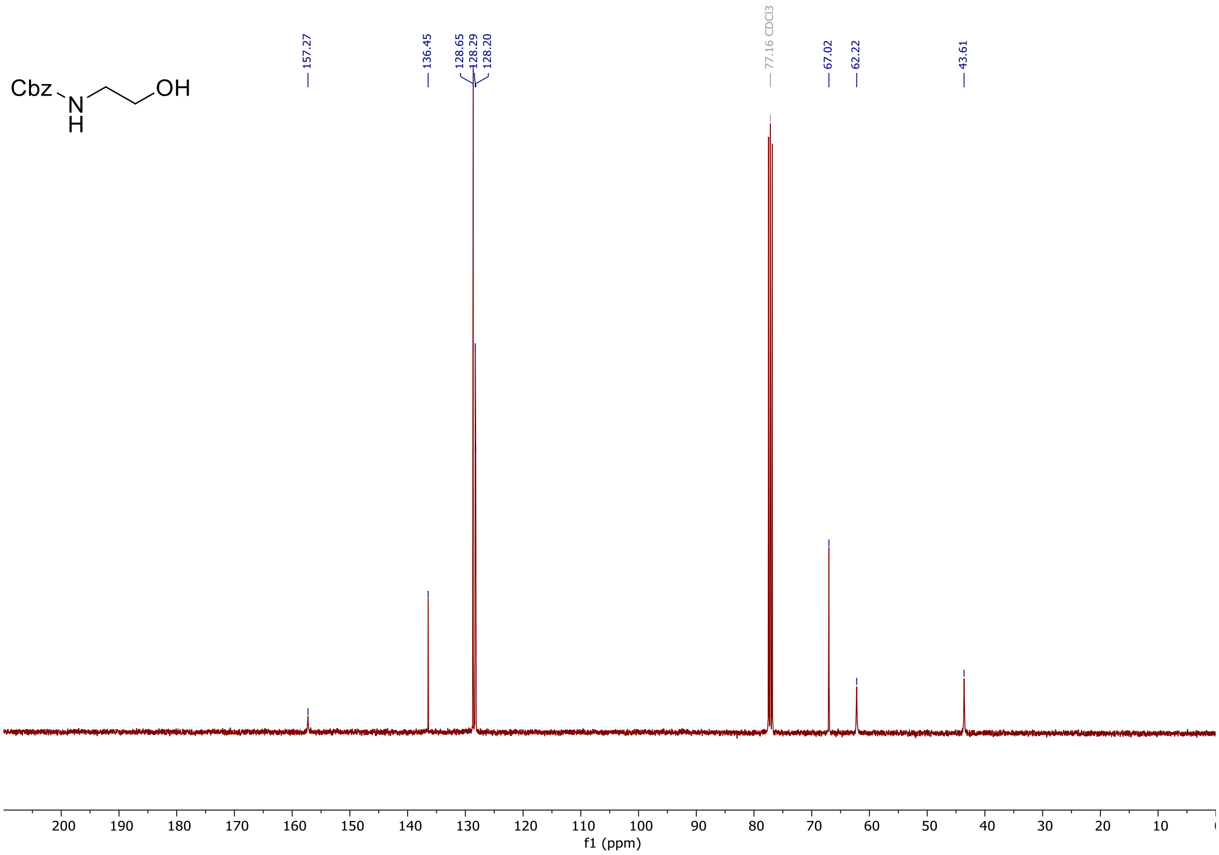

*Figure 3: ^{1}H (400 MHz) and ^{13}C (101 MHz) NMR spectra of **S1c** in $CDCl_3$.*

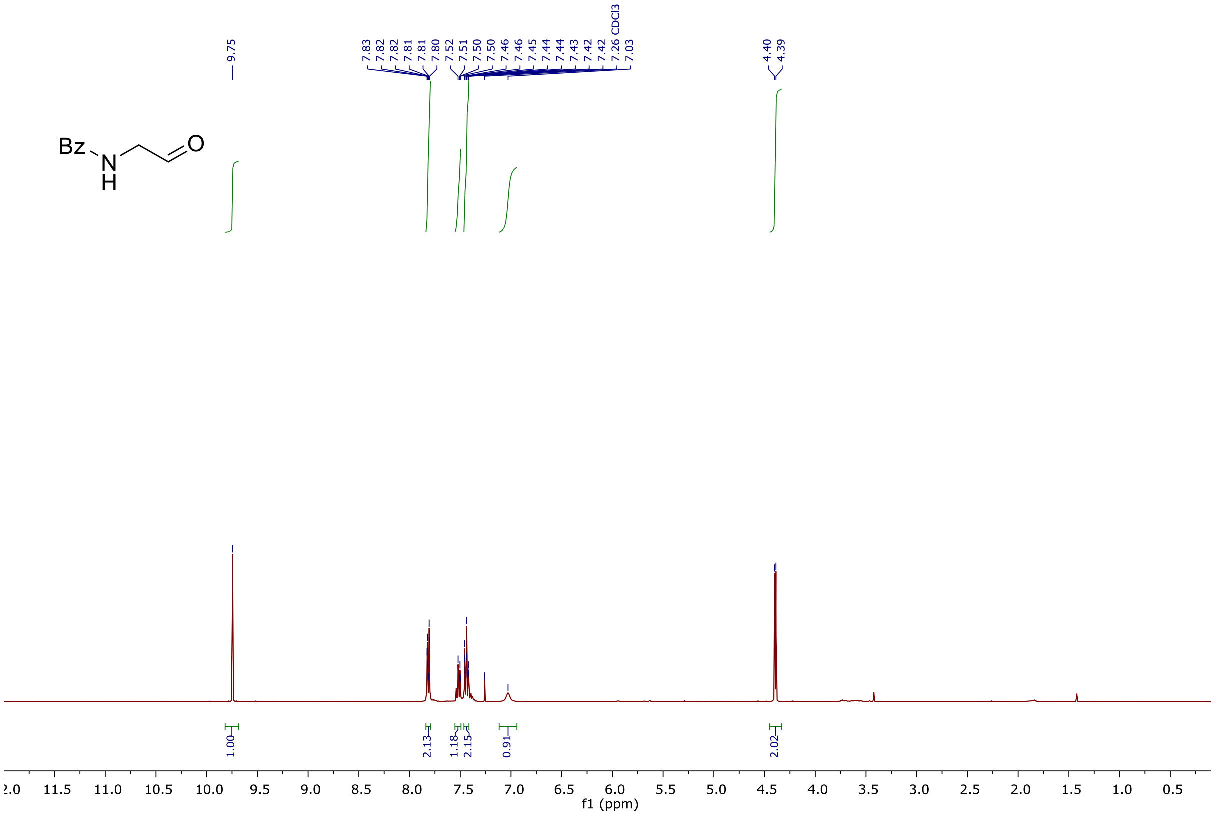

Figure 4: ¹H (400 MHz) NMR spectra of ***2a*** *in $CDCl_3$.*

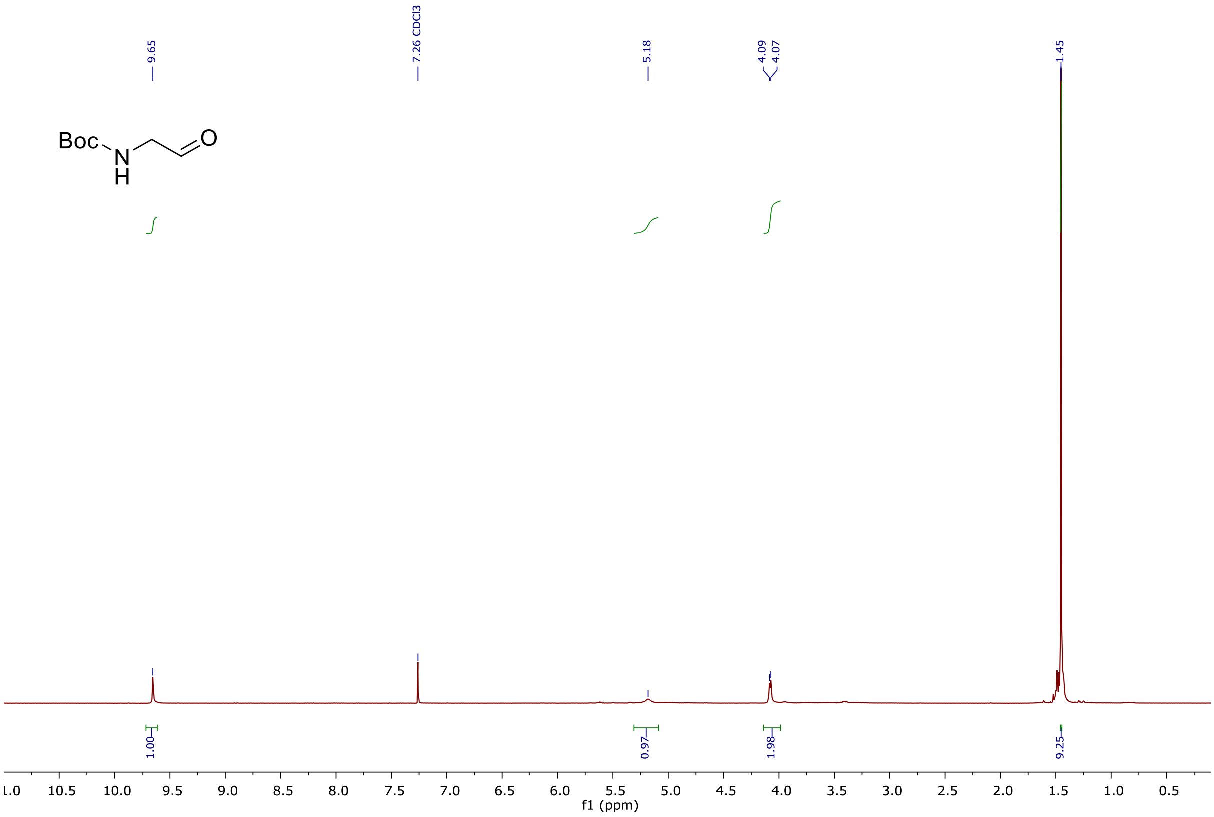

Figure 5: ¹H (400 MHz) NMR spectra of ***2b*** *in $CDCl_3$.*

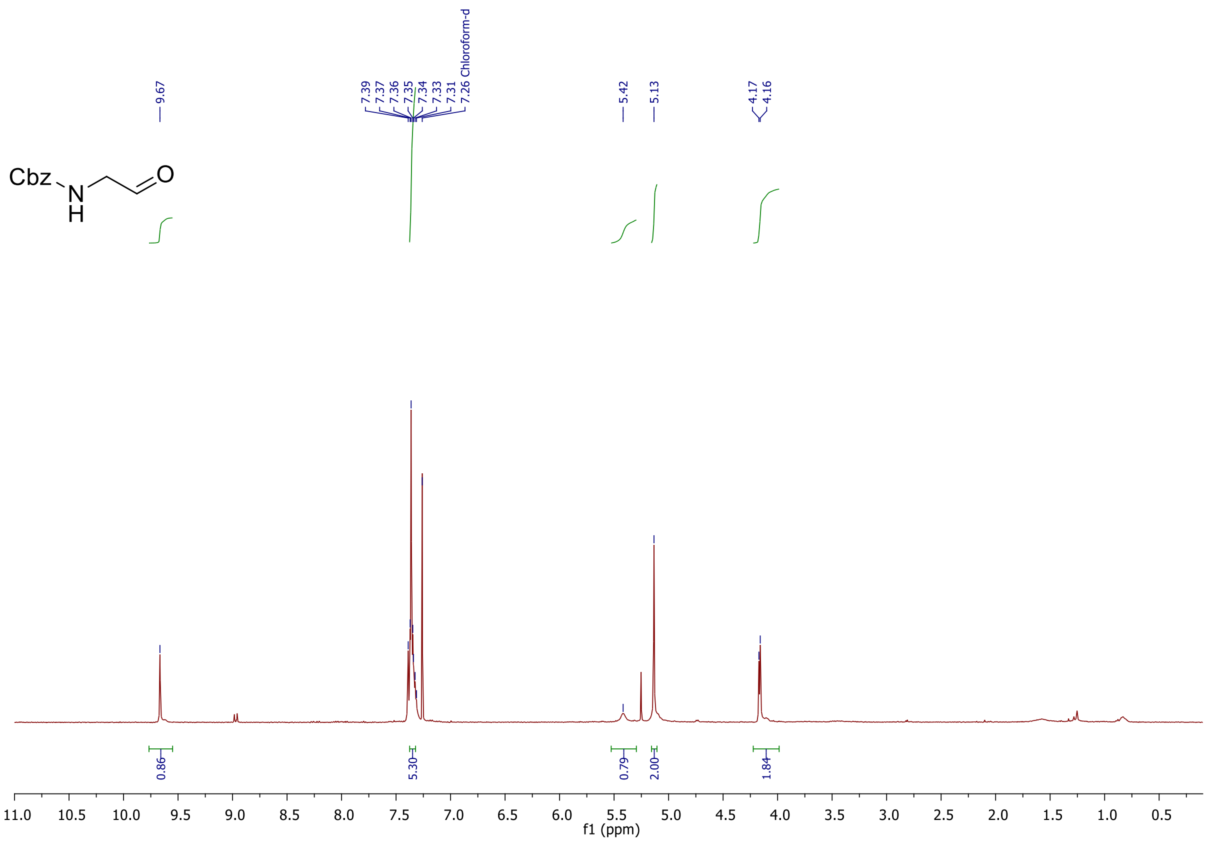

*Figure 6: ^{1}H (400 MHz) NMR spectra of **2c** in $CDCl_3$.*

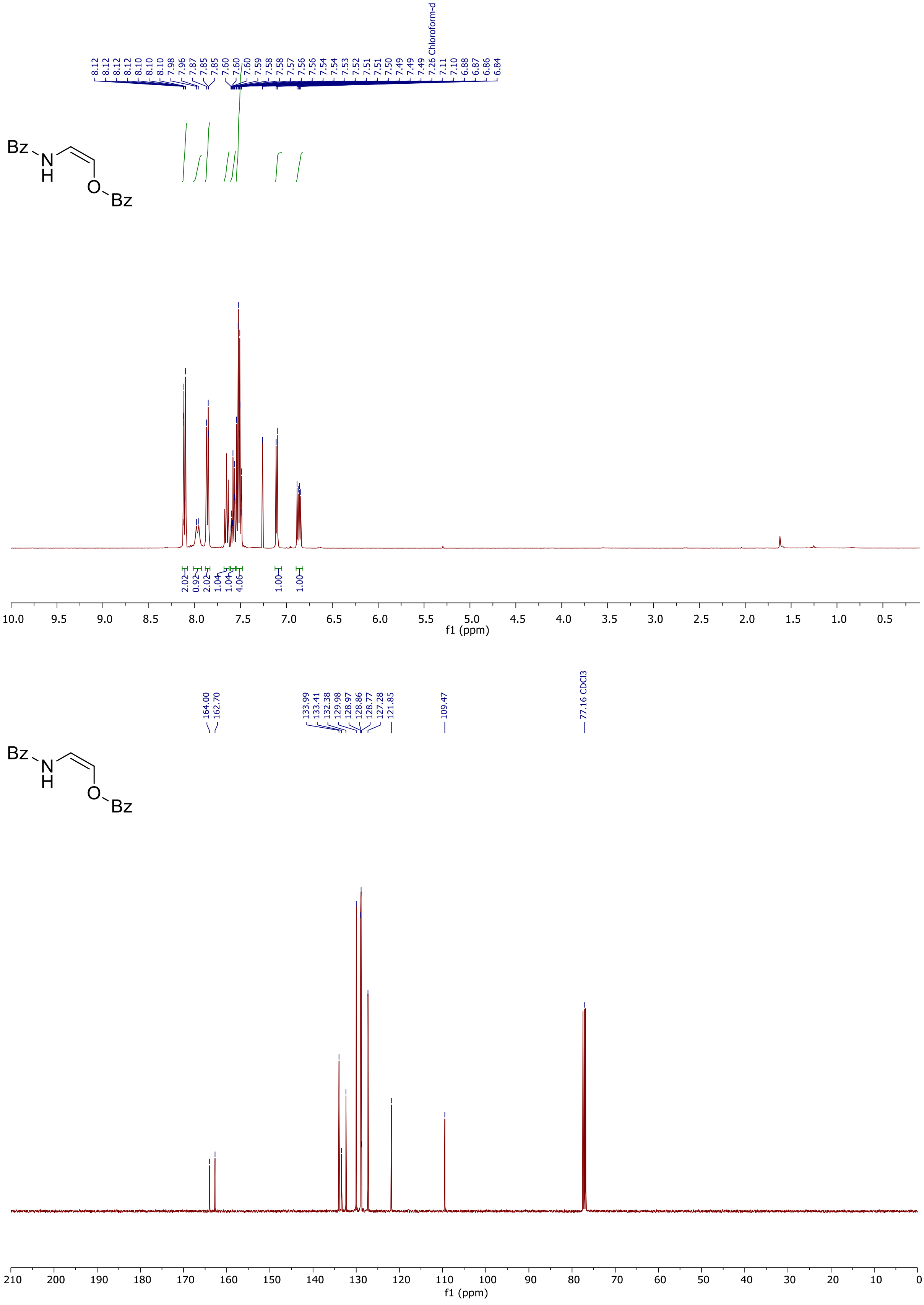

*Figure 7: ^{1}H (400 MHz) and ^{13}C (101 MHz) NMR spectra of **1a** in $CDCl_3$.*

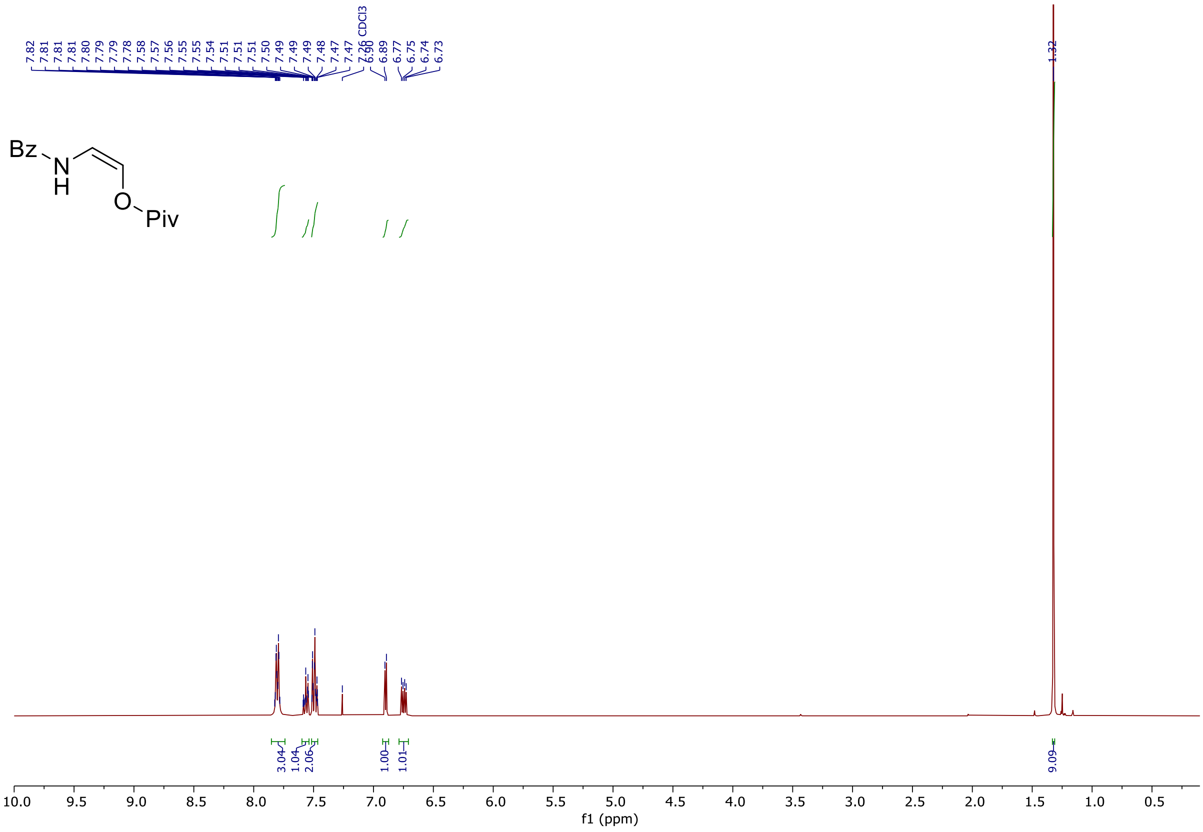

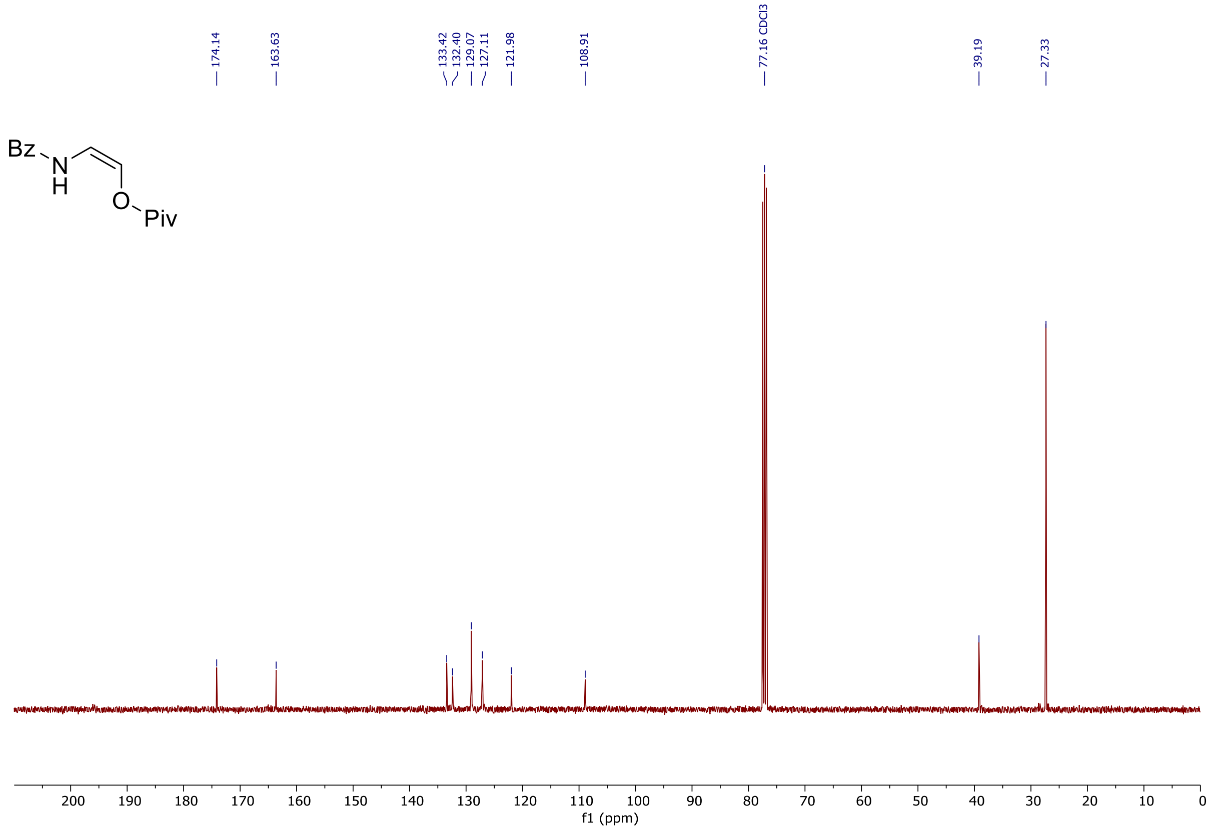

*Figure 8: ¹H (400 MHz) and ¹³C (101 MHz) NMR spectra of **1b** in CDCl₃.*

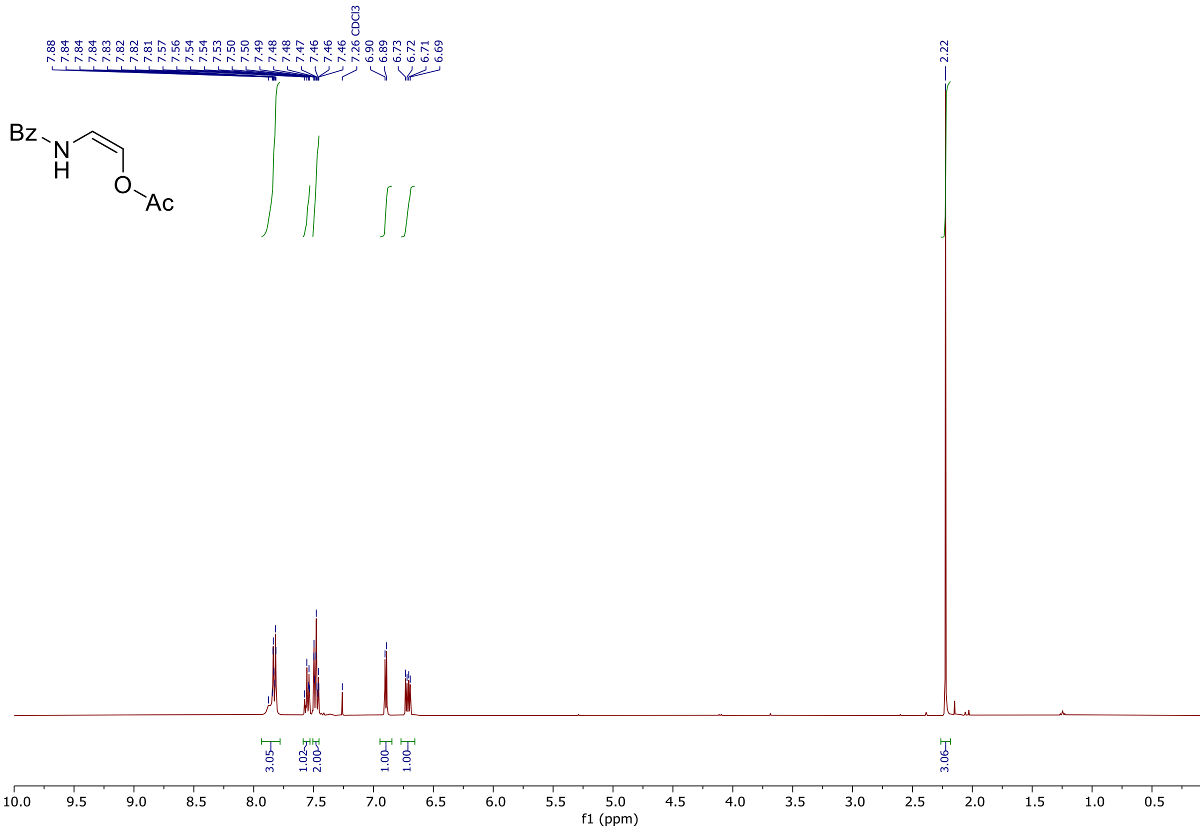

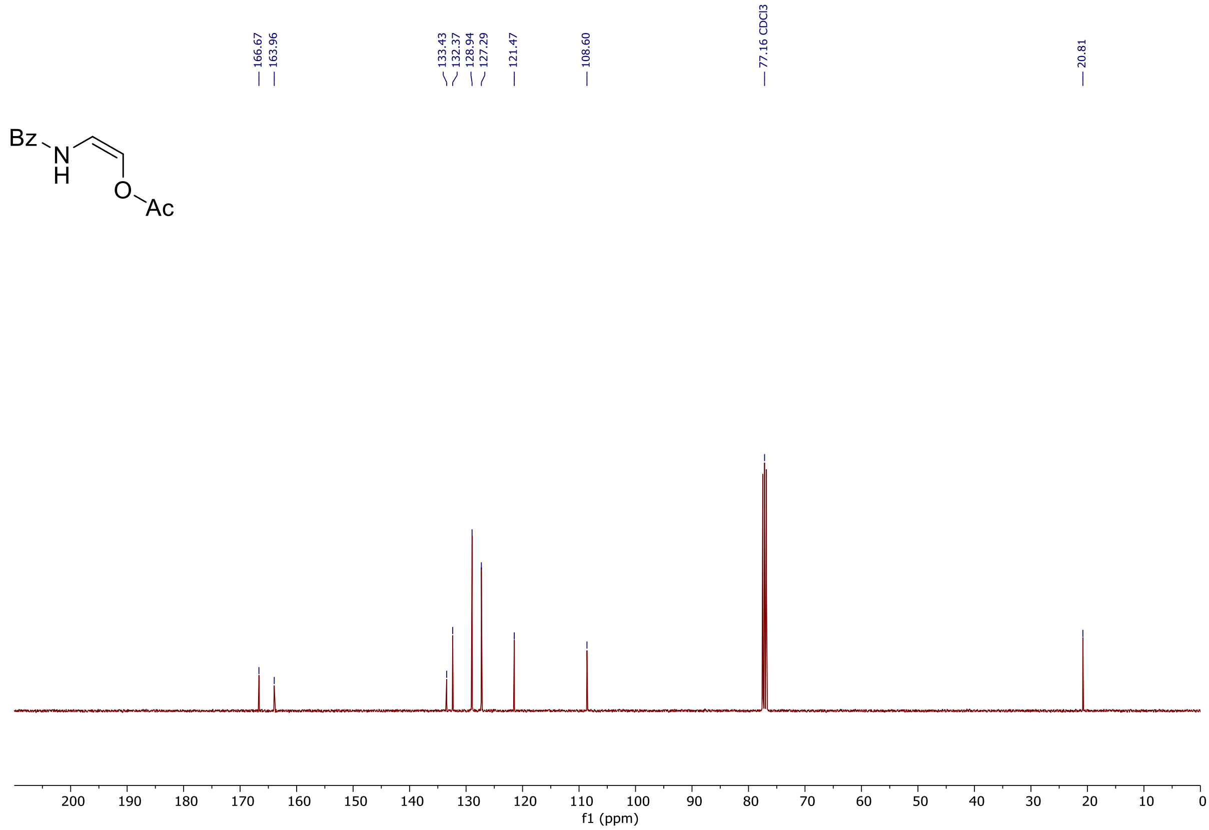

*Figure 9: ^{1}H (400 MHz) and ^{13}C (101 MHz) NMR spectra of **1c** in $CDCl_3$.*

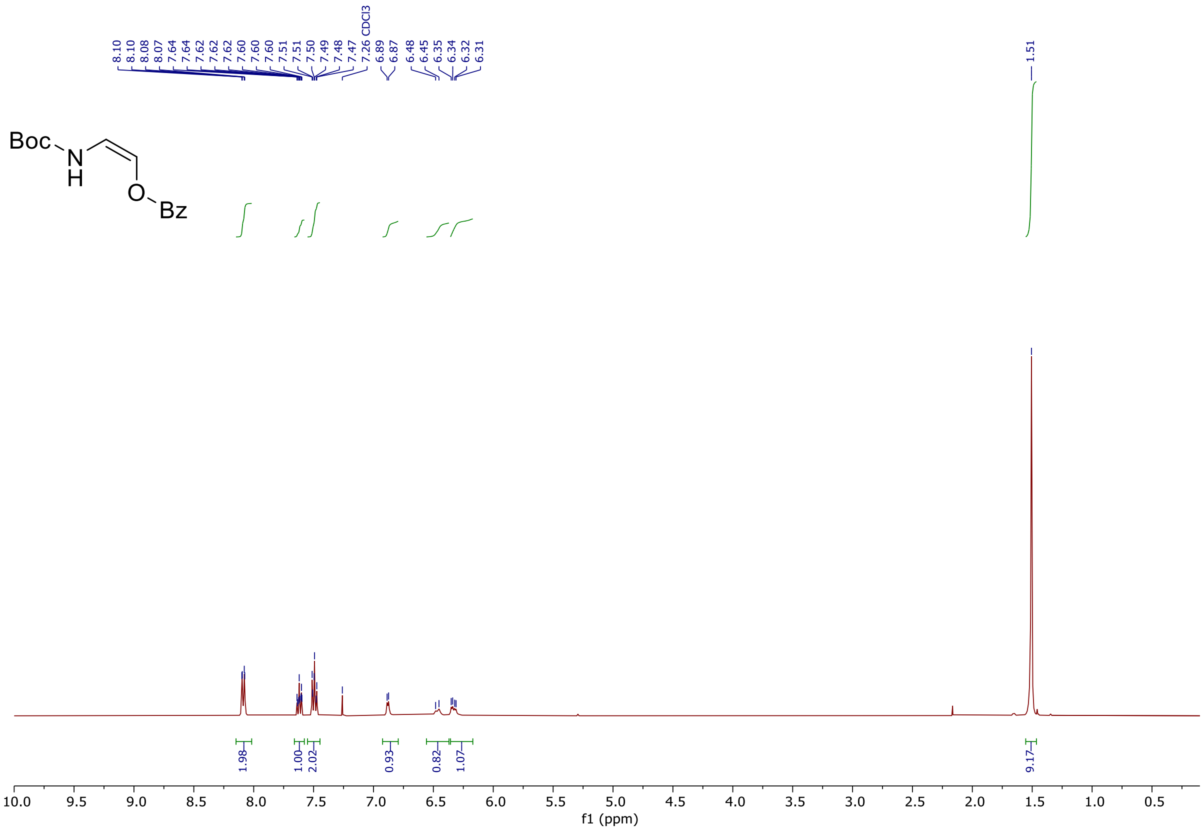

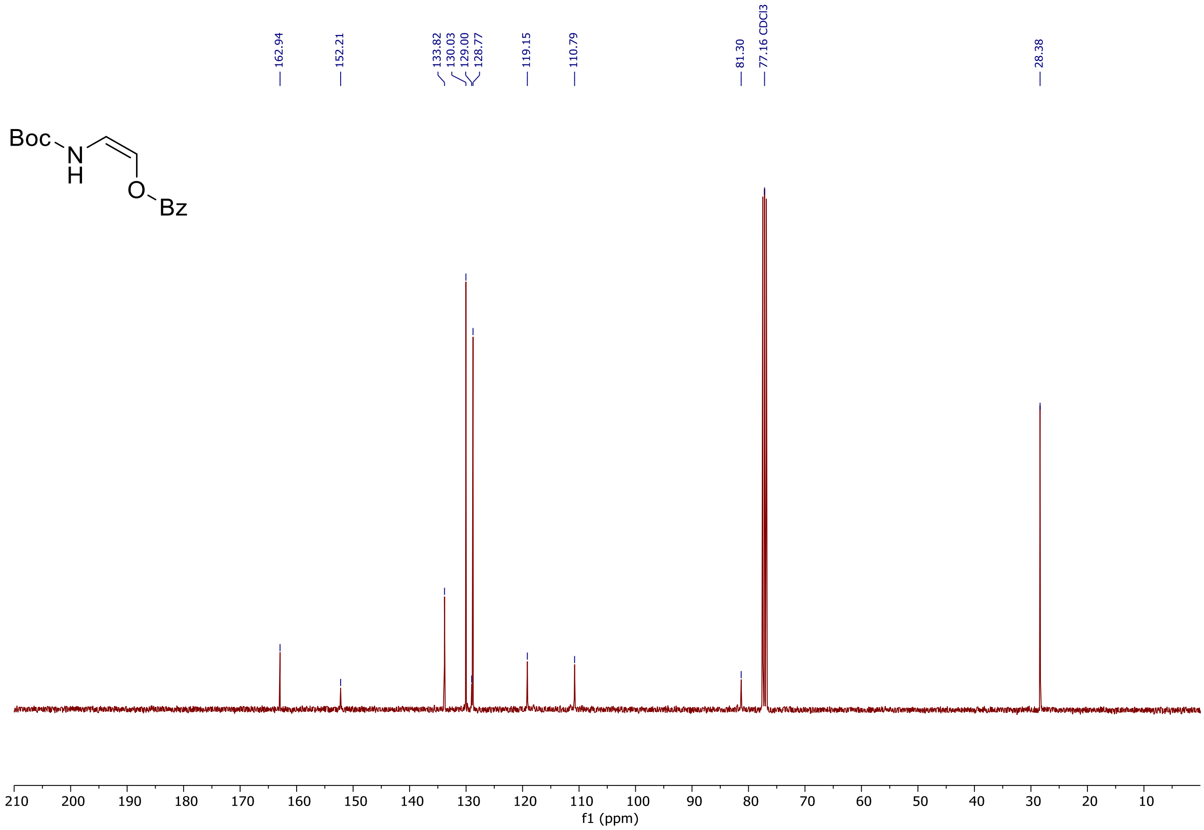

*Figure 10: ^{1}H (400 MHz) and ^{13}C (101 MHz) NMR spectra of **1d** in $CDCl_3$.*

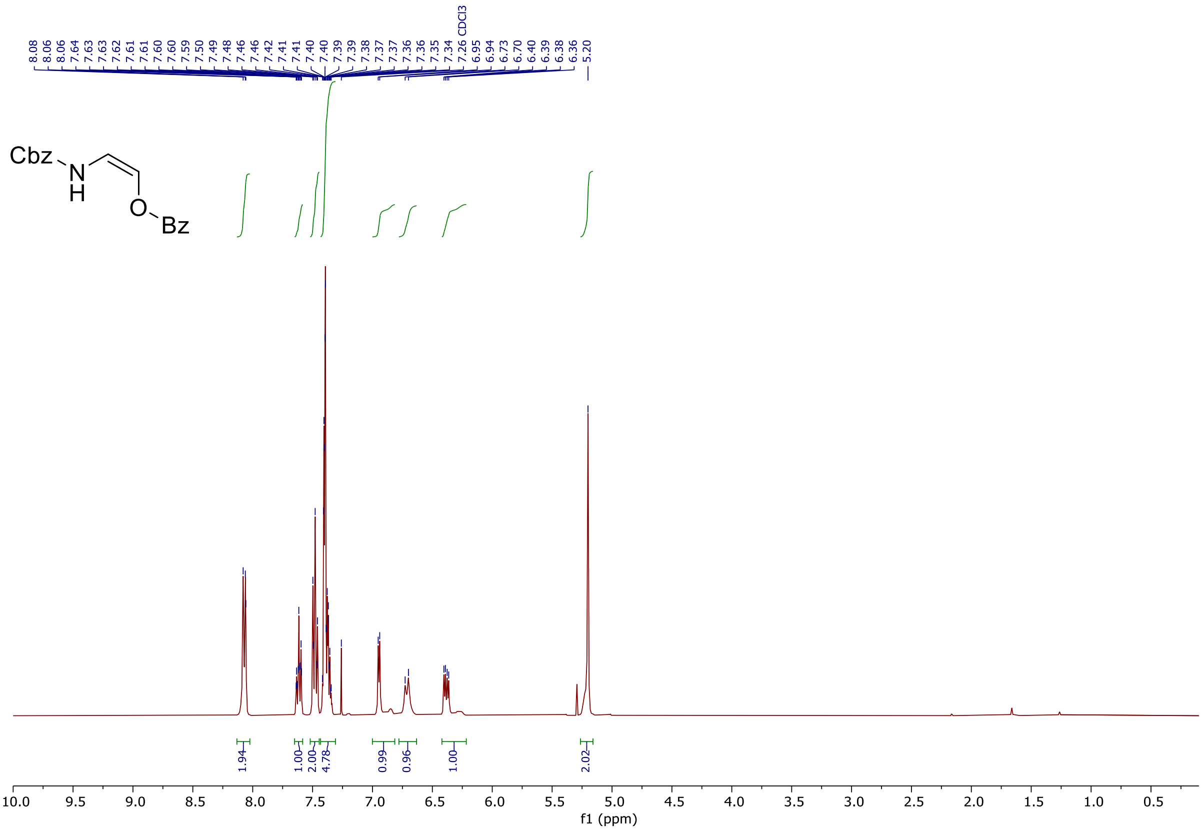

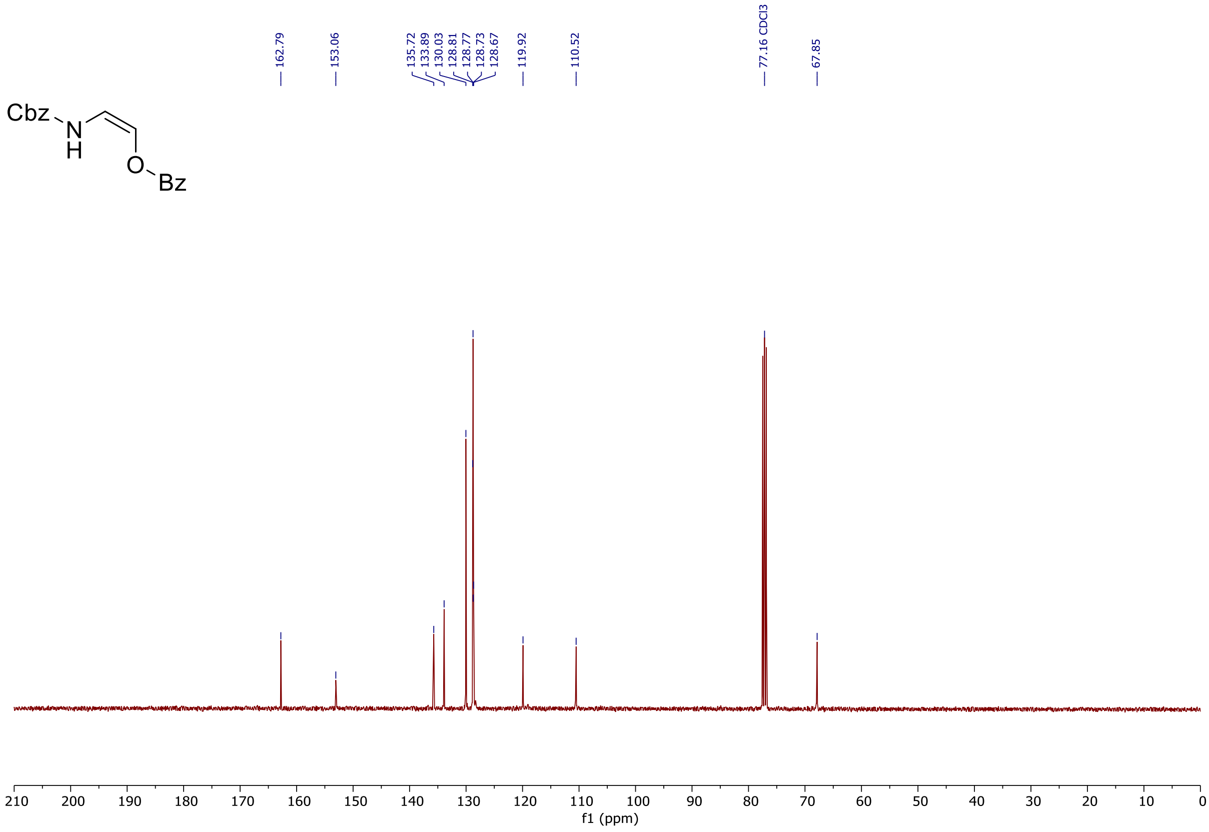

*Figure 11: ^{1}H (400 MHz) and ^{13}C (101 MHz) NMR spectra of **1e** in $CDCl_3$.*

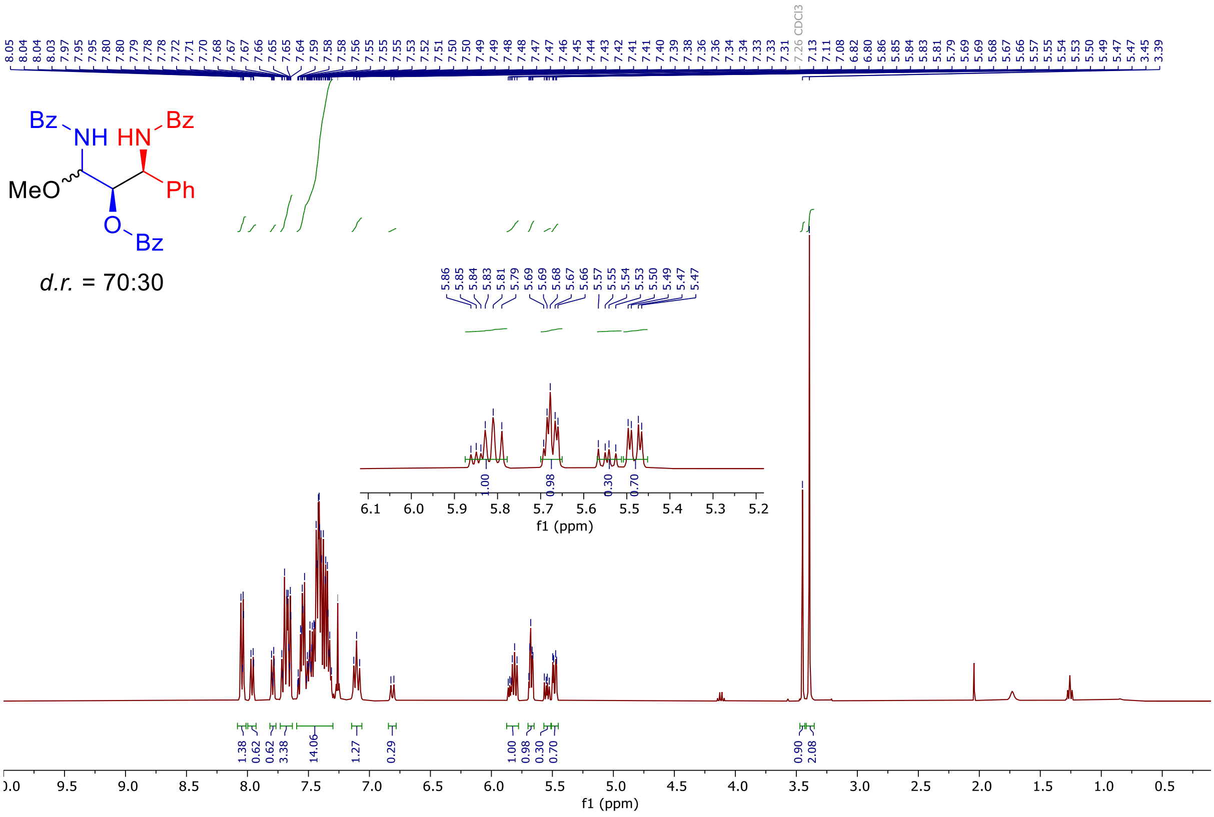

Figure 12: ^{1}H (400 MHz) NMR spectra of ***4a*** in $CDCl_3$.

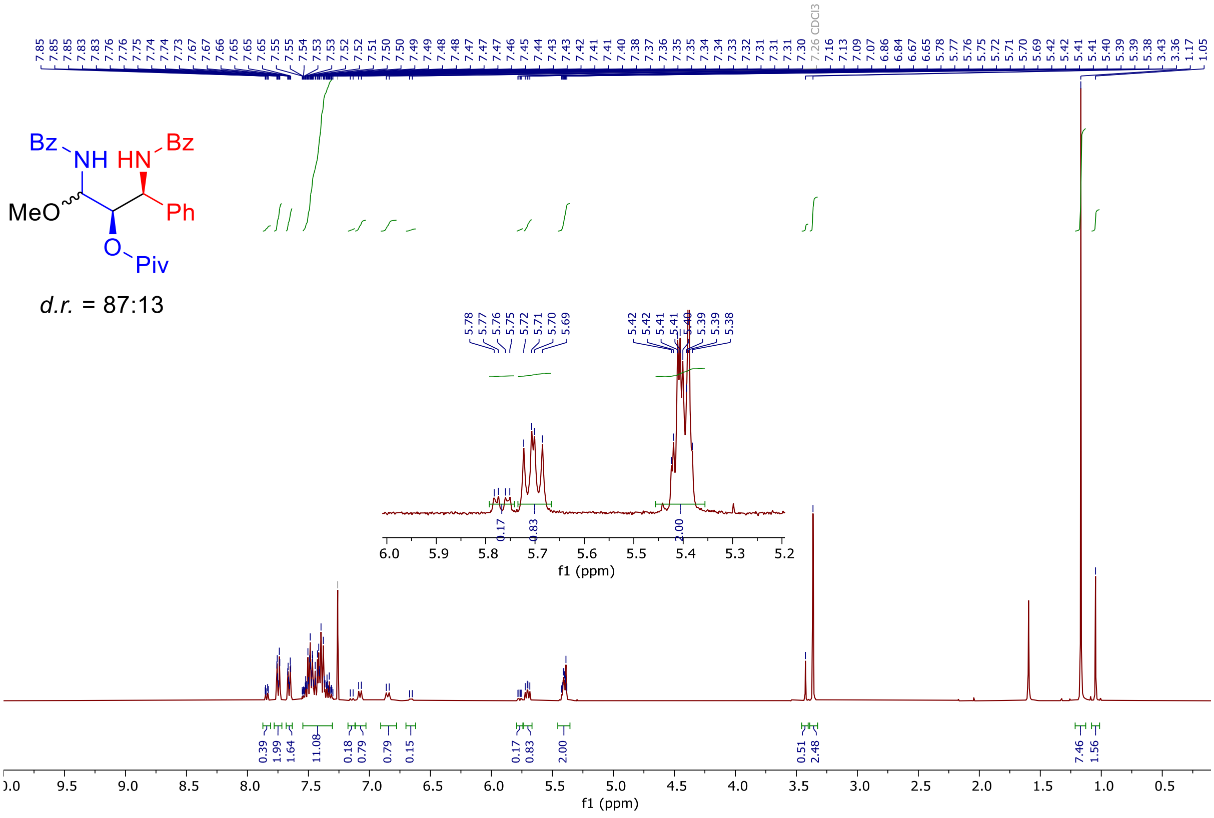

Figure 13: ^{1}H (400 MHz) NMR spectra of **4b** in $CDCl_3$.

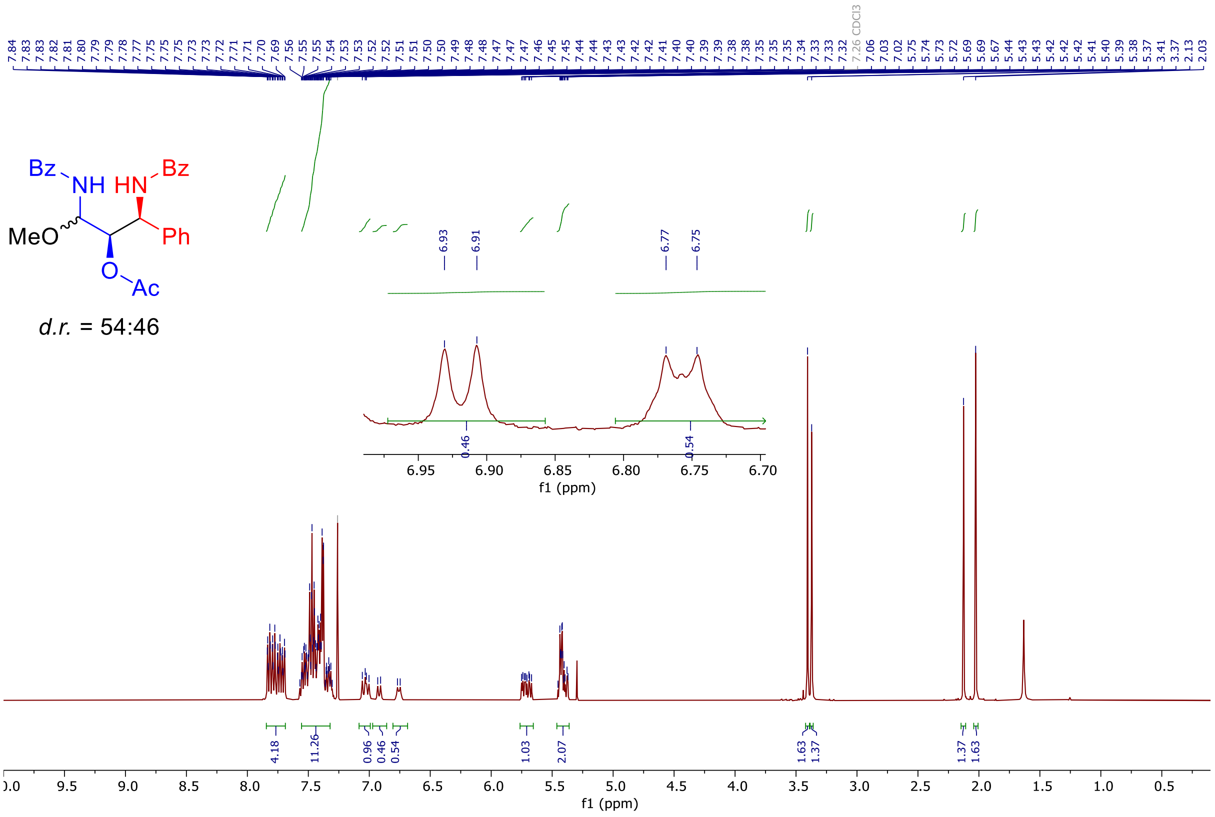

*Figure 14: ^{1}H (400 MHz) NMR spectra of **4c** in $CDCl_3$.*

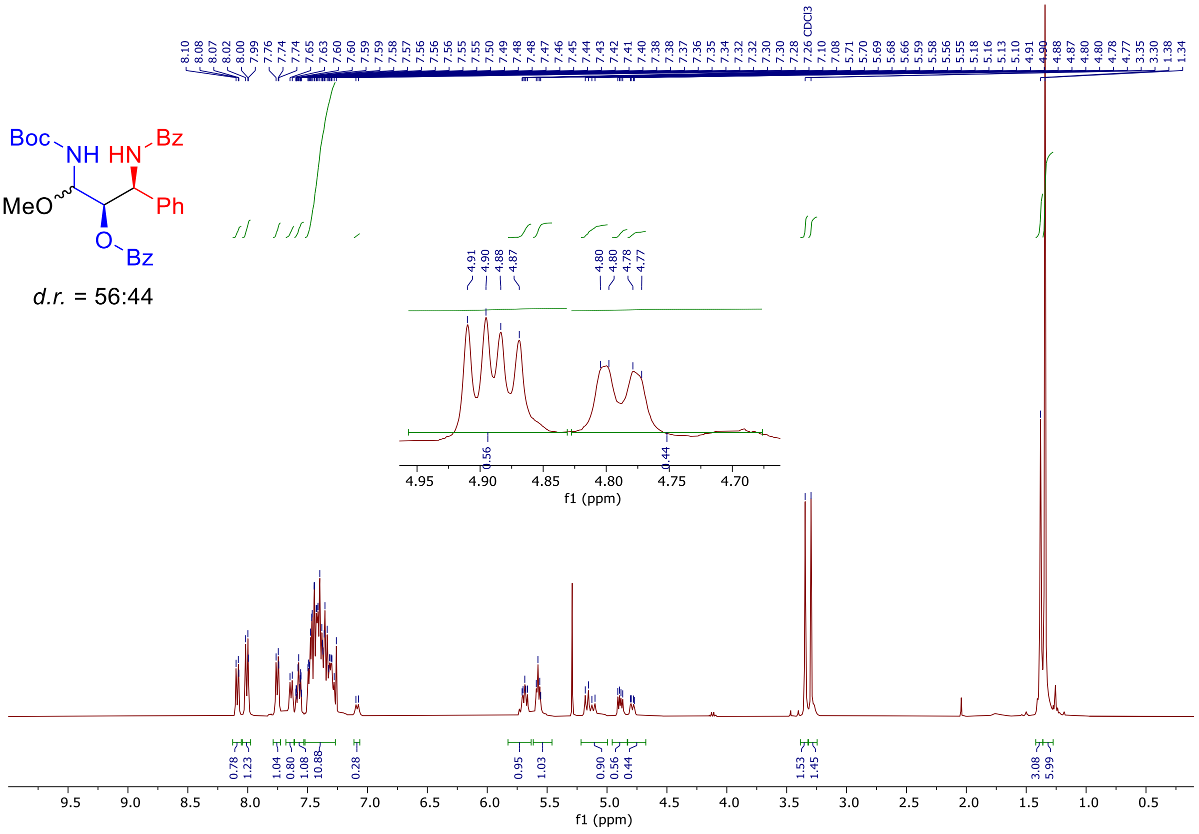

*Figure 15: ^{1}H (400 MHz) NMR spectra of **4d** in $CDCl_3$.*

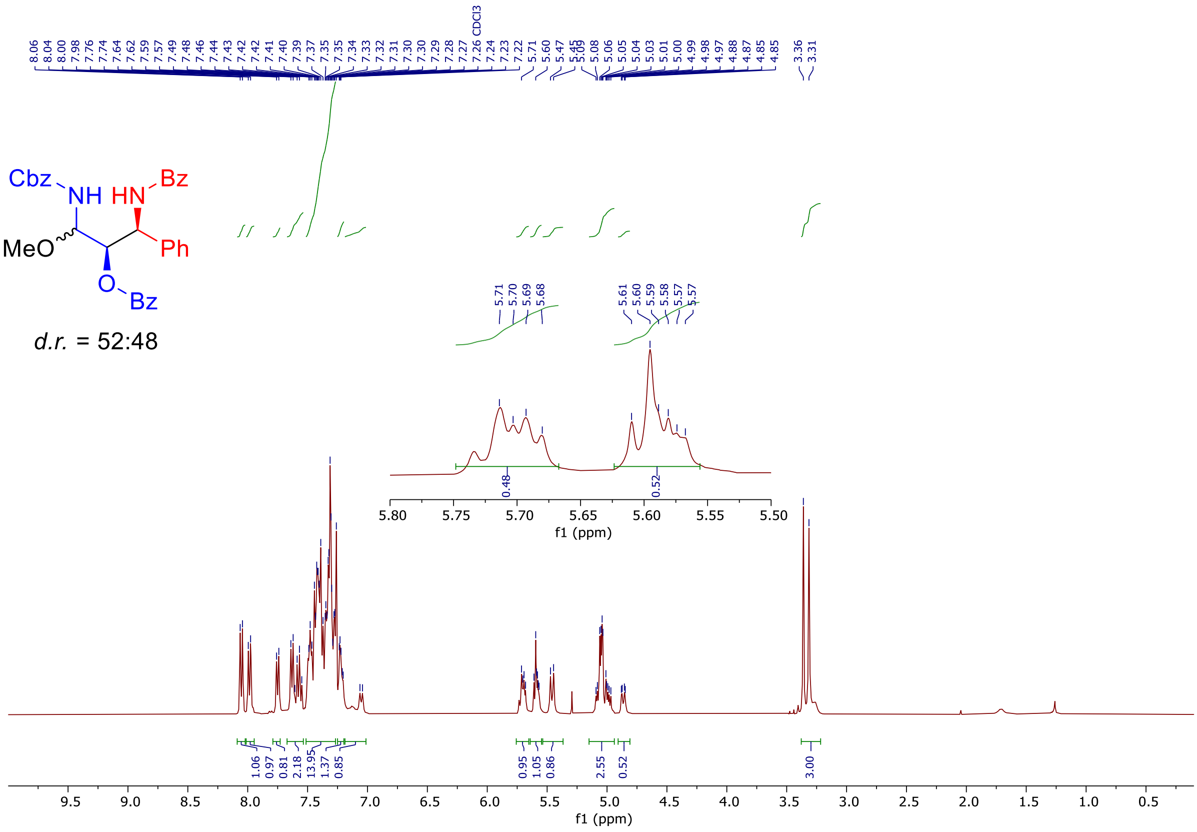

Figure 16: ^{1}H (400 MHz) NMR spectra of **4e** in $CDCl_3$.

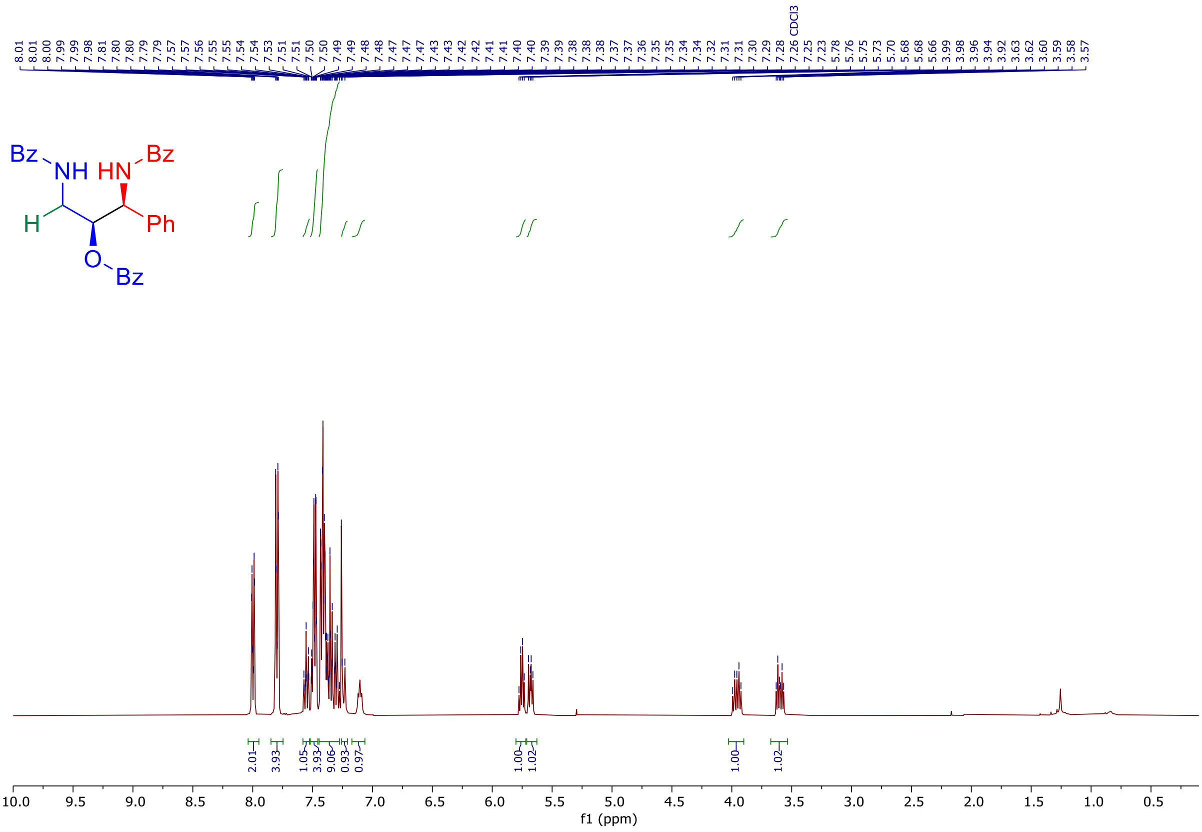

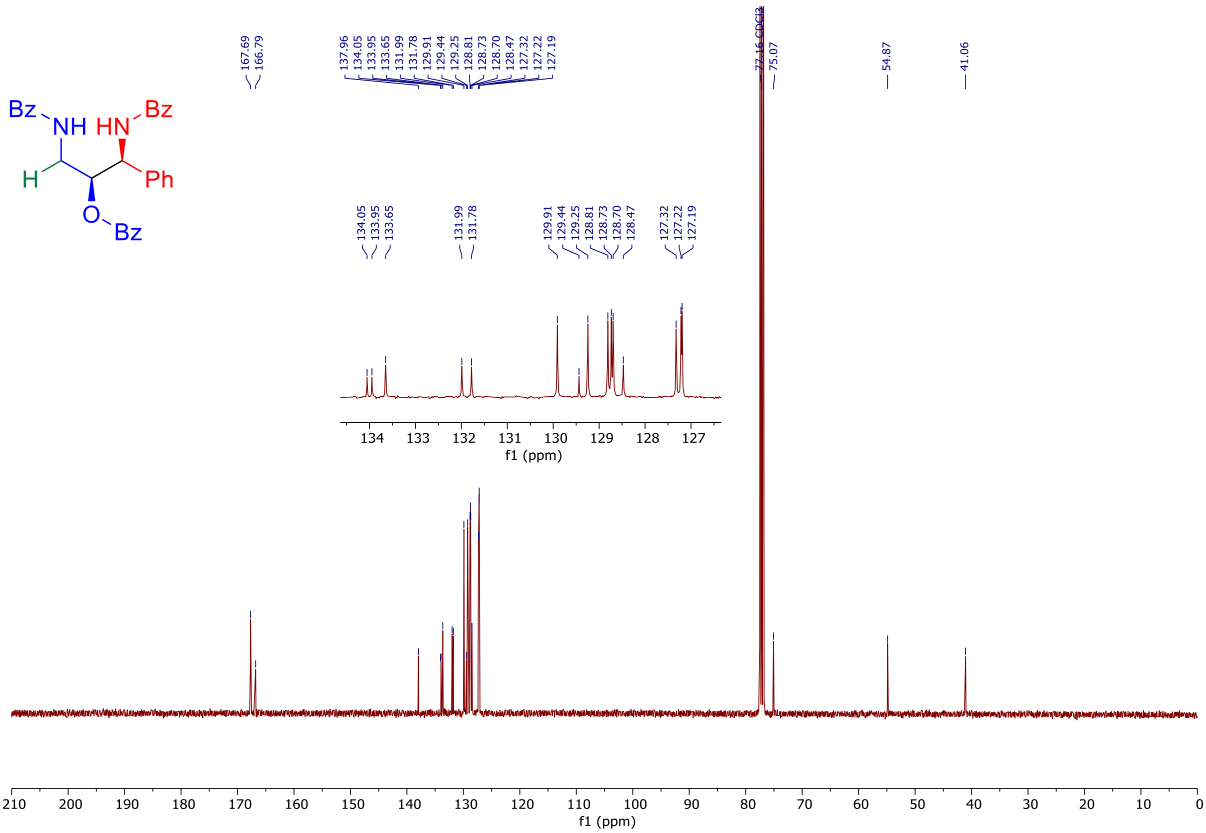

*Figure 17: ^{1}H (400 MHz) and ^{13}C (101 MHz) NMR spectra of **5a** in $CDCl_3$.*

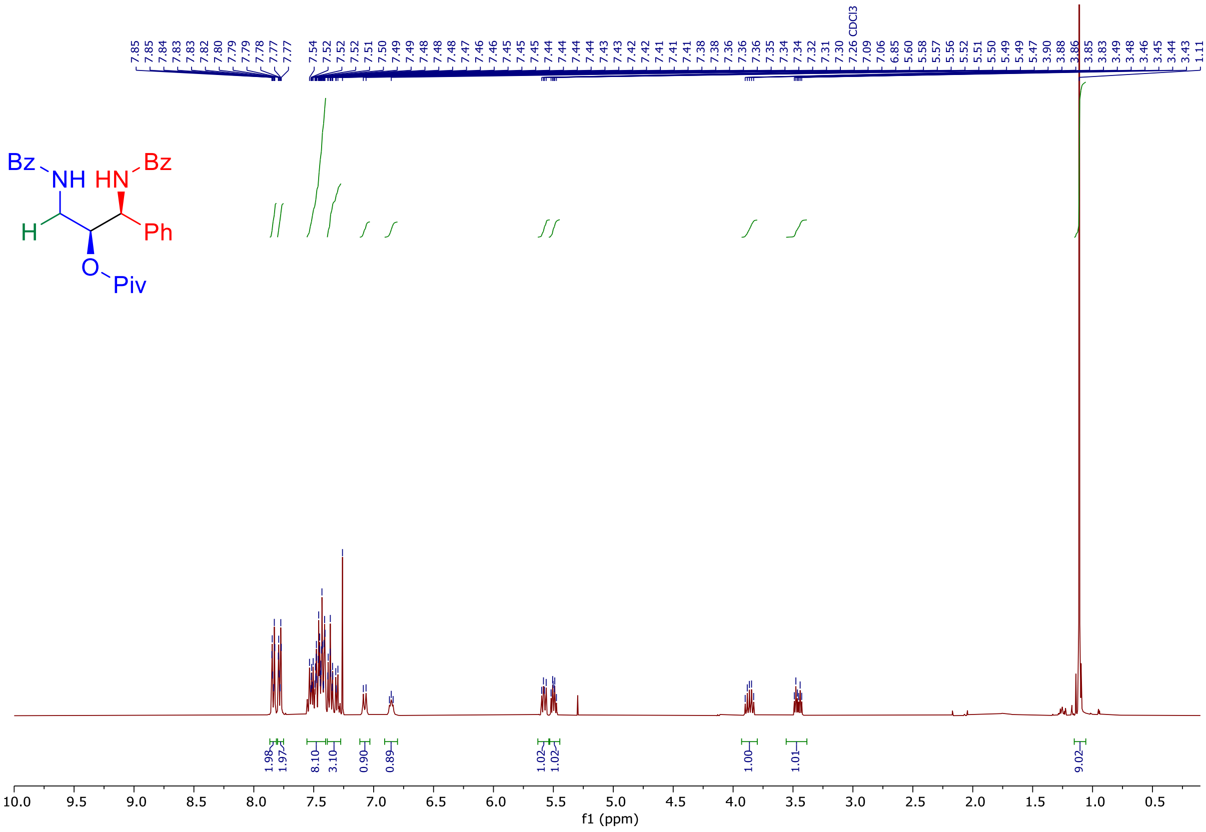

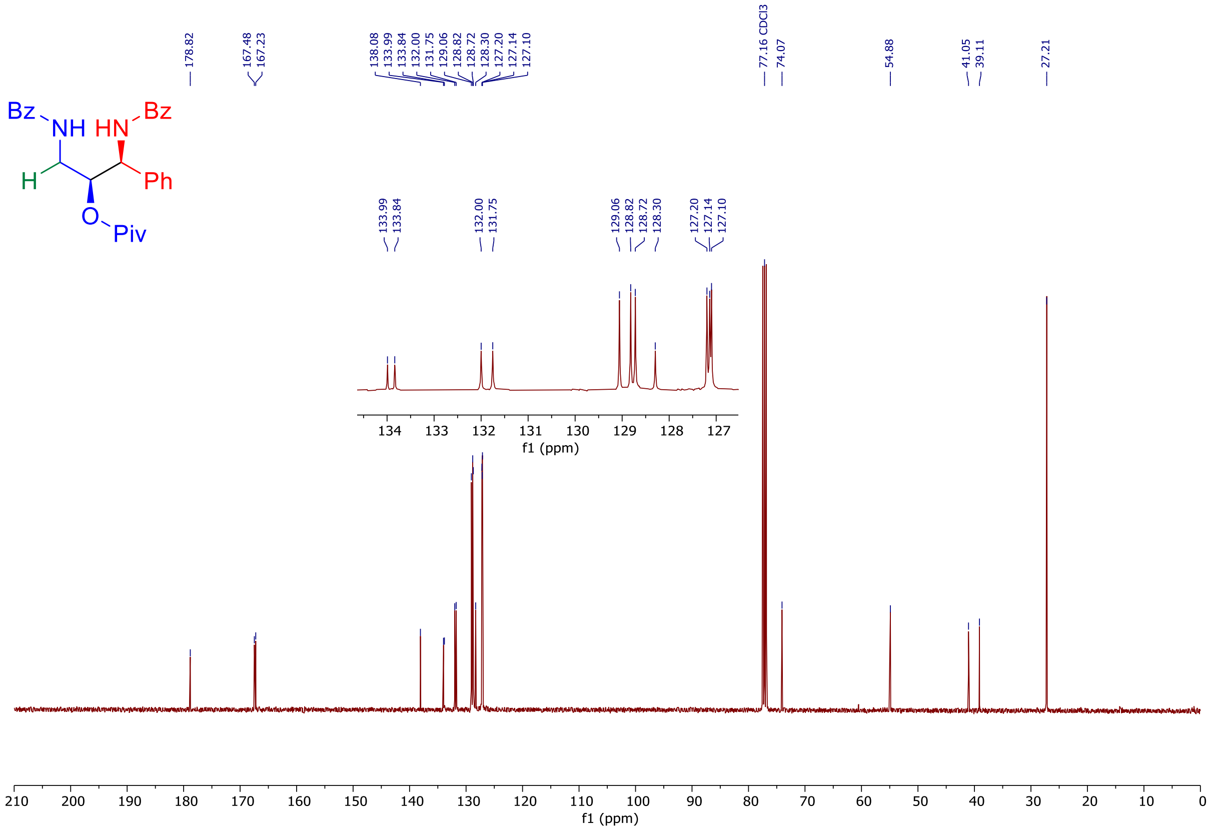

*Figure 18: ^{1}H (400 MHz) and ^{13}C (101 MHz) NMR spectra of **5b** in $CDCl_3$.*

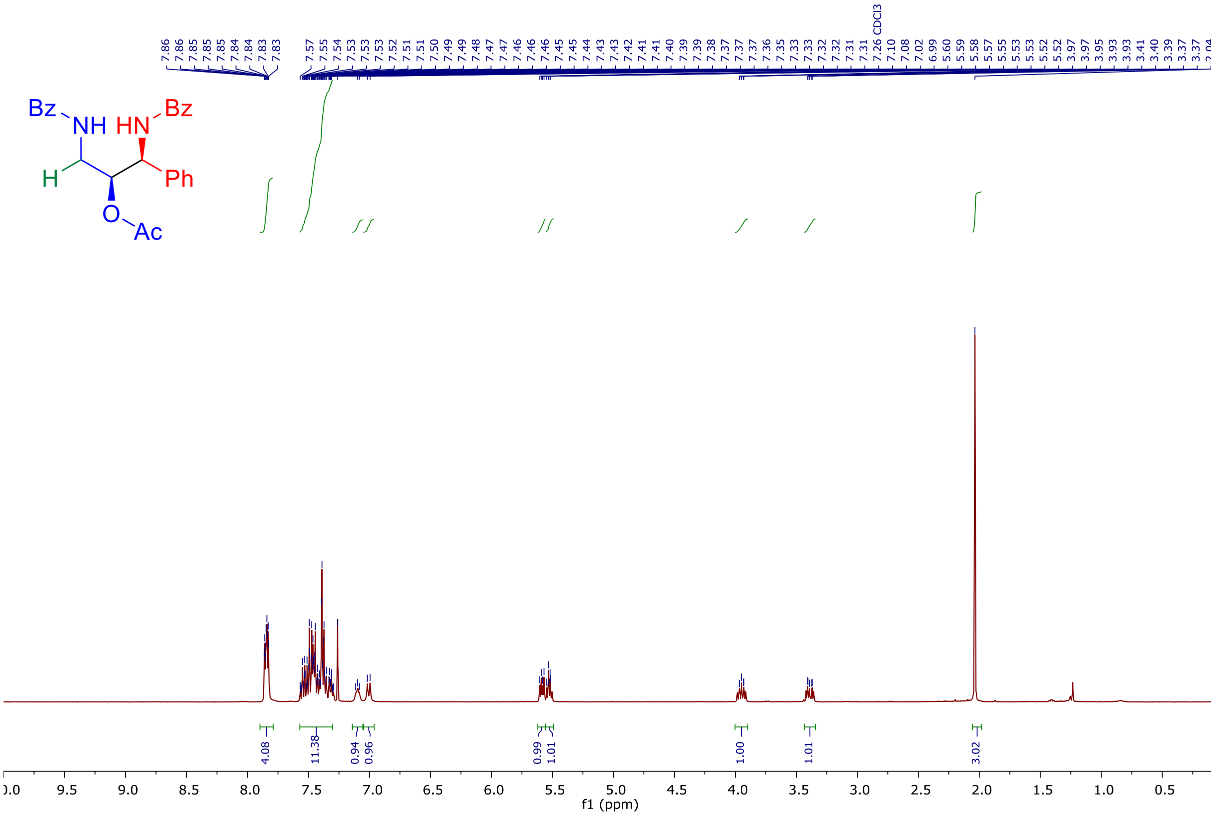

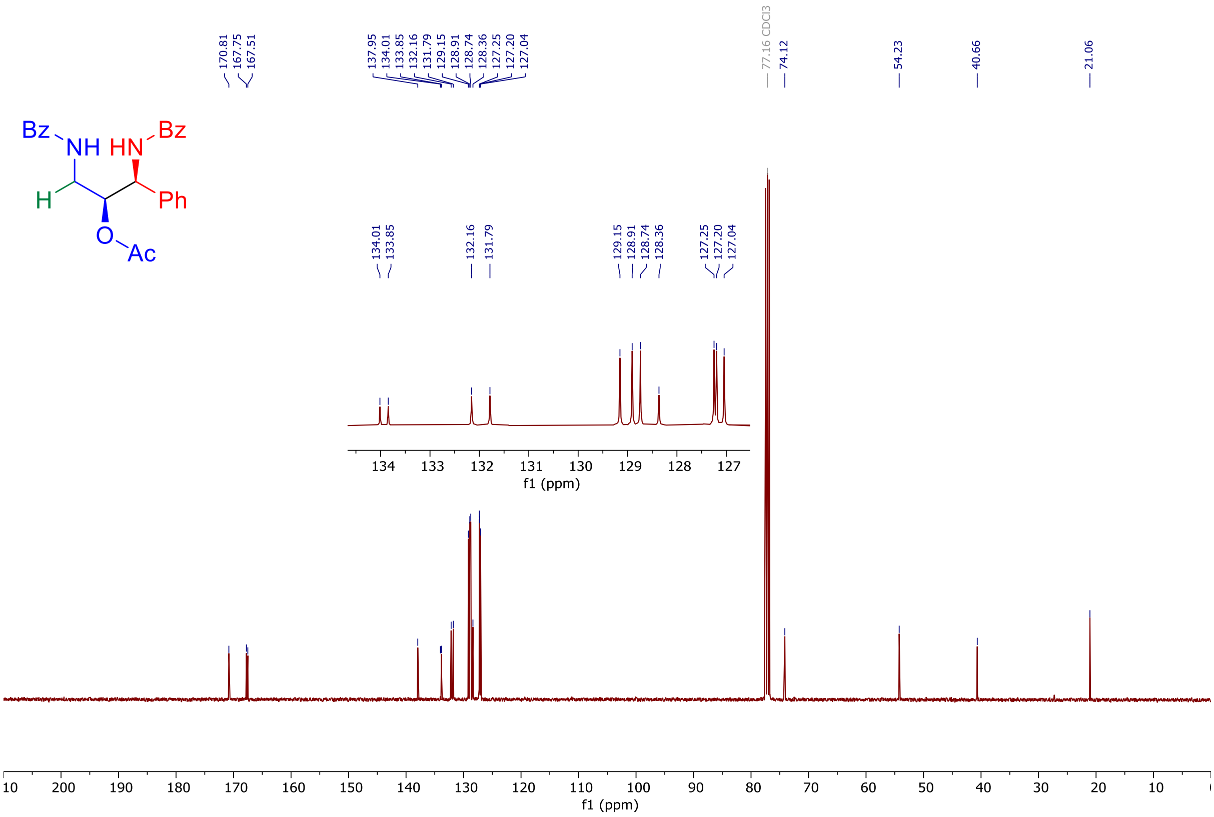

*Figure 19: ^{1}H (400 MHz) and ^{13}C (101 MHz) NMR spectra of **5c** in $CDCl_3$.*

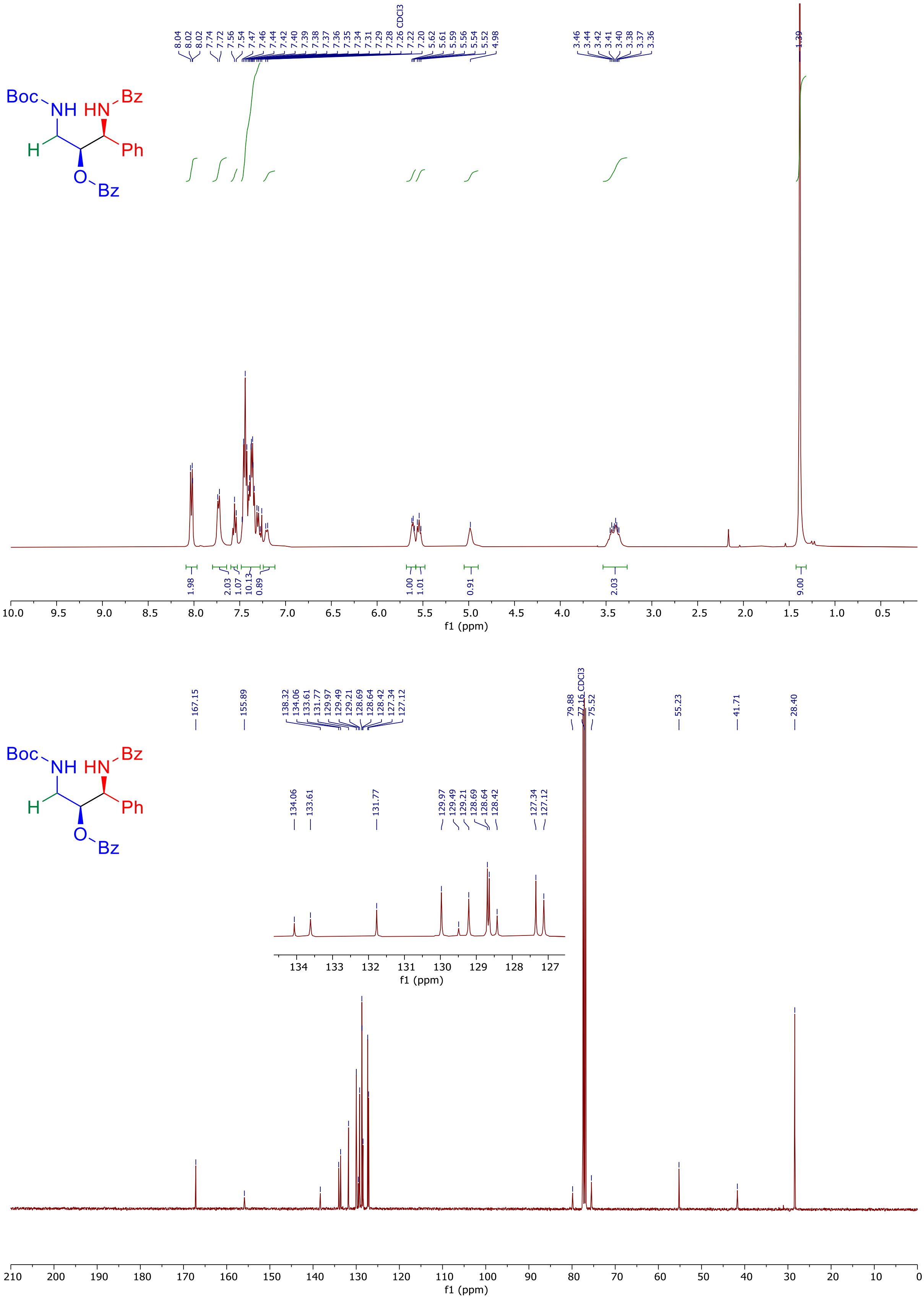

*Figure 20: ^{1}H (400 MHz) and ^{13}C (101 MHz) NMR spectra of **5d** in $CDCl_3$.*

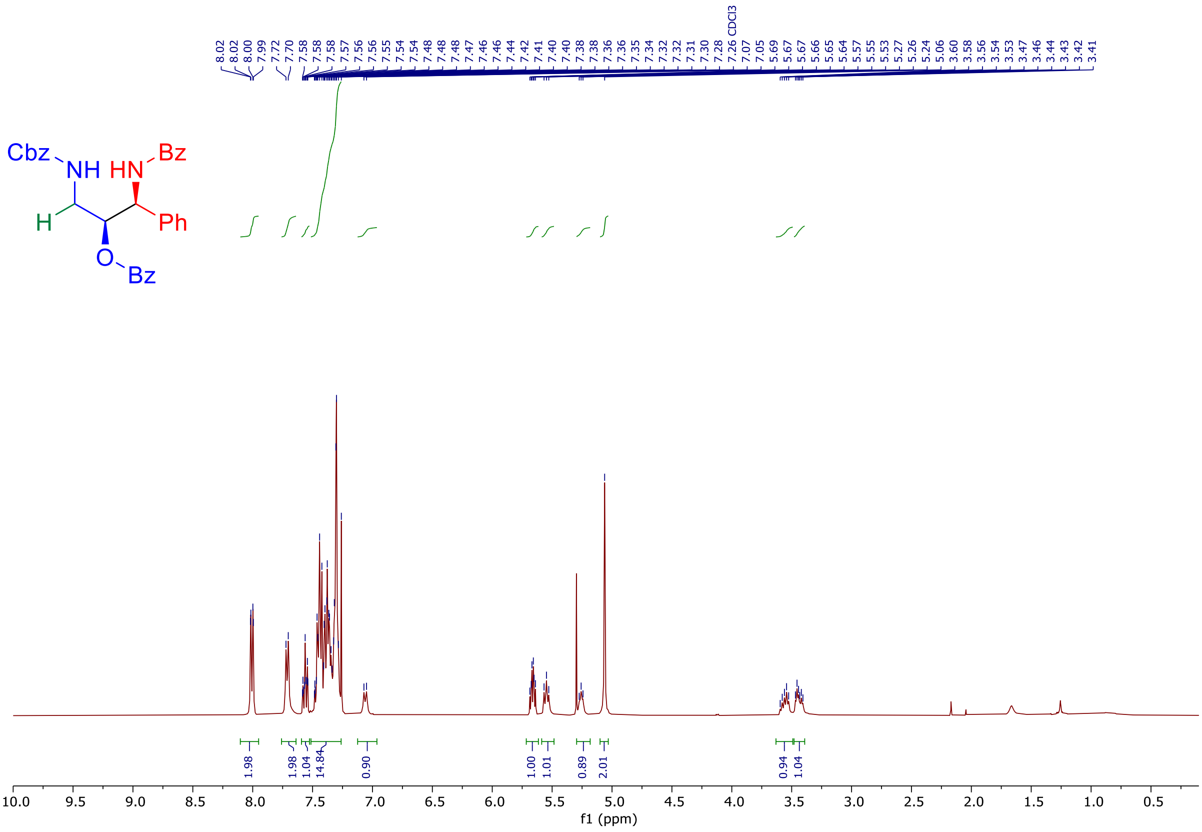

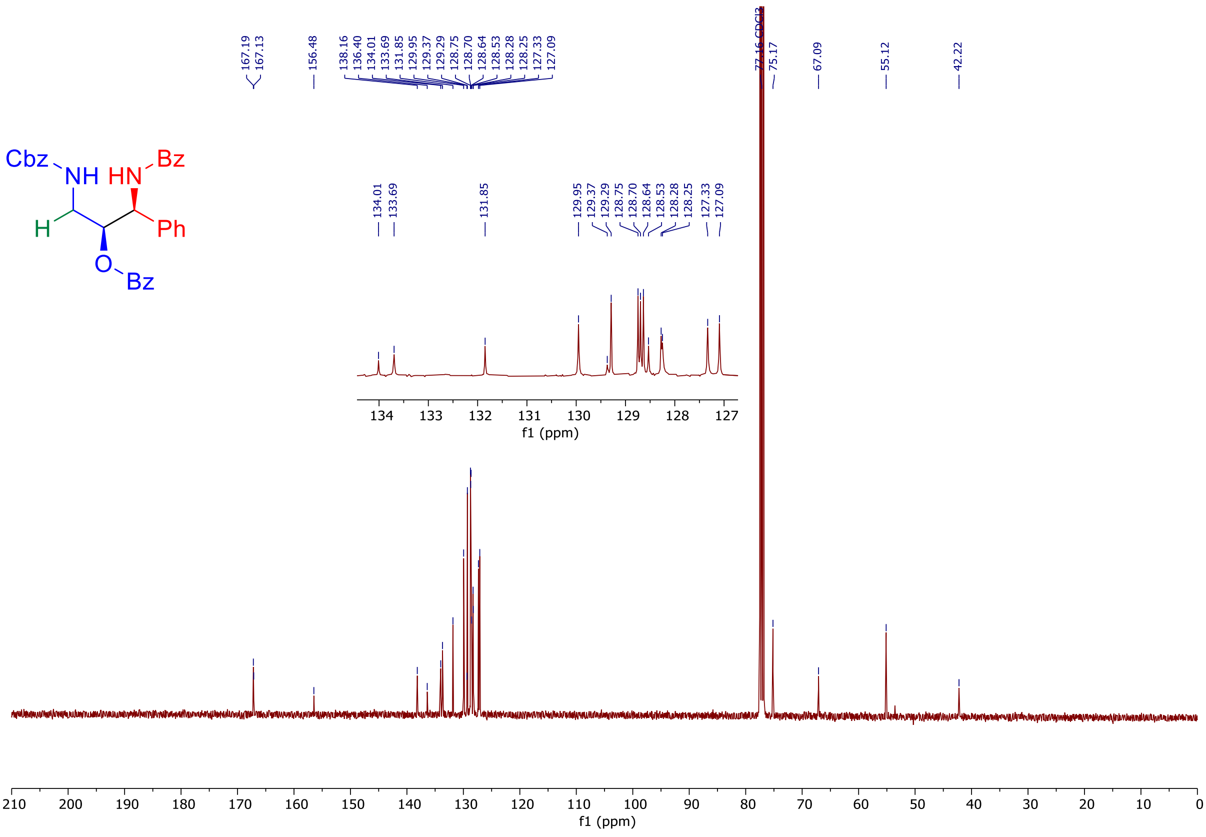

*Figure 21: ^{1}H (400 MHz) and ^{13}C (101 MHz) NMR spectra of **5e** in $CDCl_3$.*

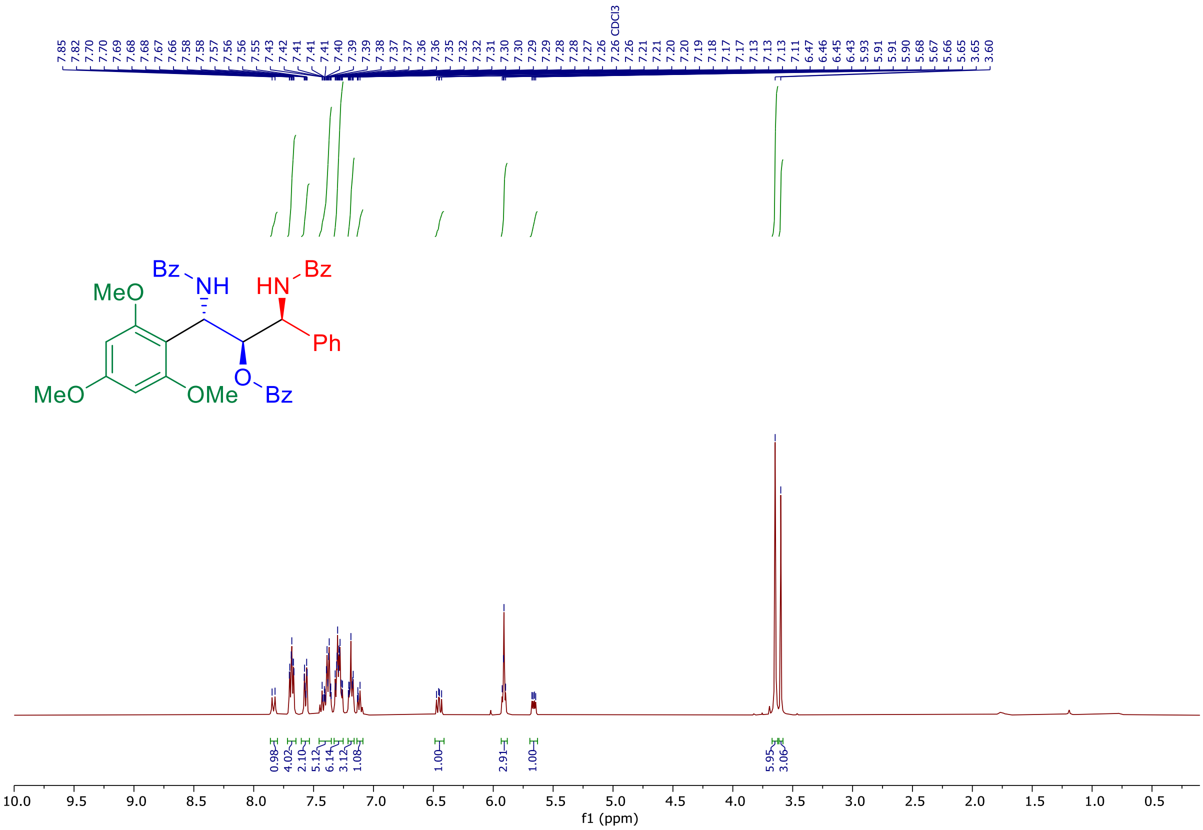

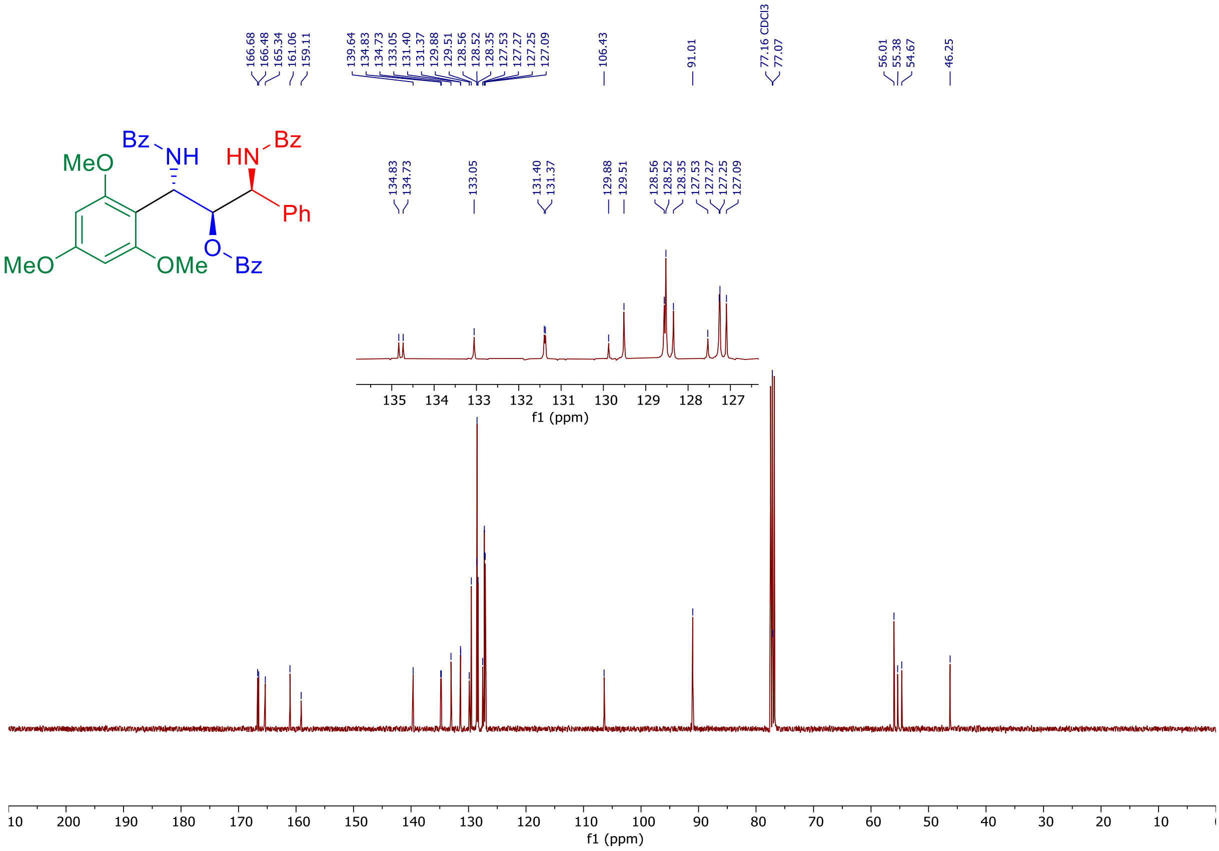

*Figure 22: ^{1}H (400 MHz) and ^{13}C (101 MHz) NMR spectra of **6a** in $CDCl_3$.*

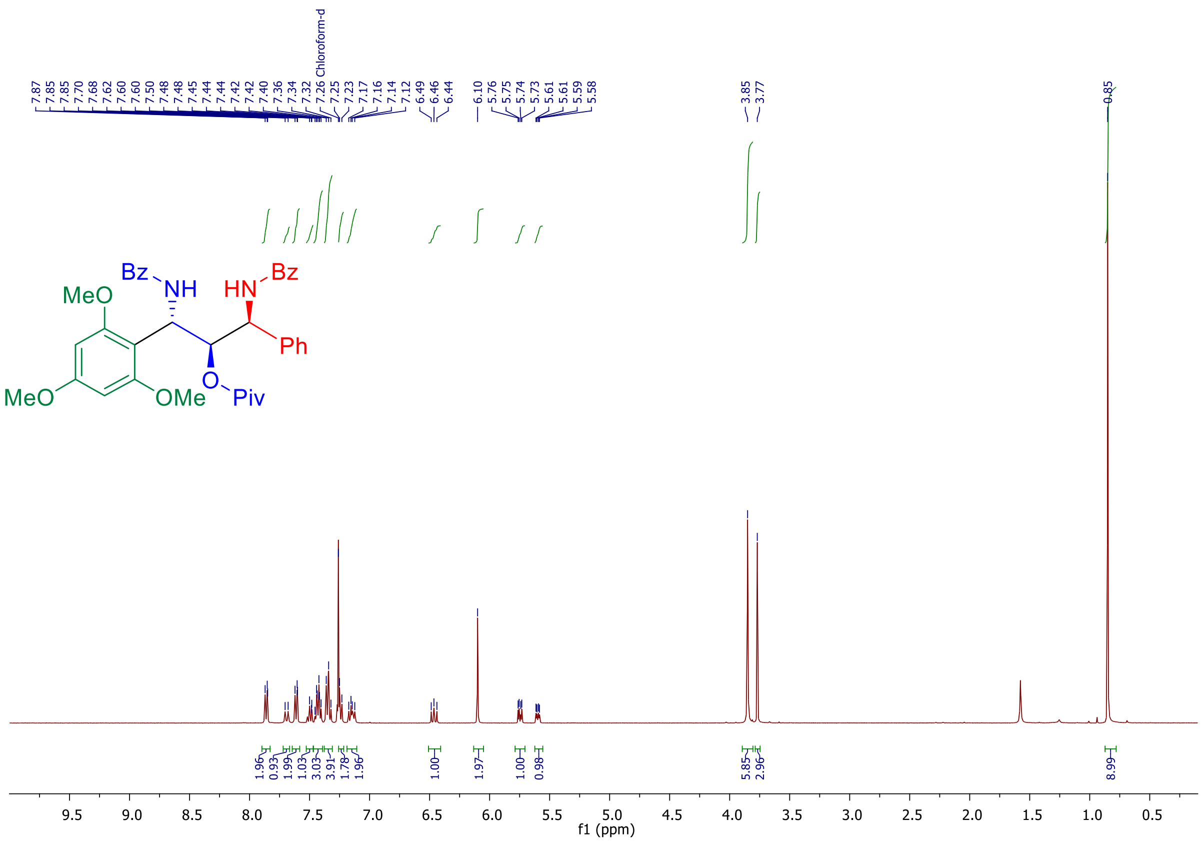

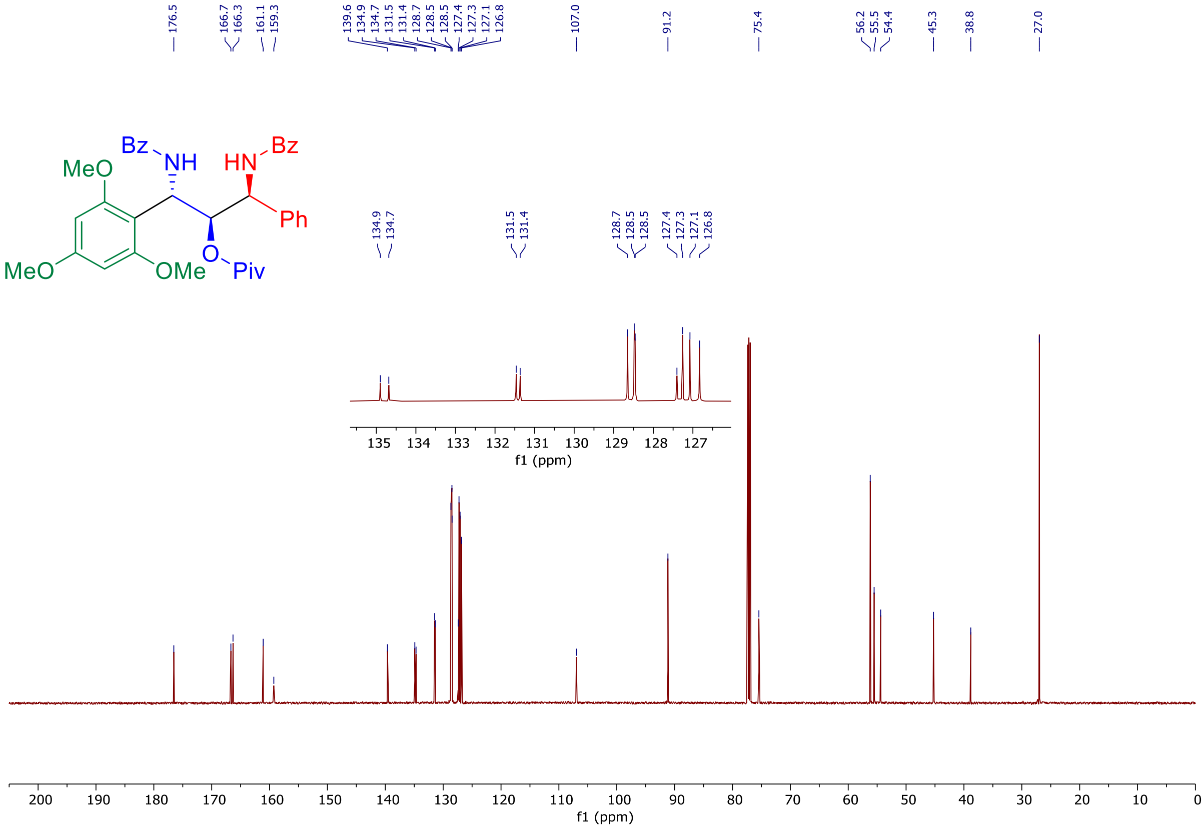

*Figure 23: ^{1}H (400 MHz) and ^{13}C (101 MHz) NMR spectra of **6b** in $CDCl_3$.*

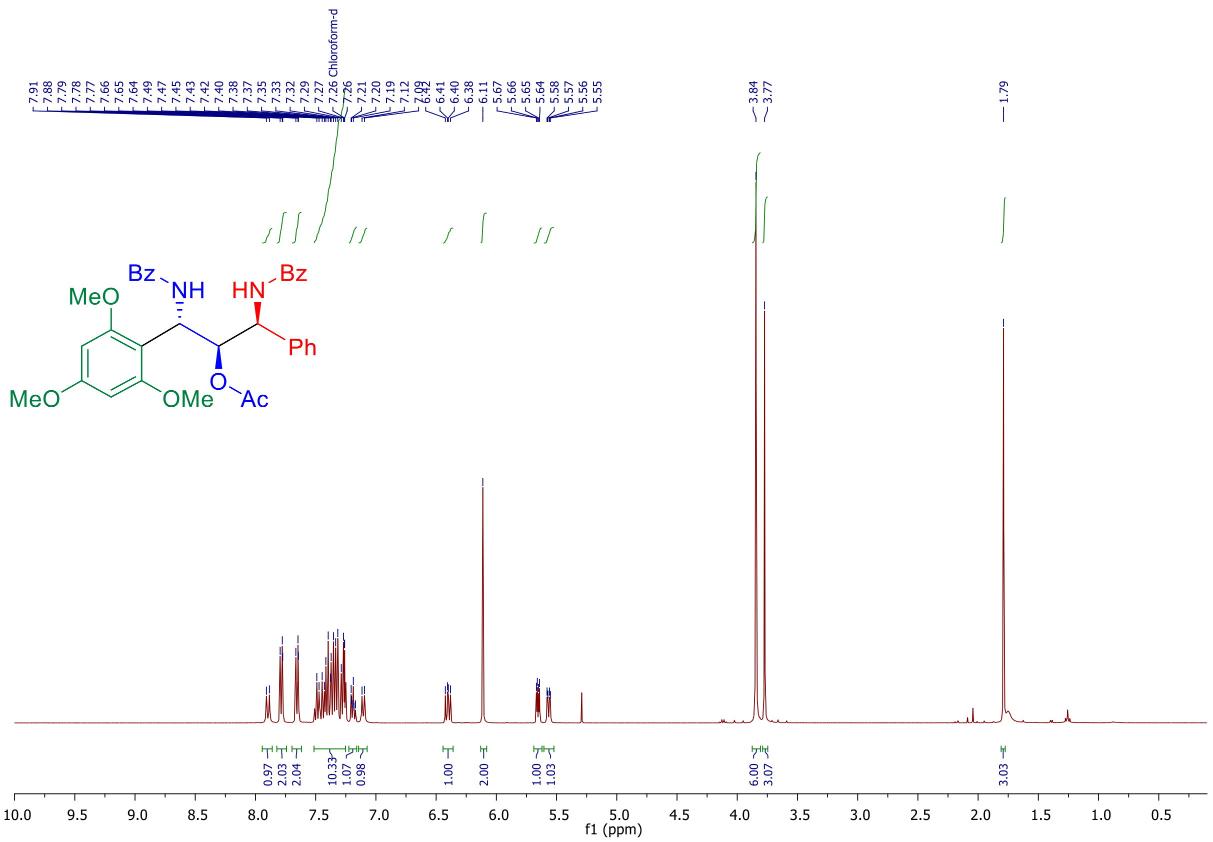

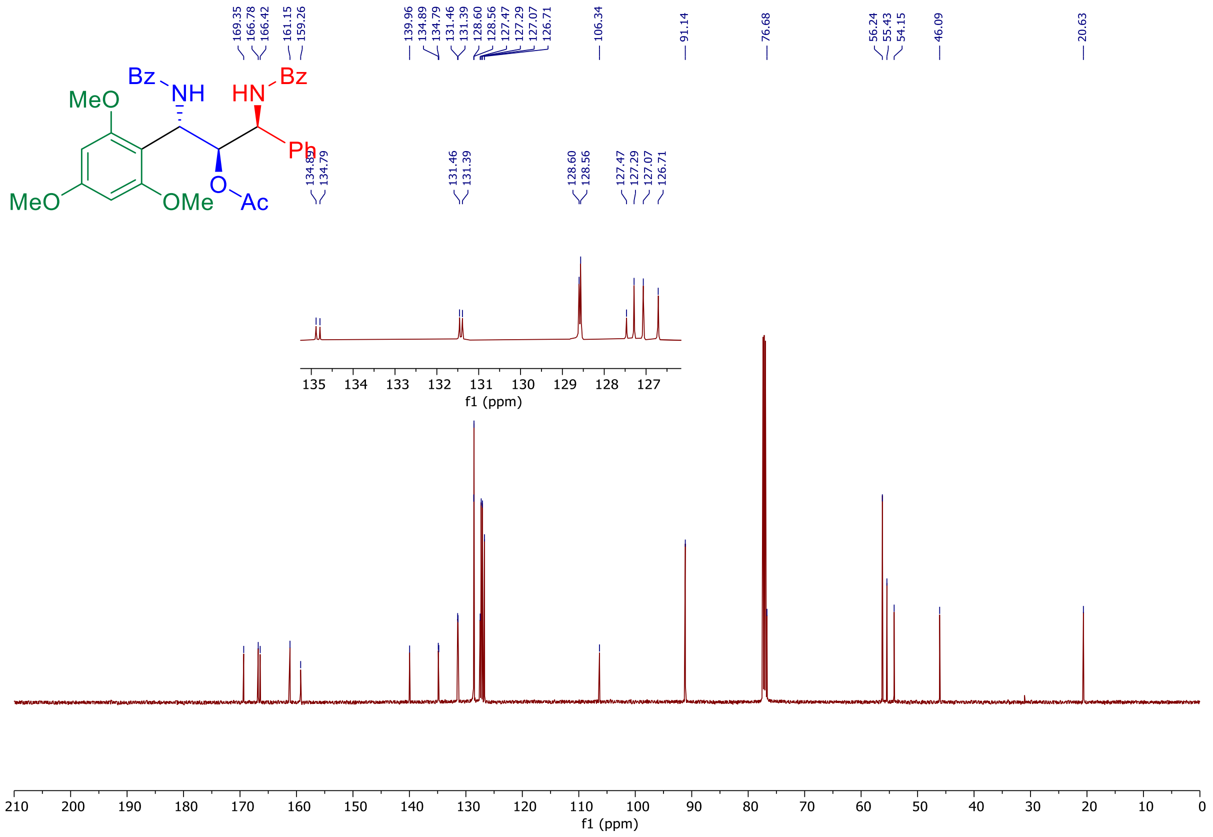

*Figure 24: ^{1}H (400 MHz) and ^{13}C (101 MHz) NMR spectra of **6c** in $CDCl_3$.*

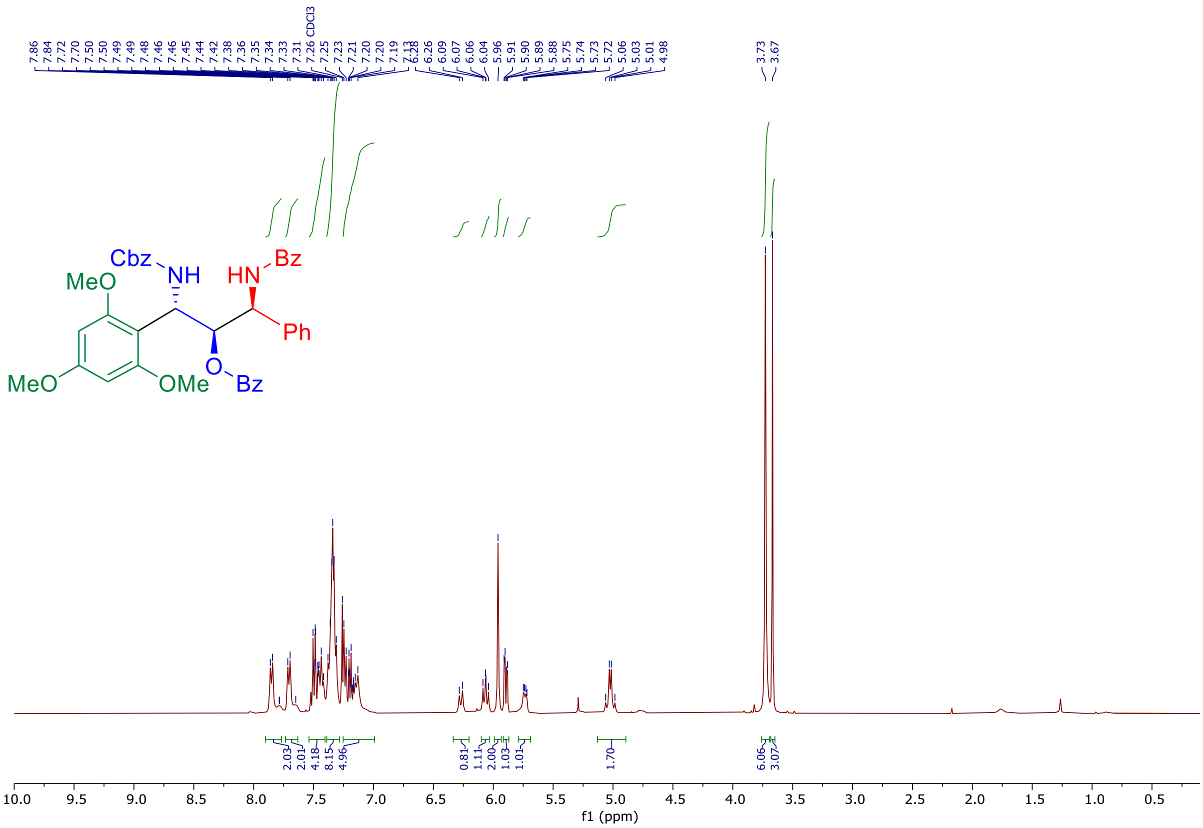

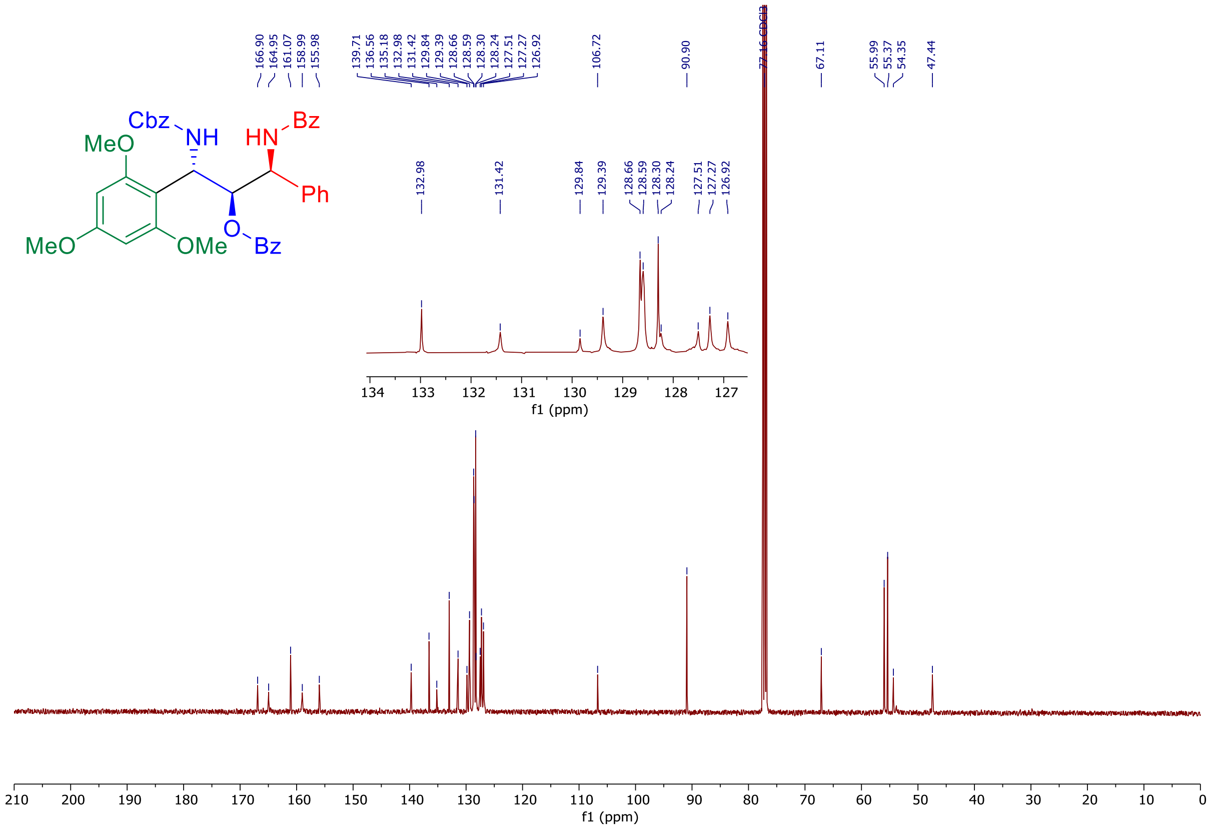

*Figure 25: ¹H (400 MHz) and ¹³C (101 MHz) NMR spectra of **6e** in CDCl$_3$.*

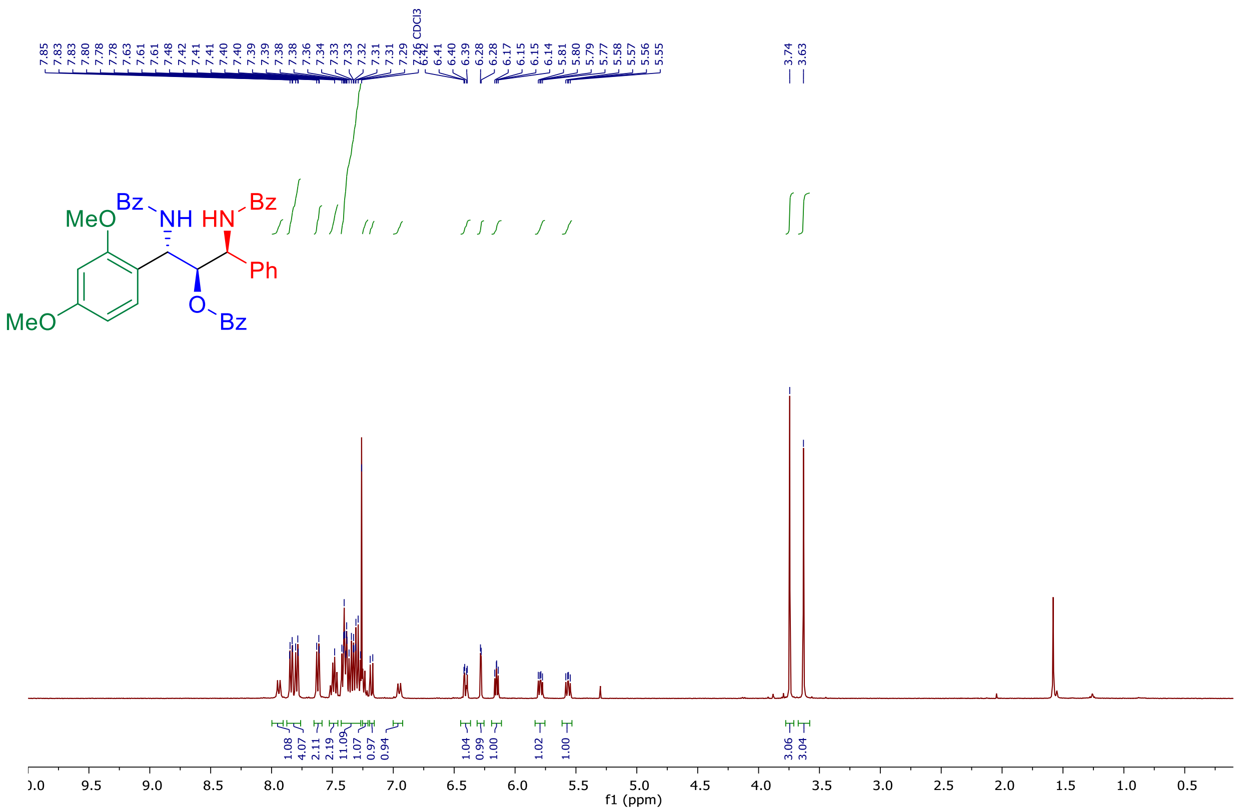

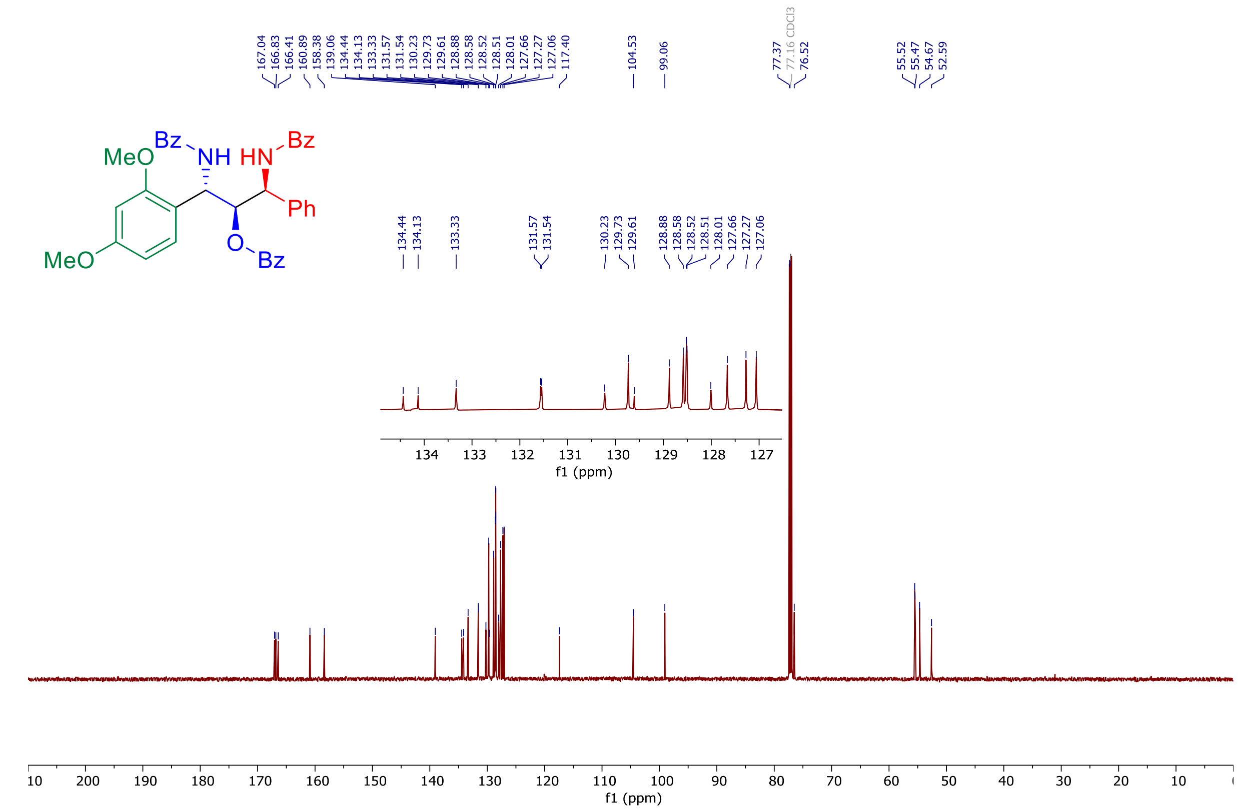

Figure 26: ^{1}H (400 MHz) and ^{13}C (101 MHz) NMR spectra of ***7a*** in $CDCl_3$.

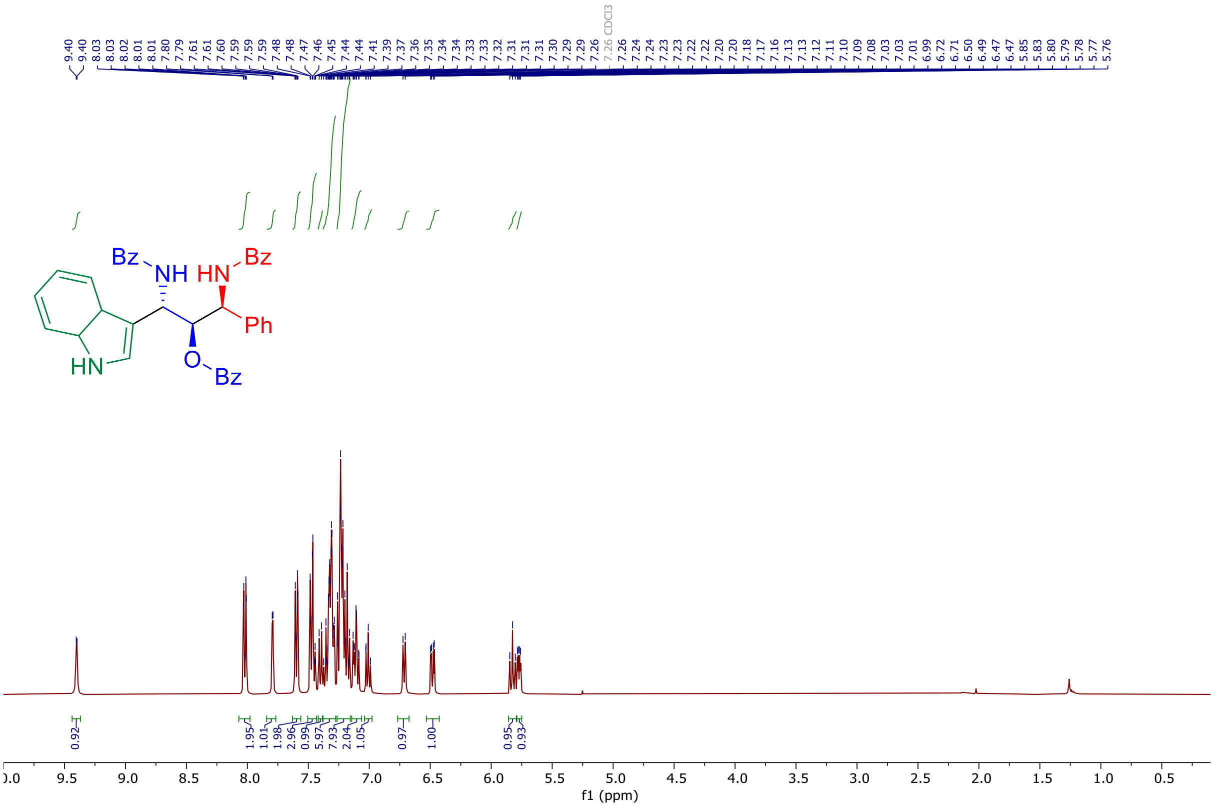

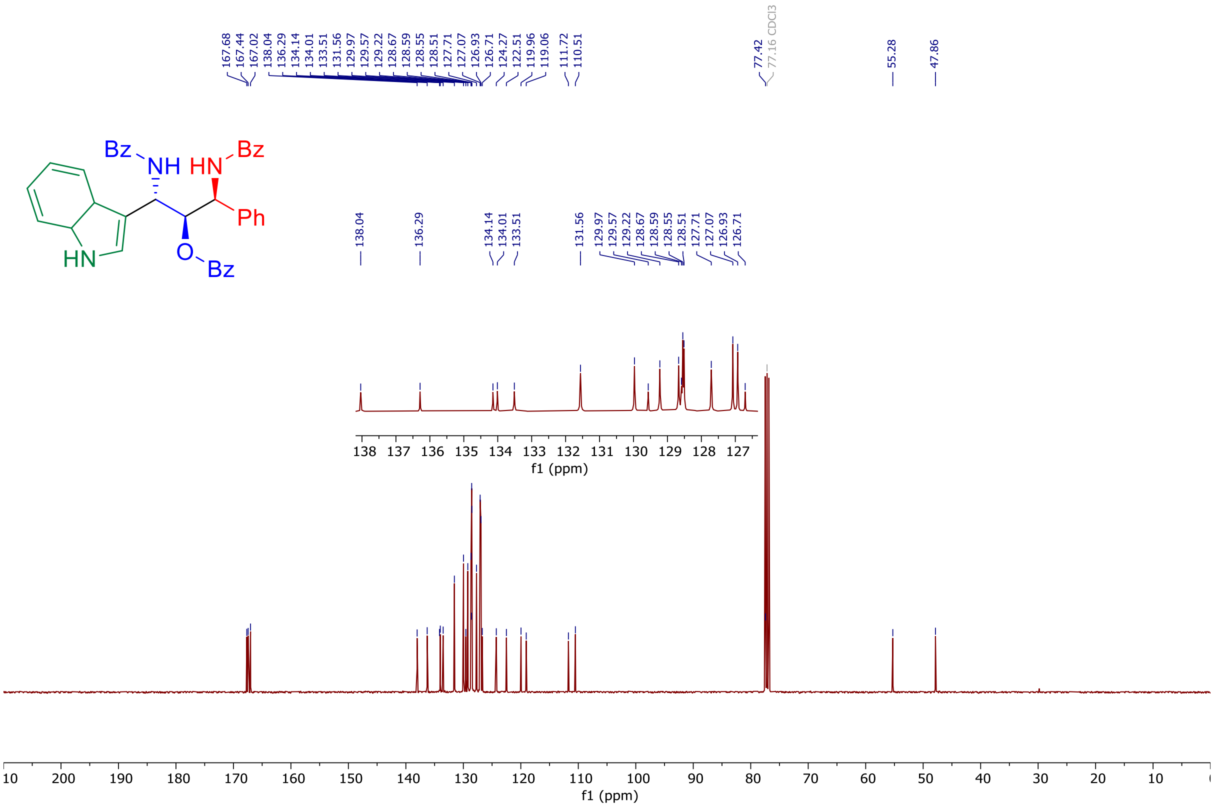

*Figure 27: ¹H (400 MHz) and ¹³C (101 MHz) NMR spectra of **7b** in $CDCl_3$.*

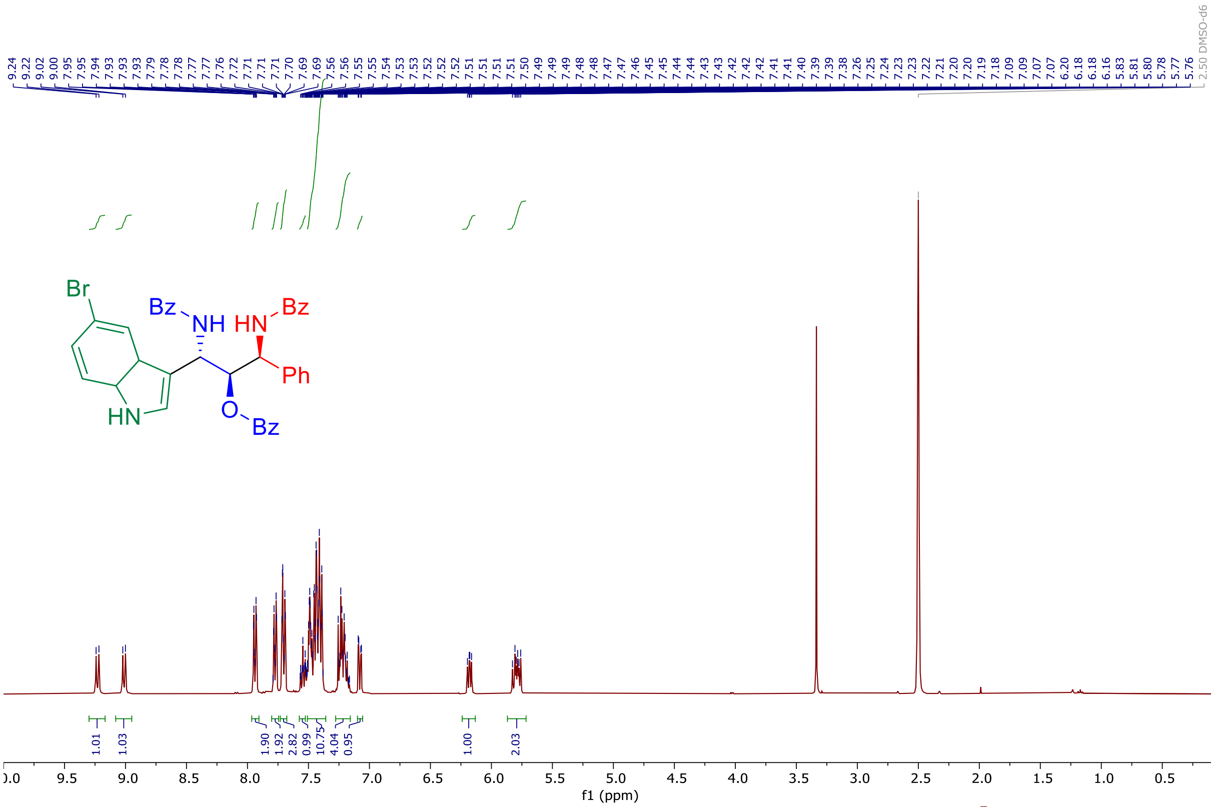

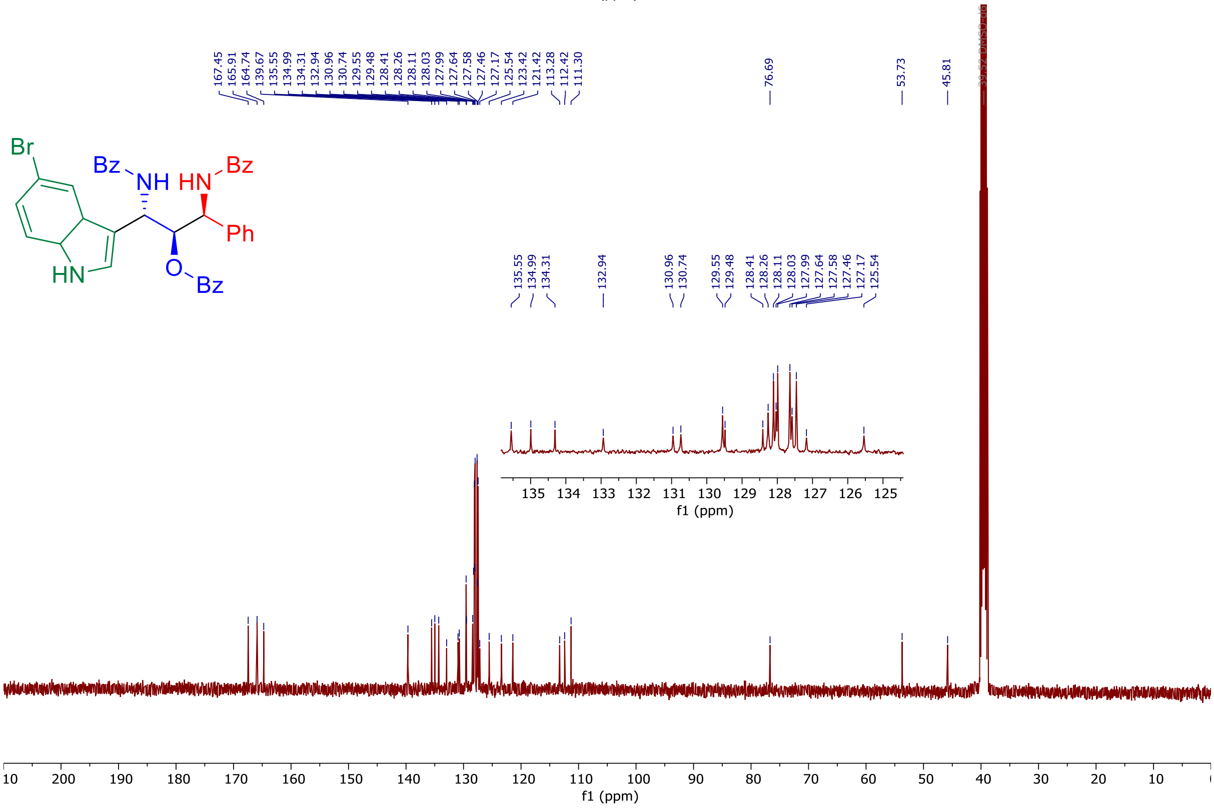

*Figure 28: ^{1}H (400 MHz) and ^{13}C (101 MHz) NMR spectra of **7c** in DMSO-d_6.*

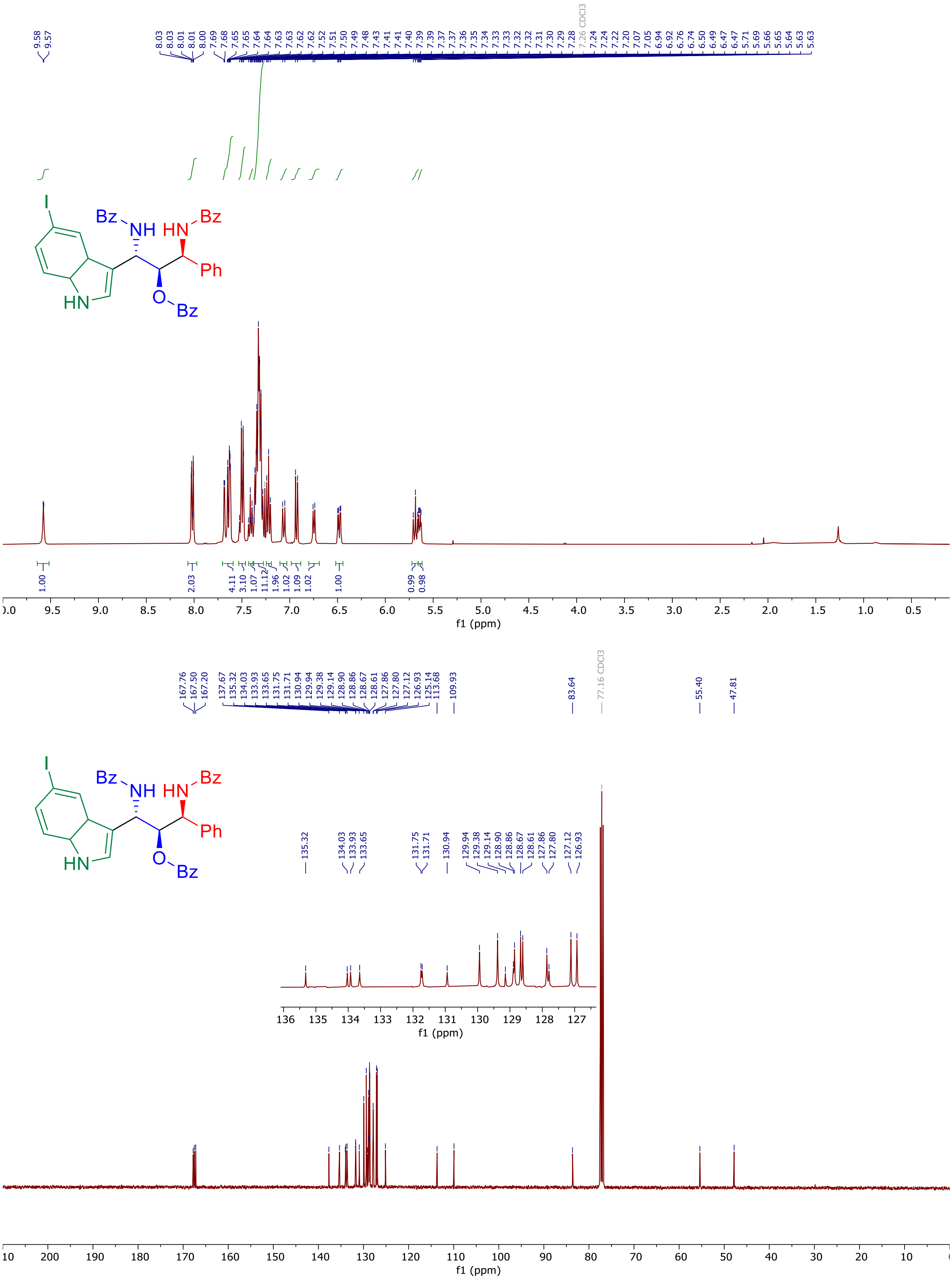

*Figure 29: ¹H (400 MHz) and ¹³C (101 MHz) NMR spectra of **7d** in $CDCl_3$.*

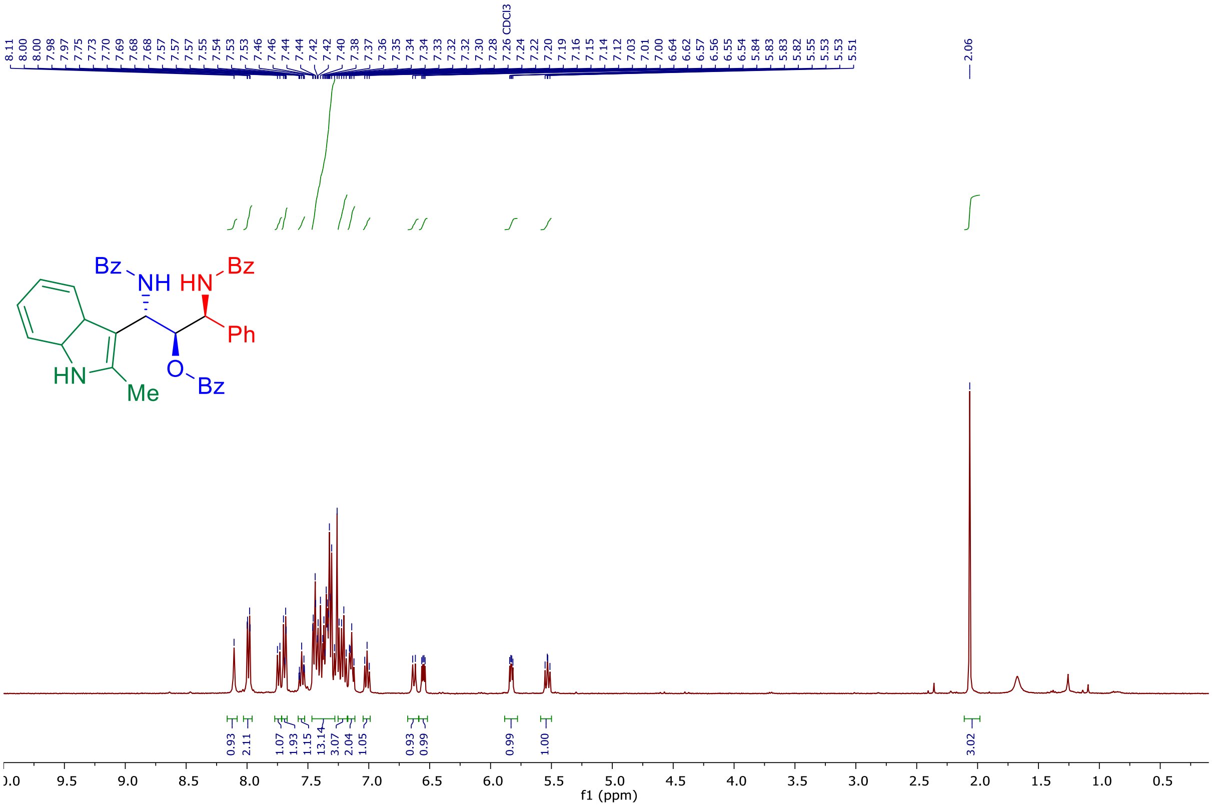

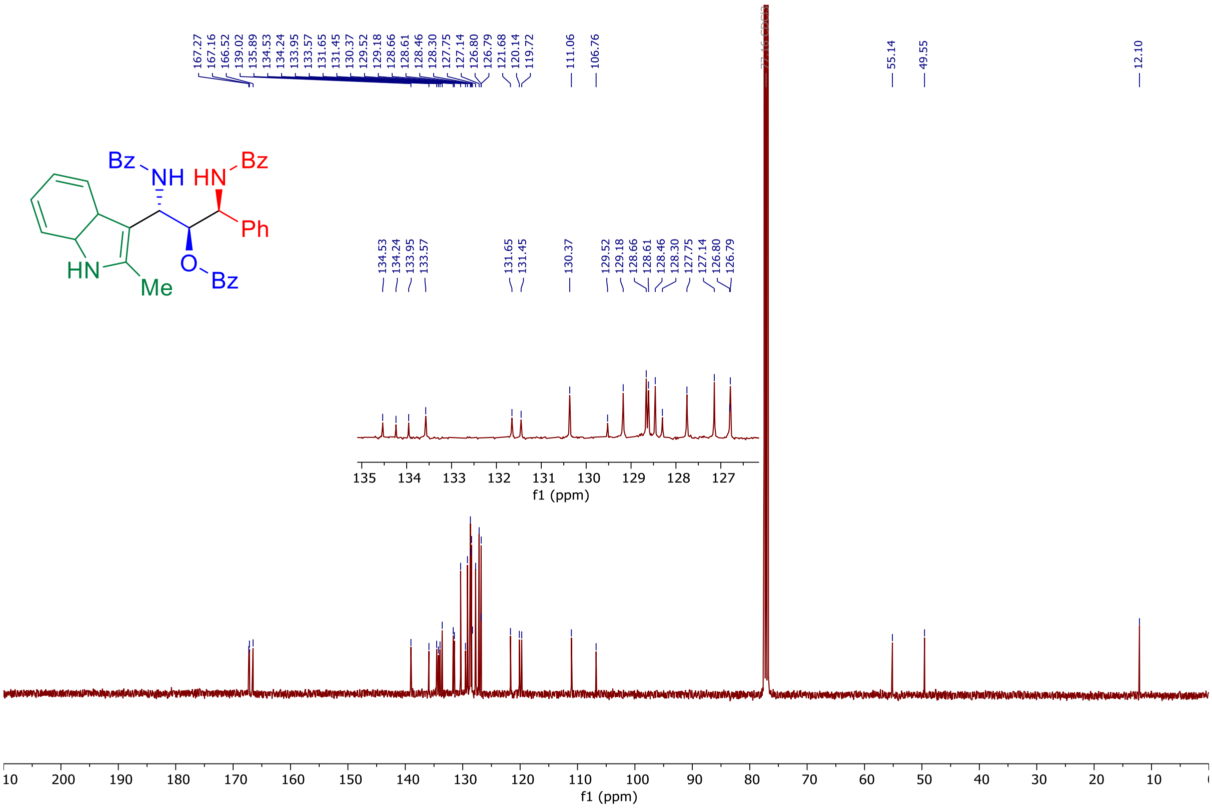

*Figure 30: ^{1}H (400 MHz) and ^{13}C (101 MHz) NMR spectra of **7e** in $CDCl_3$.*

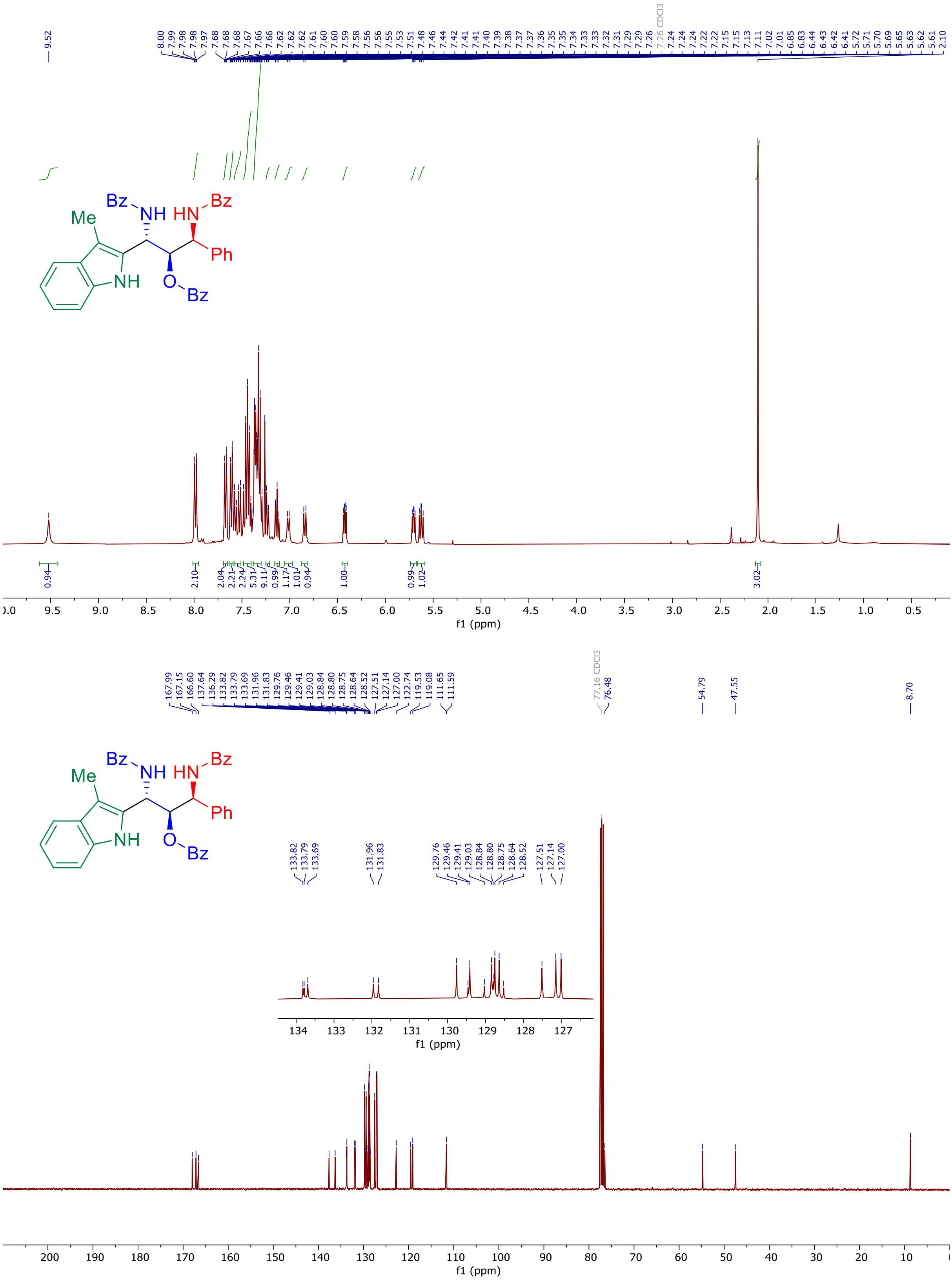

*Figure 31: ^{1}H (400 MHz) and ^{13}C (101 MHz) NMR spectra of **7f** in $CDCl_3$.*

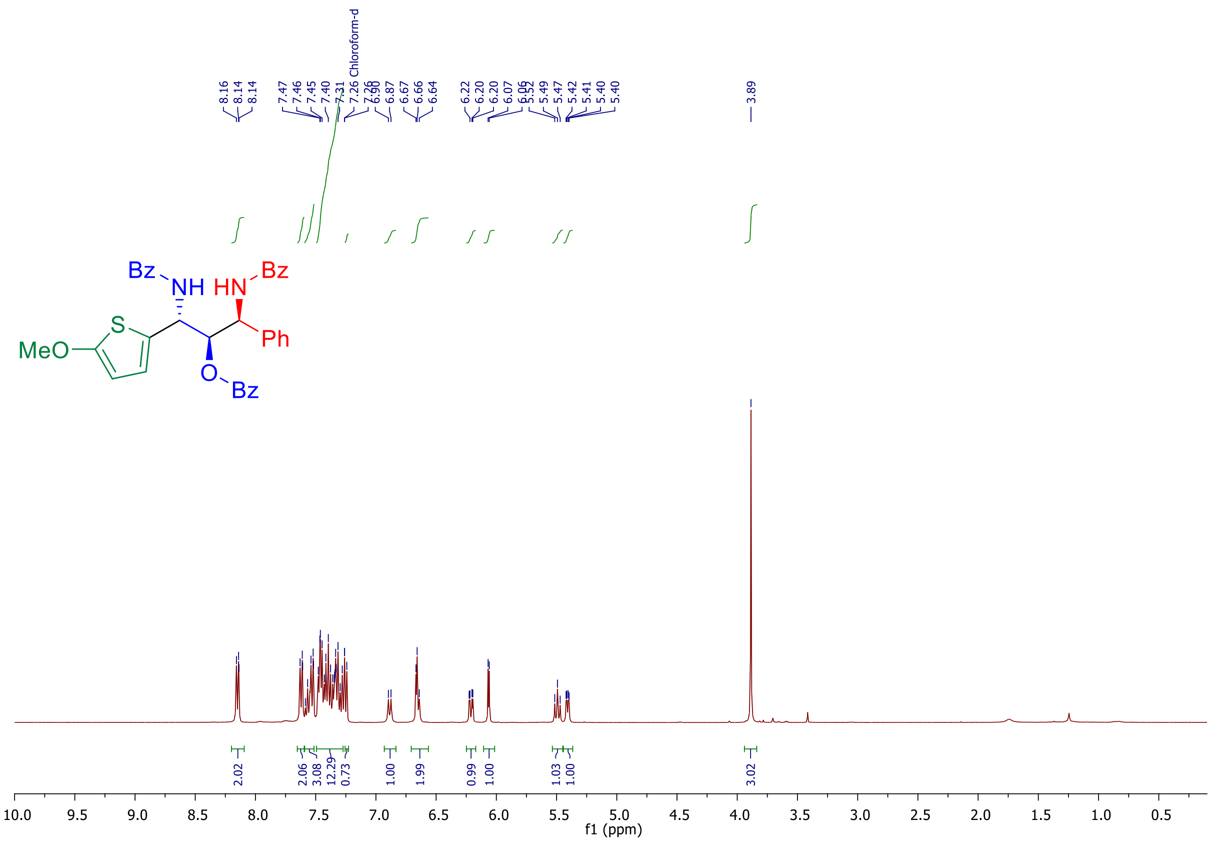

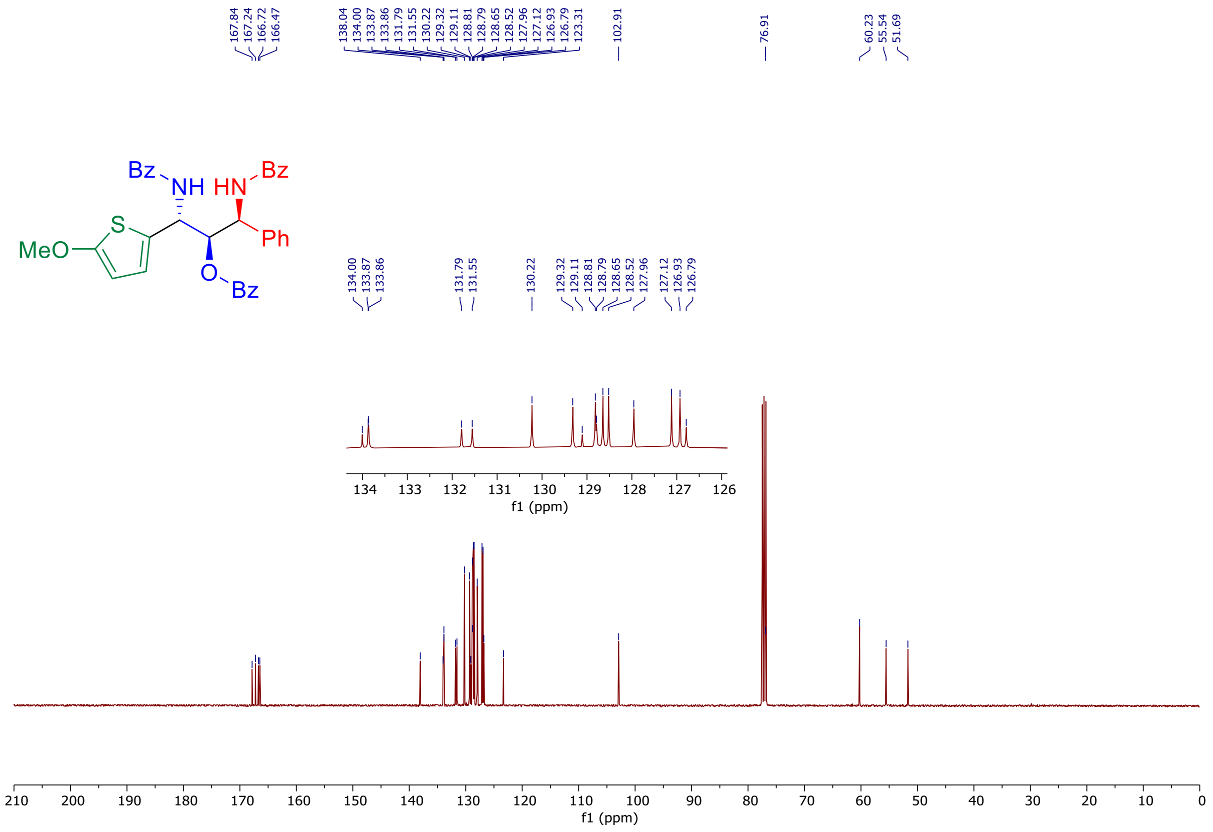

*Figure 32: ^{1}H (400 MHz) and ^{13}C (101 MHz) NMR spectra of **7g** in $CDCl_3$.*

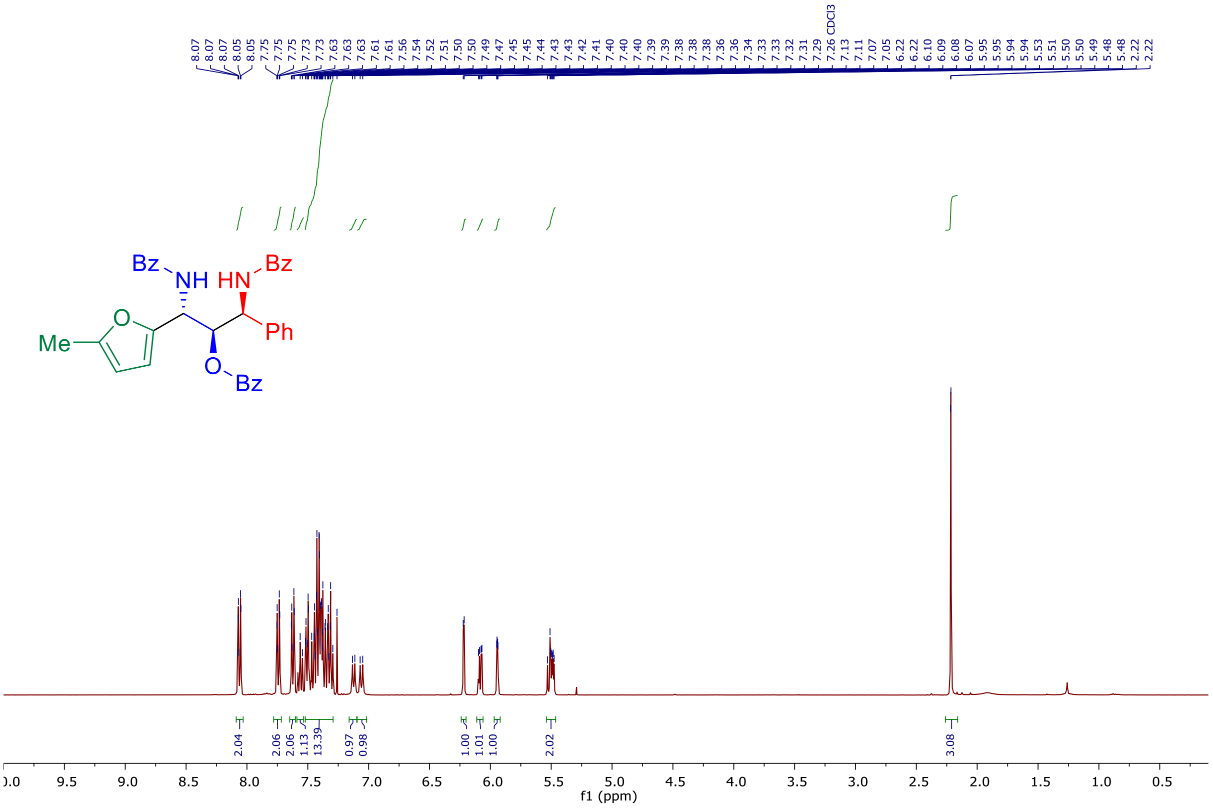

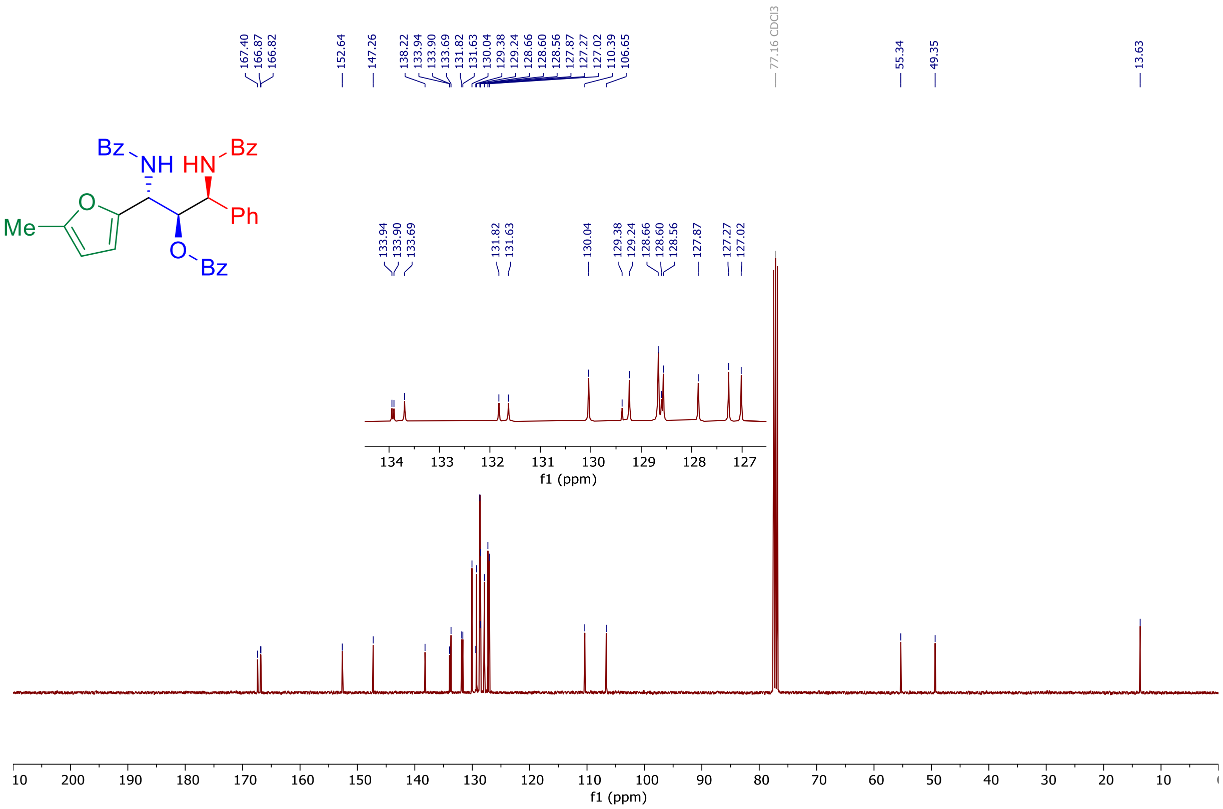

*Figure 33: ¹H (400 MHz) and ¹³C (101 MHz) NMR spectra of **7h** in CDCl₃.*

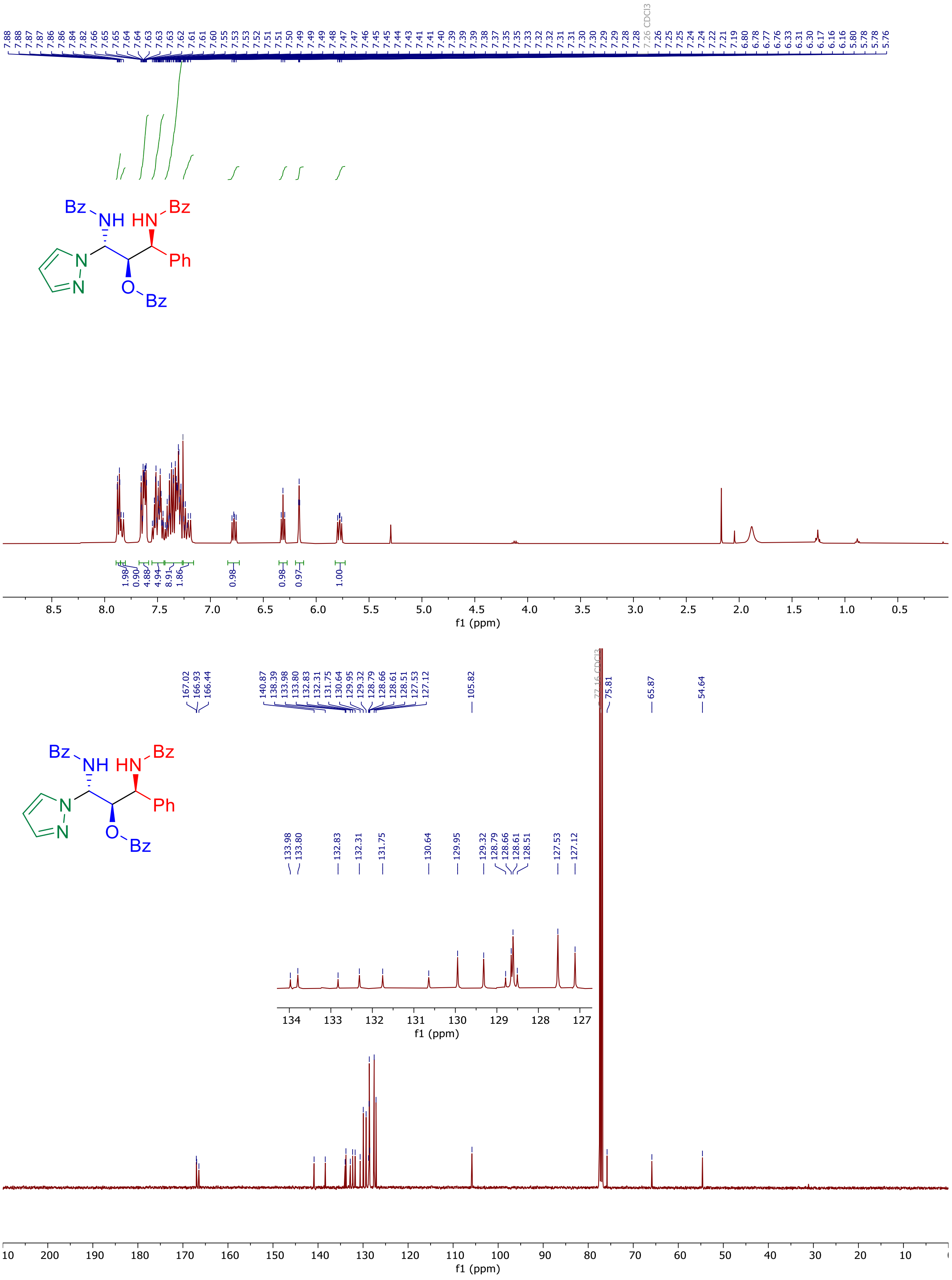

*Figure 34: ^{1}H (400 MHz) and ^{13}C (101 MHz) NMR spectra of **7g** in $CDCl_3$.*

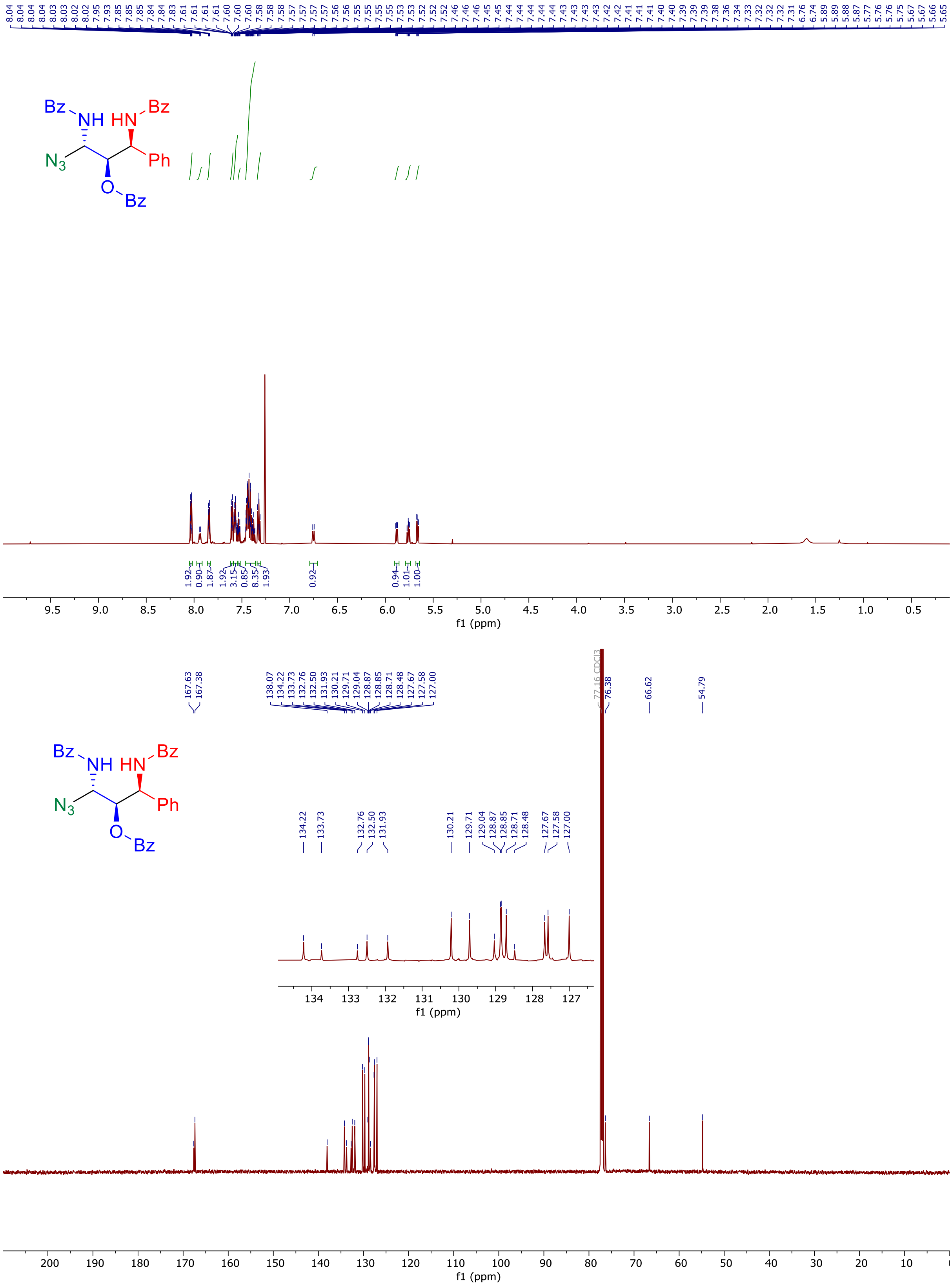

*Figure 35: ^{1}H (400 MHz) and ^{13}C (101 MHz) NMR spectra of **7h** in $CDCl_3$.*

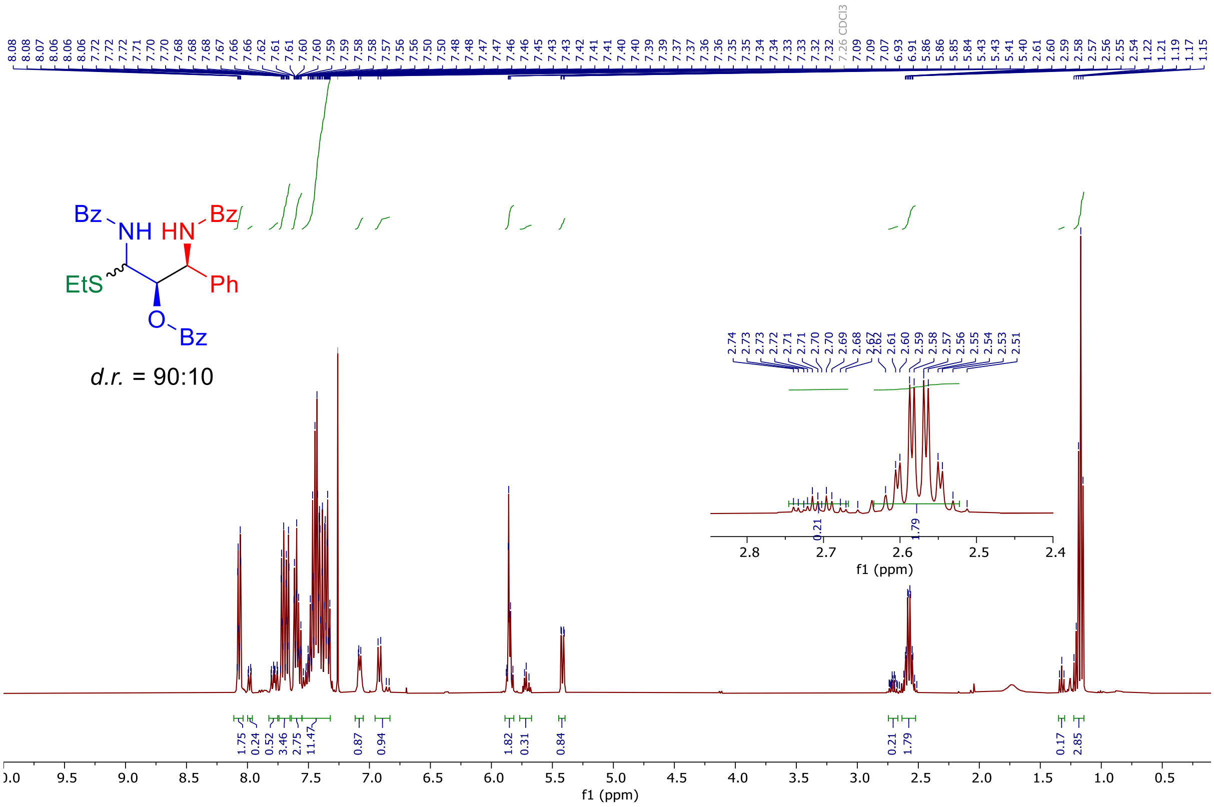

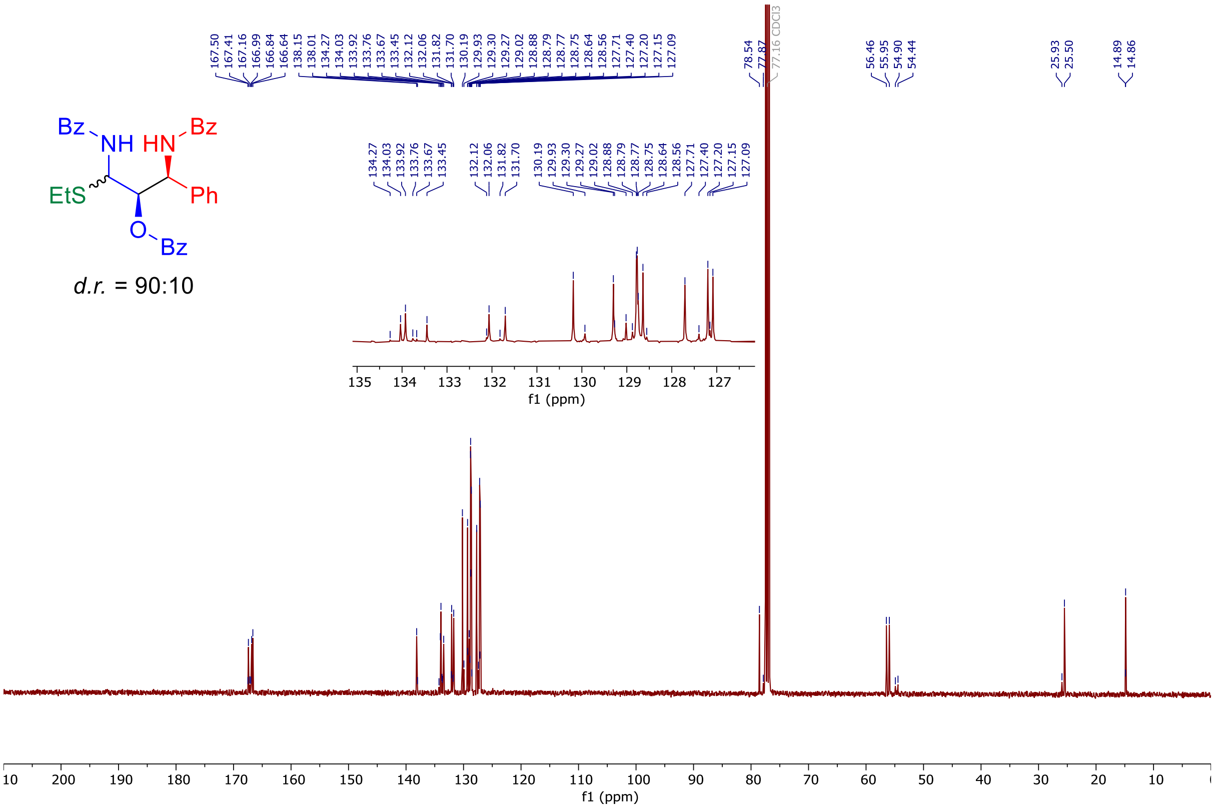

*Figure 36: ^{1}H (400 MHz) and ^{13}C (101 MHz) NMR spectra of **7i** in $CDCl_3$.*

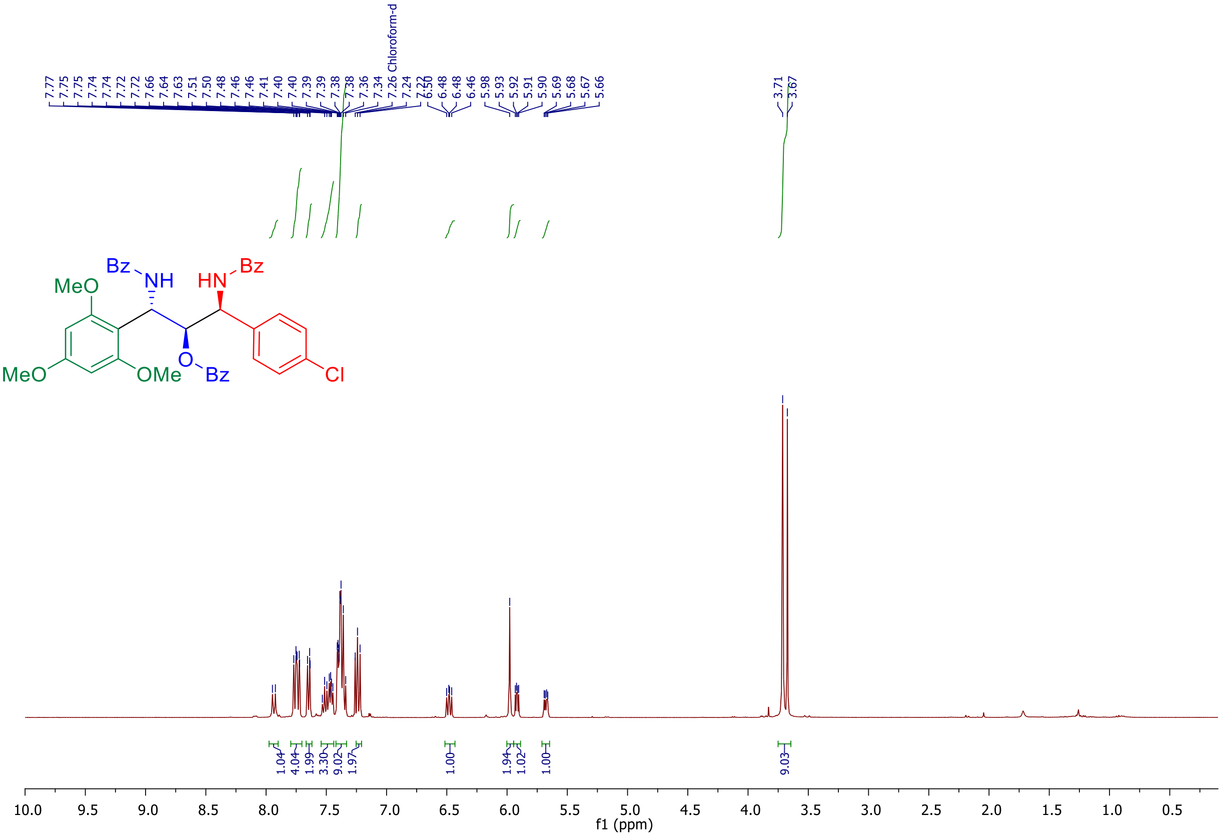

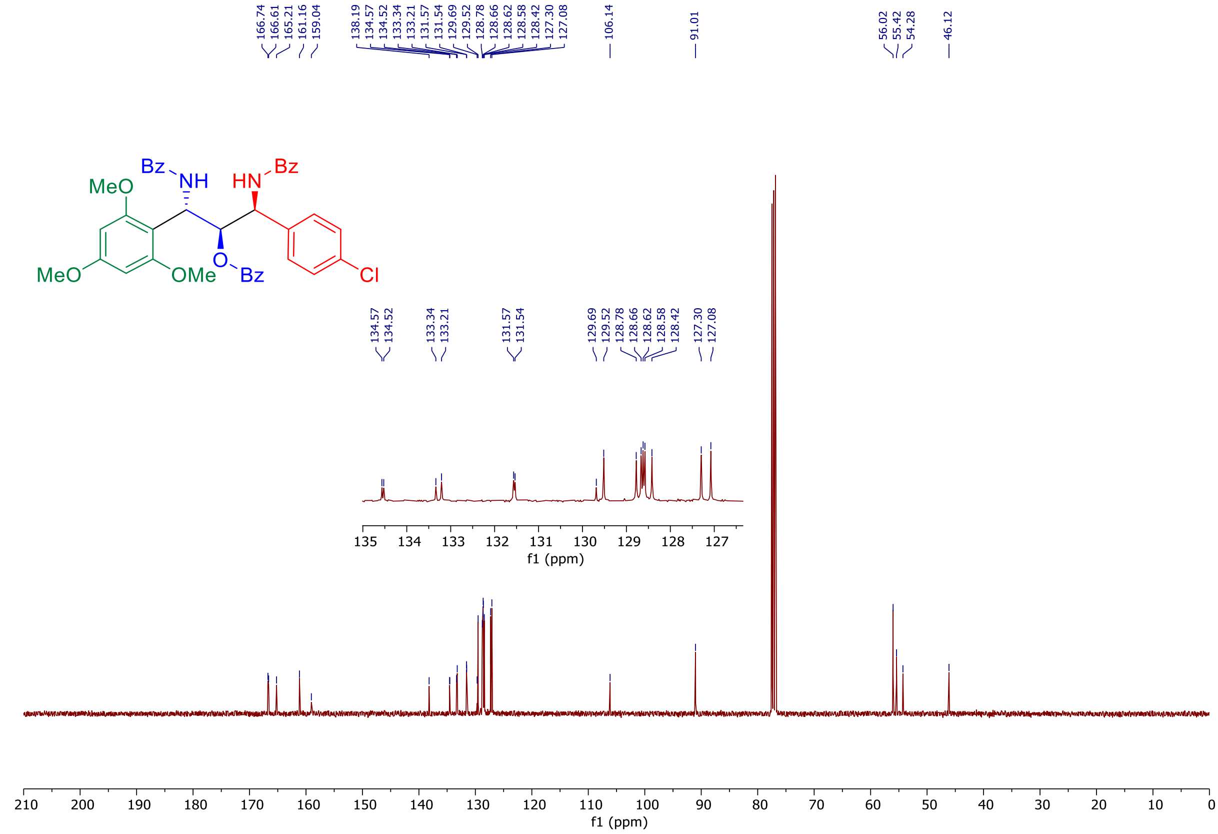

*Figure 37: ^{1}H (400 MHz) and ^{13}C (101 MHz) NMR spectra of **8a** in $CDCl_3$.*

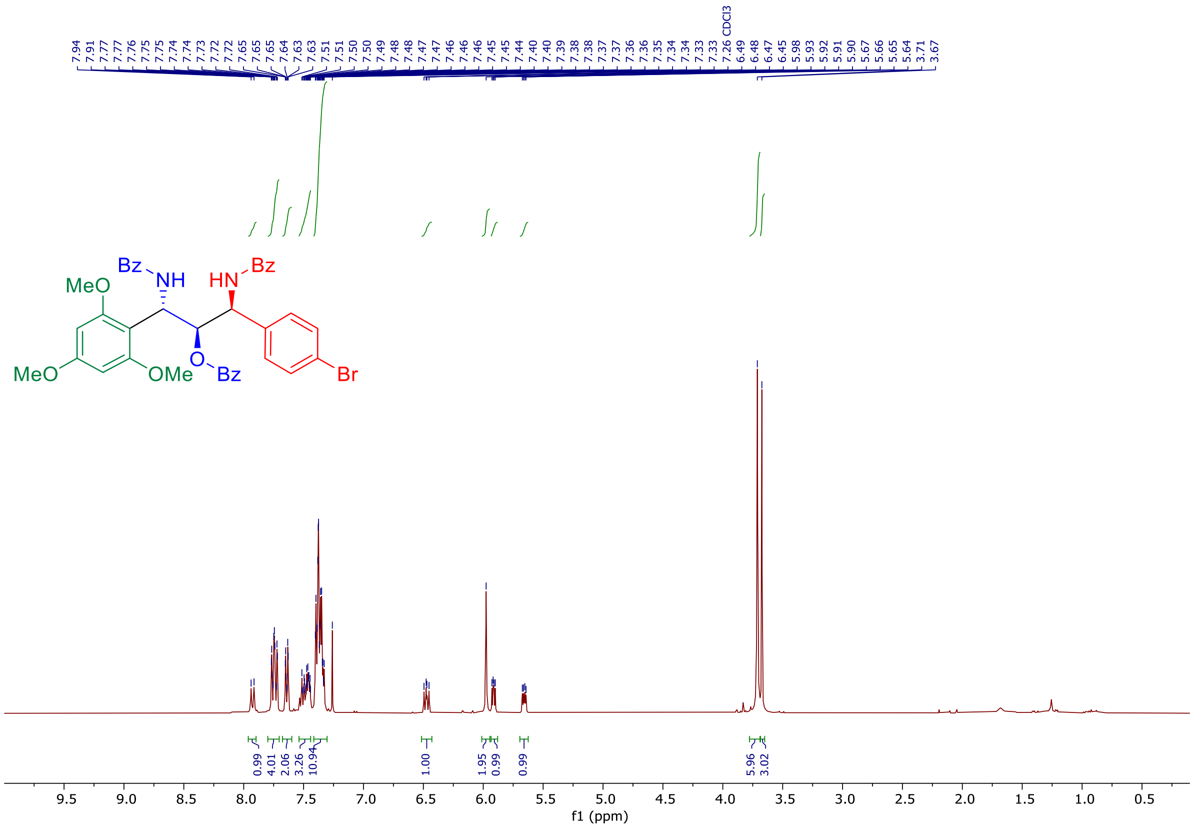

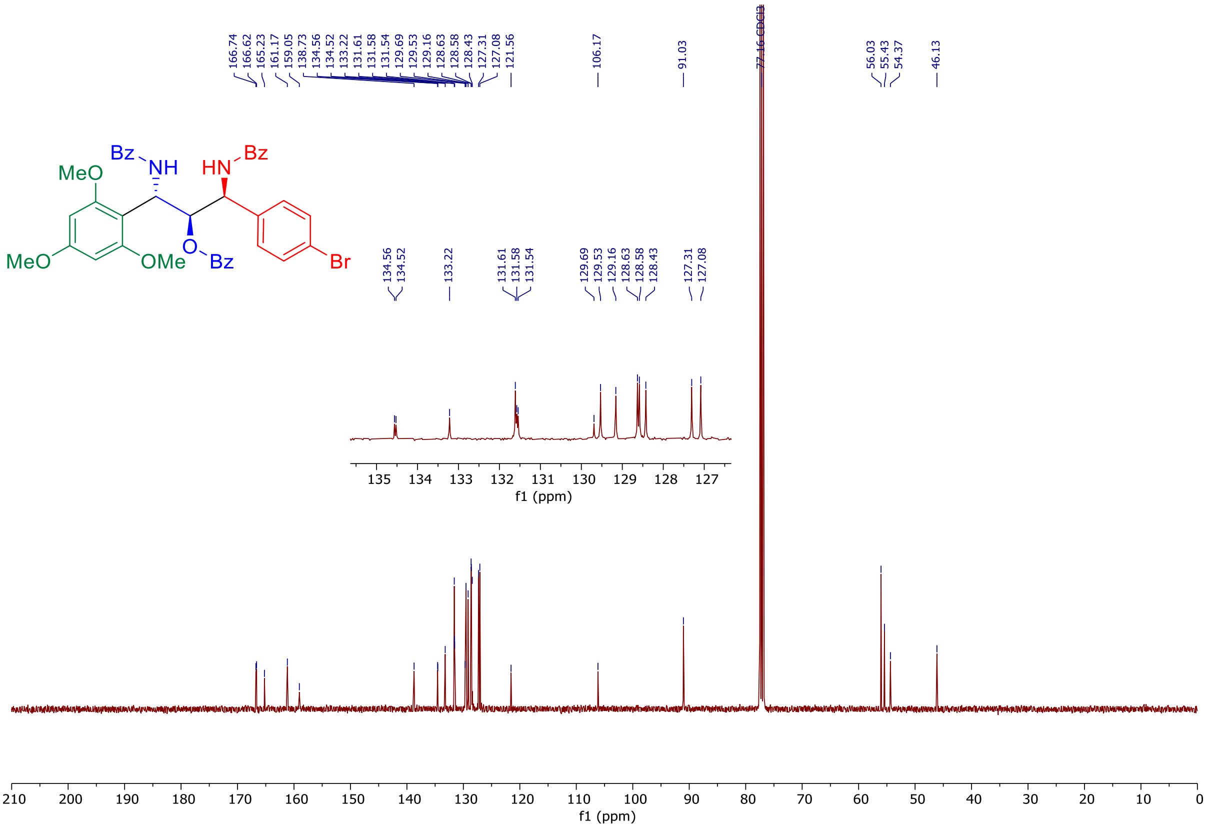

*Figure 38: ¹H (400 MHz) and ¹³C (101 MHz) NMR spectra of **8b** in CDCl₃.*

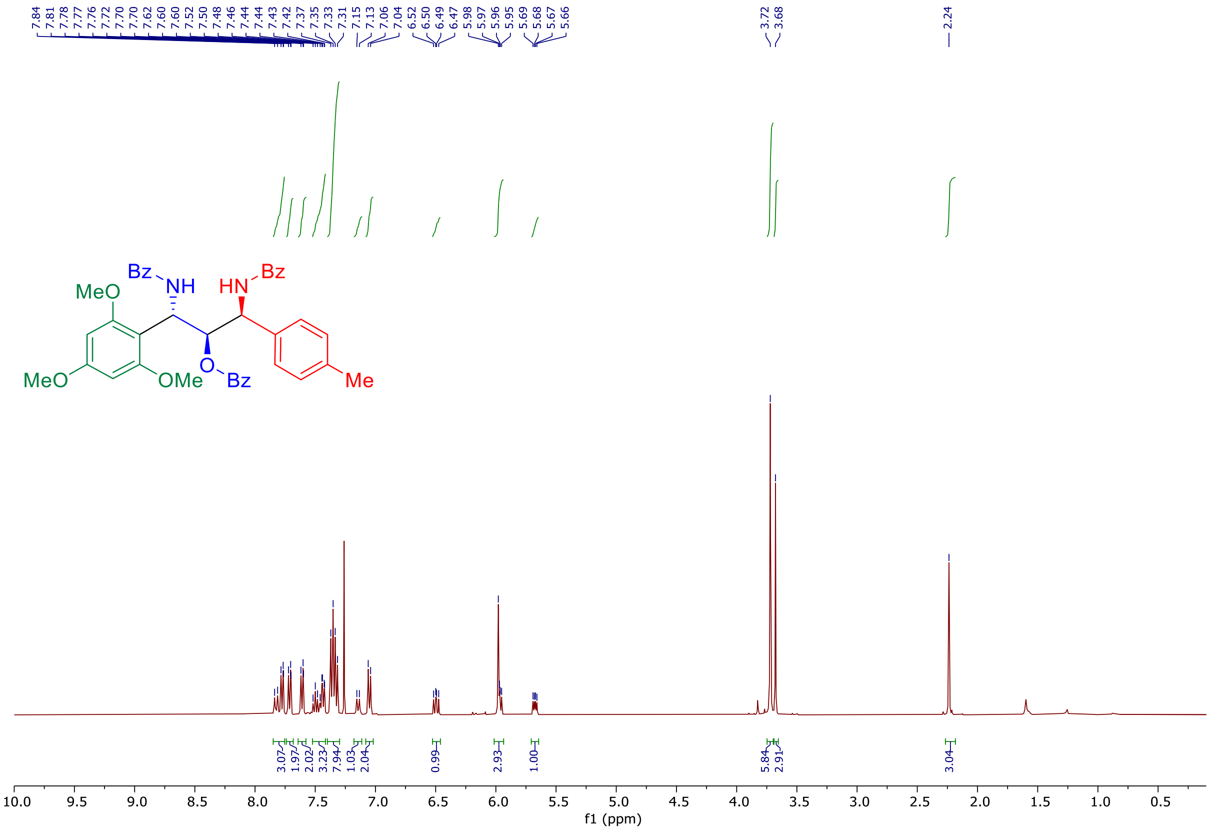

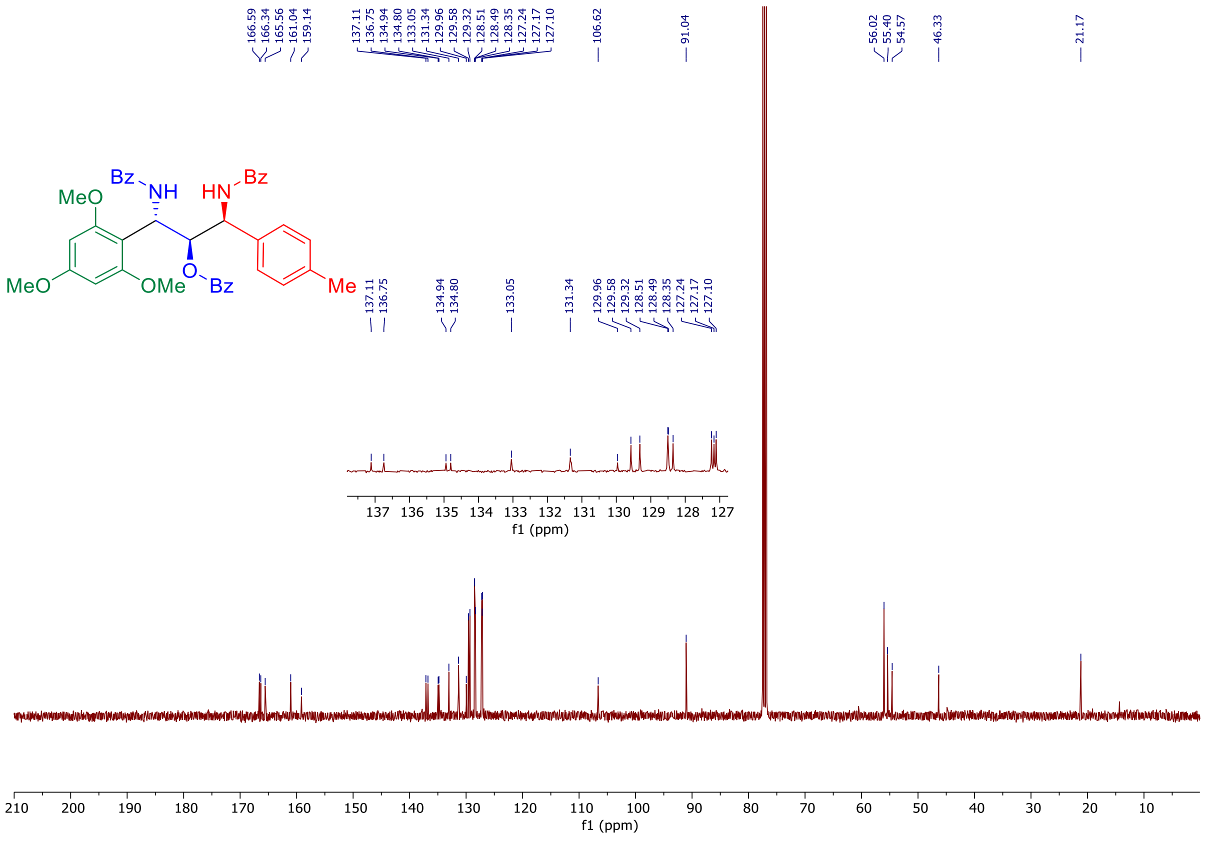

*Figure 39: ^{1}H (400 MHz) and ^{13}C (101 MHz) NMR spectra of **8c** in $CDCl_3$.*

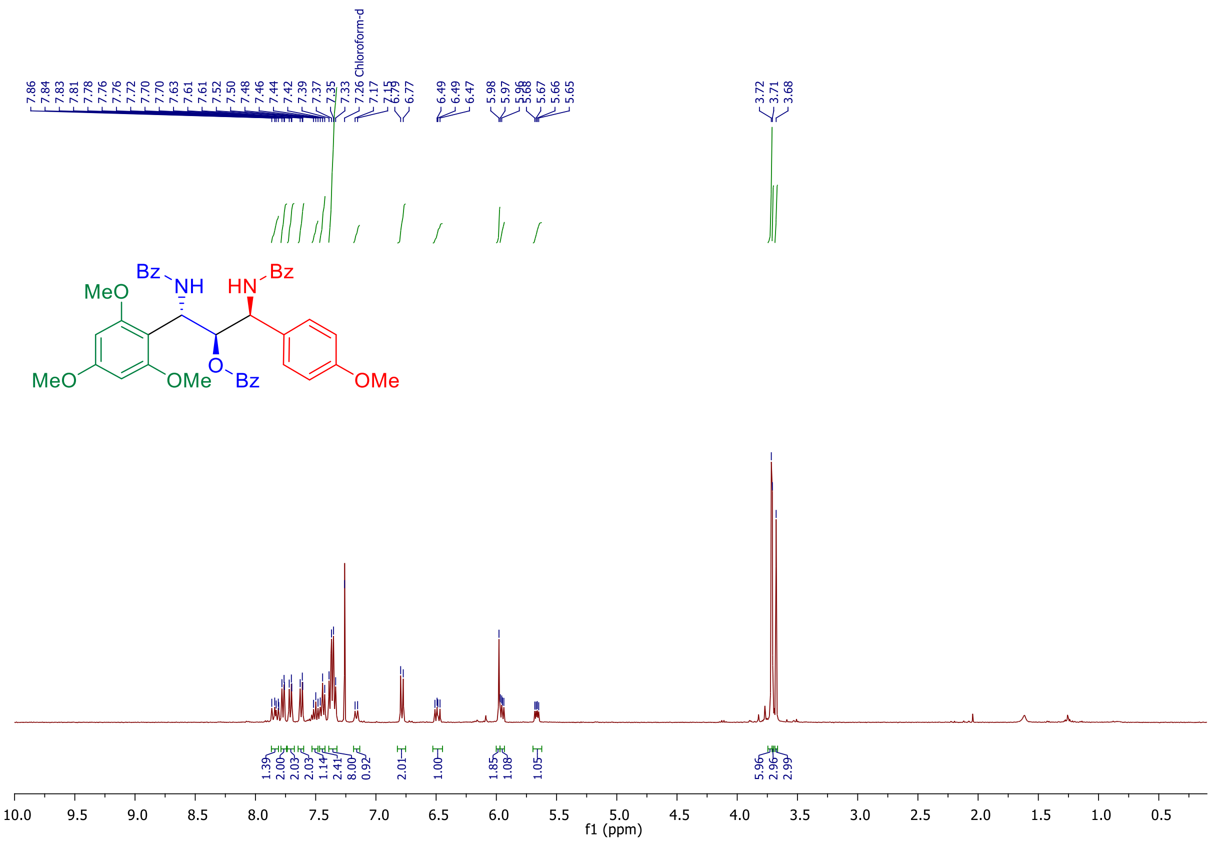

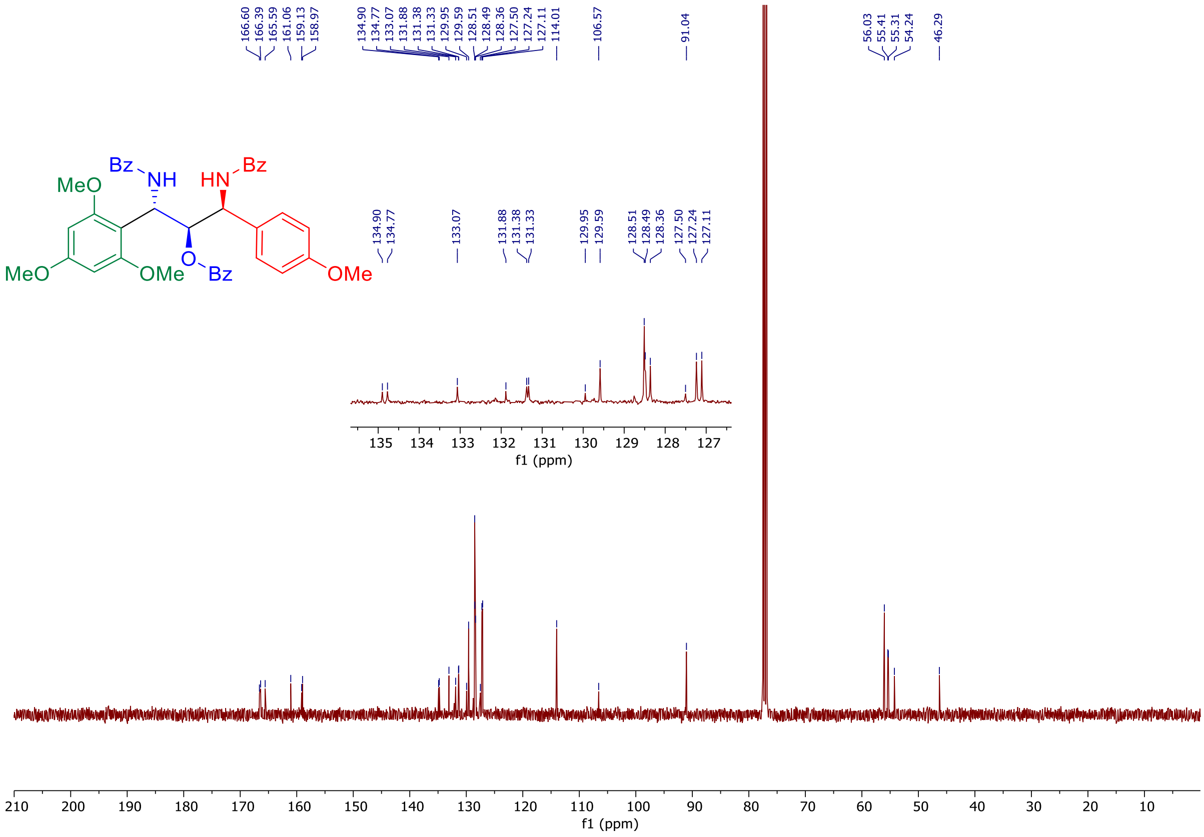

Figure 40: ^{1}H (400 MHz) and ^{13}C (101 MHz) NMR spectra of **8d** in $CDCl_3$.

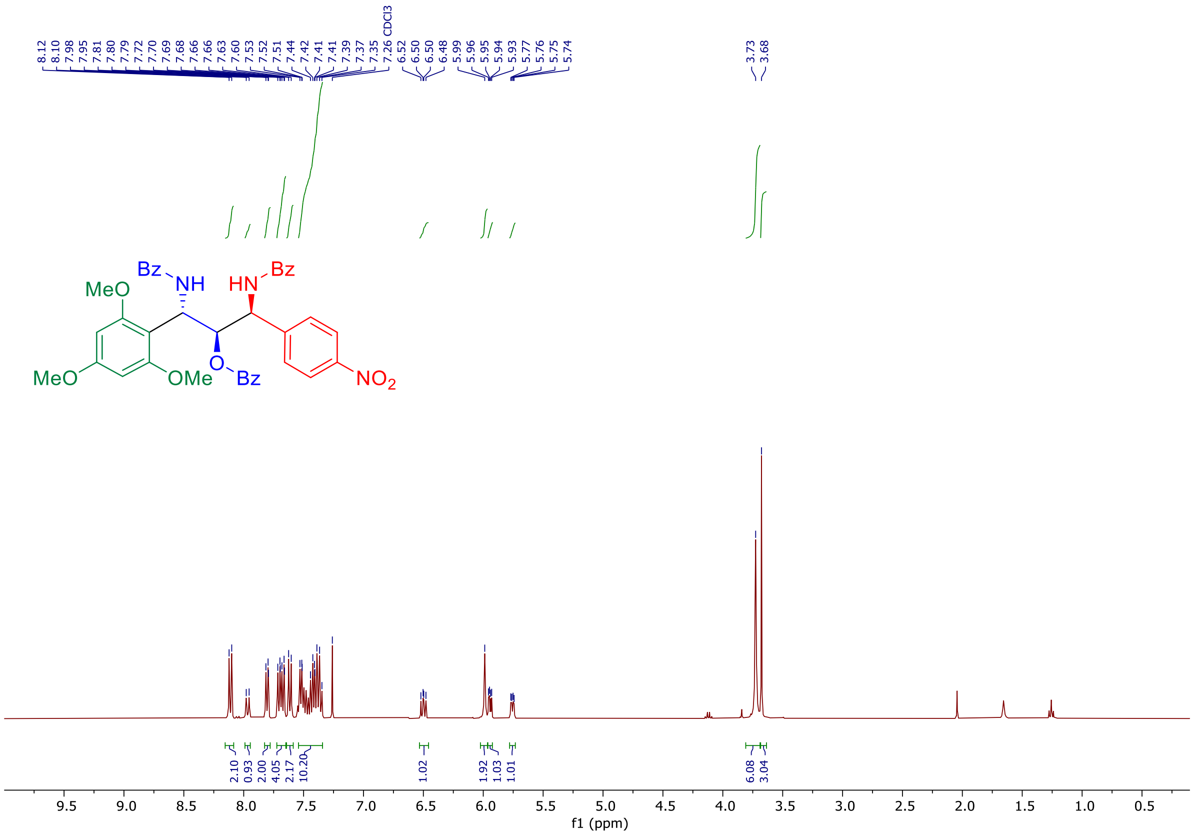

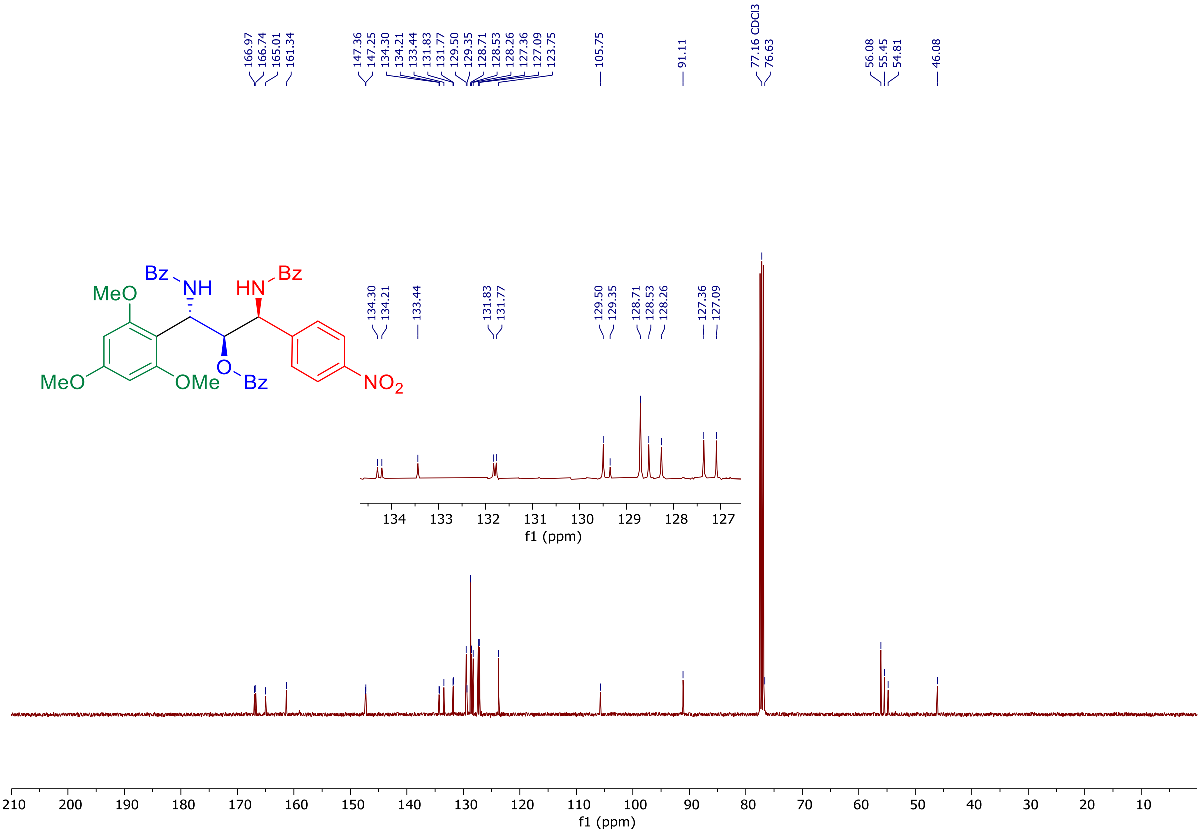

*Figure 41: ^{1}H (400 MHz) and ^{13}C (101 MHz) NMR spectra of **8e** in $CDCl_3$.*

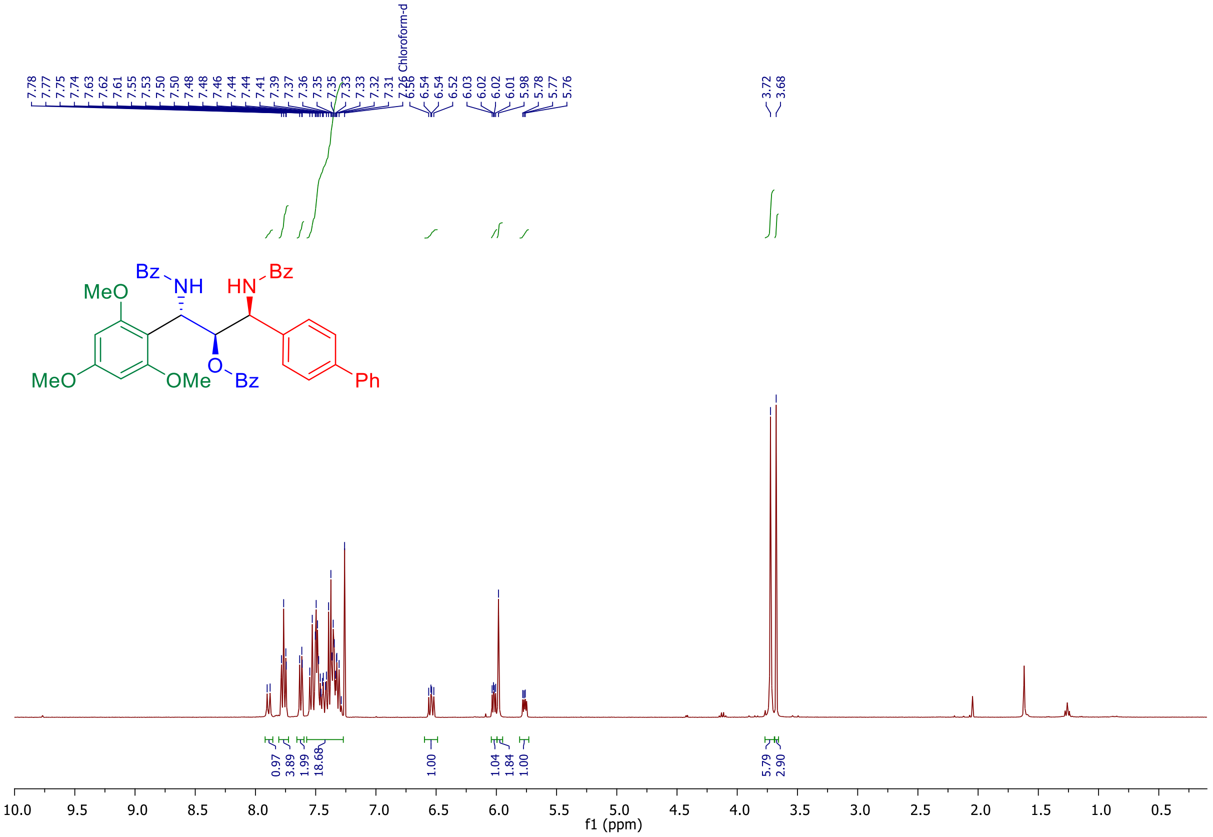

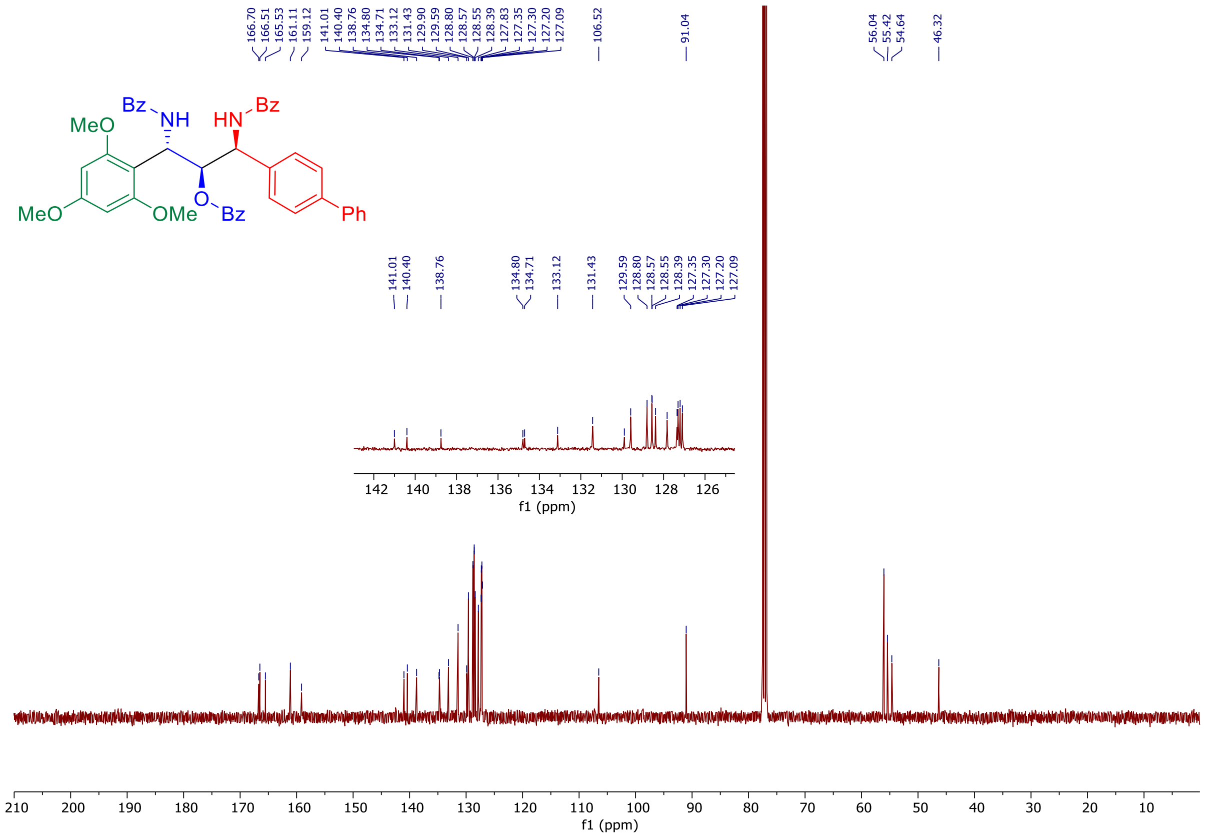

Figure 42: ^{1}H (400 MHz) and ^{13}C (101 MHz) NMR spectra of **8f** in $CDCl_3$.

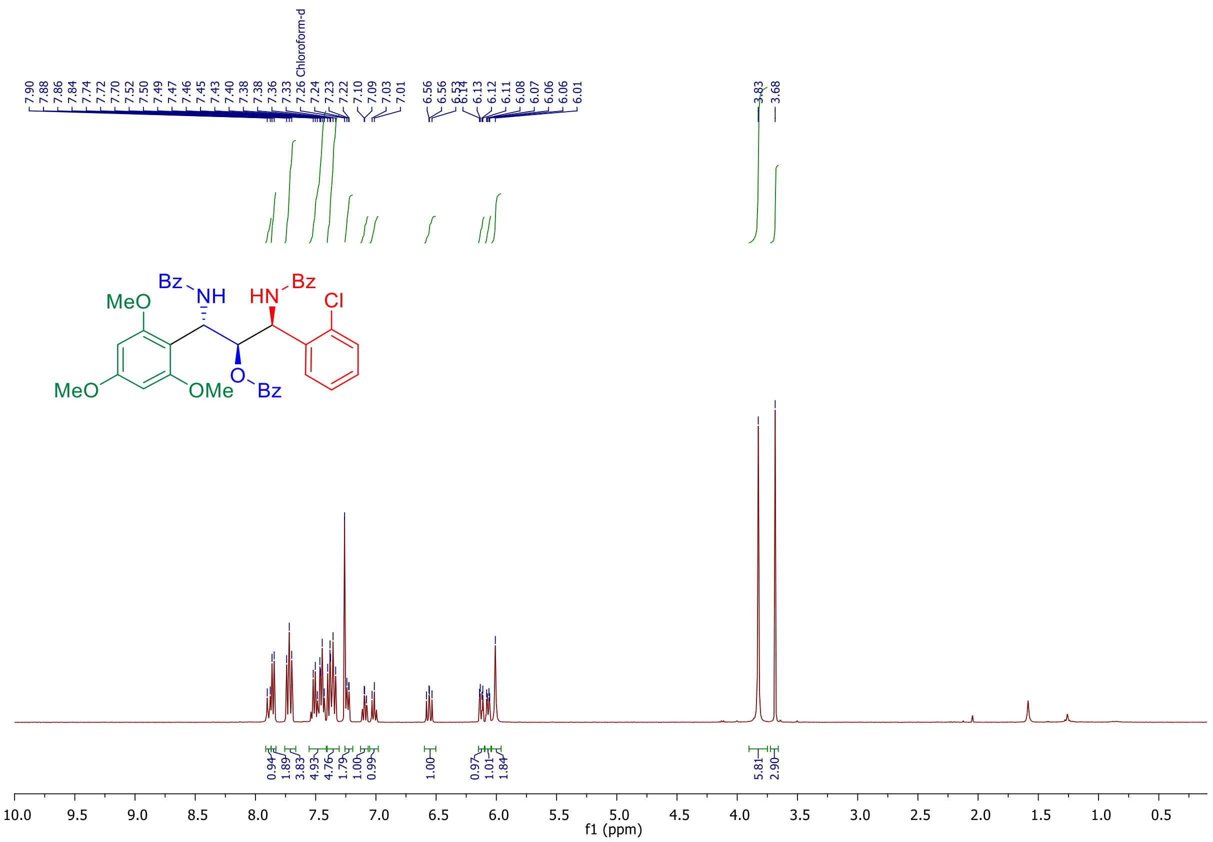

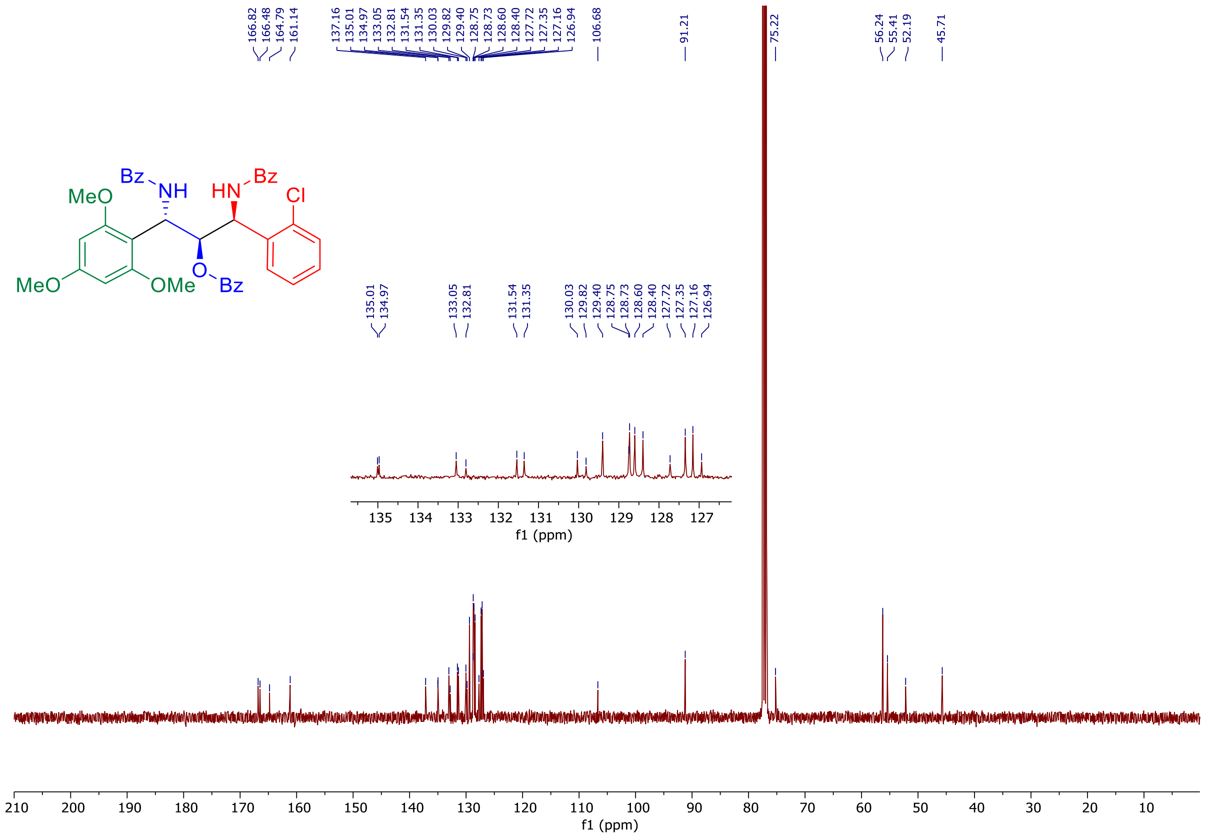

*Figure 43: ^{1}H (400 MHz) and ^{13}C (101 MHz) NMR spectra of **8g** in $CDCl_3$.*

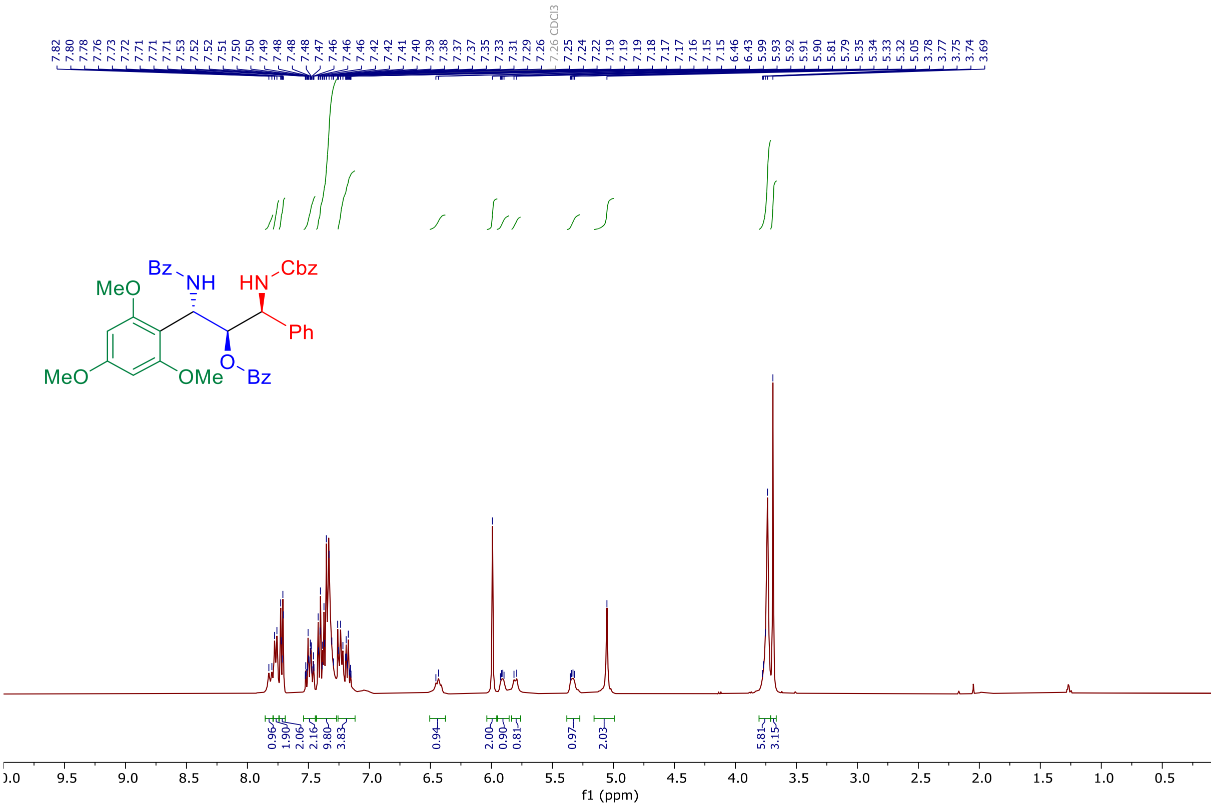

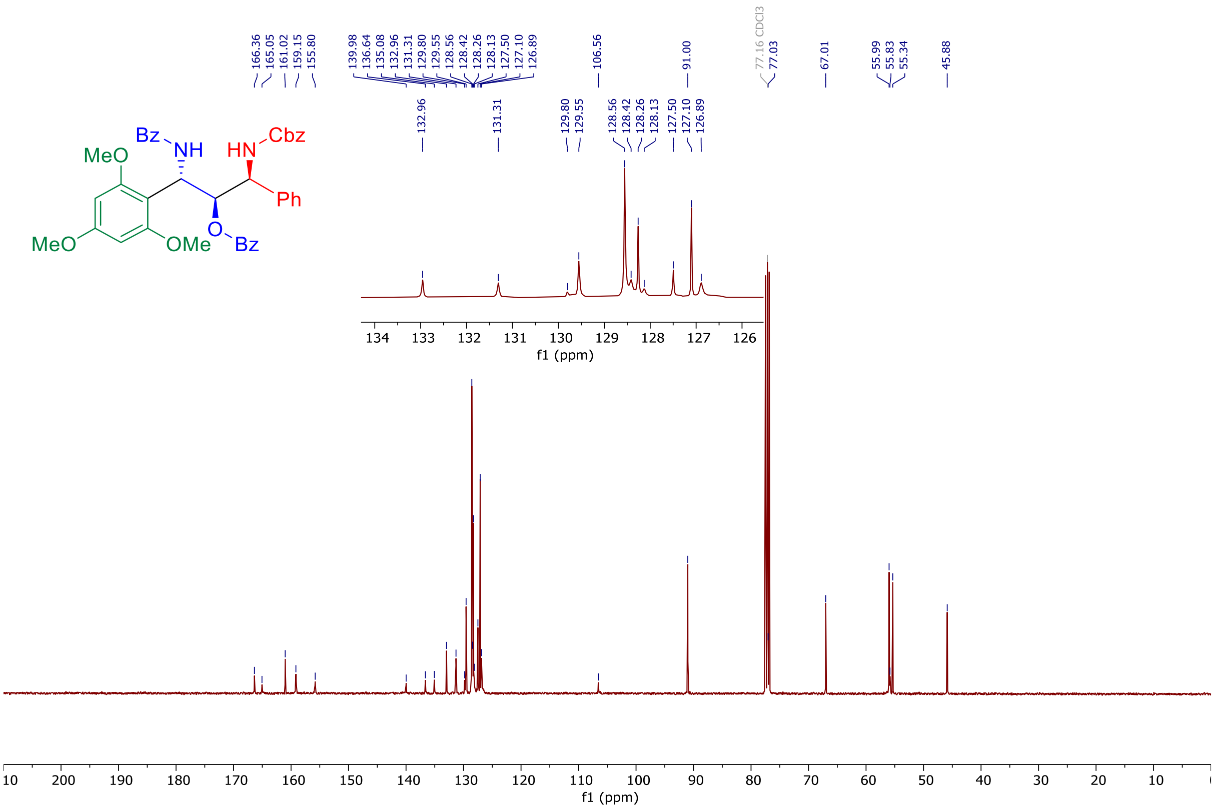

*Figure 44: ^{1}H (400 MHz) and ^{13}C (101 MHz) NMR spectra of **8h** in $CDCl_3$.*

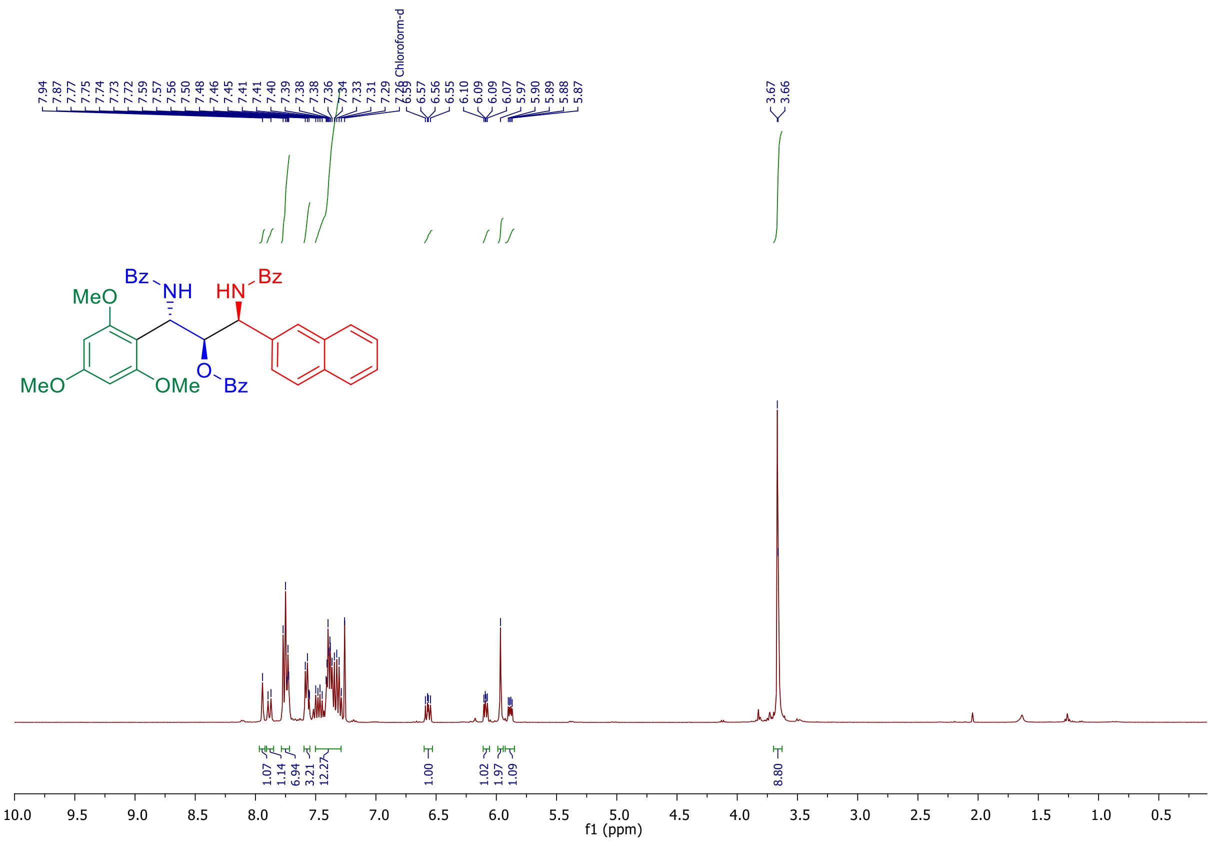

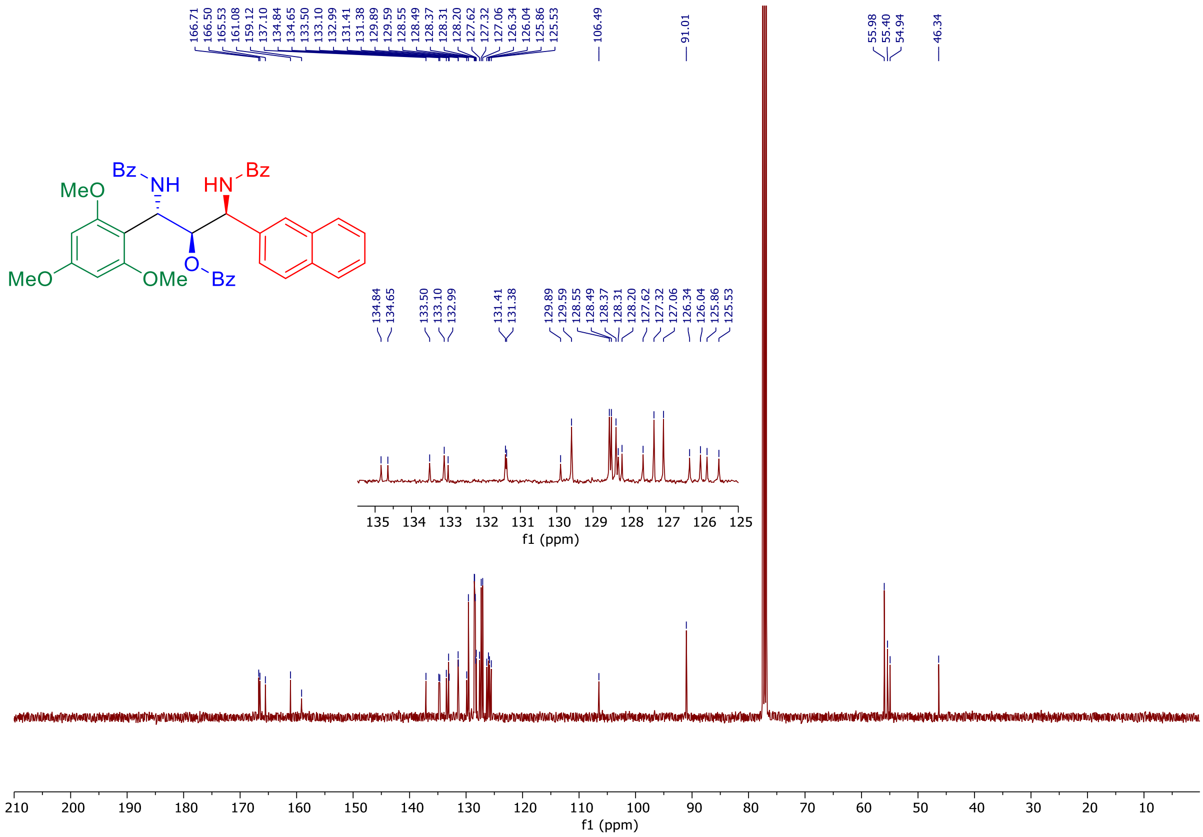

*Figure 45: ^{1}H (400 MHz) and ^{13}C (101 MHz) NMR spectra of **8i** in $CDCl_3$.*

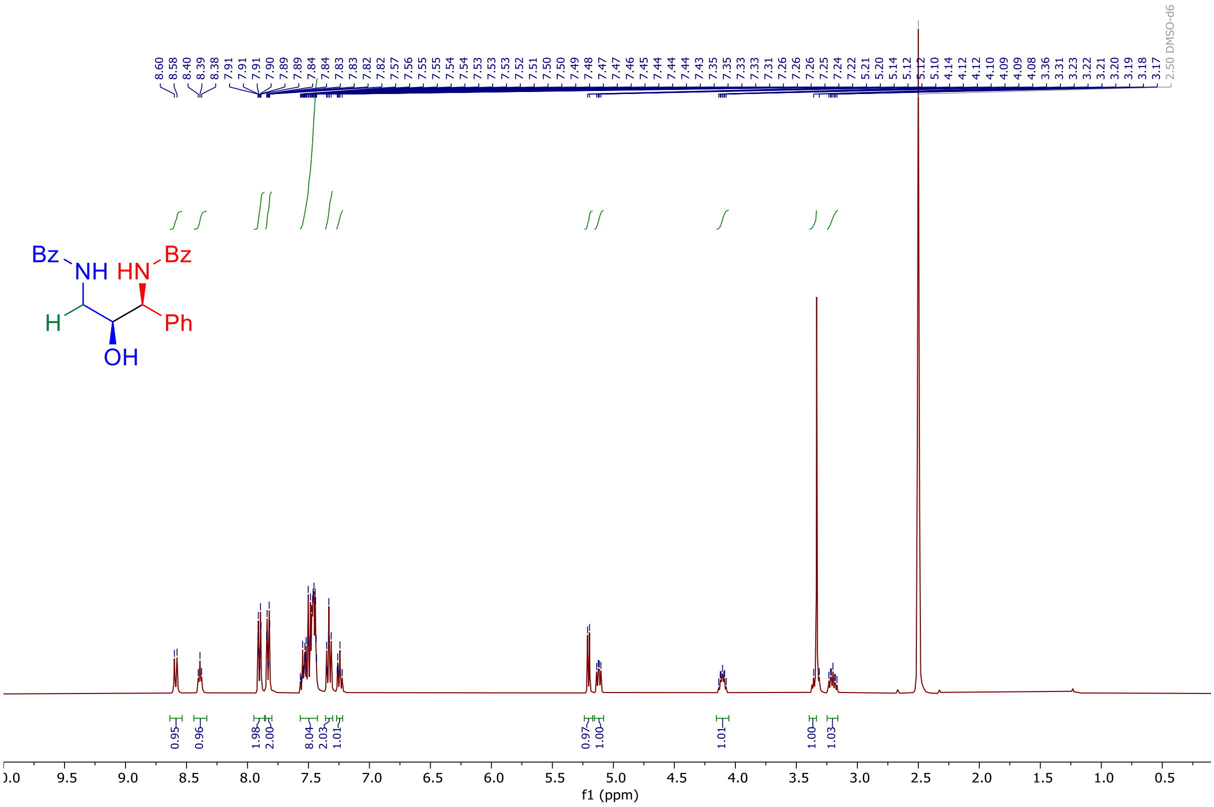

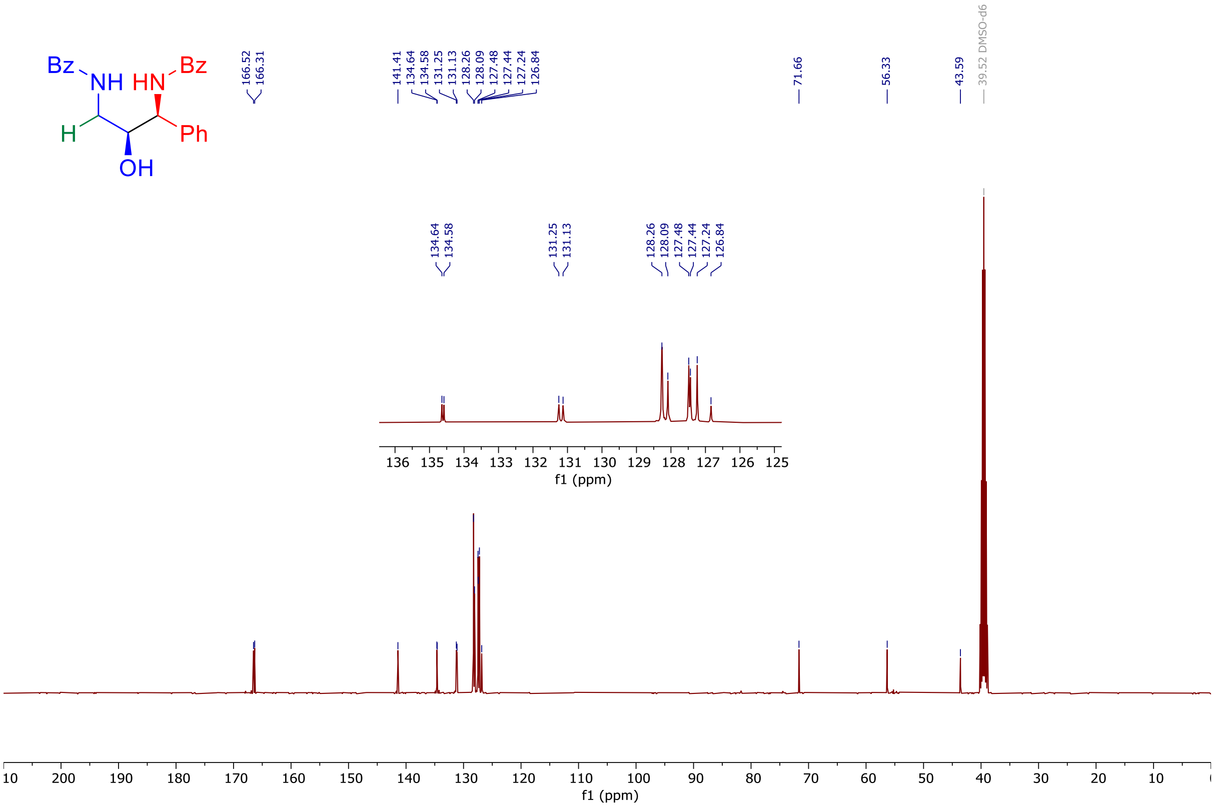

Figure 46: ^{1}H (400 MHz) and ^{13}C (101 MHz) NMR spectra of **9a** in in DMSO-d_6.

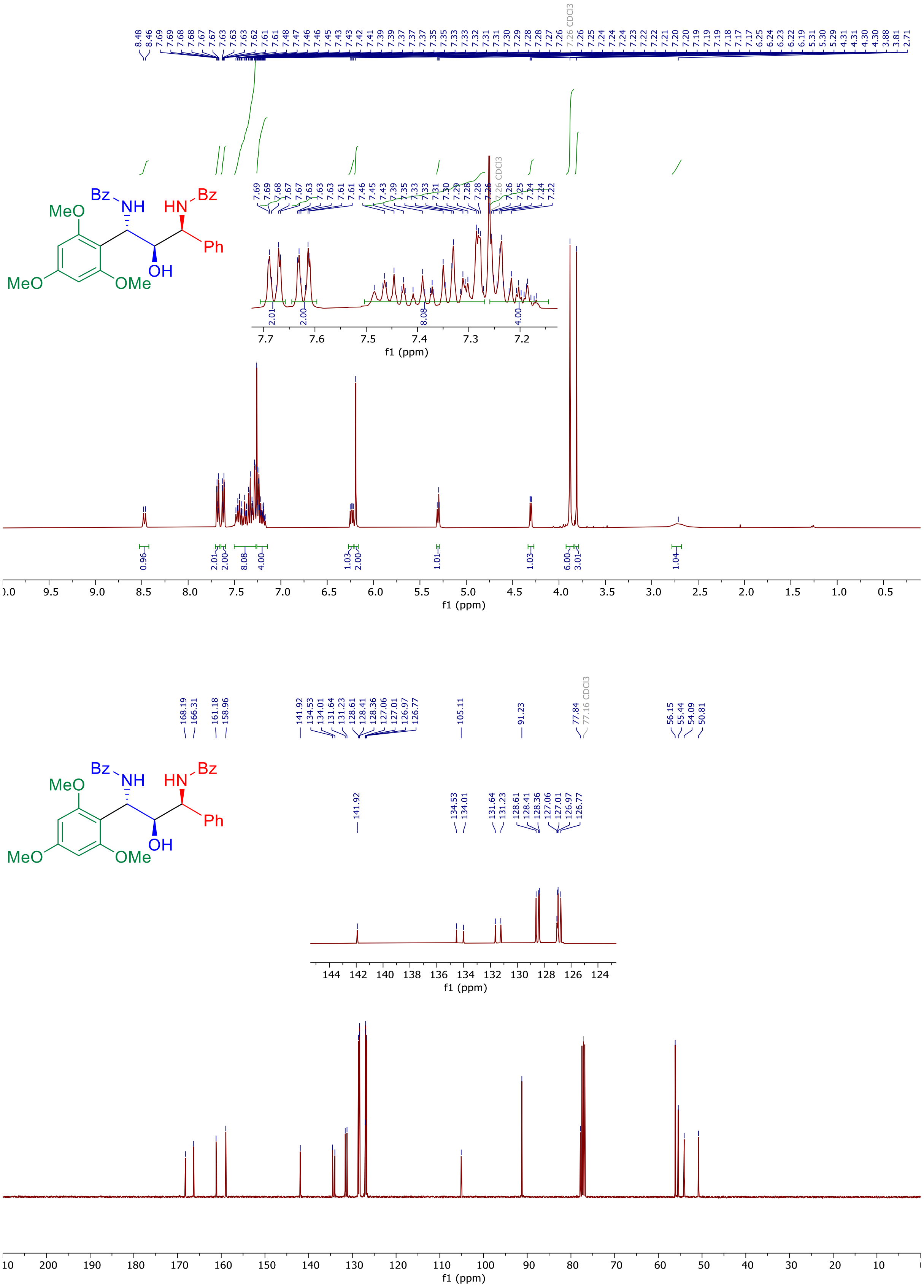

Figure 47: ^{1}H (400 MHz) and ^{13}C (101 MHz) NMR spectra of **9b** in $CDCl_3$.

4 X-ray Data

Crystal structure determinations.

Data for **1c**, **5b** and **5c** (CCDC 2087484, 2087485, 2087486) were collected on a STOE IPDS II two-circle diffractometer with a Genix Microfocus tube with mirror optics using MoKα radiation (λ = 0.71073 Å). The data were scaled using the frame scaling procedure in the X-AREA program system (Stoe & Cie, 2002)[2]. The structures were solved by direct methods using the program SHELXS-2014/6 and refined against F^2 with full-matrix least-squares techniques using the program SHELXL-2014/6.[3]

The crystal of **3c** was an extremely thin weakly diffracting needle. All H atoms were geometrically positioned and refined using a riding model.

In **6b** and in **6c**, the H atoms bonded to N were freely refined.

Data for **6a**, **7a**, **7c**, **7g**, **7h** and **7i** (CCDC 2097900, 2097895, 2097896, 2097898, 2097987, 2097899) were collected at 150.0(1) K on a Gemini S Ultra by Rigaku Oxford Diffraction, equipped with a molybdenum (λ = 0.71073 Å) and a copper (λ = 1.54184 Å) radiation source and a low-temperature control device. Due to the positioning of the two sources in the device, data collection is somewhat limited to smaller angles, which may result in alerts in some checkcif files. Absorption correction was done with CrysAlis Pro 1.171.38.41 and 1.171.40.67a, respectively. All structures were solved using the software programs SHELXS-2018/3, and the positions of all non-hydrogen atoms were refined with SHELXL-2018/3.[3]

In **6a**, **7a**, **7c**, **7g**, **7h** and **7i** the hydrogen atoms were calculated and refined using a riding model and isotropic thermal parameters.

In structure **7a** chloroform and water were embedded. In structure **7h** dichlormethane was embedded. Due to multiple misplacements and an unsatisfying solution a SQUEEZE[10] calculation was performed before refinement with SHELXL-2018/3.

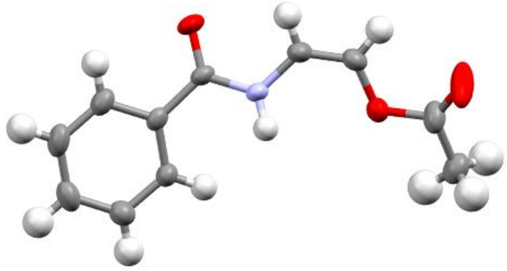

Table 1. Crystal data and structure refinement for **1c**. Displacement ellipsoids are shown at the 50% probability level.

Identification code	CCDC 2087484	
Empirical formula	$C_{11} H_{11} N O_3$	
Formula weight	205.21	
Temperature	173(2) K	
Wavelength	0.71073 Å	
Crystal system	Orthorhombic	
Space group	*P b c a*	
Unit cell dimensions	a = 9.3400(7) Å	α= 90°.
	b = 9.8923(10) Å	β= 90°.
	C = 22.655(3) Å	γ = 90°.
Volume	2093.2(4) $Å^3$	
Z	8	
Density (calculated)	1.302 Mg/m^3	
Absorption coefficient	0.096 mm^{-1}	
F(000)	864	
Crystal size	0.260 x 0.190 x 0.110 mm^3	
Theta range for data collection	2.827 to 25.487°.	
Index ranges	-11<=h<=9, -10<=k<=11, -27<=l<=22	
Reflections collected	4914	
Independent reflections	1912 [R(int) = 0.0418]	
Completeness to theta = 25.000°	99.6 %	
Absorption correction	Semi-empirical from equivalents	
Max. and min. transmission	1.000 and 0.800	
Refinement method	Full-matrix least-squares on F^2	
Data / restraints / parameters	1912 / 0 / 141	
Goodness-of-fit on F^2	0.985	
Final R indices [I>2sigma(I)]	R1 = 0.0540, wR2 = 0.1310	
R indices (all data)	R1 = 0.0754, wR2 = 0.1412	
Largest diff. peak and hole	0.318 and -0.301 e.$Å^{-3}$	

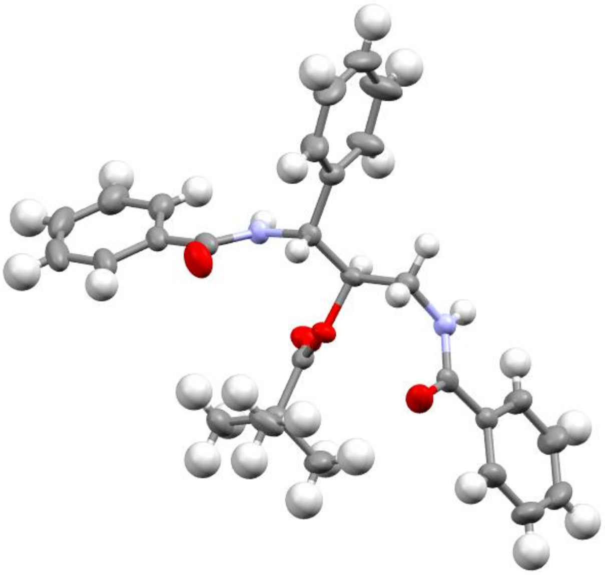

Table 2. Crystal data and structure refinement for **5b**. Displacement ellipsoids are shown at the 50% probability level.

Identification code	CCDC 2087485	
Empirical formula	$C_{28} H_{30} N_2 O_4$	
Formula weight	458.54	
Temperature	173(2) K	
Wavelength	0.71073 Å	
Crystal system	Orthorhombic	
Space group	*P b c a*	
Unit cell dimensions	a = 20.4044(7) Å	α= 90°.
	b = 19.7803(10) Å	β= 90°.
	c = 24.9582(9) Å	γ = 90°.
Volume	10073.3(7) Å^3	
Z	16	
Density (calculated)	1.209 Mg/m^3	
Absorption coefficient	0.081 mm^{-1}	
F(000)	3904	
Crystal size	0.180 x 0.090 x 0.040 mm^3	
Theta range for data collection	2.172 to 25.027°.	
Index ranges	-24<=h<=24, -21<=k<=23, -29<=l<=29	
Reflections collected	91129	
Independent reflections	8890 [R(int) = 0.1172]	
Completeness to theta = 25.000°	99.9 %	
Absorption correction	Semi-empirical from equivalents	
Max. and min. transmission	1.000 and 0.787	
Refinement method	Full-matrix least-squares on F^2	
Data / restraints / parameters	8890 / 0 / 629	
Goodness-of-fit on F^2	0.994	
Final R indices [I>2sigma(I)]	R1 = 0.0568, wR2 = 0.0946	
R indices (all data)	R1 = 0.1052, wR2 = 0.1092	
Largest diff. peak and hole	0.141 and -0.166 e.Å^{-3}	

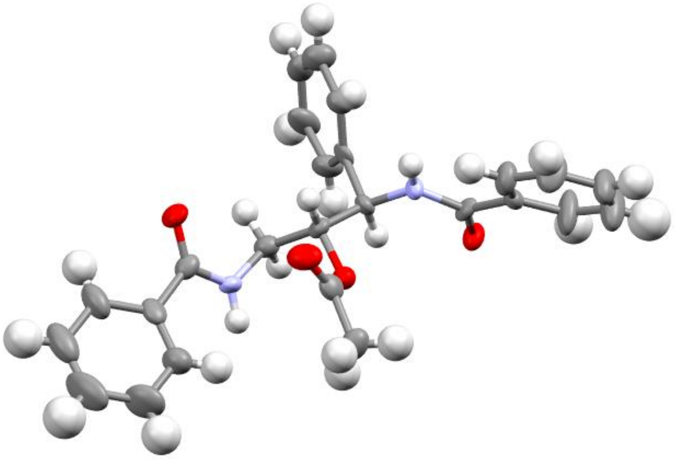

Table 3. Crystal data and structure refinement for **5c**. Displacement ellipsoids are shown at the 50% probability level.

Identification code	CCDC 2087486	
Empirical formula	$C_{25} H_{24} N_2 O_4$	
Formula weight	416.46	
Temperature	173(2) K	
Wavelength	0.71073 Å	
Crystal system	Monoclinic	
Space group	P 21/c	
Unit cell dimensions	a = 14.266(4) Å	α= 90°.
	b = 16.785(4) Å	β= 97.40(2)°.
	c = 9.785(3) Å	γ = 90°.
Volume	2323.6(11) $Å^3$	
Z	4	
Density (calculated)	1.191 Mg/m^3	
Absorption coefficient	0.081 mm^{-1}	
F(000)	880	
Crystal size	0.130 x 0.020 x 0.010 mm^3	
Theta range for data collection	3.209 to 25.025°.	
Index ranges	-16<=h<=15, -19<=k<=18, -11<=l<=11	
Reflections collected	15515	
Independent reflections	4096 [R(int) = 0.2581]	
Completeness to theta = 25.000°	99.8 %	
Absorption correction	Semi-empirical from equivalents	
Max. and min. transmission	1.000 and 0.511	
Refinement method	Full-matrix least-squares on F^2	
Data / restraints / parameters	4096 / 0 / 281	
Goodness-of-fit on F^2	0.811	
Final R indices [I>2sigma(I)]	R1 = 0.0904, wR2 = 0.1108	
R indices (all data)	R1 = 0.2971, wR2 = 0.1816	
Largest diff. peak and hole	0.195 and -0.177 e.$Å^{-3}$	

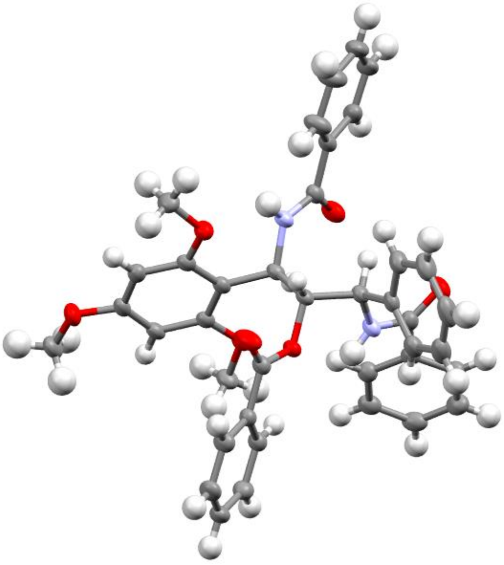

Table 4. Crystal data and structure refinement for **6a**. Displacement ellipsoids are shown at the 50% probability level.

Identification code	CCDC 2097900	
Empirical formula	$C_{39} H_{36} N_2 O_7$	
Formula weight	644.70	
Temperature	150(2) K	
Wavelength	1.54184 Å	
Crystal system	Monoclinic	
Space group	P 21/n	
Unit cell dimensions	a = 16.4179(2) Å	α= 90°.
	b = 12.60170(10) Å	β= 112.839(2)°.
	c = 17.2107(2) Å	γ = 90°.
Volume	3281.61(8) Å^3	
Z	4	
Density (calculated)	1.305 Mg/m^3	
Absorption coefficient	0.732 mm^{-1}	
F(000)	1360	
Crystal size	0.440 x 0.330 x 0.320 mm^3	
Theta range for data collection	3.159 to 62.686°.	
Index ranges	-18<=h<=17, -14<=k<=14, -17<=l<=19	
Reflections collected	28318	
Independent reflections	5249 [R(int) = 0.0219]	
Completeness to theta = 62.686°	99.8 %	
Absorption correction	Semi-empirical from equivalents	
Max. and min. transmission	1.00000 and 0.73913	
Refinement method	Full-matrix least-squares on F^2	
Data / restraints / parameters	5249 / 0 / 443	
Goodness-of-fit on F^2	1.043	
Final R indices [I>2sigma(I)]	R1 = 0.0318, wR2 = 0.0826	
R indices (all data)	R1 = 0.0338, wR2 = 0.0841	
Extinction coefficient	0.00205(13)	
Largest diff. peak and hole	0.184 and -0.146 e.Å^{-3}	

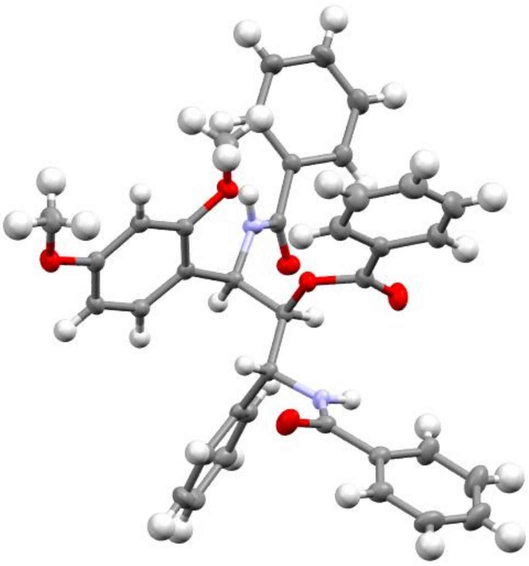

Table 5. Crystal data and structure refinement for **7a**. Displacement ellipsoids are shown at the 50% probability level.

Identification code	CCDC 2097895	
Empirical formula	$C_{38} H_{35} N_2 O_6$	
Formula weight	734.04	
Temperature	150(2) K	
Wavelength	1.54184 Å	
Crystal system	Triclinic	
Space group	P -1	
Unit cell dimensions	a = 11.5431(6) Å	α= 92.986(4)°.
	b = 13.0136(5) Å	β= 110.690(5)°.
	c = 13.9496(8) Å	γ = 112.077(5)°.
Volume	1774.80(17) $Å^3$	
Z	2	
Density (calculated)	1.374 Mg/m^3	
Absorption coefficient	2.752 mm^{-1}	
F(000)	764	
Crystal size	0.350 x 0.300 x 0.210 mm^3	
Theta range for data collection	3.467 to 62.694°.	
Index ranges	-12<=h<=13, -13<=k<=14, -16<=l<=15	
Reflections collected	13753	
Independent reflections	5637 [R(int) = 0.0185]	
Completeness to theta = 62.694°	99.2 %	
Absorption correction	Analytical	
Max. and min. transmission	0.613 and 0.450	
Refinement method	Full-matrix least-squares on F^2	
Data / restraints / parameters	5637 / 31 / 472	
Goodness-of-fit on F^2	1.047	
Final R indices [I>2sigma(I)]	R1 = 0.0467, wR2 = 0.1292	
R indices (all data)	R1 = 0.0490, wR2 = 0.1315	
Extinction coefficient	n/a	
Largest diff. peak and hole	0.353 and -0.764 e.$Å^{-3}$	

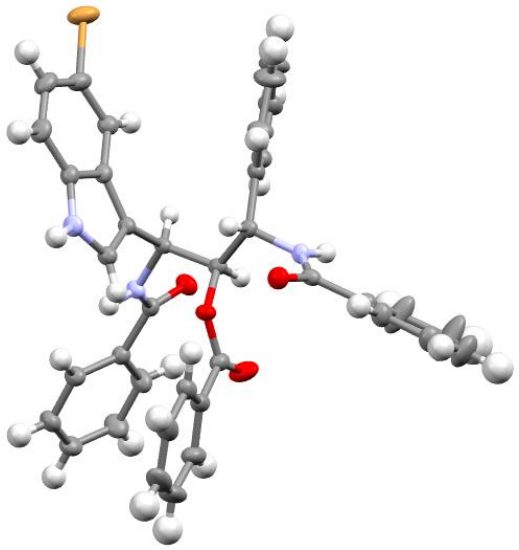

Table 6. Crystal data and structure refinement for **7c**. Displacement ellipsoids are shown at the 50% probability level.

Identification code	CCDC 2097896	
Empirical formula	$C_{38} H_{30} Br N_3 O_4$	
Formula weight	672.56	
Temperature	150(2) K	
Wavelength	1.54184 Å	
Crystal system	Triclinic	
Space group	P -1	
Unit cell dimensions	a = 12.3348(7) Å	α= 61.803(6)°.
	b = 12.8057(8) Å	β= 65.982(5)°.
	c = 13.5088(6) Å	γ = 63.355(6)°.
Volume	1628.5(2) Å^3	
Z	2	
Density (calculated)	1.372 Mg/m^3	
Absorption coefficient	2.084 mm^{-1}	
F(000)	692	
Crystal size	0.320 x 0.140 x 0.060 mm^3	
Theta range for data collection	3.832 to 62.717°.	
Index ranges	-14<=h<=14, -14<=k<=14, -15<=l<=15	
Reflections collected	12558	
Independent reflections	5177 [R(int) = 0.0332]	
Completeness to theta = 62.717°	99.0 %	
Absorption correction	Analytical	
Max. and min. transmission	0.885 and 0.615	
Refinement method	Full-matrix least-squares on F^2	
Data / restraints / parameters	5177 / 0 / 415	
Goodness-of-fit on F^2	1.245	
Final R indices [I>2sigma(I)]	R1 = 0.0376, wR2 = 0.1265	
R indices (all data)	R1 = 0.0448, wR2 = 0.1301	
Extinction coefficient	n/a	
Largest diff. peak and hole	0.769 and -0.729 e.Å^{-3}	

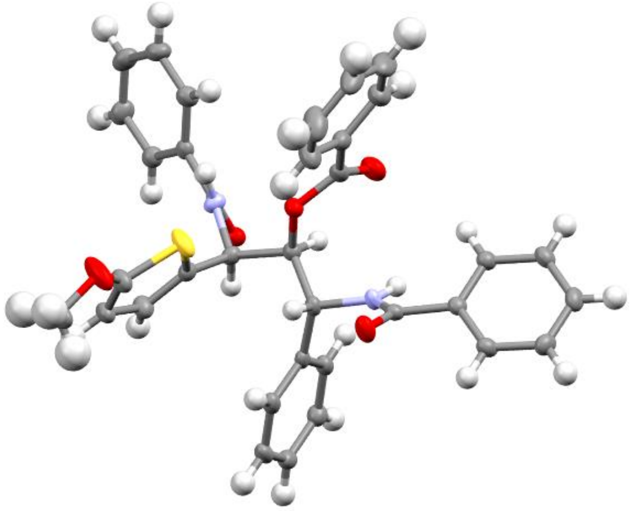

Table 7. Crystal data and structure refinement for **7g**. Displacement ellipsoids are shown at the 50% probability level.

Identification code	CCDC 2097898	
Empirical formula	$C_{35} H_{30} N_2 O_5 S$	
Formula weight	590.67	
Temperature	150(2) K	
Wavelength	1.54184 Å	
Crystal system	Monoclinic	
Space group	P 21/c	
Unit cell dimensions	a = 12.2754(2) Å	α= 90°.
	b = 11.0203(2) Å	β= 96.455(2)°.
	c = 22.3353(5) Å	γ = 90°.
Volume	3002.33(10) $Å^3$	
Z	4	
Density (calculated)	1.307 Mg/m^3	
Absorption coefficient	1.333 mm^{-1}	
F(000)	1240	
Crystal size	0.340 x 0.220 x 0.110 mm^3	
Theta range for data collection	3.624 to 62.722°.	
Index ranges	-14<=h<=12, -12<=k<=11, -25<=l<=23	
Reflections collected	13700	
Independent reflections	4777 [R(int) = 0.0252]	
Completeness to theta = 62.722°	99.4 %	
Absorption correction	Semi-empirical from equivalents	
Max. and min. transmission	1.00000 and 0.30403	
Refinement method	Full-matrix least-squares on F^2	
Data / restraints / parameters	4777 / 0 / 389	
Goodness-of-fit on F^2	1.028	
Final R indices [I>2sigma(I)]	R1 = 0.0374, wR2 = 0.0916	
R indices (all data)	R1 = 0.0411, wR2 = 0.0946	
Extinction coefficient	n/a	
Largest diff. peak and hole	0.412 and -0.574 e.$Å^{-3}$	

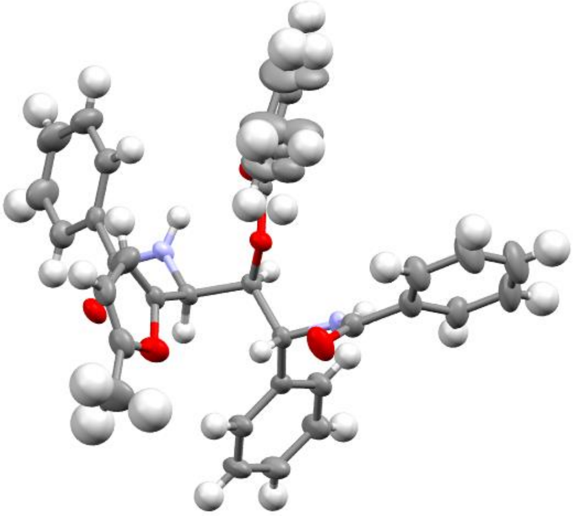

Table 8. Crystal data and structure refinement for **7h**. Displacement ellipsoids are shown at the 50% probability level.

Identification code	CCDC 2097897	
Empirical formula	$C_{35} H_{30} N_2 O_5$	
Formula weight	558.61	
Temperature	150(2) K	
Wavelength	1.54184 Å	
Crystal system	Monoclinic	
Space group	P 21/c	
Unit cell dimensions	a = 10.2985(3) Å	α= 90°.
	b = 26.7638(6) Å	β= 111.086(4)°.
	c = 12.0788(4) Å	γ = 90°.
Volume	3106.32(17) $Å^3$	
Z	4	
Density (calculated)	1.194 Mg/m^3	
Absorption coefficient	0.648 mm^{-1}	
F(000)	1176	
Crystal size	0.420 x 0.210 x 0.080 mm^3	
Theta range for data collection	3.303 to 62.723°.	
Index ranges	-11<=h<=11, -30<=k<=30, -12<=l<=13	
Reflections collected	13211	
Independent reflections	4961 [R(int) = 0.0249]	
Completeness to theta = 62.723°	99.7 %	
Absorption correction	Semi-empirical from equivalents	
Max. and min. transmission	1.00000 and 0.57116	
Refinement method	Full-matrix least-squares on F^2	
Data / restraints / parameters	4961 / 75 / 436	
Goodness-of-fit on F2	1.038	
Final R indices [I>2sigma(I)]	R1 = 0.0472, wR2 = 0.1238	
R indices (all data)	R1 = 0.0529, wR2 = 0.1286	
Extinction coefficient	0.00112(14)	
Largest diff. peak and hole	0.715 and -0.248 e.$Å^{-3}$	

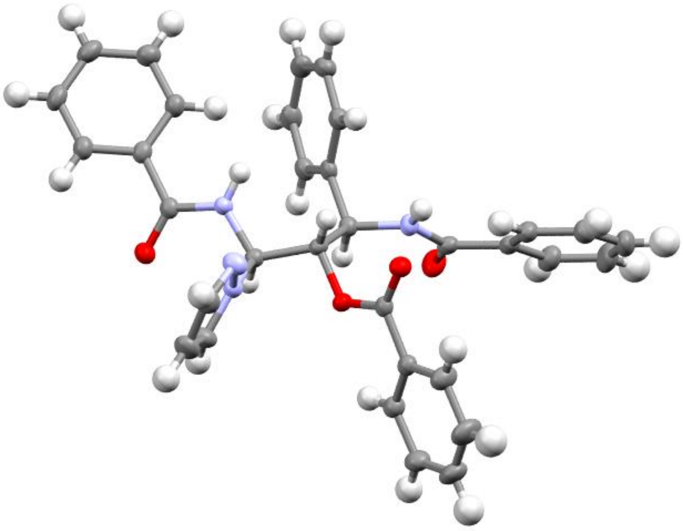

Table 9. Crystal data and structure refinement for **7i**. Displacement ellipsoids are shown at the 50% probability level.

Identification code	CCDC 2097899	
Empirical formula	$C_{33} H_{28} N_4 O_4$	
Formula weight	544.59	
Temperature	150(2) K	
Wavelength	1.54184 Å	
Crystal system	Monoclinic	
Space group	P 21/n	
Unit cell dimensions	a = 10.8901(2) Å	α= 90°.
	b = 15.3683(3) Å	β= 98.621(2)°.
	c = 16.9357(4) Å	γ = 90°.
Volume	2802.37(10) Å^3	
Z	4	
Density (calculated)	1.291 Mg/m^3	
Absorption coefficient	0.698 mm^{-1}	
F(000)	1144	
Crystal size	0.480 x 0.070 x 0.070 mm^3	
Theta range for data collection	3.904 to 62.732°.	
Index ranges	-9<=h<=12, -17<=k<=17, -17<=l<=19	
Reflections collected	11769	
Independent reflections	4484 [R(int) = 0.0255]	
Completeness to theta = 62.732°	99.5 %	
Absorption correction	Semi-empirical from equivalents	
Max. and min. transmission	1.00000 and 0.90782	
Refinement method	Full-matrix least-squares on F^2	
Data / restraints / parameters	4484 / 0 / 370	
Goodness-of-fit on F^2	1.049	
Final R indices [I>2sigma(I)]	R1 = 0.0352, wR2 = 0.0876	
R indices (all data)	R1 = 0.0403, wR2 = 0.0917	
Extinction coefficient	n/a	
Largest diff. peak and hole	0.332 and -0.253 e.Å^{-3}	

5 References

[1] J. Halli, K. Hofman, T. Beisel, G. Manolikakes *Eur. J. Org. Chem*. **2015**, 4624–4627.

[2] Stoe & Cie, X-AREA. Diffractometer control program system. Stoe & Cie, Darmstadt, Germany, 2002.

[3] G. M. Sheldrick, *Acta Cryst. Sect. A*, **2008**, *64*, 112-122.

[4] M. Mroczkiewicz, R. Ostaszewski, *Tetrahedron* **2009**, 65, 4025–4034.

[5] T. W. Price, G. Firth, C. J. Eling, M. Kinnon, N. J. Long, J. Sturgea, G. J. Stasiuk, *Chem. Commun*. **2018**, *54*, 3227.

[6] D. J. Miller, M. Bashir-Uddin Surfraz, M. Akhtar, D. Gani, R. K. Allemann, *Org. Biomol. Chem*. **2004**, *2*, 671–688.

[7] B. Bernardim, L. D. Lordello, A. C.B. Burtoloso, *Curr. Top. Med. Chem.* **2013**, *13*, 2099-2103.

[8] Y. Bendiabdellah, I. Villanueva-Margalef, A. Misale, K. S. Nahar, M. R. Haque, D. E. Thurston, G. Zinzalla, Synthesis **2011**, *14*, 2321-2333.

[9] G. Zhang, R. Huang *RSC Adv.* **2016**, *6*, 6768.

[10] A. L. Spek, *Acta Cryst. Sect. D*, **2009**, 65, 148-155.
